# Strong Implications From Small Deviations in Labeling Patterns: The Mechanism of *Burkholderia gladioli* Pacifigorgiadiene Synthase

**DOI:** 10.1002/chem.202600029

**Published:** 2026-02-10

**Authors:** Zhiyong Yin, Zhehui Hu, Xiyuan He, Juan Xu, Bernd Goldfuss, Jeroen S. Dickschat, Guangkai Bian

**Affiliations:** ^1^ Kekulé‐Institute For Organic Chemistry and Biochemistry University of Bonn Bonn Germany; ^2^ State Key Laboratory of Quantitative Engineering Biology, Center of Materials Synthetic Biology, Shenzhen Institute of Synthetic Biology Shenzhen Institutes of Advanced Technology Shenzhen P.R. China; ^3^ National Key Laboratory for Germplasm Innovation & Utilization of Horticultural Crops College of Horticulture and Forestry Huazhong Agricultural University Wuhan P.R. China; ^4^ Institute of Organic Chemistry Department of Chemistry University of Cologne Cologne Germany

**Keywords:** biosynthesis, DFT calculations, enzyme mechanisms, isotopes, terpenes

## Abstract

The sesquiterpene synthase BgPgS from *Burkholderia gladioli* produces the main product (–)‐1‐*epi*‐pacifigorgia‐6,10‐diene, besides a few structurally related compounds. The enzyme mechanism of BgPgS was addressed through isotopic labeling experiments, revealing several intricate mechanistic problems. Unexpectedly, the isotopic labelings for the Me groups C12 and C13 occurred in different positions for the main and the side products. Moreover, as demonstrated in this study, two hydrogen atoms must change from the bottom to the top hemisphere, which is not possible through standard terpene biosynthesis routines with suprafacial hydrogen migrations. Our rational solution involves two mechanistic explanations: First, a key rearrangement may be associated with a conformational change that rotates one hydrogen from bottom to top. Second, a “break‐flip‐cyclize” sequence explains the change of side by the other hydrogen. DFT calculations show that the proposed terpene cyclization cascades are energetically feasible; only one problematic activation barrier (>25 kcal/mol) remains. However, several mechanistic alternatives either failed to explain the experimental results of the isotopic labeling experiments or were associated with even higher activation barriers. Our biosynthetic proposal for pacifigorgiadiene biosynthesis can be understood as a contribution that awaits further investigation and scientific debate for its ultimate resolution.

## Graphical abstract

The mechanism of pacifigorgiadiene synthase from *Burkholderia gladioli* was investigated through isotopic labeling experiments and DFT calculations. The results revealed an unexpected labeling pattern which appeared to be different in the main and the side products, requiring unprecedented mechanistic explanations, including a “break‐flip‐cyclise” sequence to set the correct stereochemistry.

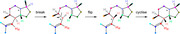

## 1 Introduction

Terpene synthases (TSs) are remarkable enzymes that catalyse the most complex transformations in nature. Class I enzymes [1] convert acyclic and achiral precursors, including geranyl (GPP), farnesyl (FPP), geranylgeranyl (GGPP), and geranylfarnesyl pyrophosphate (GFPP), through diphosphate abstraction, yielding an allyl cation that reacts in a cascade reaction involving cyclisations, rearrangements, hydride and proton migrations, before a terminal deprotonation or water quench produces a terpene hydrocarbon or alcohol.

**SCHEME 1:**
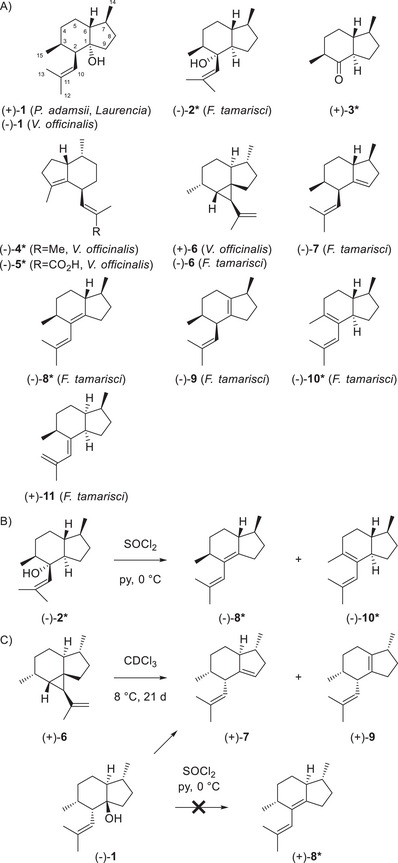
(a) Pacifigorgianes and related sesquiterpenes. (b) Chemical correlation of (–)‐(−)‐2* to (–)‐(−)‐8* and (–)‐(−)‐10*. (c) Chemical correlation of (+)‐**6** and (–)‐(−)‐**1** to (+)‐**7** and (+)‐**9**.

The biosynthesis of many compounds is obvious, but some terpenes pose a biosynthetic riddle. One such case is pacifigorgianes, with ichthyotoxic (+)‐pacifigorgiol (**1**) from the octocoral *Pacifigorgia adamsii* as the first representative (Scheme 1a) [2]. The compound was reisolated from several red algae (*Laurencia*) [3, 4, 5], while (–)‐**1** is known from *Valeriana officinalis* [6]. The second reported compound is (–)‐tamariscol (2*) from the liverwort *Frullania tamarisci* [7], whose absolute configuration has been assigned by degradation to (+)‐3* and comparison to its enantiomer (asterisks indicate compounds of known absolute configuration) [8]. In a subsequent study the structurally related (–)‐valerena‐4(7),11‐diene (4*) from *V. officinalis* was reported, with the absolute configuration assigned based on its biosynthetic relationship to (–)‐valerenic acid (5*) [9]. This assumption was confirmed by chemical correlation of (–)‐5* to (–)‐4* [10]. The same study also disclosed (+)‐tamariscene (**6**) and (–)‐**6** from *V. officinalis* and *F. tamarisci*, respectively. In addition, the liverwort contained a series of pacifigorgianes, including (–)‐pacifigorgia‐1(9),10‐diene (**7**), (–)‐pacifigorgia‐1,10‐diene (8*), (–)‐pacifigorgia‐1(6),10‐diene (**9**), (–)‐pacifigorgia‐2,10‐diene (10*), and pacifigorgia‐2(10),11‐diene (**11**). The absolute configurations of (–)‐8* and (–)‐10* were determined through a correlation with (–)‐**2** (Scheme 1b) [9]. Further experiments showed the conversion of (+)‐**6** into (+)‐**7** and (+)‐**9** upon storage in CHCl_3_, and of (–)‐**1** into the same products with SOCl_2_‐pyridine, but not into (+)‐**8,** requiring *syn* elimination (Scheme 1c) [9]. Because the absolute configurations of (+)‐**6** and (–)‐**1** are unknown [11], these experiments did not firmly establish the absolute configurations of (+)‐**7** and (+)‐**9**, but confirmed that the four compounds represent one enantiomeric series.

Class I plant TSs often produce the opposite enantiomers as formed by microbial TSs, and recent studies uncovered that octocorals make use of microbial‐type TSs [12, 13]. Furthermore, a typical plant TS for 4* has been described from *V. officinalis* [14], and a microbial type TS producing 2* was recently discovered in *F. tamarisci* [15]. This may suggest that (–)‐**1**, (–)‐4* and (+)‐**6** from *V. officinalis* belong to one enantiomeric series with Me14 and Me15 pointing down, whereas compounds from *P. adamsii*, *Laurencia* and *F. tamarisci* may generally belong to the enantiomeric series with these groups pointing up. For C2 and C6 configurations, some flexibility occurs.

Speculations about pacifigorgiane biosynthesis have been published [7, 9, 16], but little experimental evidence is available. A feeding experiment with (1‐^13^C)acetate gave some insights into the biosynthetic origin of the carbon backbone of 4*, further elaborating an early suggestion by Connolly (Scheme S1) [7]. Here we report on the discovery of a TS for pacifigorgianes from *Burkholderia gladioli*. Isotopic labeling experiments and DFT calculations revealed an unexpectedly complex mechanistic problem, whose rationalisation required several iterations to approximate a model consistent with all experimental data.

## 2 Results and Discussion

A phylogenetic tree containing 5667 TS homologs revealed the presence of an uncharacterised enzyme clade in diverse strains of *B. gladioli* (Figure S1). One enzyme of unknown function (WP_196317566) was selected for functional study. The purified protein (Figure S2) was incubated with GPP, FPP, GGPP, and GFPP, revealing efficient conversion only of FPP into several sesquiterpenes (Figures S3 and S4). Four compounds were identified as the main product (–)‐1‐*epi*‐pacifigorgia‐6,10‐diene (**12**), besides (–)‐1,2‐*diepi*‐pacifigorgia‐6,10‐diene (**13**), (–)‐6‐*epi*‐pacifigorgia‐1,10‐diene (**14**), and (–)‐ledene (**15**) (Scheme 2a, Tables S2–S5, and Figures S5–S36). These findings identified the enzyme as *B. gladioli* 1‐*epi*‐Pacifigorgia‐6,10‐diene Synthase (BgPgS). To improve the production of two minor constituents, an alanine scanning of proposed active site residues based on an AlphaFold3 model docked with FPP was performed (Figures S37 and S38 and Table S6). Enhanced production in the G299A variant allowed for isolation of (–)‐pacifigorgia‐1(6),10‐diene (**9**) and (–)‐tamariscene (**6**) (Scheme 2a, Tables S7 and S8, and Figures S39–S54).

**SCHEME 2:**
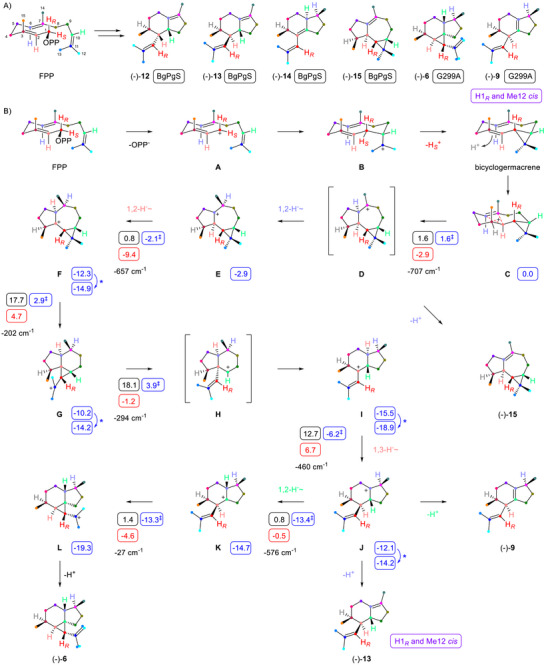
Characterisation of BgPgS. (a) Structures of products and results of labeling experiments. Boxes indicate the source enzyme for compound isolation. (b) Cyclisation mechanism from FPP to **6**, **9**, **13,** and **15**. Results from DFT calculations (wB97M‐V/Def2‐TZVPPD//B97D3/6‐31G(d,p), 298 K) are given in boxes (blue: Gibbs energies, with ‡ denoting free energies of transition states, both relative to **C** set to 0.0 kcal/mol; black: Gibbs reaction barriers; red: free reaction energies; all in kcal/mol). Blue asterisks indicate minor conformational changes between the product of one computed step and the starting structure of the next step. Imaginary frequencies of transition states are given in cm^−1^.

The absolute configurations of **6**, **9,** and **12**–**15** were determined through stereoselective deuteration. The method enzymatically introduces stereoselective deuterations in CH_2_ groups from labeled precursors, setting stereocentres of known configuration [17]. Absolute configuration assignment then simplifies to a problem of relative configuration determination through NOESY. Specifically, DMAPP and (*E*)‐ or (*Z*)‐(4‐^13^C,4‐^2^H)IPP [18] were converted with FPP synthase [19] (FPPS) and BgPgS (Table S9). The additional ^13^C‐label allowed for a sensitive detection of incorporation through HSQC (Figures S55–S60). Additional experiments using (*R*)‐ and (*S*)‐(1‐^13^C,1‐^2^H)IPP [20] with isopentenyl diphosphate isomerase (IDI) [21], FPPS, and BgPgS (Figures S61–S65) established the absolute configurations as in Scheme 2a. These data confirmed unchanged CH_2_ groups at C4, C5, C8, and C9 for all products (for **9** signals at C5 and C9 were not resolved), and indicated the same absolute configuration as for **15** from *Plagiochila yokogurensis* [22]. The absolute configurations of **6**, **9**, and **12**–**14** are newly assigned here.

The biosynthesis of **6**, **9**, and **12**–**15** was investigated through isotopic labeling experiments (Table S9). Carbon origins were determined using the 15 isotopomers of (^13^C)FPP [23], as indicated by the color code in Scheme 2a (Figures S66–S71). The (*E*)‐Me group of FPP (C12) ended up in the *E* position of the dimethylvinyl group of **13**, **14,** and **9**, but in the *Z* position of **12** (and the opposite was observed for C13). Similarly, for **15,** a clear stereochemical course was observed, but a distribution of labeling over both positions was found for **6**. The incubation of (3‐^13^C)FPP in D_2_O revealed incorporation of one deuterium into all six compounds by mass spectrometry (Figure S72). ^13^C‐NMR analysis showed an upfield shifted triplet for C3 of **12** (Δ*δ* = –0.50 ppm, ^1^ *J*_C,D_ = 19.7 Hz), establishing C3 as the site of deuterium incorporation (Figure S73). The same position for deuterium uptake was assumed for the minor compounds. The stereoselective deuterations revealed selective loss of the 1‐*pro*‐*S* hydrogen for all six compounds (Figure S74), while the 1‐*pro*‐*R* hydrogen remains bound to C1 (Figure S75).

The situation for the remaining hydrogen atoms (H2, H6, and H10 of FPP) was different for the individual compounds, requiring a detailed discussion. For **12,** the incubation of (3‐^13^C,2‐^2^H)FPP [24] with BgPgS and product analysis by NMR spectroscopy returned an upfield shifted singlet for C3 (Δ*δ* = –0.10 ppm, Figure S76), typical for deuterium in a neighbouring position. C2 is the only reasonable possibility, thus H2 shows no net migration. Conversion of (2‐^2^H)DMAPP [25] and (2‐^13^C)IPP [26] with FPPS and BgPgS yielded **12** showing upfield shifted doublets for C2 and C6 (Δ*δ* = –0.07 and –0.06 ppm, respectively; the doublets result from ^13^C–^13^C spin coupling between C2 and C6; Figure S77). This is in line with deuterium located at C10, revealing H10 stays at C10. Incubation of (2‐^2^H)GPP [27] with (2‐^13^C)IPP demonstrated loss of H6 in the terminal deprotonation step (Figure S78).

These experiments showed the same fate for H2, H6, and H10 for the most abundant side product **13** (Figures S79–S81). For **15** H2 was localised at C2, and the loss of H6 was demonstrated (Figures S82 and S83). Using the G299A variant, the experiment with (3‐^13^C,2‐^2^H)FPP confirmed retainment of H2 at C2 for **6** (Figure S84). Conversion of (2‐^2^H)GPP and (2‐^13^C)IPP with FPPS and BgPgS‐G299A gave a signal of unchanged chemical shift for C2, in agreement with deuterium in a distant position (C7, Figure S85). Localisation of H10 at its likely target position C6 with (2‐^2^H)DMAPP and (2‐^13^C)IPP was impossible, because the expected triplet signal for C6 of **6** was too weak, but the combination of (2‐^2^H)DMAPP and (3‐^13^C)IPP [28] confirmed incorporation of H10 at C6 by a slight upfield shift for C7 (Figure S86). For **9,** the G299A variant was used to confirm retainment of H2 at C2 (Figure S87), while H6 migrated into a position without influence on the chemical shift of C2 (→C7, Figure S88), and H10 was lost (Figure S89). For **14** retainment of H6 and H10, but loss of H2, was confirmed through deuteration experiments with BgPgS and product analysis by GC/MS (Figure S90). H6 likely resides at C7 and H10 at C6, which best parallels the situation of the other products.

For **6**, **9**, **13,** and **15**, these results align with the reaction mechanism of Scheme 2b, while for **12,** with its different outcome regarding Me12/Me13 incorporations, and for **14,** independent explanations are required (vide infra). The proposed sequence toward the side products starts from FPP in a chair–chair fold with H2 and H6 down (these hydrogens also point down in the side products) and H10 up (as in the side products). Ionisation to **A** and 1,10‐cyclisation to **B** are followed by a deprotonation with loss of the 1‐*pro*‐*S* hydrogen and cyclopropanation to bicyclogermacrene, observed as its Cope rearrangement product bicycloelemene by GC/MS (Figure S3). Reprotonation at the *Re* face of C3 sets the correct stereochemistry in **C** that proceeds in a 2,6‐cyclisation to **D** as precursor of **15**. A 1,2‐hydride shift of H6 to C7 results in **E** that reacts in another 1,2‐hydride migration of H2 to C6 to yield **F**. A cyclopropylcarbinyl rearrangement results in **G** that undergoes ring opening to **H** and rearrangement to **I**. A subsequent 1,3‐hydride shift of H2 back to C2 leads to **J** representing the precursor of **9** and **13**, while the biosynthesis of **6** requires another 1,2‐hydride shift of H10 to C6, yielding **K**, a ring closure to **L**, and deprotonation. Intermediate **I** could serve as a precursor to **14**, but only with loss of H10, and not of H2 as observed. Conclusively, **14** requires an alternative mechanism.

The proposed cyclisation cascade was further investigated through DFT calculations (Table S10 and Figure S91), identifying **C** as a cation‐π complex. Indeed, conversion into **E** is a single‐step process with skipping of **D** associated with a low activation barrier (1.6 kcal/mol). The 1,2‐hydride shift to **F** also has a very low barrier, but the subsequent rearrangements and 1,3‐hydride shift to **J** show substantially higher activation barriers of ca. 12–18 kcal/mol. Nevertheless, such data are in the range for an enzyme‐catalysed reaction, and the whole sequence explains the positions of all carbons and hydrogens.

**SCHEME 3:**
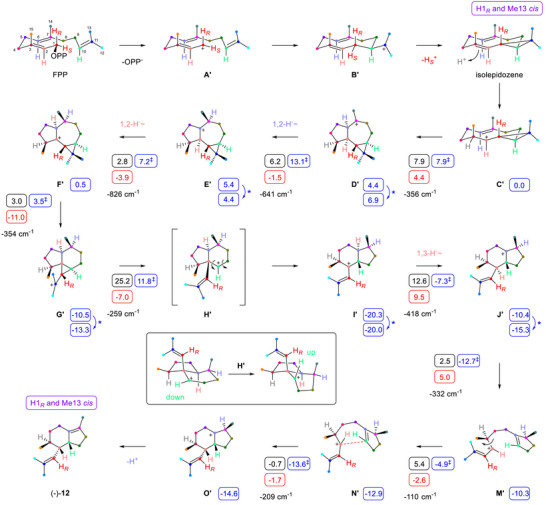
Cyclisation mechanism of BgPgS for the main product **12**. For explanations regarding computational data (small boxes), cf. legend of Scheme 2.

The biosynthesis of **12** provided a complex mechanistic riddle. For this molecule, Me13, not Me12, stands *cis* with respect to the original 1‐*pro*‐*R* hydrogen (H*_R_*) in the dimethylvinyl group (Scheme 3). For this reason, Me13 and H*_R_* should be *cis* in **C’**, and the preceding intermediate should be isolepidozene, a stereoisomer of bicyclogermacrene that is not observed among the enzyme products, possibly because of its efficient downstream conversion. Similar findings were made for geosmin synthase, for which the proposed intermediate isolepidozene is only detected in an enzyme variant, but not in the wild‐type [29]. Consequently, all three hydrogens H2, H6, and H10 should be oriented down in the starting conformation of FPP, but two of these hydrogens (H2 and H10) point up in **12**. For the rigid structures along the cyclisation cascade, no antarafacial hydride shift can be expected, raising the question of how the hydrogen atoms may change from bottom to top. A conformational flip by 180° rotation of the C2═C3 plane of FPP could solve the problem for H2, but this should lead to a conformer of isolepidozene in which protonation at C3 (from the outer *Si* face) results in the wrong configuration at this carbon.

The following mechanism resolves this problem: In FPP, all three hydrogens H2, H6, and H10 are oriented down, leading through **A′** and **B′** to isolepidozene, presenting the correct outside *Re* face at C3. Deprotonation of **B’** with loss of H*_S_* leads to H*_R_* and Me13 in *cis* orientation. Reprotonation at C3 yields **C’** that reacts in a 2,6‐cyclisation to **D’**, followed by two sequential 1,2‐hydride shifts to **E’** and **F’**, and cyclopropylcarbinyl rearrangement to **G’**. At this stage, H2 and H10 are oriented down and must change sides. Ring opening to **H’** allows for a conformational change moving H10 from bottom to top (box in Scheme 3), prior to rearrangement to **I’**. A subsequent 1,3‐hydride shift leads to **J’** still with H2 on the wrong side. Ring opening to **M’** allows for another conformational change with counterclockwise rotation around C2‐C3 that leads to **N’** with H2 on the top side. Ring closure to **O’** and deprotonation complete the biosynthesis of **12**, involving an unusual “break‐flip‐cyclise” mechanism (**M’** to **O’**).

This mechanistic proposal was further studied through DFT computations (Table S11 and Figure S92), revealing a two‐step process from **C’** to **E’**. Until **G’,** the cascade proceeds through low activation barriers and is exergonic (−13.3 kcal/mol). The transformation from **G’** through the secondary cation **H’** to **I’** is associated with the highest activation barrier (+25.1 kcal/mol), while all subsequent steps toward **12** again show lower barriers.

**SCHEME 4:**
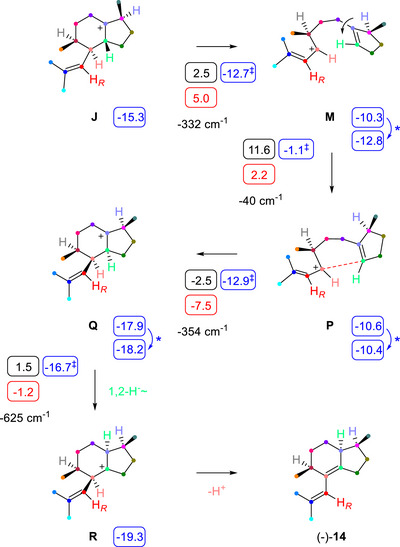
Cyclisation mechanism of BgPgS for the side product **14**. For explanations regarding computational data (small boxes), cf. legend of Scheme 2.

Explaining the biosynthesis of **14** requires the following two concepts: First, the fate of Me12/Me13 is the same as for all side products, and thus **14** arises through bicyclogermacrene (Scheme 2). Downstream reactions lead to **J**, in which H10 is located on the wrong side. Second, a similar “break‐flip‐cyclise” mechanism as for **12** installs the required C10 configuration (Scheme 4): Ring opening in **J** leads to **M**, followed by conformational change to **P** and ring closure to **Q**. A 1,2‐hydride shift yields **R**, the precursor to **14**, on an energetically plausible path (Table S12 and Figure S93).

## 3 Conclusions

This study has addressed the complex mechanism of BgPgS in pacifigorgiane biosynthesis through experiment and theory. Labeling experiments returned unexpected results regarding Me12/Me13 distribution, showing different outcomes for **12** and the side products. This observation has major implications, demonstrating the relevance of detailed atom tracing in terpene biosynthesis research. Additional investigations on the origin of hydrogens (H2, H6, and H10) revealed a difficult stereochemical problem, requiring a migration of H2 and H10 from one hemisphere into the other. Since normal hydride migrations must proceed suprafacially, standard terpene biosynthesis routines cannot explain the formation of **12**, and several biosynthetic alternatives considered during the course of this study were excluded, because they showed reaction barriers even >50 kcal/mol or were not in line with the results of labeling experiments (Schemes S2–S23, Figure S94). Our mechanistic proposal involving 1) a conformational change during a rearrangement and 2) a “break‐flip‐cyclise” sequence as key steps offers creative solutions to these problems beyond the usual cyclisation—hydride shift—deprotonation cascades. It should be emphasised that any mechanistic model in chemistry can, in principle, never be absolutely verified, while falsification only requires one contradictory experiment. The model presented here is in line with all the numerous experiments performed in this study, and is also largely supported by computational chemistry. One problematic step toward **12** is associated with a comparably high activation barrier of 25.1 kcal/mol found for a key transformation that suggests a solution for the change of one hydrogen from bottom to top. This barrier may be mitigated by enzyme catalysis, posing a question that will need to be clarified in future work, for example, through QM/MM modeling. Compound **14** can be explained from different biosynthetic intermediates, **I** (Scheme 2) or **I’** (Scheme 3), but neither of these precursors correctly explains all labeling results, showing the requirement of a unique explanation. Overall, the unexpected complexity of pacifigorgiane biosynthesis impressively demonstrates the need for rigorous approaches to gain conclusive insights into terpene biosynthesis. The proposal for pacifigorgiadienes publicly placed with this article is made to ignite further debate on this complex biosynthetic problem.

## Conflicts of Interest

The authors declare no conflicts of interest.

## Supporting information




**Supporting File 1**: The authors have cited additional references within the Supporting Information [30, 31, 32, 33, 34, 35, 36, 37, 38, 39, 40, 41, 42, 43, 44, 45, 46, 47, 48, 49, 50, 51, 52, 53]

## References

[1] K. U. Wendt and G. E. Schulz , “Isoprenoid Biosynthesis: Manifold Chemistry Catalyzed by Similar Enzymes,” Structure 6 (1998): 127–133, 10.1016/S0969-2126(98)00015-X. 9519404

[2] R. R. Izac , S. E. Poet , W. Fenical , D. van Engen , and J. Clardy , “The Structure of Pacifigorgiol, an Ichthyotoxic Sesquiterpenoid From the Pacific Gorgonian Coral,” Tetrahedron Letters 23 (1982): 3743–3746, 10.1016/S0040-4039(00)87695-9.

[3] R. de Nys , G. M. König , A. D. Wright , and O. Sticher , “Two Metabolites From the Red Alga *Laurencia Flexilis* ,” Phytochemistry 34 (1993): 725–728.

[4] M. Kuniyoshi , M. S. Marma , T. Higa , G. Bernardinelli , and C. W. Jefford , “New Bromoterpenes From the Red Alga Laurencialuzonensis,” Journal of Natural Products 64 (2001): 696–700, 10.1021/np000638o. 11421726

[5] H. Su , Z.‐H. Yuan , J. Li , et al., “Sesquiterpenes From the Marine Red Alga Laurencia saitoi,” Helvetica Chimica Acta 92 (2009): 1291–1297, 10.1002/hlca.200800437.

[6] R. Bos , H. Hendriks , J. Kloosterman , and G. Sipma , “Isolation of the Sesquiterpene Alcohol (‐)‐Pacifigorgiol from *Valeriana officinalis* ,” Phytochemistry 25 (1986): 1234–1235.

[7] J. D. Connolly , L. J. Harrison , and D. S. Rycroft , “The Structure of Tamariscol, a New Pacifigorgiane Sesquiterpenoid Alcohol From the Liverwort,” Tetrahedron Letters 25 (1984): 1401–1402, 10.1016/S0040-4039(01)80169-6.

[8] M. Tori , M. Sono , and Y. Asakawa , “Absolute Configuration and Synthesis of the Liverwort Sesquiterpene Alcohol Tamariscol,” Journal of the Chemical Society, Perkin Transactions 1 (1990): 2849, 10.1039/p19900002849.

[9] C. Paul , W. A. König , and H. Muhle , “Pacifigorgianes and Tamariscene as Constituents of *Frullania tamarisci* and *Valeriana officinalis* ,” Phytochemistry 57 (2001): 307–313, 10.1016/S0031-9422(01)00018-8. 11382249

[10] T. Kitayama , G. Kawabata , and M. Ito , “Concise Synthesis of Valerena‐4,7(11)‐diene, a Highly Active Sedative, From Valerenic Acid,” Bioscience, Biotechnology, and Biochemistry 74 (2010): 1963–1964, 10.1271/bbb.100297. 20834146

[11] Reference [9]mentions the absolute configuration of 1 was assigned through a total synthesis (M. G. Martin, Total Synthesis of Pacifigorgiol, PhD thesis, Cornell University, 1983), but according to the supervisor of this thesis this is not the case (Prof. Jon Clardy, personal communication).

[12] P. D. Scesa , Z. Lin , and E. W. Schmidt , “Ancient Defensive Terpene Biosynthetic Gene Clusters in the Soft Corals,” Nature Chemical Biology 18 (2022): 659–663, 10.1038/s41589-022-01027-1. 35606556 PMC10262820

[13] I. Burkhardt , T. de Rond , P. Y.‐T. Chen , and B. S. Moore , “Ancient Plant‐Like Terpene Biosynthesis in Corals,” Nature Chemical Biology 18 (2022): 664–669, 10.1038/s41589-022-01026-2. 35606558 PMC9179088

[14] Y.‐S. Yeo , S. E. Nybo , A. G. Chittiboyina , et al., “Functional Identification of Valerena‐1,10‐diene Synthase, a Terpene Synthase Catalyzing a Unique Chemical Cascade in the Biosynthesis of Biologically Active Sesquiterpenes in *Valeriana officinalis* ,” Journal of Biological Chemistry 288 (2013): 3163–3173, 10.1074/jbc.M112.415836. 23243312 PMC3561538

[15] C. Conart and H. T. Simonsen , “Tamariscol Biosynthesis in *Frullania tamarisci* ,” Phytochemistry 229 (2025): 114301, 10.1016/j.phytochem.2024.114301. 39424091

[16] S. K. Paknikar , S. H. Kadam , A. L. Ehrlich , and R. B. Bates , “Alternate Biosynthesis of Valerenadiene and Related Sesquiterpenes,” Natural Products Communications 8 (2013): 1195–1196. 24273843

[17] J. W. Cornforth , R. H. Cornforth , G. Popjak , and L. Yengoyan , Studies on the Biosynthesis of Cholesterol 241 (1966): 3970–3987. 4288360

[18] L. Lauterbach , J. Rinkel , and J. S. Dickschat , “Zwei Bakterielle Diterpensynthasen aus Allokutzneria Albata für Bonnadien Sowie für Phomopsen und Allokutzneren,” Angewandte Chemie 130 (2018): 8412–8415, 10.1002/ange.201803800.

[19] P. Rabe , J. Rinkel , B. Nubbemeyer , T. G. Köllner , F. Chen , and J. S. Dickschat , “Terpene Cyclases from Social Amoebae,” Angewandte Chemie 55 (2016): 15646–15649. 27862766 10.1002/anie.201608971

[20] J. Rinkel and J. S. Dickschat , “Addressing the Chemistry of Germacrene A by Isotope Labeling Experiments,” Organic Letters 21 (2019): 2426–2429, 10.1021/acs.orglett.9b00725. 30859837

[21] F. M. Hahn , A. P. Hurlburt , and C. D. Poulter , “Escherichia coli Open Reading Frame 696 Is idi , a Nonessential Gene Encoding Isopentenyl Diphosphate Isomerase,” Journal of Bacteriology 181 (1999): 4499–4504, 10.1128/JB.181.15.4499-4504.1999. 10419945 PMC103578

[22] Y. Asakawa , M. Toyota , and T. Takemoto , “Three *Ent*‐Secoaromadendrane‐Type Sesquiterpene Hemiacetals and a Bicyclogermacrene From *Plagiochila Ovalifolia* and *Plagiochila Yokogurensis* ,” Phytochemistry 19 (1980): 2141–2145, 10.1016/S0031-9422(00)82211-6.

[23] P. Rabe , L. Barra , J. Rinkel , et al., “Konformationsanalyse, Thermische Umlagerung und EI‐MS‐Fragmentierungsmechanismus von (1(10) E ,4 E ,6 S ,7 R )‐Germacradien‐6‐Ol durch^13^ C‐Markierungsexperimente,” Angewandte Chemie 127 (2015): 13649–13653, 10.1002/ange.201507615.

[24] T. A. Klapschinski , P. Rabe , and J. S. Dickschat , “Pristinol, Ein Sesquiterpen‐Alkohol mit Ungewöhnlichem Gerüst aus Streptomyces pristinaespiralis,” Angewandte Chemie 128 (2016): 10296–10299, 10.1002/ange.201605425. 27403888

[25] J. Rinkel , P. Rabe , X. Chen , T. G. Köllner , F. Chen , and J. S. Dickschat , “Mechanisms of the Diterpene Cyclases β‐Pinacene Synthase From Dictyostelium Discoideum and Hydropyrene Synthase From Streptomyces Clavuligerus,” Chemistry—A European Journal 23 (2017): 10501–10505, 10.1002/chem.201702704. 28696553

[26] J. Rinkel , L. Lauterbach , and J. S. Dickschat , “Eine Verzweigte Diterpenkaskade: Der Mechanismus der Spinodien‐Synthase Aus Saccharopolyspora Spinosa,” Angewandte Chemie 131 (2019): 461–465, 10.1002/ange.201812216. 30426646

[27] G. Bian , J. Rinkel , Z. Wang , et al., “Eine Chimäre Pilzliche Diterpensynthase Der Klade II‐D Aus Colletotrichum Gloeosporioides Produziert Dolasta‐1(15),8‐Dien,” Angewandte Chemie 130 (2018): 16113–16117, 10.1002/ange.201809954. 30277637

[28] P. Rabe , J. Rinkel , E. Dolja , et al., “Mechanistische Studien an Zwei Bakteriellen Diterpencyclasen: Spiroviolen‐Synthase und Tsukubadien‐Synthase,” Angewandte Chemie 129 (2017): 2820–2823, 10.1002/ange.201612439.

[29] J. Jiang , X. He , and D. E. Cane , “Biosynthesis of the Earthy Odorant Geosmin by a Bifunctional *Streptomyces Coelicolor* Enzyme,” Nature Chemical Biology 3 (2007): 711–715, 10.1038/nchembio.2007.29. 17873868 PMC3013058

[30] T. Siemon , Z. Wang , G. Bian , et al., “Semisynthesis of Plant‐Derived Englerin A Enabled by Microbe Engineering of Guaia‐6, 10 (14)‐Diene as Building Block,” Journal of the American Chemical Society 142 (2020): 2760–2765, 10.1021/jacs.9b12940. 31999448

[31] M. M. Bradford , “A Rapid and Sensitive Method for the Quantitation of Microgram Quantities of Protein Utilizing the Principle of Protein‐dye Binding,” Analytical Biochemistry 72 (1976): 248–254, 10.1016/0003-2697(76)90527-3. 942051

[32] G. R. Fulmer , A. J. M. Miller , N. H. Sherden , et al., “NMR Chemical Shifts of Trace Impurities: Common Laboratory Solvents, Organics, and Gases in Deuterated Solvents Relevant to the Organometallic Chemist,” Organometallics 29 (2010): 2176–2179, 10.1021/om100106e.

[33] D. N. Tran and N. Cramer , “Biomimetic Synthesis of (+)‐Ledene, (+)‐Viridiflorol, (−)‐Palustrol, (+)‐Spathulenol, and Psiguadial A, C, and D via the Platform Terpene (+)‐Bicyclogermacrene,” Chemistry—A European Journal 20 (2014): 10654–10660, 10.1002/chem.201403082. 24867775

[34] J. Abramson , J. Adler , J. Dunger , et al., “Accurate Structure Prediction of Biomolecular Interactions With AlphaFold 3,” Nature 630 (2024): 493–500. 38718835 10.1038/s41586-024-07487-w PMC11168924

[35] O. Trott and A. J. Olson , “AutoDock Vina: Improving the Speed and Accuracy of Docking With a New Scoring Function, Efficient Optimization, and Multithreading,” Journal of Computational Chemistry 31 (2010): 455–461, 10.1002/jcc.21334. 19499576 PMC3041641

[36] R. D. Gietz and R. H. Schiestl , “Large‐Scale High‐Efficiency Yeast Transformation Using the LiAc/SS Carrier DNA/PEG Method,” Nature Protocols 2 (2007): 38–41, 10.1038/nprot.2007.15. 17401336

[37] T. Lou , A. Li , H. Xu , et al., “Structural Insights Into Three Sesquiterpene Synthases for the Biosynthesis of Tricyclic Sesquiterpenes and Chemical Space Expansion by Structure‐Based Mutagenesis,” Journal of the American Chemical Society 145 (2023): 8474–8485, 10.1021/jacs.3c00278. 37018048

[38] H. Xu , H. Li , B. Goldfuss , G. Schnakenburg , and J. S. Dickschat , “Skeletal Rearrangements in the Enzyme‐Catalysed Biosynthesis of Coral‐Type Diterpenes From *Chitinophaga pinensis* ,” Angewandte Chemie International Edition 63 (2024): e202413860. 39195349 10.1002/anie.202413860

[39] S. Grimme , S. Ehrlich , and L. Goerigk , “Effect of the Damping Function in Dispersion Corrected Density Functional Theory,” Journal of Computational Chemistry 32 (2011): 1456–1465, 10.1002/jcc.21759. 21370243

[40] Gaussian 16, Revision C.01 , M. J. Frisch , G. W. Trucks , H. B. Schlegel , et al. (Wallingford CT: Gaussian, Inc., 2019).

[41] S. Grimme , “Supramolecular Binding Thermodynamics by Dispersion‐Corrected Density Functional Theory,” Chemistry—A European Journal 18 (2012): 9955–9964, 10.1002/chem.201200497. 22782805

[42] GoodVibes v3.0.1 , G. Luchini , J. V. Alegre‐Requena , Y. Guan , I. Funes‐Ardoiz , and R. S. Paton , 2019.

[43] N. Mardirossian and M. Head‐Gordon , “ω B97M‐V: A Combinatorially Optimized, Range‐Separated Hybrid, Meta‐GGA Density Functional With VV10 Nonlocal Correlation,” The Journal of Chemical Physics 144 (2016): 214110, 10.1063/1.4952647. 27276948

[44] F. Weigend and R. Ahlrichs , “Balanced Basis Sets of Split Valence, Triple Zeta Valence and Quadruple Zeta Valence Quality for H to Rn: Design and Assessment of Accuracy,” Physical Chemistry Chemical Physics 7 (2005): 3297, 10.1039/b508541a. 16240044

[45] F. Neese , “Software Update: The ORCA Program System—Version 6.0,” Computational Molecular Science 2025, 15, e70019.

[46] F. Neese , F. Wennmohs , A. Hansen , and U. Becker , “Efficient, Approximate and Parallel Hartree–Fock and Hybrid DFT Calculations. A ‘Chain‐of‐spheres’ Algorithm for the Hartree–Fock Exchange,” Chemical Physics 356 (2009): 98–109, 10.1016/j.chemphys.2008.10.036.

[47] M. Bursch , J.‐M. Mewes , A. Hansen , and S. Grimme , “Best‐Practice DFT Protocols for Basic Molecular Computational Chemistry**,” Angewandte Chemie International Edition 61 (2022): e202205735, 10.1002/anie.202205735. 36103607 PMC9826355

[48] S. Zev , P. K. Gupta , E. Pahima , and D. T. Major , “A Benchmark Study of Quantum Mechanics and Quantum Mechanics‐Molecular Mechanics Methods for Carbocation Chemistry,” Journal of Chemical Theory and Computation 18 (2022): 167–178, 10.1021/acs.jctc.1c00746. 34905380

[49] P. Pracht , F. Bohle , and S. Grimme , “Automated Exploration of the Low‐Energy Chemical Space With Fast Quantum Chemical Methods,” Physical Chemistry Chemical Physics 22 (2020): 7169–7192, 10.1039/C9CP06869D. 32073075

[50] S. Grimme , “Exploration of Chemical Compound, Conformer, and Reaction Space With Meta‐Dynamics Simulations Based on Tight‐Binding Quantum Chemical Calculations,” Journal of Chemical Theory and Computation 15 (2019): 2847–2862, 10.1021/acs.jctc.9b00143. 30943025

[51] P. Pracht and S. Grimme , “Calculation of Absolute Molecular Entropies and Heat Capacities Made Simple,” Chemical Science 12 (2021): 6551–6568, 10.1039/D1SC00621E. 34040731 PMC8139639

[52] P. Pracht , C. A. Bauer , and S. Grimme , “Automated and Efficient Quantum Chemical Determination and Energetic Ranking of Molecular Protonation Sites,” Journal of Computational Chemistry 38 (2017): 2618–2631, 10.1002/jcc.24922. 28861911

[53] S. Spicher , C. Plett , P. Pracht , A. Hansen , and S. Grimme , “Automated Molecular Cluster Growing for Explicit Solvation by Efficient Force Field and Tight Binding Methods,” Journal of Chemical Theory and Computation 18 (2022): 3174–3189, 10.1021/acs.jctc.2c00239. 35482317

