# Supplementary material for: Strong Implications From Small Deviations in Labeling Patterns: The Mechanism of Burkholderia gladioli Pacifigorgiadiene Synthase
Source: Chemistry. 2026 Feb 10;32(16):e00029. doi: 10.1002/chem.202600029 (PMC13109686; doi:10.1002/chem.202600029)
Supplement: Supplementary file 1 — Supporting File 1: The authors have cited additional references within the Supporting Information [30, 31, 32, 33, 34, 35, 36, 37, 38, 39, 40, 41, 42, 43, 44, 45, 46, 47, 48, 49, 50, 51, 52, 53] [file CHEM-32-e00029-s001.pdf]

Table of Contents

Biosynthetic hypothesis for valerena-1,10-diene	2
Phylogenetic tree	3
Culture conditions, gene expression, protein purification, incubation experiments	4
Chemicals and chromatography, spectroscopic methods	6
EI mass spectra of **12** – **15**, **9** and **6**	8
Compound isolation and spectroscopic data of **12** – **15**, **9** and **6**	9
Structure elucidation and NMR data of **12**	10
Structure elucidation and NMR data of **13**	15
Structure elucidation and NMR data of **14**	20
Structure elucidation and NMR data of **15**	25
Molecular simulation and site-directed mutagenesis	30
Structure elucidation and NMR data of **9**	34
Structure elucidation and NMR data of **6**	39
List of isotopic labelling experiments	44
The absolute configuration of **12** – **15**, **9** and **6**	45
^{13}C Labelling of individual carbons	56
The reprotonation step in the formation of BgPgS products	62
The reprotonation of isolepidozene in the formation of **12**	63
The deprotonation from bicyclogermacrene to **B** and isolepidozene to **B'**	64
The site of incorporation of H2 in **12**	66
The site of incorporation of H10 in **12**	67
The loss of H6 in the biosynthesis of **12**	68
The site of incorporation of H2 in **13**	69
The site of incorporation of H10 in **13**	70
The loss of H6 in the biosynthesis of **13**	71
The site of incorporation of H2 in **15**	72
The loss of H6 in the biosynthesis of **15**	73
The site of incorporation of H2 in **6**	74
The site of incorporation of H6 in **6**	75
The site of incorporation of H10 in **6**	76
The site of incorporation of H2 in **9**	77
The site of incorporation of H6 in **9**	78
The loss of H10 in the biosynthesis of **9**	79
The loss of H2 in the biosynthesis of **14**	80
Computational methods	81
Results of DFT calculations for Scheme 2B of main text	82
Results of DFT calculations for Scheme 3 of main text	84
Results of DFT calculations for Scheme 4 of main text	86
Discussion of mechanistic alternatives for the biosynthesis of **12** by BgPgS	88
Cartesian coordinates of computed structures (Scheme 2B of main text)	111
Cartesian coordinates of computed structures (Scheme 3 of main text)	129
Cartesian coordinates of computed structures (Scheme 4 of main text)	152
Cartesian coordinates of computed structures (mechanistic alternatives)	160

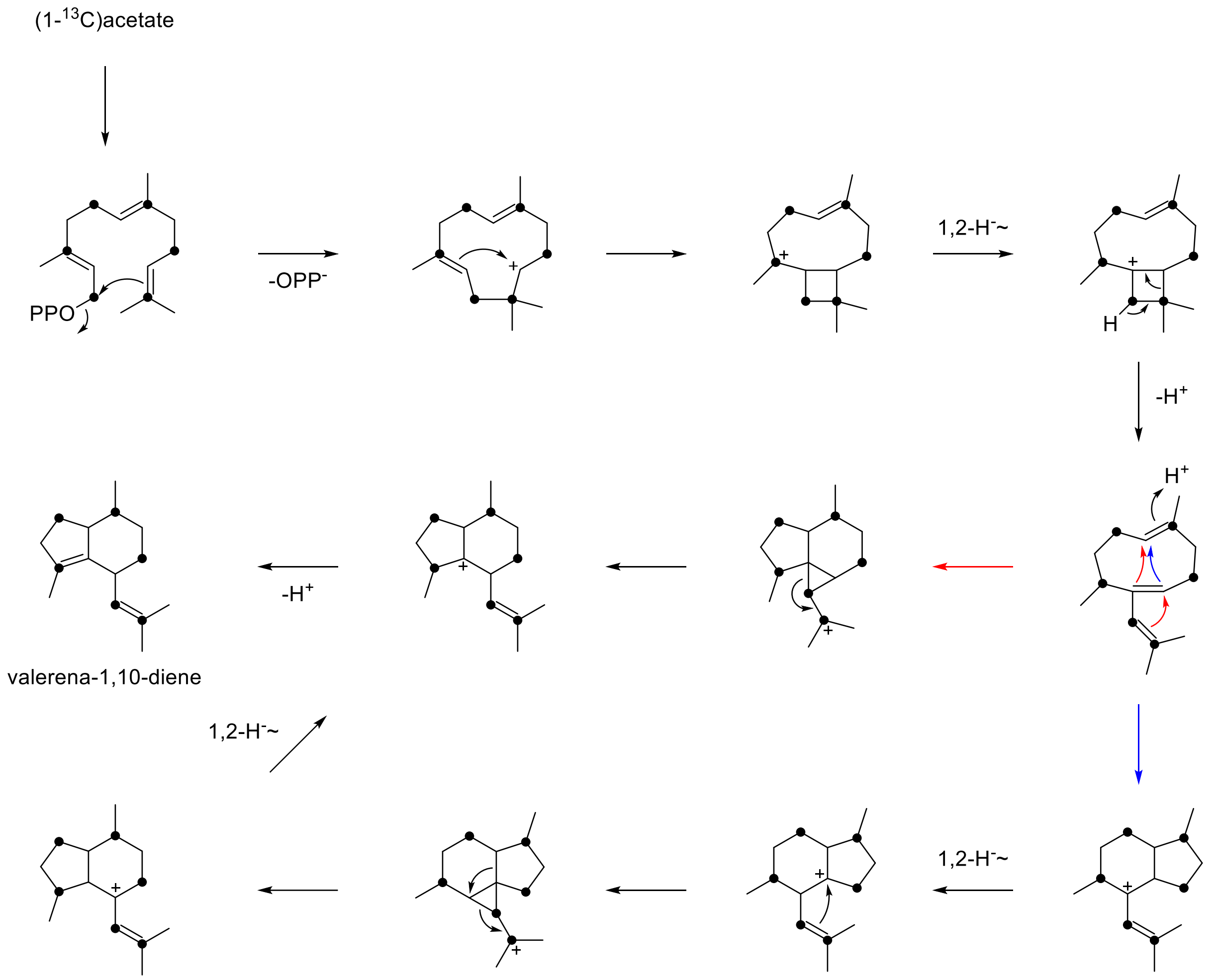

Scheme S1. Feeding experiment and biosynthetic hypothesis for valerena-1,10-diene.[7]

Construction of phylogenetic tree

Continuous BLAST searches during the past ~15 years using the amino acid sequences of various characterised terpene synthases as probes resulted in the discovery of 5667 bacterial terpene synthase homologs, verified by individual inspection for the presence of the highly conserved motifs in type I terpene synthases. All these sequences are included in the phylogenetic tree of Figure S1. The tree was constructed using the tree builder function of Geneious (alignment type: global alignment with free end gaps, cost matrix: Blosum45, genetic distance model: Jukes-Cantor, tree build method: neighbor-joining, gap open panelty: 8, gap extension penalty: 2).

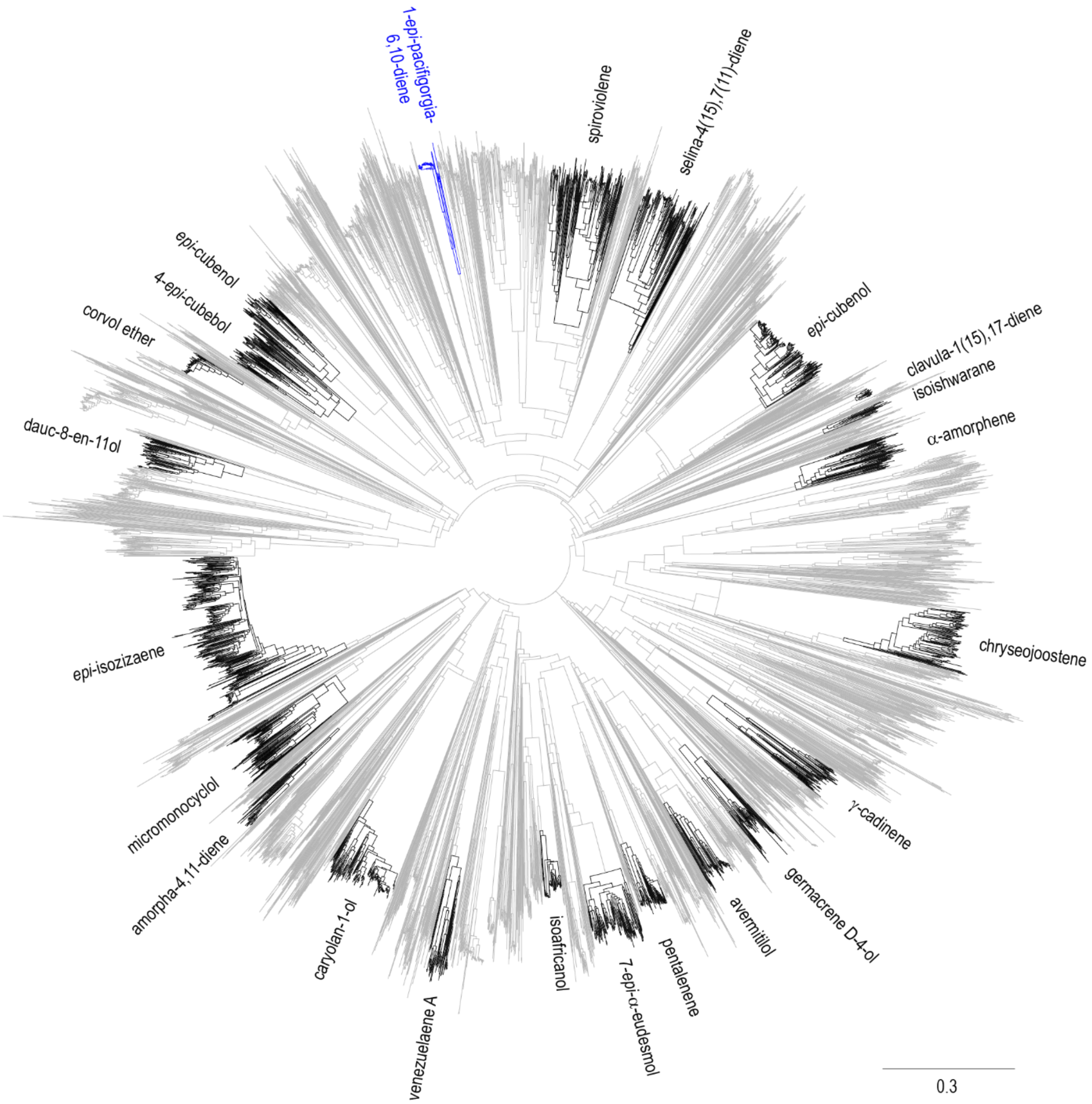

Figure S1. Phylogenetic tree constructed from the amino acid sequences of 5667 bacterial terpene synthase homologs. Clades with at least one characterised member are highlighted in black, the clade containing BgPgS characterised in this study is highlighted in blue.

Strains, culture conditions and media

Saccharomyces cerevisiae JCR27[30] was used as the host for heterologous expression. Transformants of the *S. cerevisiae* strain was grown in shaking cultures in YPD (1% glucose, 1% galactose) medium at 30 °C and 220 rpm. *Escherichia coli* DH5 transformants carrying the recombinant plasmids were grown in LB medium containing ampicillin (100 µg mL^{-1}). *E. coli* BL21 (DE3) was used for protein expression.

Construction of recombinant plasmids

The coding sequence of *BgPgS* was synthesized by Beijing Tsingke Biotech Co., Ltd., and codon optimized for *S. cerevisiae.* The yeast transformation fragment was amplified using primers BgPgS-pESC-F/R (Table S1), followed by cloning into pESC-URA using the ClonExpress® II One Step Cloning Kit C112 (Vazyme, Nanjing, China) to yield plasmid pESC-BgPgS. Using the same strategy, the fragment amplified with primers BgPgS-C6T-F/R (Table S1) was inserted into the pMAL-c6T vector for expression in *E. coli*, yielding pMAL-BgPgS.

Table S1. Oligonucleotide primers for the construction of plasmids used in this study.[a]

Primers	Sequence 5' → 3'
BgPgS-pESC-F	GAAAAAACTATAGCGGCCGCATGCCTGACGATTTCAGATC
BgPgS-pESC-R	AACTAATTACATGAACTAGTCTACCACCAACCTGCTAAAG
BgPgS-C6T-F	AGATGCTGATGGGCGGCCGCATGCCTGACGATTTCAGATC
BgPgS-C6T-R	CGTTTTATTTGAAGCTTTCACTACCACCAACCTGCTAAAG
BgPgS-F177A-F	TCCATTG**gct**CCAGCAAGATCTATGCCAGATGC
BgPgS-F177A-R	TTGCTGG**agc**CAATGGAAATCTAGCTCTTGCTAAGT
BgPgS-V191A-F	GGCTGGTAAAGAA**gct**ATGTGGTTACAATCATTCGACGAC
BgPgS-V191A-R	T**agc**TTCTTTACCAGCCAATGTCAGACCATGA
BgPgS-L194A-F	GTTATGTGG**gct**CAATCATTCGACGACGTTCATT
BgPgS-L194A-R	GATTG**agc**CCACATAACTTCTTTACCAGCCAA
BgPgS-Q195A-F	GTGGTTA**gct**TCATTCGACGACGTTCATTCTG
BgPgS-Q195A-R	CGAATGA**agc**TAACCACATAACTTCTTTACCAGCCA
BgPgS-Y268A-F	ATTGAC**gct**TTCGATGGTCTATTATGGGAAGC
BgPgS-Y268A-R	CCATCGAA**agc**GTCAATATGTGCTCTAACCCATCTATC
BgPgS-W294A-F	CGCTATG**gct**TTGAAGCAATCTGGTGTTTATCCA
BgPgS-W294A-R	GCTTCAA**agc**CATAGCGATGTAATCGTCTATTCTTG
BgPgS-Q297A-F	GTTGAAG**gct**TCTGGTGTTTATCCATGTATCGCT
BgPgS-Q297A-R	CACCAGA**agc**CTTCAACCACATAGCGATGTAATCG
BgPgS-S298A-F	GAAGCAA**gct**GGTGTTTATCCATGTATCGCTTTC
BgPgS-S298A-R	AAACACC**agc**TTGCTTCAACCACATAGCGATG
BgPgS-G299A-F	GTTGAAGCAATCT**gct**GTTTATCCATGTATCGCTTTCACAG
BgPgS-G299A-R	C**agc**AGATTGCTTCAACCACATAGCGATGTAA
BgPgS-V300A-F	TCTGGT**gct**TATCCATGTATCGCTTTCACAGATT
BgPgS-V300A-R	CATGGATA**agc**ACCAGATTGCTTCAACCACATAGC
BgPgS-C303A-F	GTTTATCCA**gct**ATCGCTTTCACAGATTTGGCA
BgPgS-C303A-R	GCGAT**agc**TGGATAAACACCAGATTGCTTCAA
BgPgS-I336A-F	CAATC**gct**GGTTGGGATAATGATCTGACTTCA
BgPgS-I336A-R	ATCCCAACC**agc**GATTGCAGCGGTCATTTCTCTT
BgPgS-Y422A-F	CAGAGGT**gct**CTAGACTATTCTGCACTTTCTCCGAG
BgPgS-Y422A-R	AGTCTAG**agc**ACCTCTGATCCATTGAGCTAAACC

[a] Modified triplet codons in mutational primers are shown in bold lowercase letters.

Gene expression and protein purification

E. coli BL21 (DE3) cells harboring the expression plasmid pMAL-BgPgS were used to inoculate a starter culture in LB medium (10 mL) supplied with ampicillin (50 µg/mL), which was grown with shaking at 37 °C overnight. The starter culture was used to inoculate the expression culture (1/100 v/v) in LB medium (1 L) with ampicillin and the cells were grown with shaking at 37 °C until OD_{600} = 0.4 – 0.6 was reached. The culture was cooled to 18 °C, before IPTG (0.4 mM final concentration) was added to induce expression. The culture was shaken at the same temperature overnight and then centrifuged (3500 x g, 40 min, 4 °C).

For the preparation scale of bTS305, the medium was discarded and the cell pellet was resuspended in binding buffer (10 mL L^{-1} culture; 20 mM Na_2HPO_4, 500 mM NaCl, 20 mM imidazole, 1 mM $MgCl_2$, pH 7.4, 4 °C). The cells were lysed by ultrasonication (10 x 1 min) under ice cooling. The cell debris was spun down (14600 x g, 10 min, 4 °C), the protein solution was filtered with disposable syringe filter (Macherey-Nagel GmbH & Co. KG), and loaded onto a Ni^{2+}-NTA affinity chromatography column (10 mL column volume; Ni-NTA superflow, Qiagen, Venlo, Netherlands). The column was washed with two column volumes of binding buffer (10 mL L^{-1} culture) to elute non-binding proteins, followed by desorption of the target protein from the stationary phase with two column volumes of elution buffer (10 mL L^{-1} culture; 20 mM Na_2HPO_4, 500 mM NaCl, 500 mM imidazole, 1 mM $MgCl_2$, pH 7.4, 4 °C) with fractionation. The fractions were analysed by SDS-PAGE and fractions containing pure protein were pooled (Figure S2) and used for incubation experiments. The protein concentration was determined through Bradford assay[31] and adjusted to 5.0 mg/mL.

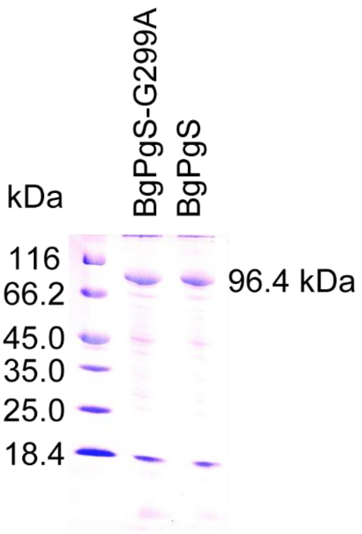

Figure S2. SDS-PAGE analysis of purified recombinant BgPgS and BgPgS-G299A with MBP tag (96.4 kDa).

Incubation experiments with recombinant BgPgS

Analytical scale incubations were performed with the substrates GPP, FPP, GGPP and GFPP (0.5 mg each) dissolved in substrate buffer (0.1 mL, 25 mM NH_4HCO_3). After dilution with incubation buffer (0.8 mL, 50 mM Tris, 10 mM $MgCl_2$, 20% glycerol, pH = 7.4), enzyme elution fraction (0.1 mL, containing 5.0 mg mL^{-1} enzyme) was added. The reaction mixture was incubated at 30 °C with shaking for 15 h, followed by extraction with n-hexane (200 µL). The organic layer was dried with $MgSO_4$ and analysed by GC/MS (Figures S3 and S4).

Chemicals and chromatography

Chemicals were purchased from Sigma Aldrich Chemie GmbH (Steinheim, Germany), Carbolution Chemicals GmbH (St. Ingbert, Germany), or Carl Roth (Karlsruhe, Germany) and used without purification. Solvents for column chromatography were purchased in p.a. grade and purified by distillation. Thin-layer chromatography (TLC) was performed with 0.2 mm precoated plastic sheets Polygram Sil G/UV254 purchased from Machery-Nagel (Düren, Germany). Column chromatography was performed using silica gel 60 purchased from Merck (Darmstadt, Germany).

GC/MS and GC/MS-QTOF analyses

GC/MS analyses were performed on a 5977A GC/MSD system (Agilent, Santa Clara, CA, USA) with a 7890B GC and a 5977A mass selective detector. The GC was equipped with a HP5-MS fused silica capillary column (30 m, 0.25 mm i. d., 0.50 μm film) or with a chiral Cyclosil-B column (30 m, 0.25 mm i. d., 0.25 μm film; used for the experiment shown in Figure S83). Specific GC settings were 1) inlet pressure: 77.1 kPa, He at 23.3 mL min^{-1}, 2) injection volume: 1 μL, 3) temperature program (HP5-MS): 5 min at 50 °C increasing at 10 °C min^{-1} to 320 °C, followed by a hold at this temperature for 5 min, and temperature program (Cyclosil-B): 5 min at 80 °C increasing at 4 °C min^{-1} to 150 °C, then increasing at 20 °C min^{-1} to 220 °C, followed by a hold at this temperature for 5 min, 4) 60 s valve time, and 5) carrier gas: He at 1.2 mL min^{-1}. MS settings were 1) source: 230 °C, 2) transfer line: 250 °C, 3) quadrupole: 150 °C and 4) electron energy: 70 eV. Retention indices (*I*) were determined from retention times in comparison to the retention times of *n*-alkanes (C_7-C_{40}). GC/MS-QTOF analyses were performed on a 7890B GC equipped with a HP5-MS fused silica capillary column (30 m, 0.25 mm i. d., 0.50 µm film) connected to a 7200 accurate-mass QTOF detector (Agilent). GC parameters were 1) inlet pressure: 83.2 kPa, He at 24.6 mL min^{-1}, 2) injection volume: 1 µL, 3) split ratio: 50:1, 60 s valve time, 4) temperature program: 5 min at 50 °C increasing at 5 °C min^{-1} to 320 °C, 5) carrier gas: He at 1 mL min^{-1}. MS parameters were 1) transfer line: 250 °C, 2) electron energy 70 eV.

NMR spectroscopy

NMR spectra were recorded on a Bruker (Billerica, MA, USA) Avance I (300 MHz), Avance I (400 MHz), Avance I (500 MHz), Avance III HD Prodigy (500 MHz) or an Avance III HD Cryo (700 MHz) NMR spectrometer. Spectra were measured in C_6D_6 and referenced against solvent signals (^{1}H-NMR, residual proton signal: δ = 7.16; ^{13}C-NMR: δ = 128.06).[32]

IR spectroscopy

IR spectra were recorded on a Bruker α infrared spectrometer with a diamond ATR probehead. Peak intensities are given as s (strong), m (medium), w (weak) and br (broad).

Optical rotations

Optical rotations were recorded on a Modular Compact Polarimeter MCP 100 (Anton Paar, Graz, Austria). The temperature setting was 25 °C; the wavelength of the light used was 589 nm (sodium D line); the path-length was 10 cm; the compound concentrations *c* are given in g 100 mL^{-1}.

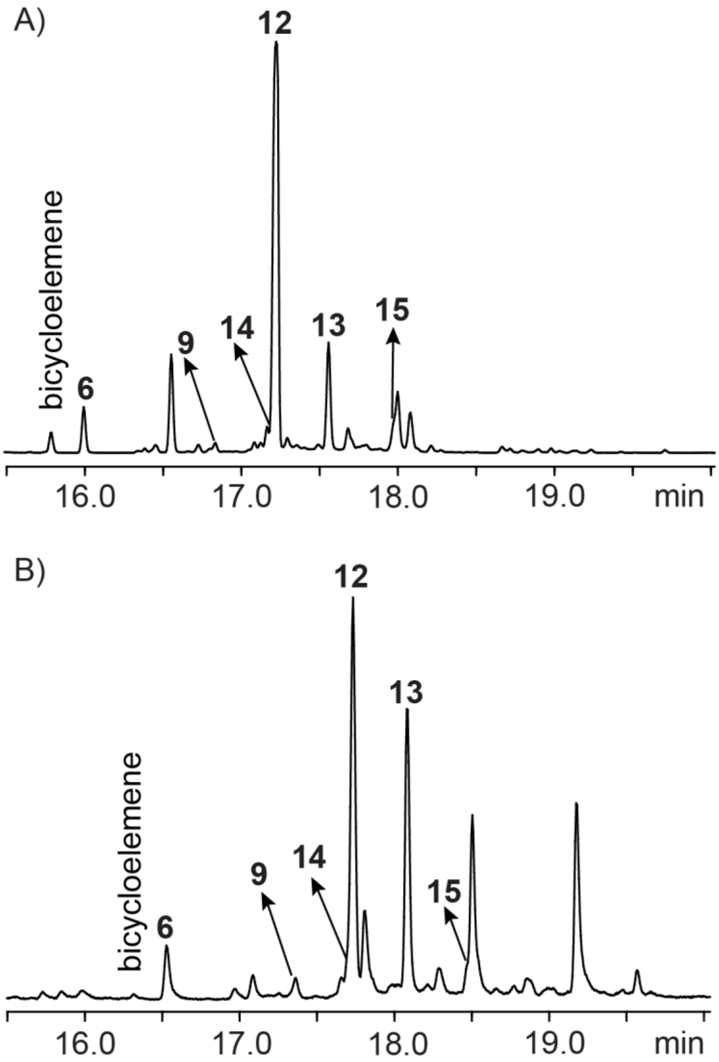

Figure S3. Total ion chromatograms of extracts from enzyme incubations of FPP with A) BgPgS and B) BgPgS-G299A. Bicycloelemene is the Cope rearrangement product of the proposed neutral intermediate bicycloelemene. Isolepidozene or its Cope rearrangement product was not detected, possibly because it is further transformed with higher efficiency into the main product **12**.

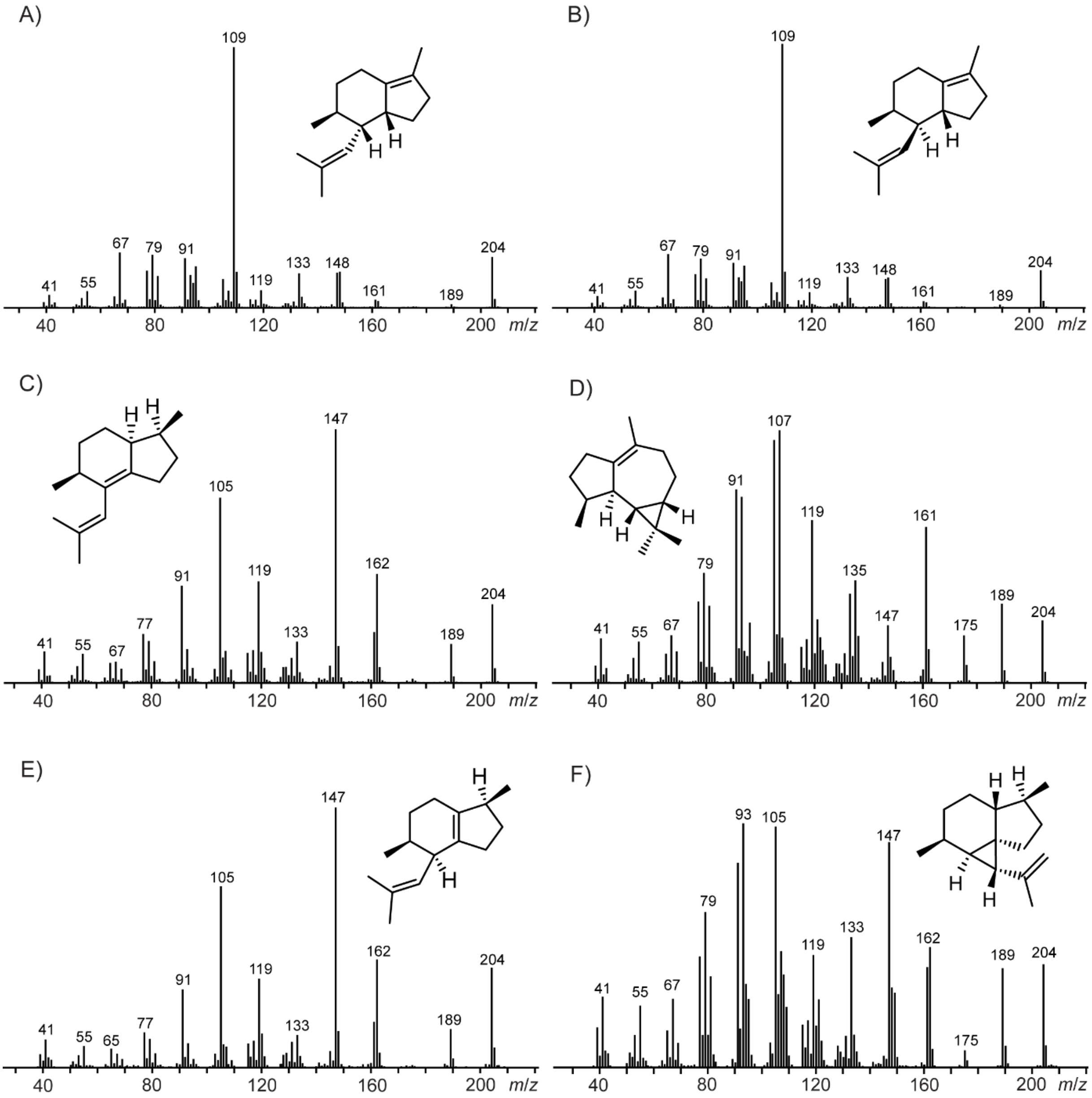

Figure S4. EI mass spectra of A) 1-*epi*-pacifigorgia-6,10-diene (**12**), B) 1,2-*diepi*-pacifigorgia-6,10-diene (**13**), C) 6-*epi*-pacifigorgia-1,10-diene (**14**), D) ledene (**15**), E) pacifigorgia-1(6),10-diene (**9**) and F) tamariscene (**6**).

Preparative scale incubation of FPP with recombinant BgPgS and compound isolation
For preparative scale incubations, FPP (200 mg, 523 μmol) in NH_4HCO_3 (25 mM, 10 mL) and an enzyme preparation of BgPgS or BgPgS-G299A (20 mL; from 4 L expression culture, 5.0 mg mL^{-1}) were added to incubation buffer (70 mL). The reaction mixture was stirred overnight at 30 °C and then extracted with Et_2O (3 x 20 mL). The combined extracts were dried with $MgSO_4$ and concentrated under reduced pressure.
1-*epi*-Pacifigorgia-6,10-diene (**12**, 7.6 mg, 37.3 μmol, 7%) was directly obtained through column chromatography on silica gel with pentane from the crude extract. All the other fractions were combined and separated through column chromatography on silica gel coated with $AgNO_3$ and elution with pentane to yield 1,2-*diepi*-pacifigorgia-6,10-diene (**13**, 1.2 mg, 5.9 μmol, 1%), 6-*epi*-pacifigorgia-1,10-diene (**14**, 0.5 mg, 2.5 μmol, 0.5%) and ledene (**15**, 0.8 mg, 3.9 μmol, 0.8%). Pacifigorgia-1(6),10-diene (**9**, 0.3 mg, 1.5 μmol, 0.3%) and tamariscene (**6**, 0.3 mg, 37.3 μmol, 0.3%) were obtained through column chromatography on silica gel ($AgNO_3$) with pentane from the crude extract of a BgPgS-G299A enzyme reaction.

(–)-1-*epi*-Pacifigorgia-6,10-diene (12). TLC (silica, pentane): R_f = 0.85. GC (HP-5MS): *I* = 1452. IR (diamond ATR): ṽ = 2962 (s), 2923 (s), 2880 (s), 2858 (s), 2840 (m), 1668 (w), 1450 (m), 1375 (m), 1260 (m), 1094 (m), 1065 (m), 1019 (m), 860 (w), 804 (w) cm^{-1}. HR-MS (EI): calc. $[C_{15}H_{24}]^+$ *m*/*z* = 204.1873; found: *m*/*z* = 204.1869. Optical rotation: $[\alpha]_D^{25}$ = –12.9 (*c* 0.14, CH_2Cl_2). NMR data are given in Table S2.

(–)-1,2-*diepi*-Pacifigorgia-6,10-diene (13). TLC (silica, pentane): R_f = 0.87. GC (HP-5MS): *I* = 1480. IR (diamond ATR): ṽ = 2958 (s), 2924 (s), 2835 (s), 1731 (m), 1458 (m), 1377 (w), 1272 (m), 1122 (w), 1174 (m) cm^{-1}. HR-MS (EI): calc. $[C_{15}H_{24}]^+$ *m*/*z* = 204.1873; found: *m*/*z* = 204.1877. Optical rotation: $[\alpha]_D^{25}$ = –18.0 (*c* 0.05, CH_2Cl_2). NMR data are given in Table S3.

(–)-6-*epi*-Pacifigorgia-1,10-diene (14). TLC (silica, pentane): R_f = 0.75. GC (HP-5MS): *I* = 1450. IR (diamond ATR): ṽ = 2959 (s), 2922 (s), 2851 (s), 1658 (m), 1632 (m), 1467 (w), 1410 (w), 1258 (s), 1084 (s), 1012 (m), 793 (s) cm^{-1}. HR-MS (EI): calc. $[C_{15}H_{24}]^+$ *m*/*z* = 204.1873; found: *m*/*z* = 204.1870. Optical rotation: $[\alpha]_D^{25}$ = –40.0 (*c* 0.03, CH_2Cl_2). NMR data are given in Table S4.

(–)-Ledene (15). TLC (silica, pentane): R_f = 0.70. GC (HP-5MS): *I* = 1513. Optical rotation: $[\alpha]_D^{25}$ = –30.0 (*c* 0.06, CH_2Cl_2), literature data for the enantiomer: $[\alpha]_D^{20}$ = +45.0 (*c* 0.20, EtOH).[33] NMR data are given in Table S5.

(–)-Pacifigorgia-1(6),10-diene (9). TLC ($AgNO_3$-coated silica, pentane): R_f = 0.50. GC (HP-5MS): *I* = 1424. Optical rotation: $[\alpha]_D^{25}$ = –30.0 (*c* 0.03, CH_2Cl_2). NMR data are given in Table S7.

(–)-Tamariscene (6). TLC ($AgNO_3$-coated silica, pentane): R_f = 0.35. GC (HP-5MS): *I* = 1363. Optical rotation: $[\alpha]_D^{25}$ = –33.0 (*c* 0.03, CH_2Cl_2). NMR data are given in Table S8.

Table S2. NMR data of (–)-1-*epi*-pacifigorgia-6,10-diene (**12**) in C_6D_6 recorded at 298 K.

C[a]	type	^{13}C[b]	^{1}H[b]
1	CH	127.15	5.41 (br d, *J* = 9.8)
2	CH	44.22	2.48 (m)
3	CH	34.90	1.86 (m)
4	CH_2	27.30	1.78 (dddd, *J* = 13.3, 13.3, 4.9, 4.9)<br>1.33 (dddd, *J* = 13.3, 5.3, 2.6, 2.2)
5	CH_2	21.27	2.32 (m)<br>2.03 (m)
6	C_q	134.63	–
7	C_q	128.85	–
8	CH_2	37.77	2.29 (m)
9	CH_2	25.14	1.85 (m)<br>1.67 (m)
10	CH	44.38	2.95 (br s)
11	C_q	131.22	–
12	CH_3	18.08	1.56 (br s)
13	CH_3	26.28	1.68 (br s)
14	CH_3	13.56	1.63 (br s)
15	CH_3	18.47	1.10 (d, *J* = 7.3)

[a] Carbon numbering as shown in Figure S5 indicates the origin of each carbon from FPP by same number. [b] Chemical shifts δ in ppm. Multiplicity: s = singlet, d = doublet, m = multiplet, br = broad. Coupling constants *J* are given in Hertz.

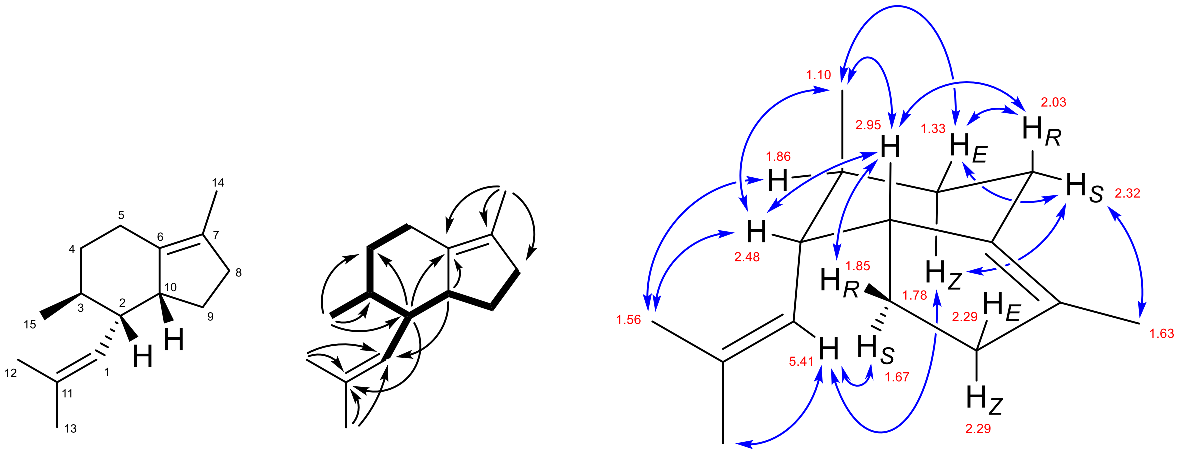

12

Figure S5. Structure elucidation of (–)-1-*epi*-pacifigorgia-6,10-diene (**12**). Bold: $^{1}H,^{1}H$-COSY, single headed arrows: key HMBC, and blue double headed arrows: NOESY correlations. H_R, H_S, H_E and H_Z indicate the results from stereoselective labelling experiments (Figures S55 and S61).

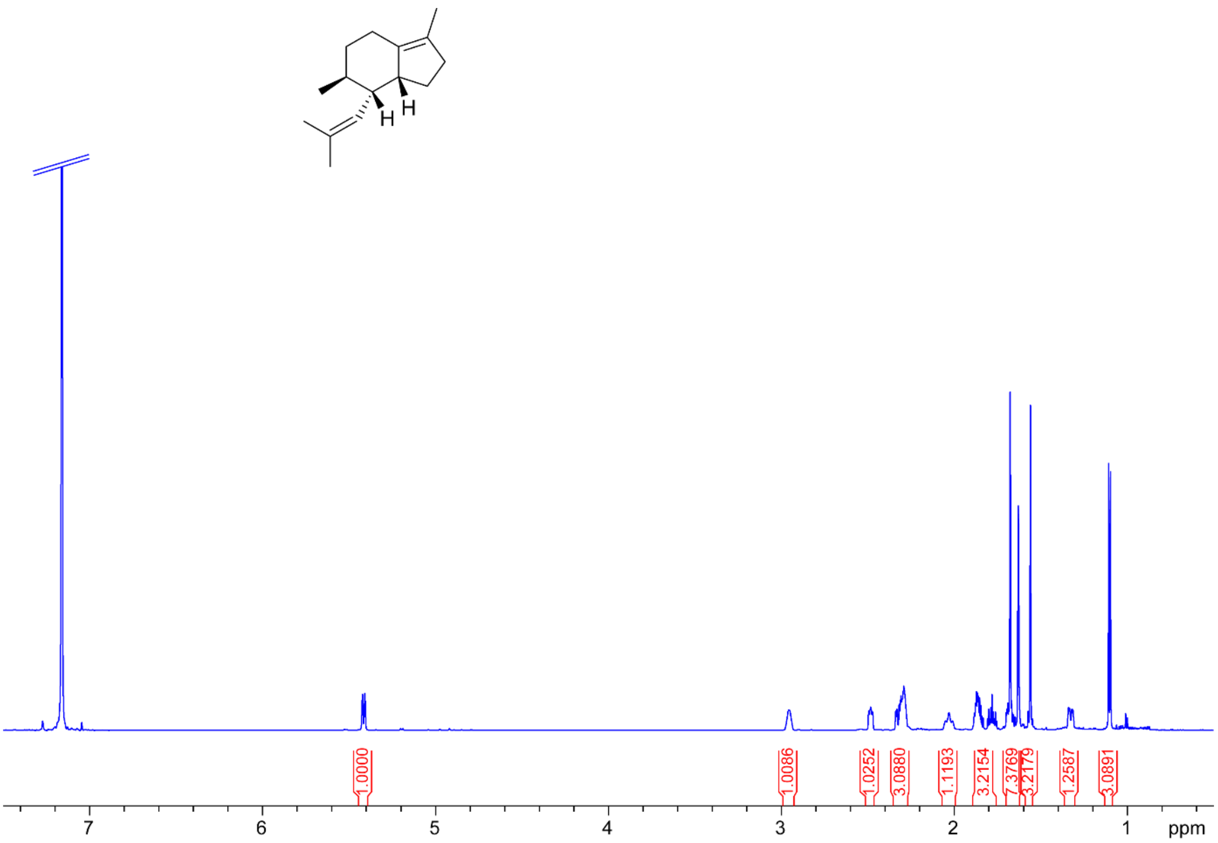

Figure S6. ^{1}H-NMR spectrum of **12** (700 MHz, C_6D_6).

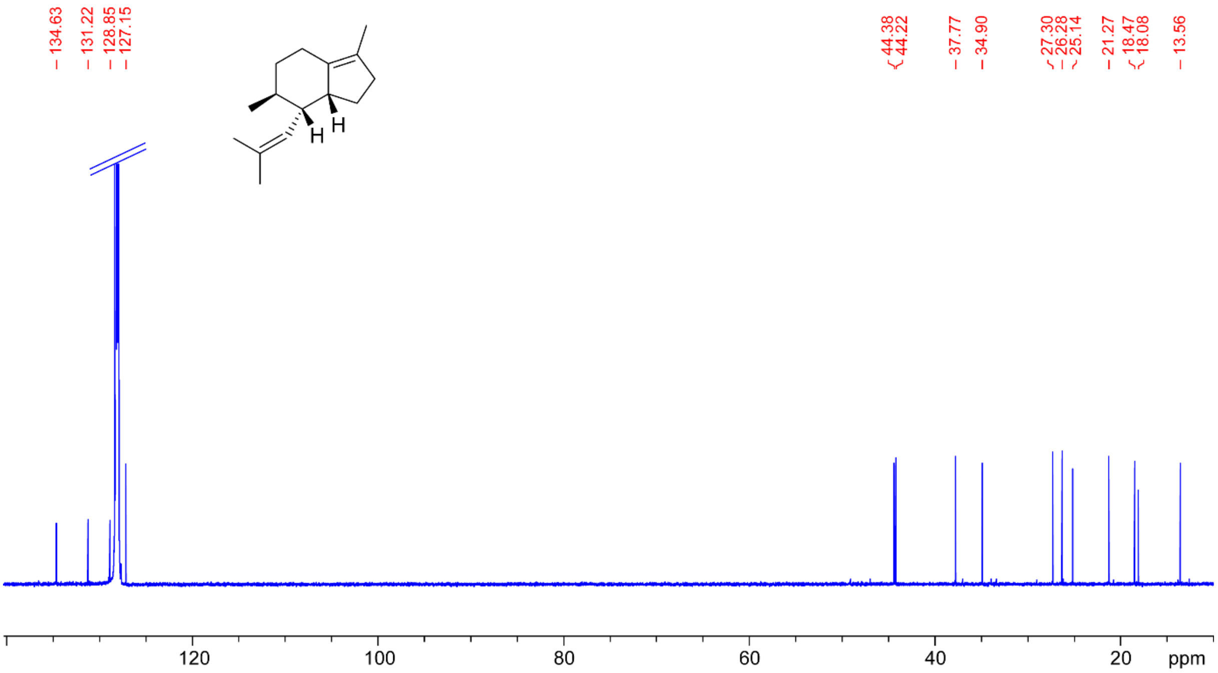

Figure S7. ^{13}C-NMR spectrum of **12** (176 MHz, C_6D_6).

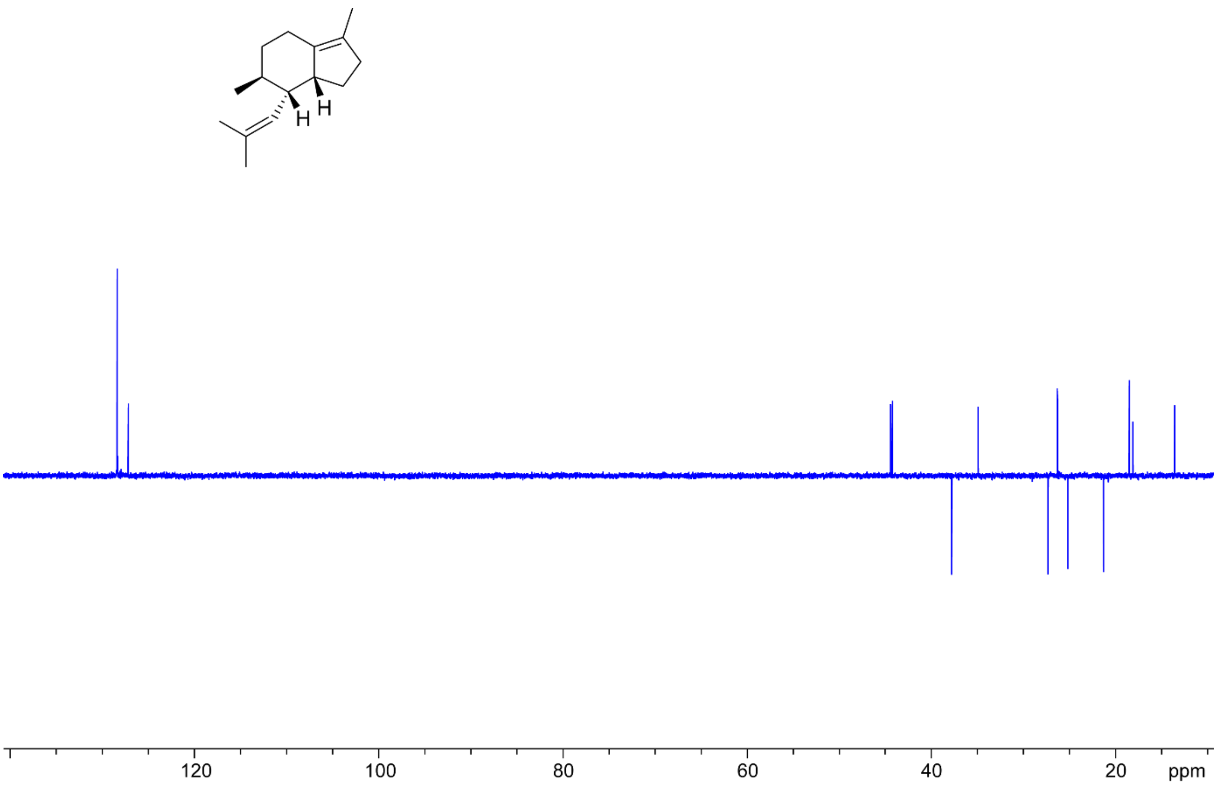

Figure S8. ^{13}C-DEPT135 spectrum of **12** (176 MHz, C_6D_6).

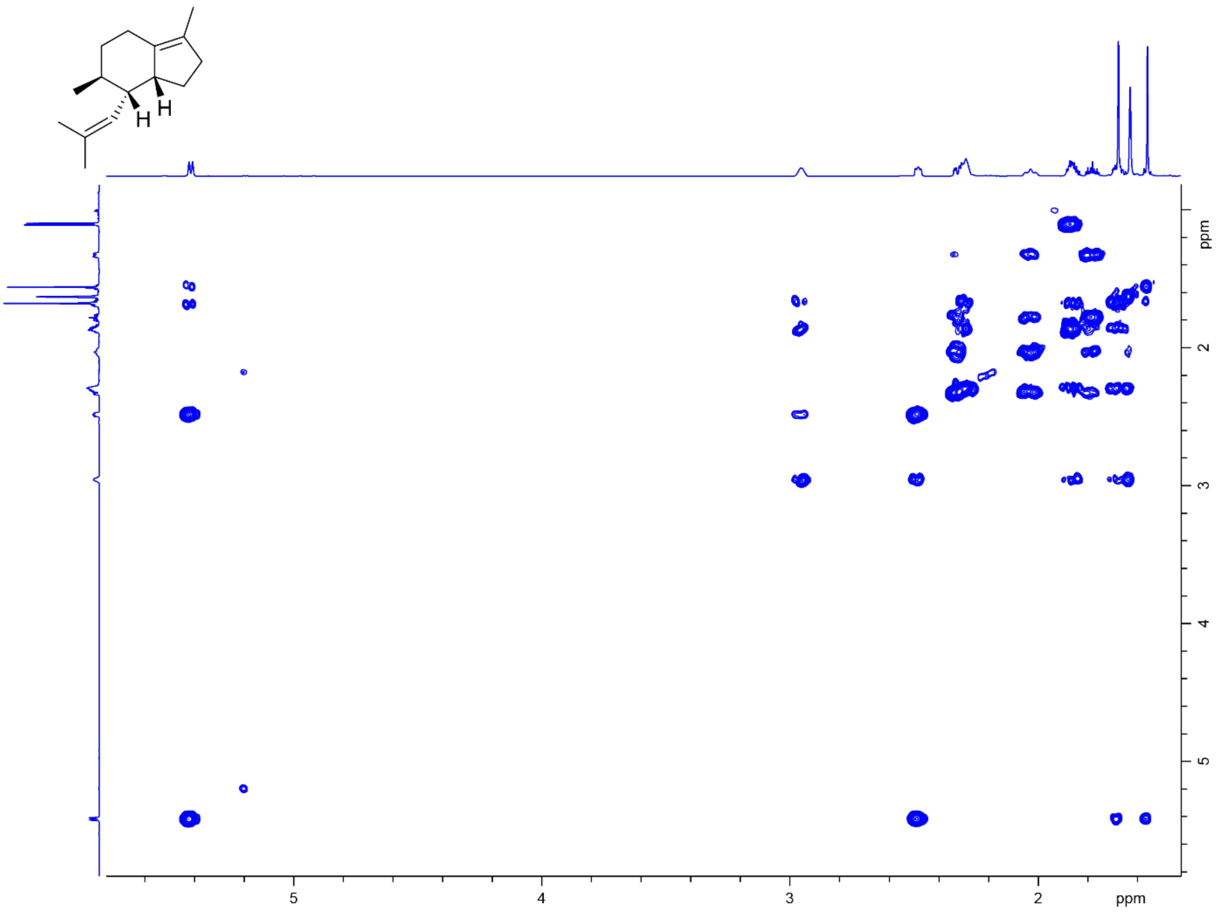

Figure S9. ^{1}H,^{1}H-COSY spectrum (C_6D_6) of **12**.

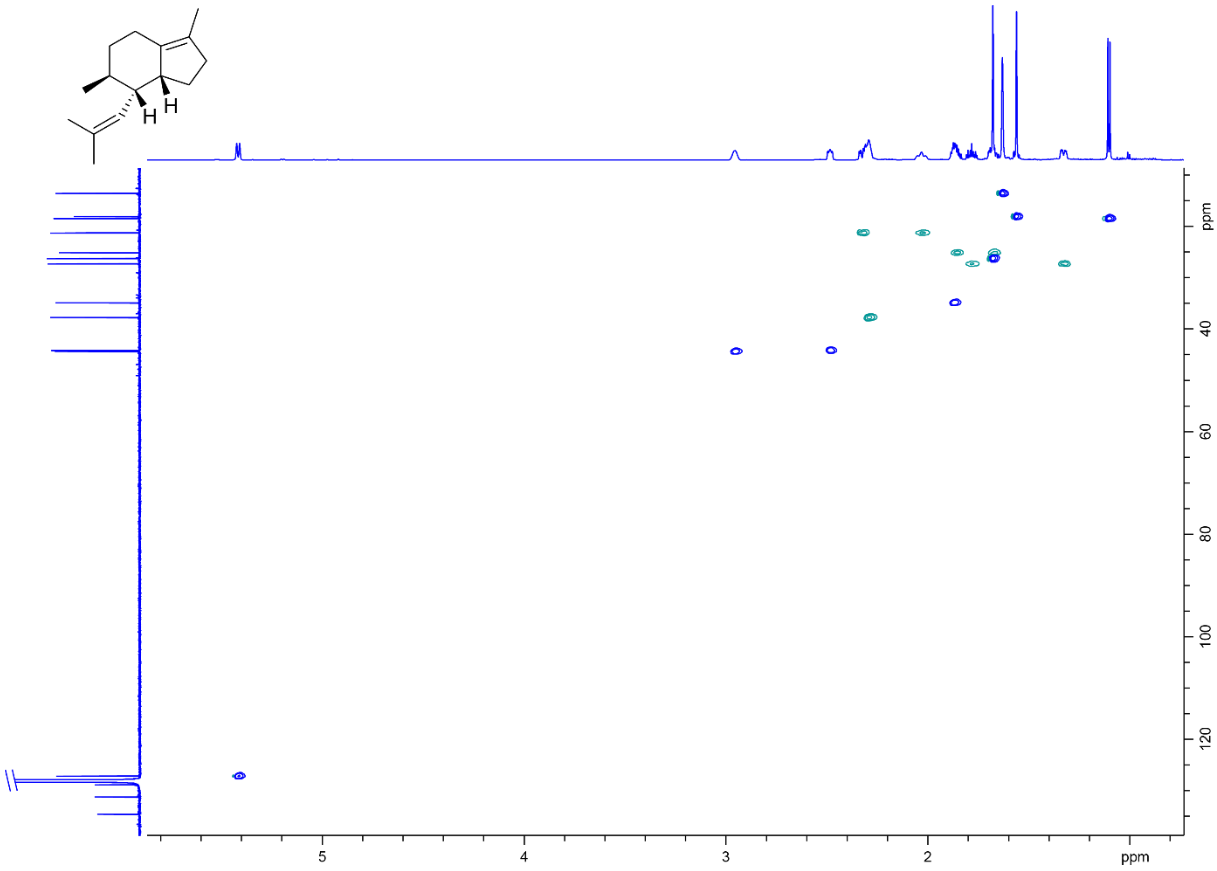

Figure S10. HSQC spectrum (C_6D_6) of **12**.

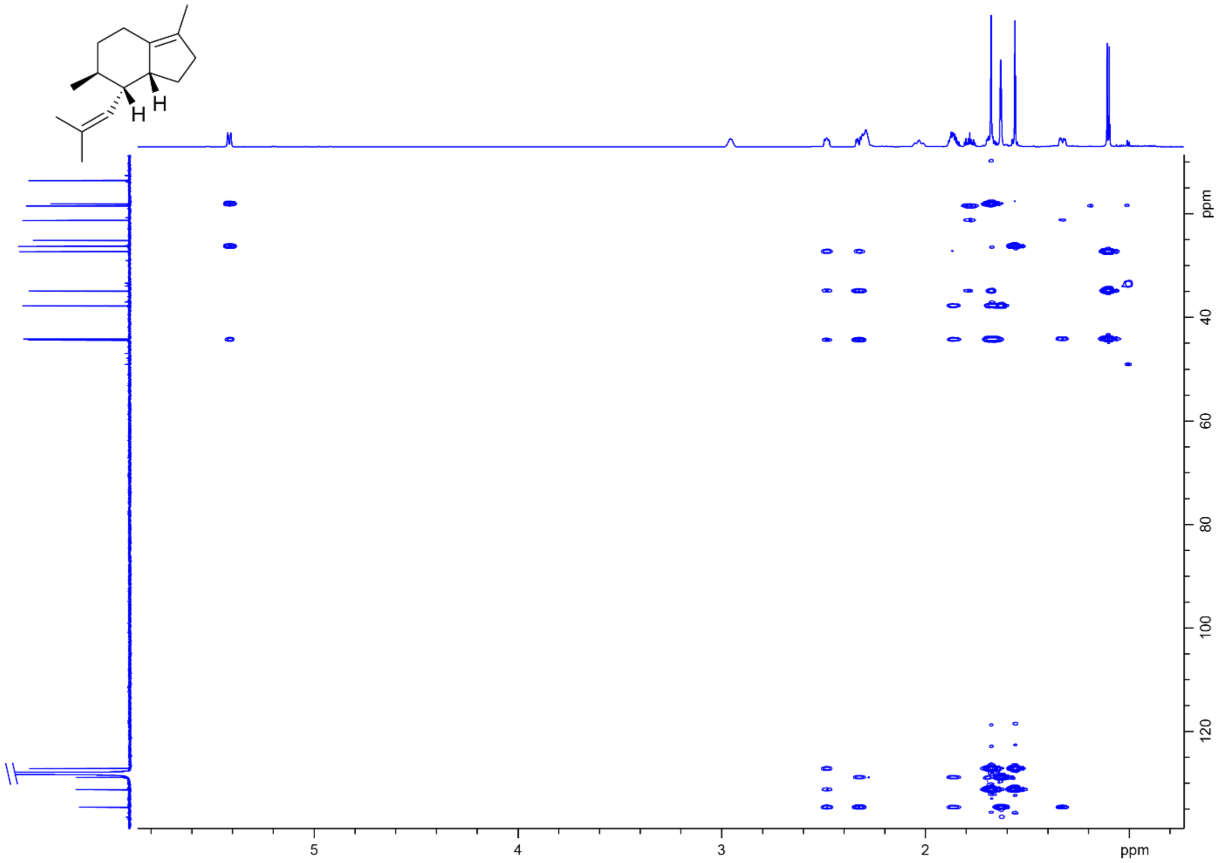

Figure S11. HMBC spectrum (C_6D_6) of **12**.

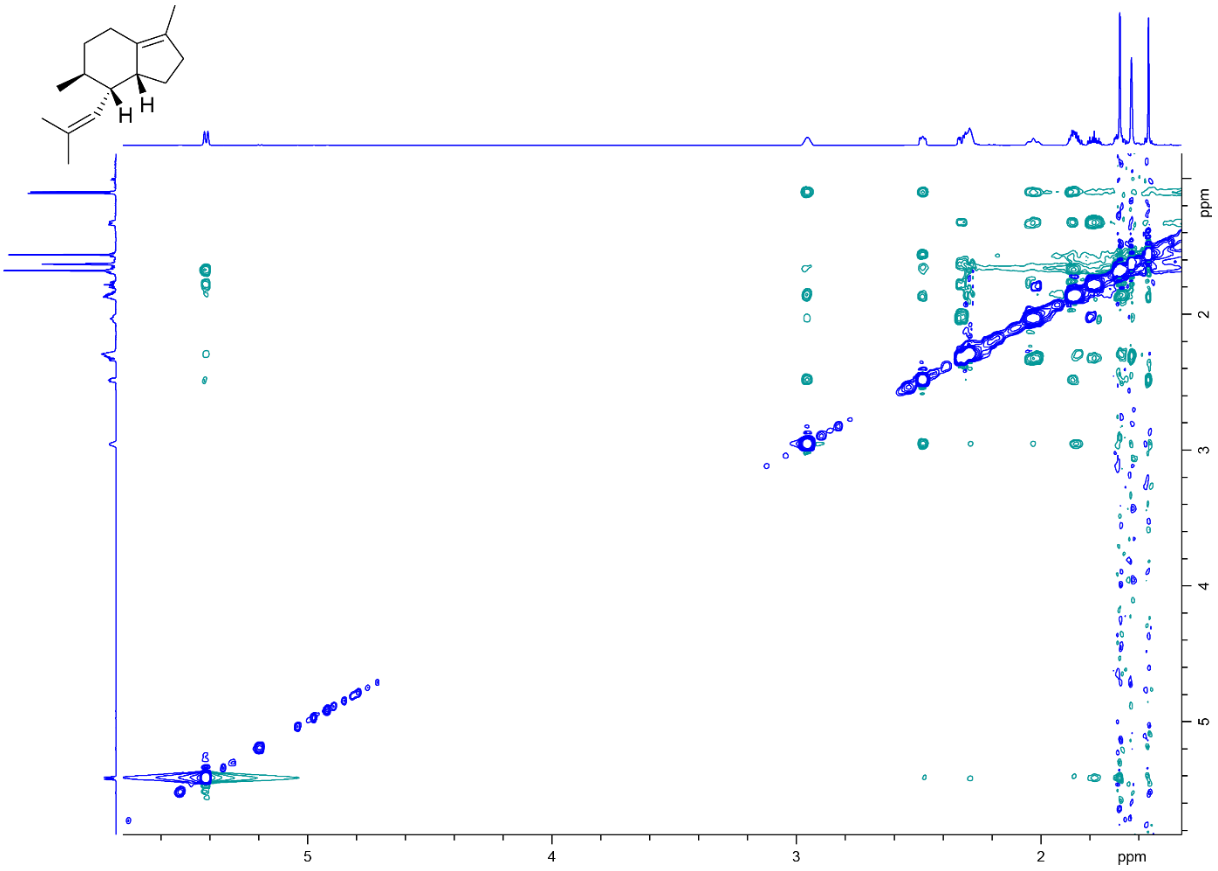

Figure S12. NOESY spectrum (C_6D_6) of **12**.

Table S3. NMR data of (–)-1,2-*diepi*-pacifigorgia-6,10-diene (**13**) in C_6D_6 recorded at 298 K.

C[a]	type	^{13}C[b]	^{1}H[b]
1	CH	128.97	5.19 (br d, *J* = 9.4)
2	CH	49.12	2.17 (ddd, *J* = 11.1, 9.4, 4.1)
3	CH	33.93	1.93 (m)
4	CH_2	33.42	1.60 (m)<br>1.55 (m)
5	CH_2	20.78	2.30 (ddd, *J* = 14.6, 4.8, 2.3)<br>1.99 (m)
6	C_q	136.58	–
7	C_q	128.31	–
8	CH_2	37.02	2.26 (m)<br>2.21 (m)
9	CH_2	29.02	2.04 (m)<br>1.36 (m)
10	CH	47.01	2.54 (br s)
11	C_q	131.19	–
12	CH_3	26.15	1.69 (br s)
13	CH_3	18.27	1.57 (br s)
14	CH_3	13.79	1.62 (br s)
15	CH_3	12.60	1.00 (d, *J* = 7.2)

[a] Carbon numbering as shown in Figure S13 indicates the origin of each carbon from FPP by same number. [b] Chemical shifts δ in ppm. Multiplicity: s = singlet, d = doublet, m = multiplet, br = broad. Coupling constants *J* are given in Hertz.

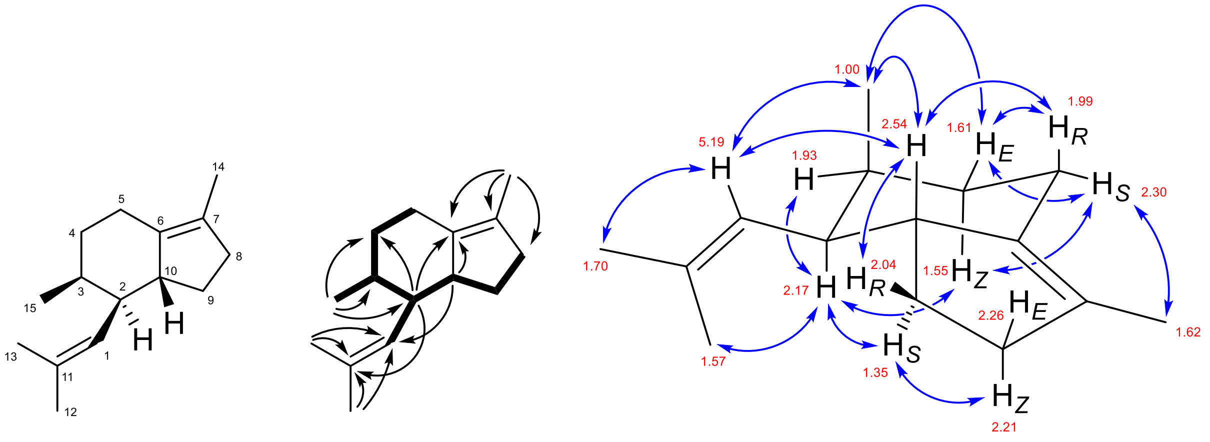

13

Figure S13. Structure elucidation of (–)-1,2-*diepi*-pacifigorgia-6,10-diene (**13**). Bold: ^{1}H,^{1}H-COSY, single headed arrows: key HMBC, and blue double headed arrows: NOESY correlations. H_R, H_S, H_E and H_Z indicate the results from stereoselective labelling experiments (Figures S56 and S62).

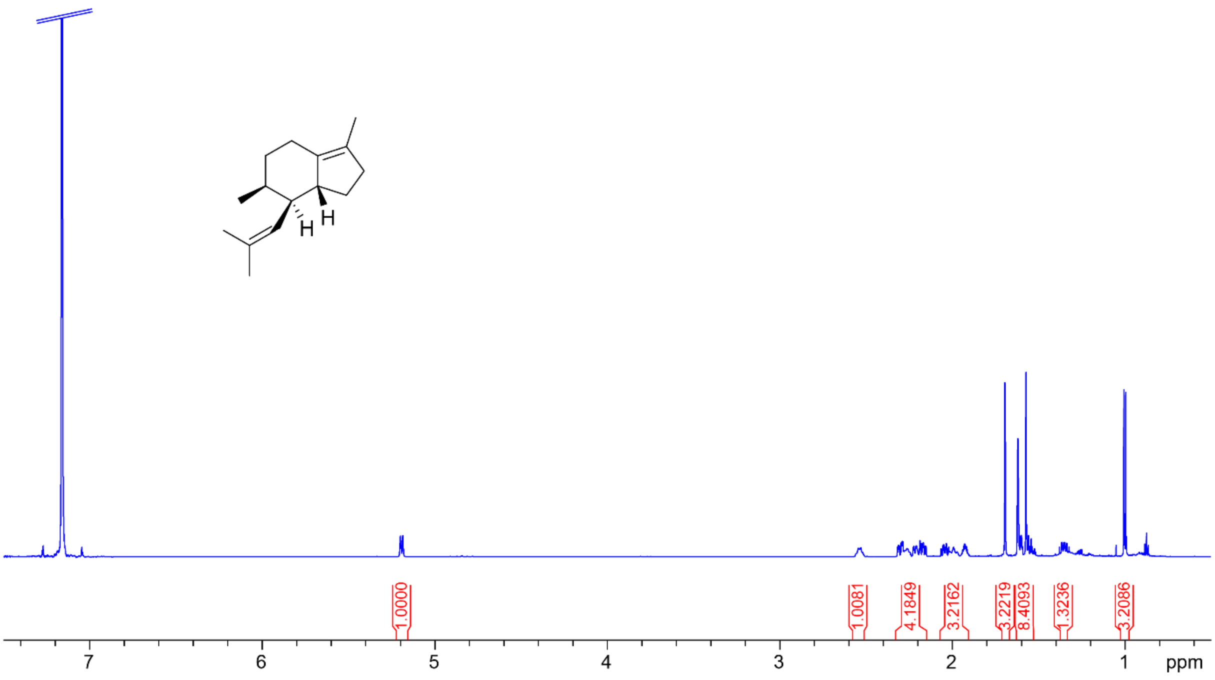

Figure S14. ^{1}H-NMR spectrum of **13** (700 MHz, C_6D_6).

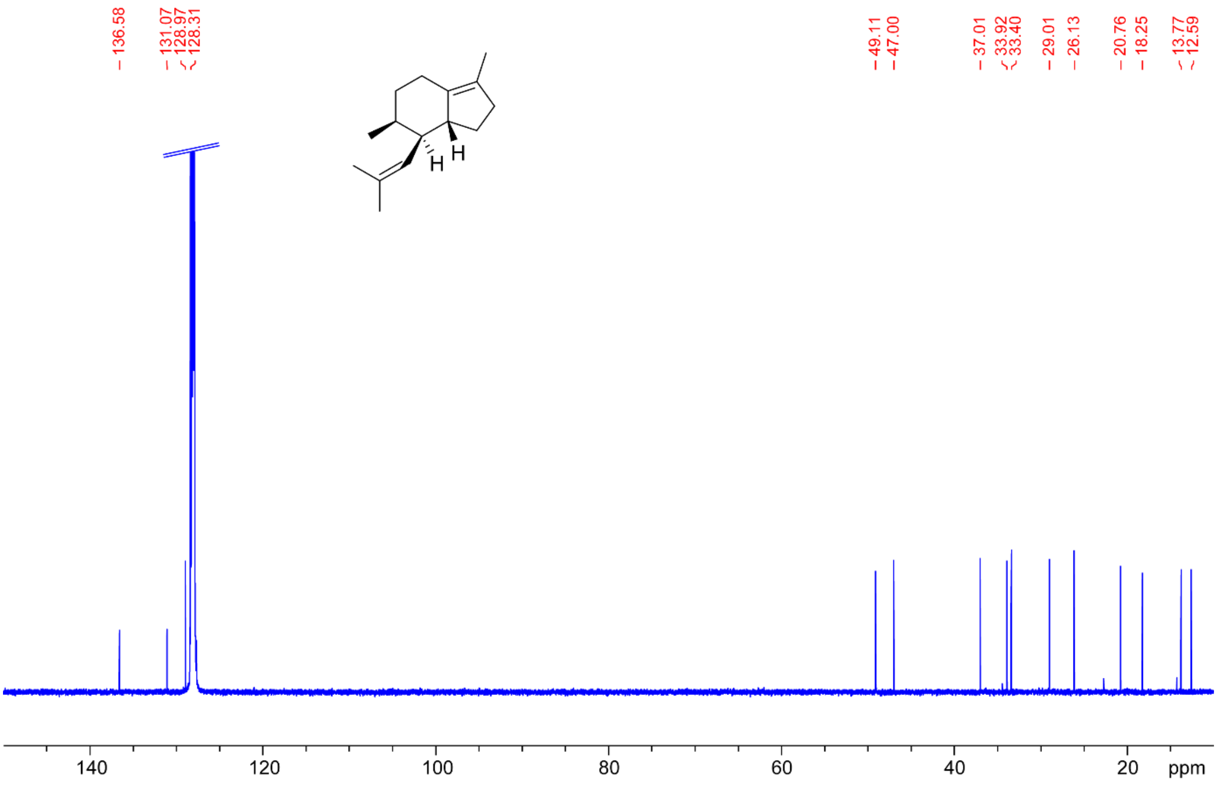

Figure S15. ^{13}C-NMR spectrum of **13** (176 MHz, C_6D_6).

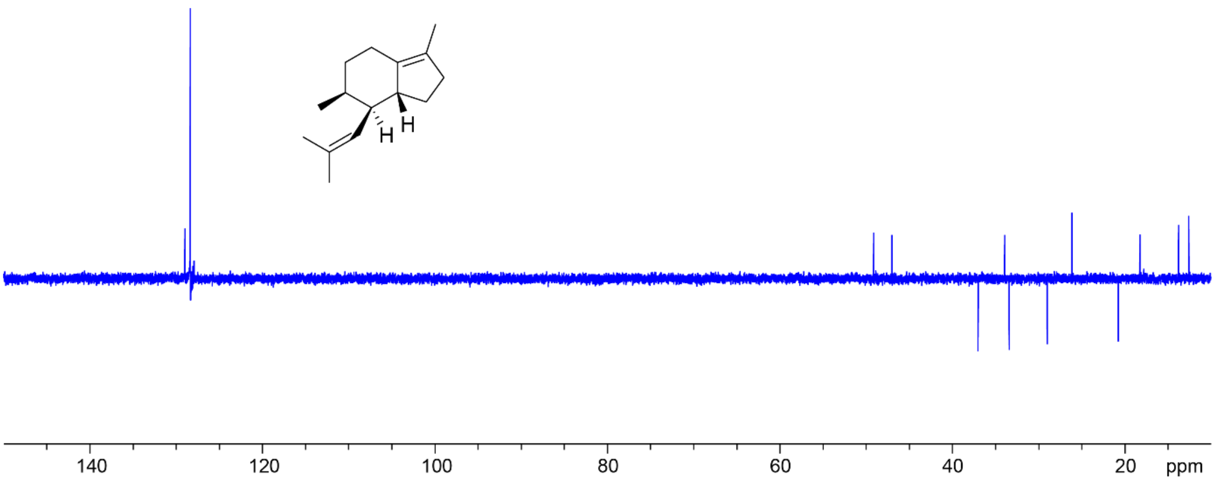

Figure S16. ^{13}C-DEPT135 spectrum of **13** (176 MHz, C_6D_6).

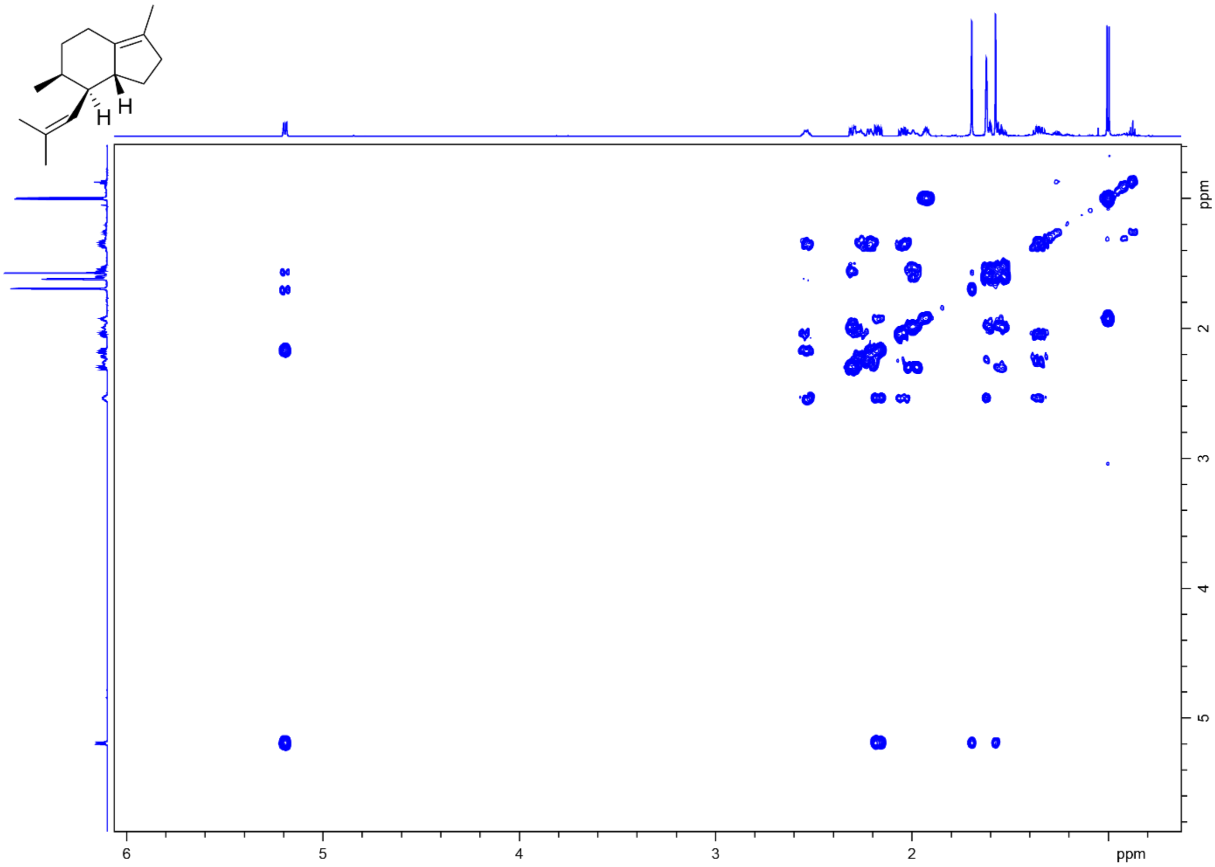

Figure S17. ^{1}H,^{1}H-COSY spectrum (C_6D_6) of **13**.

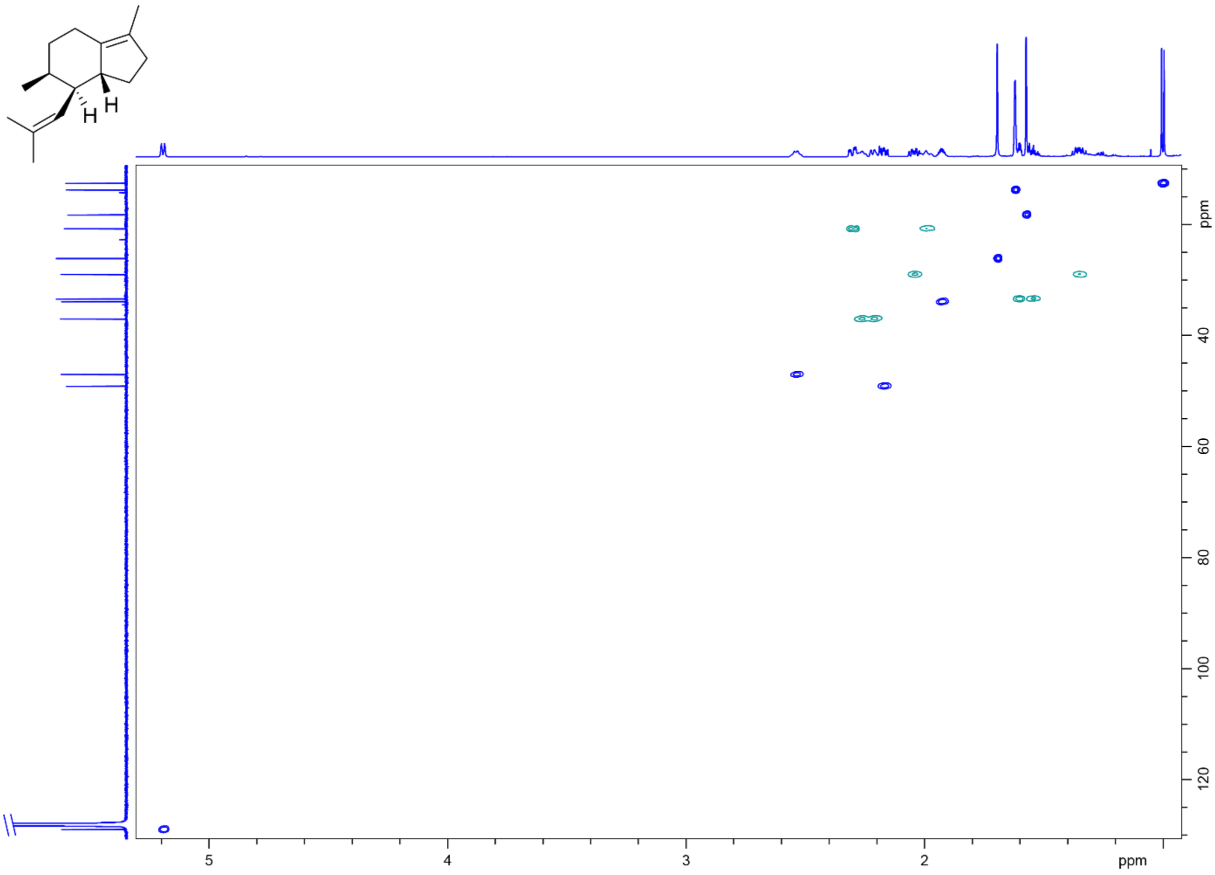

Figure S18. HSQC spectrum (C_6D_6) of **13**.

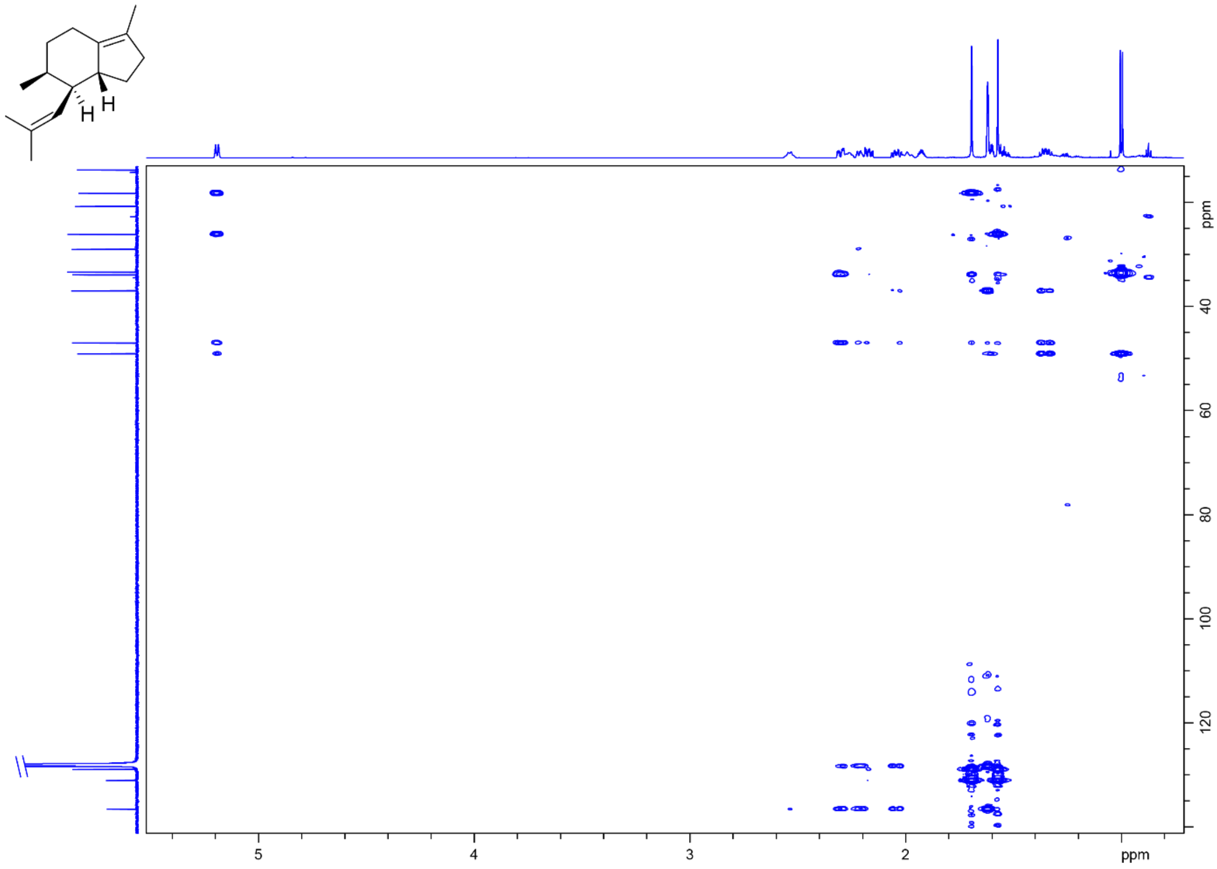

Figure S19. HMBC spectrum (C_6D_6) of **13**.

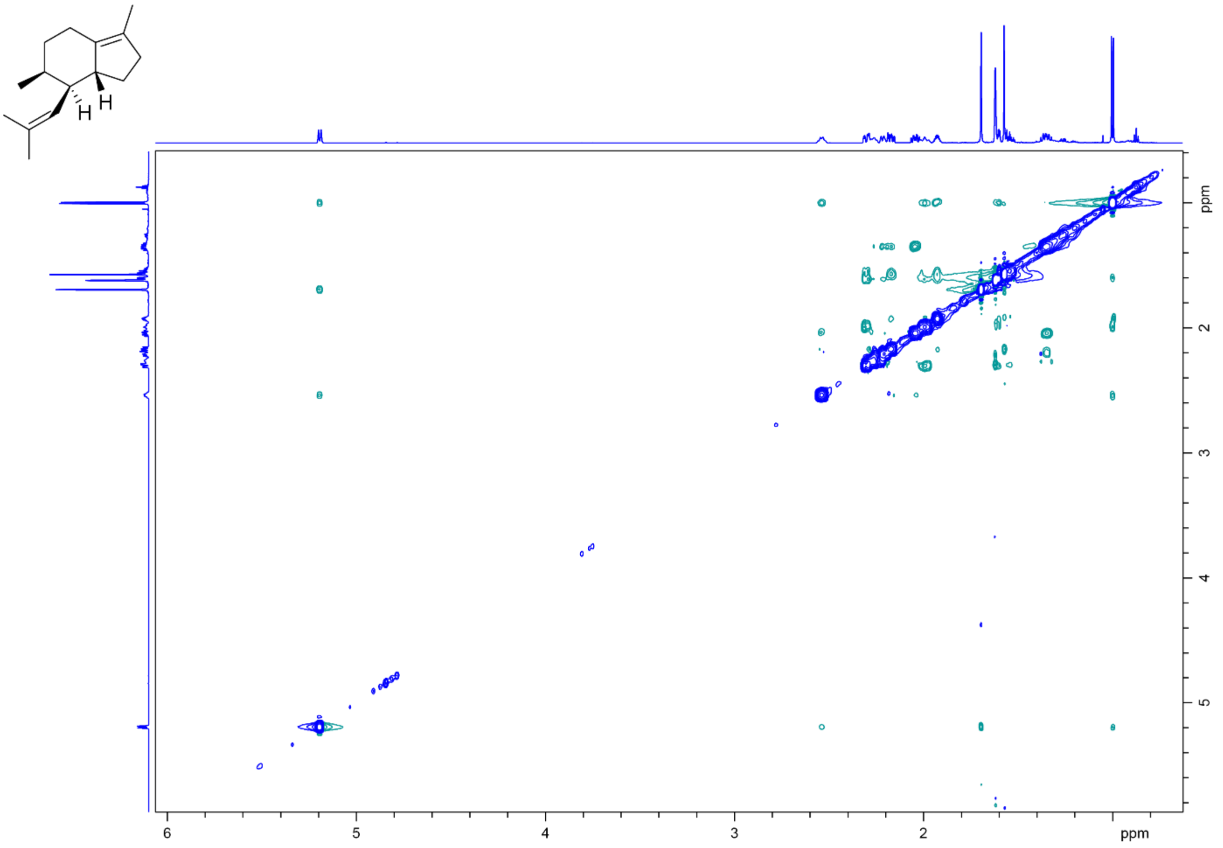

Figure S20. NOESY spectrum (C_6D_6) of **13**.

Table S4. NMR data of (–)-6-*epi*-pacifigorgia-1,10-diene (**14**) in C_6D_6 recorded at 298 K.

C[a]	type	^{13}C[b]	^{1}H[b]
1	CH	125.95	5.73 (br s)
2	C_q	132.24	–
3	CH	32.87	2.24 (m)
4	CH_2	30.98	1.75 (m)<br>1.60 (m)
5	CH_2	20.22	1.48 (m)<br>1.38 (m)
6	CH	46.44	2.26 (m)
7	CH	36.25	2.08 (m)
8	CH_2	32.00	1.66 (m)<br>1.37 (m)
9	CH_2	27.25	2.19 (m)<br>2.16 (m)
10	C_q	138.22	–
11	C_q	132.52	–
12	CH_3	25.52	1.71 (d, J = 1.3)
13	CH_3	19.79	1.60 (d, J = 1.1)
14	CH_3	15.06	1.74 (d, J = 7.1)
15	CH_3	20.65	1.07 (d, J = 7.1)

[a] Carbon numbering as shown in Figure S21 indicates the origin of each carbon from FPP by same number. [b] Chemical shifts δ in ppm. Multiplicity: s = singlet, d = doublet, m = multiplet, br = broad. Coupling constants *J* are given in Hertz.

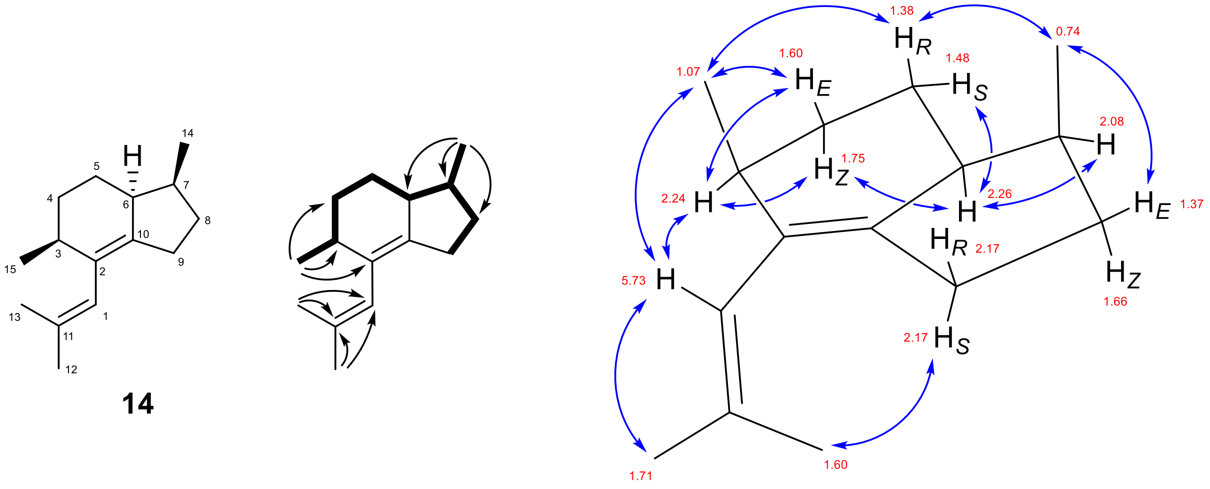

Figure S21. Structure elucidation of (–)-6-*epi*-pacifigorgia-1,10-diene (**14**). Bold: $^{1}H,^{1}H$-COSY, single headed arrows: key HMBC, and blue double headed arrows: NOESY correlations. H_R, H_S, H_E and H_Z indicate the results from stereoselective labelling experiments (Figures S57 and S63).

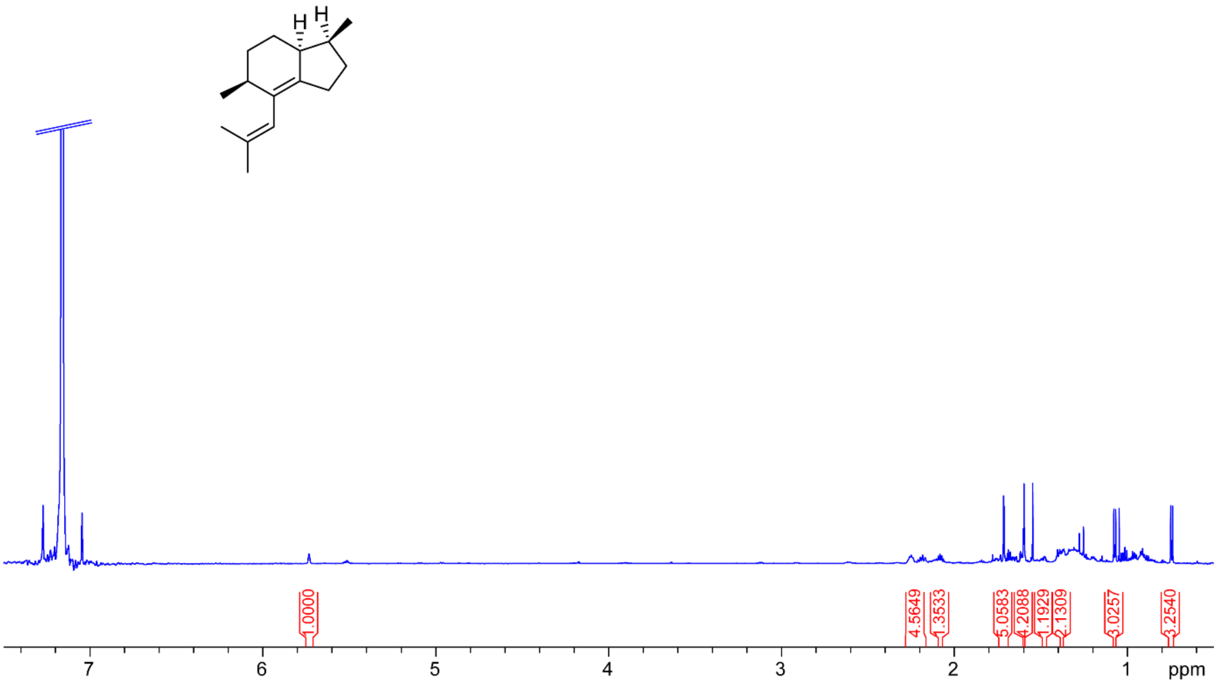

Figure S22. ^{1}H-NMR spectrum of **14** (700 MHz, C_6D_6).

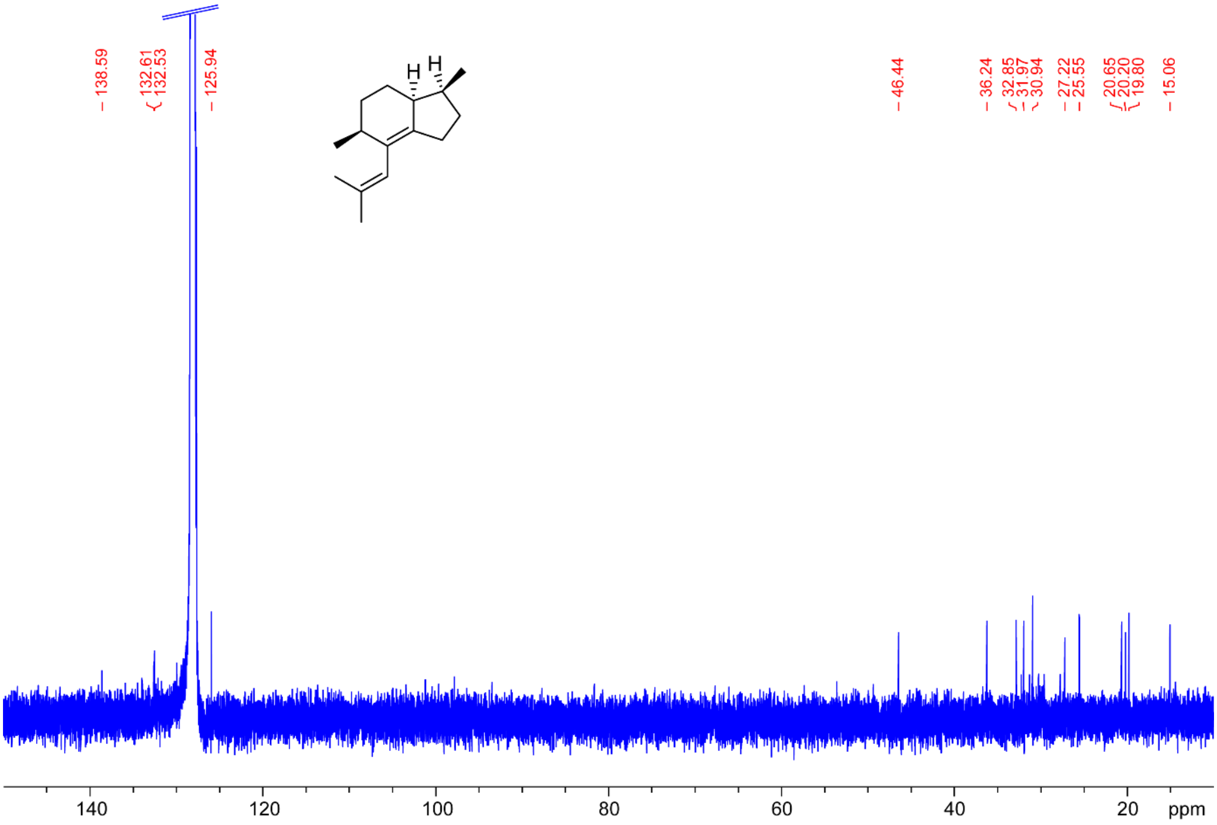

Figure S23. ^{13}C-NMR spectrum of **14** (176 MHz, C_6D_6).

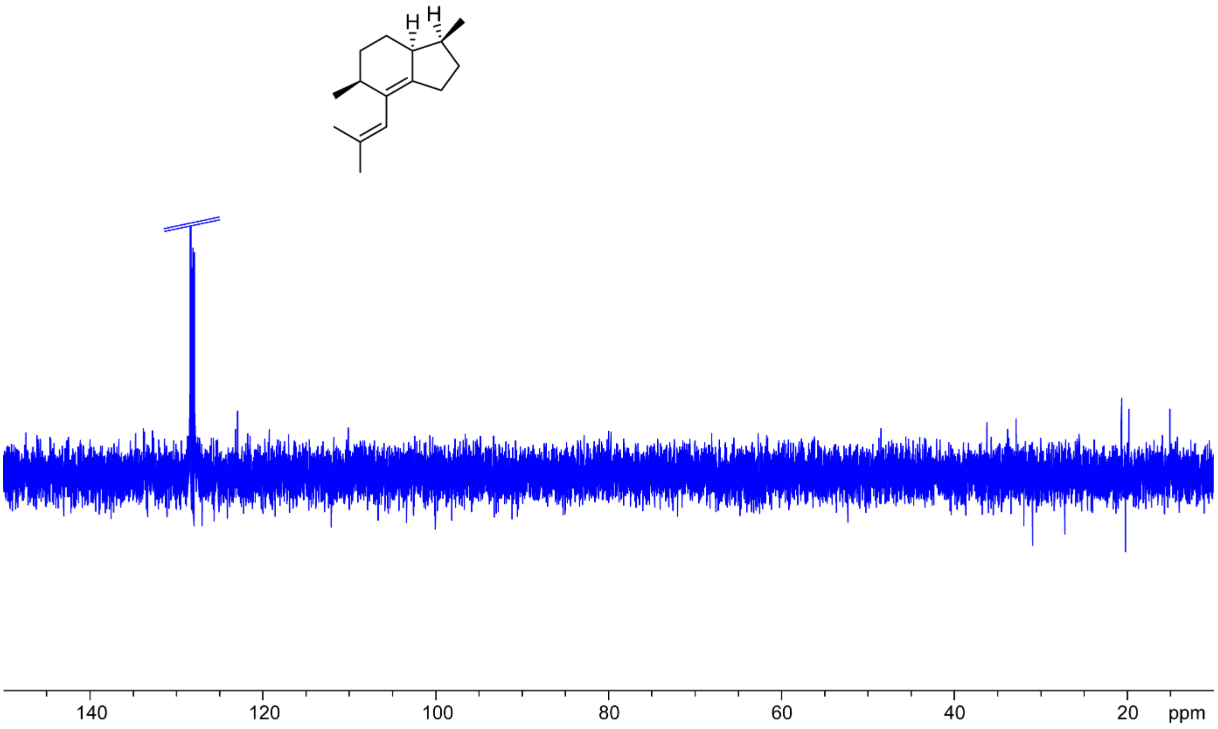

Figure S24. ^{13}C-DEPT135 spectrum of **14** (176 MHz, C_6D_6).

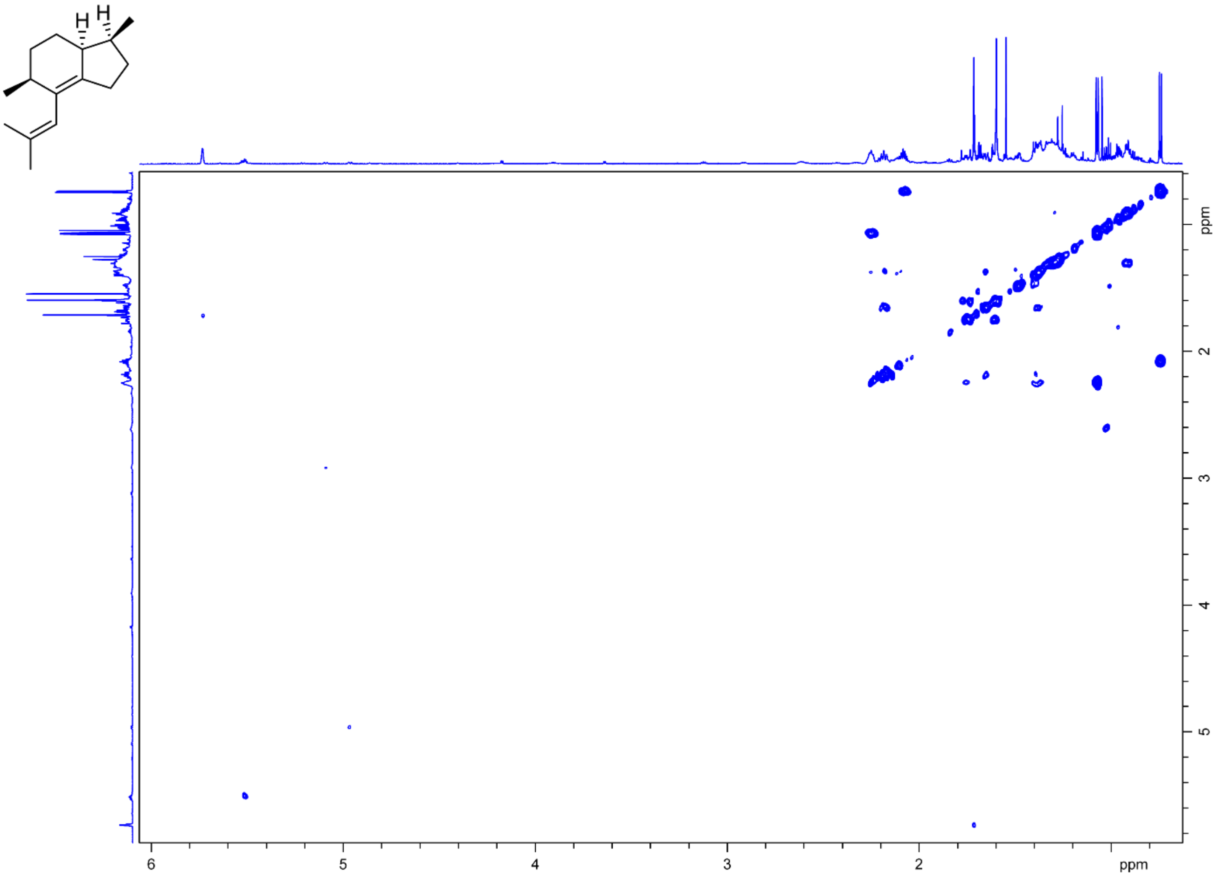

Figure S25. ^{1}H,^{1}H-COSY spectrum (C_6D_6) of **14**.

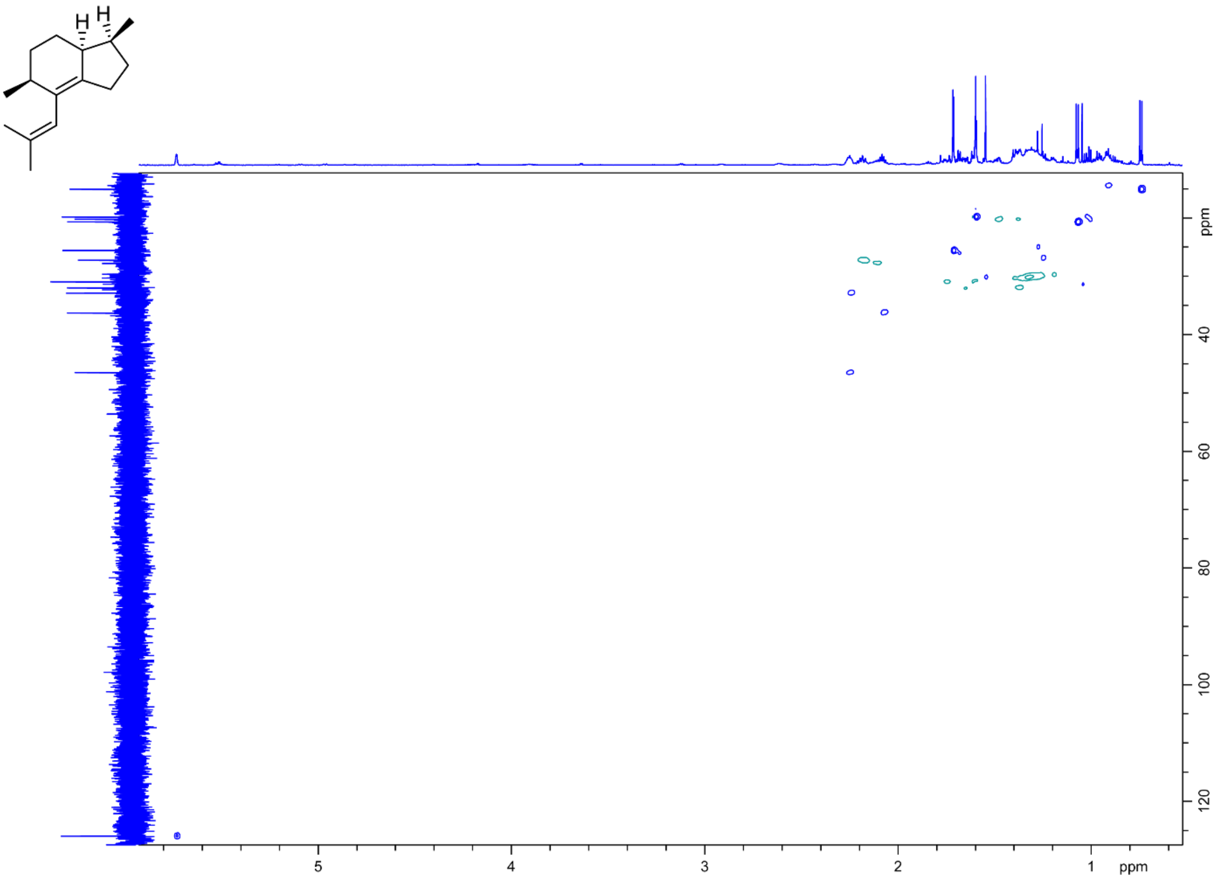

Figure S26. HSQC spectrum (C_6D_6) of **14**.

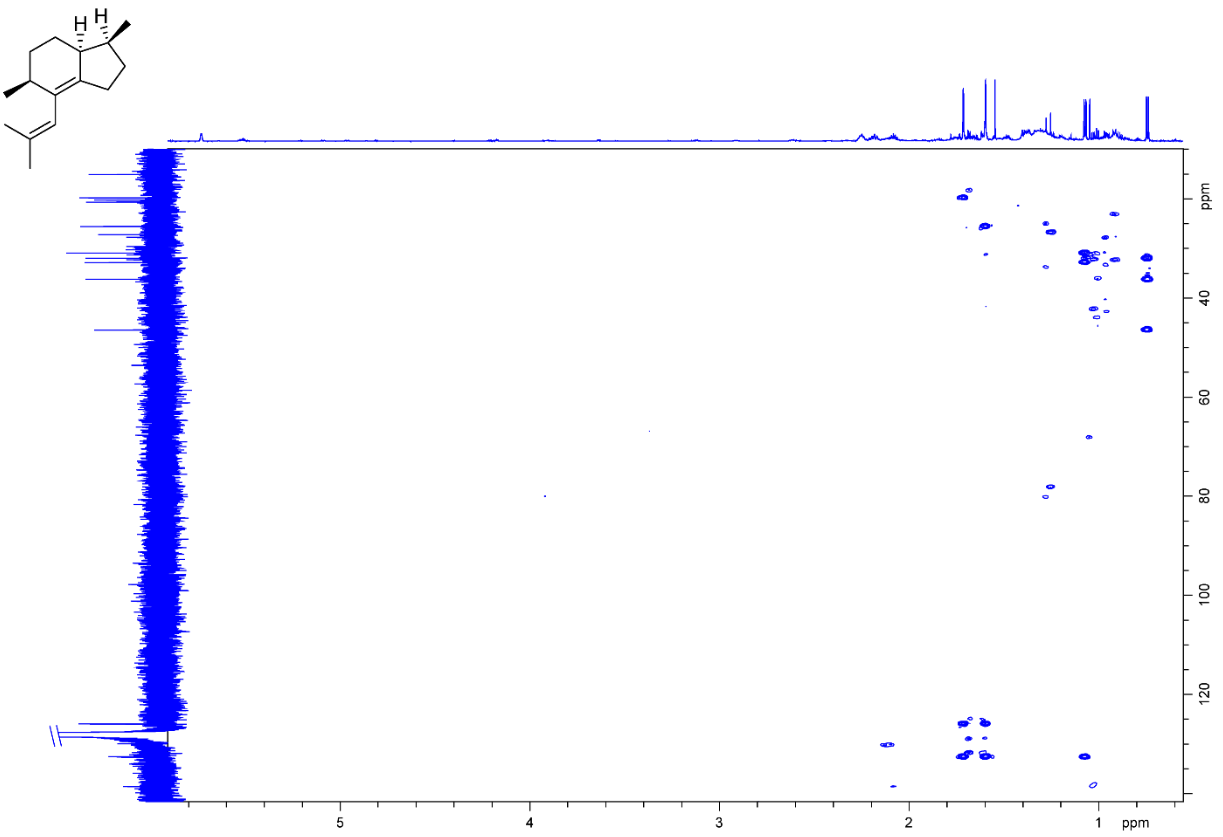

Figure S27. HMBC spectrum (C_6D_6) of **14**.

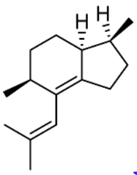

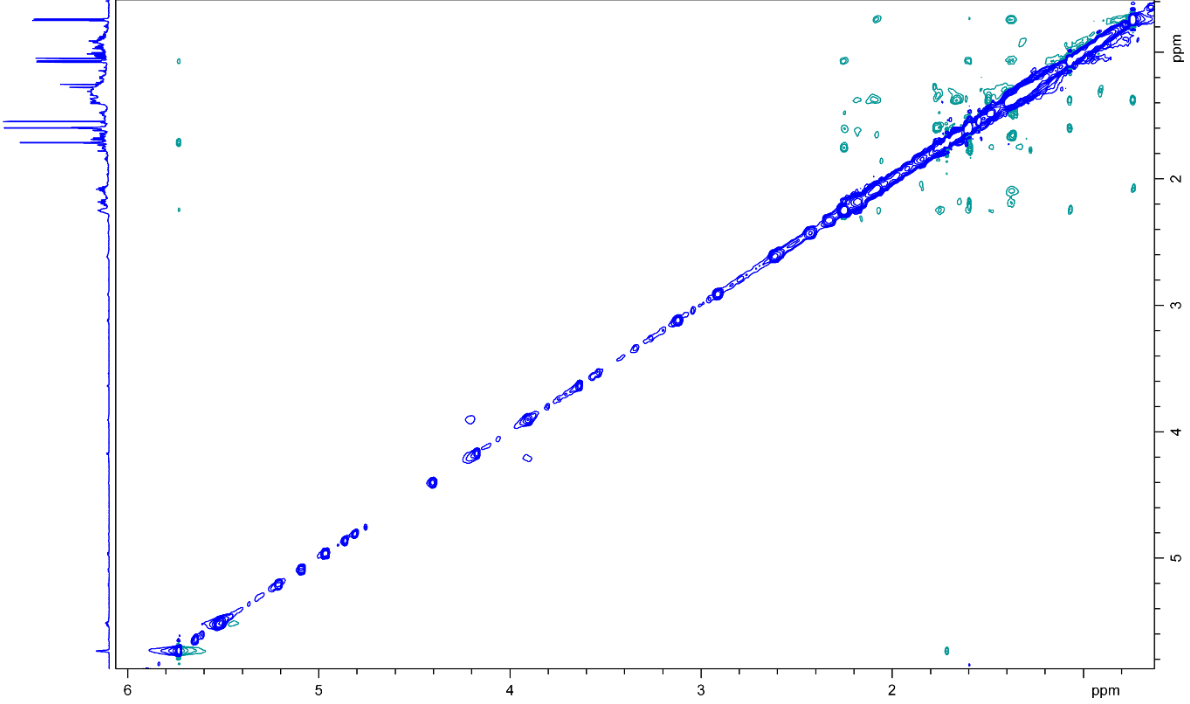

Figure S28. NOESY spectrum (C_6D_6) of **14**.

Table S5. NMR data of (–)-ledene (**15**) in C_6D_6 recorded at 298 K.

C[a]	type	^{13}C[b]	^{1}H[b]
1	CH	30.86	0.69 (dd, J = 11.2, 9.5)
2	CH	40.34	2.53 (dd, J = 10.5, 8.2)
3	CH	37.68	1.99 (m)
4	CH_2	33.00	1.63 (m)
			1.26 (m)
5	CH_2	32.16	2.44 (m)
			2.09 (m)
6	C_q	139.74	–
7	C_q	124.61	–
8	CH_2	37.03	2.21 (m)
9	CH_2	22.64	1.70 (m)
			1.64 (m)
10	CH	25.95	0.56 (ddd, J = 11.7, 9.5, 5.0)
11	C_q	18.78	–
12	CH_3	28.71	1.01 (s)
13	CH_3	15.89	1.06 (s)
14	CH_3	22.14	1.60 (s)
15	CH_3	16.05	1.00 (d, J = 6.9)

[a] Carbon numbering as shown in Figure S29 indicates the origin of each carbon from FPP by same number. [b] Chemical shifts δ in ppm. Multiplicity: s = singlet, d = doublet, m = multiplet. Coupling constants J are given in Hertz.

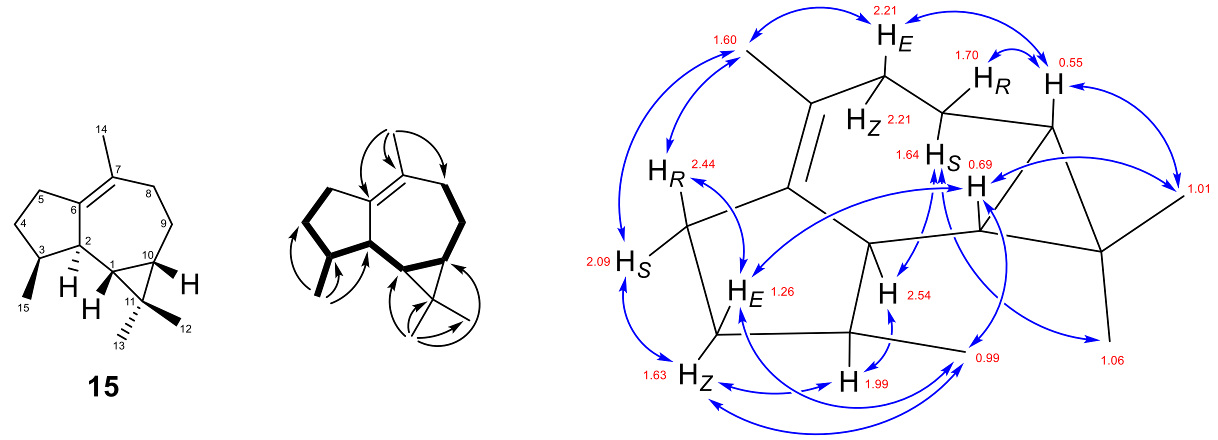

15

Figure S29. Structure elucidation of ledene (**15**). Bold: $^{1}H,^{1}H$-COSY, single headed arrows: key HMBC, and blue double headed arrows: NOESY correlations. H_R, H_S, H_E and H_Z indicate the results from stereoselective labelling experiments (Figures S58 and S64).

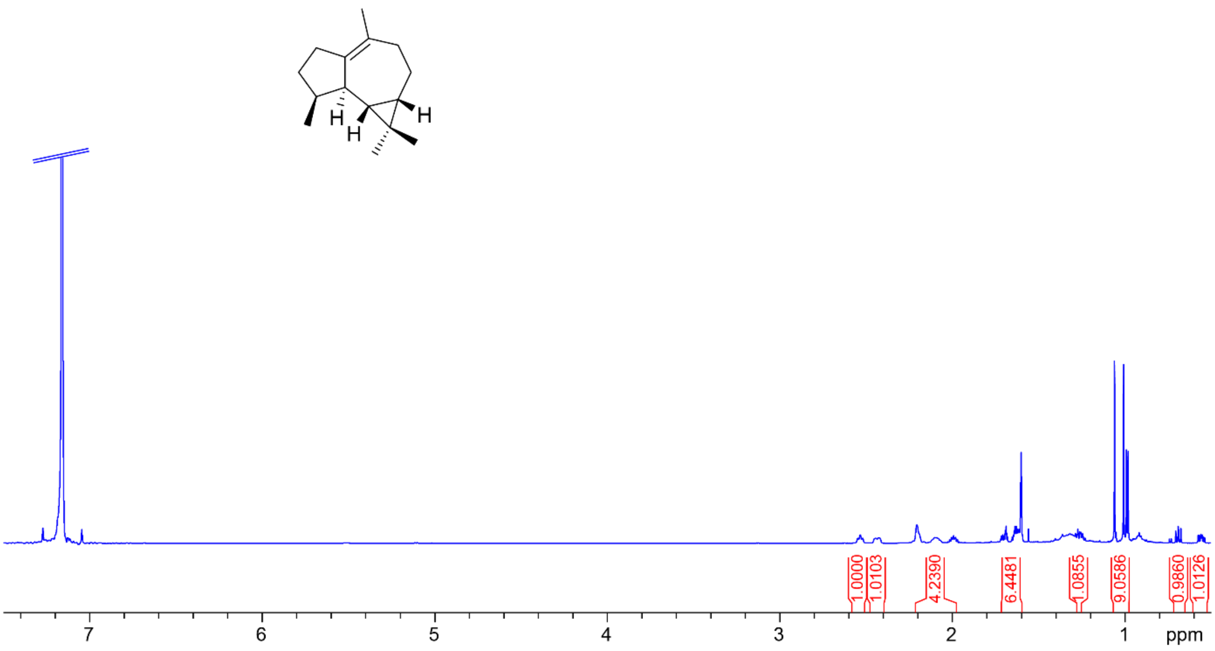

Figure S30. ^{1}H-NMR spectrum of **15** (700 MHz, C_6D_6).

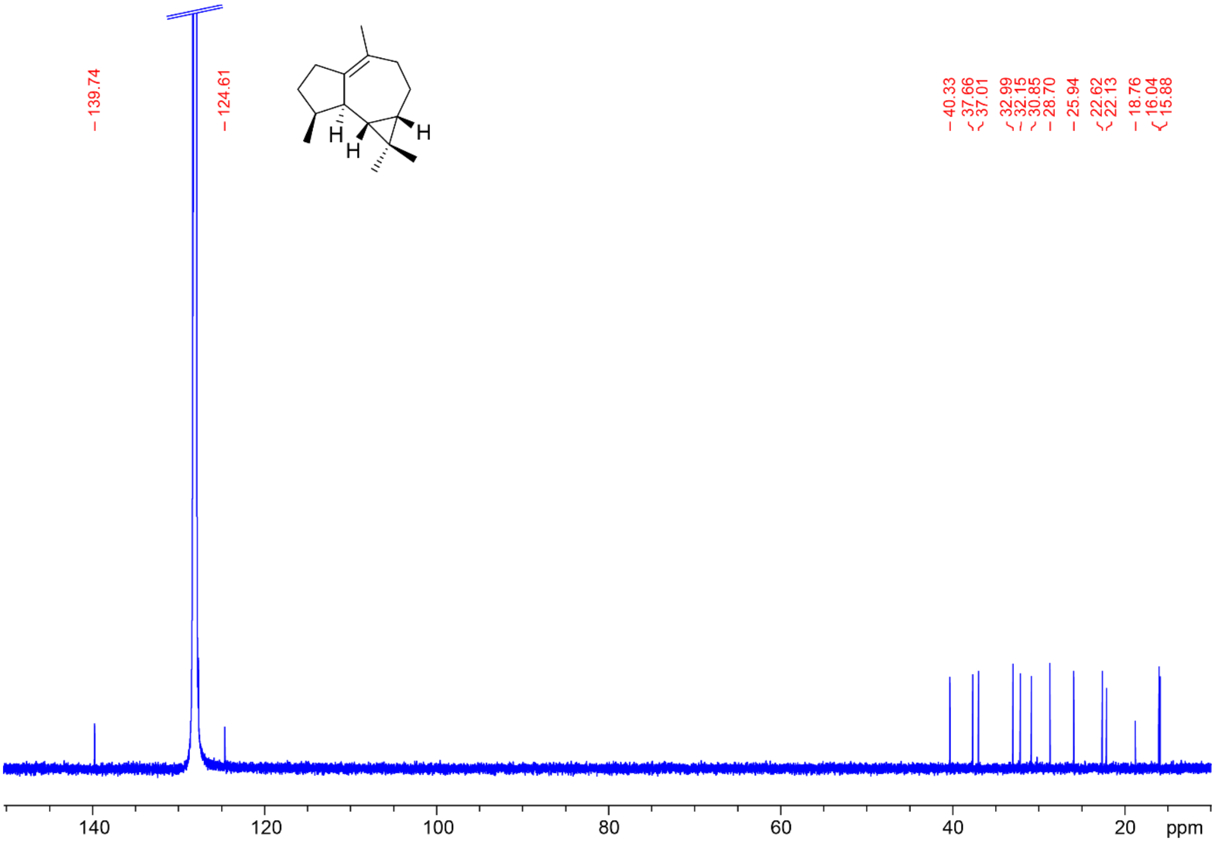

Figure S31. ^{13}C-NMR spectrum of **15** (176 MHz, C_6D_6).

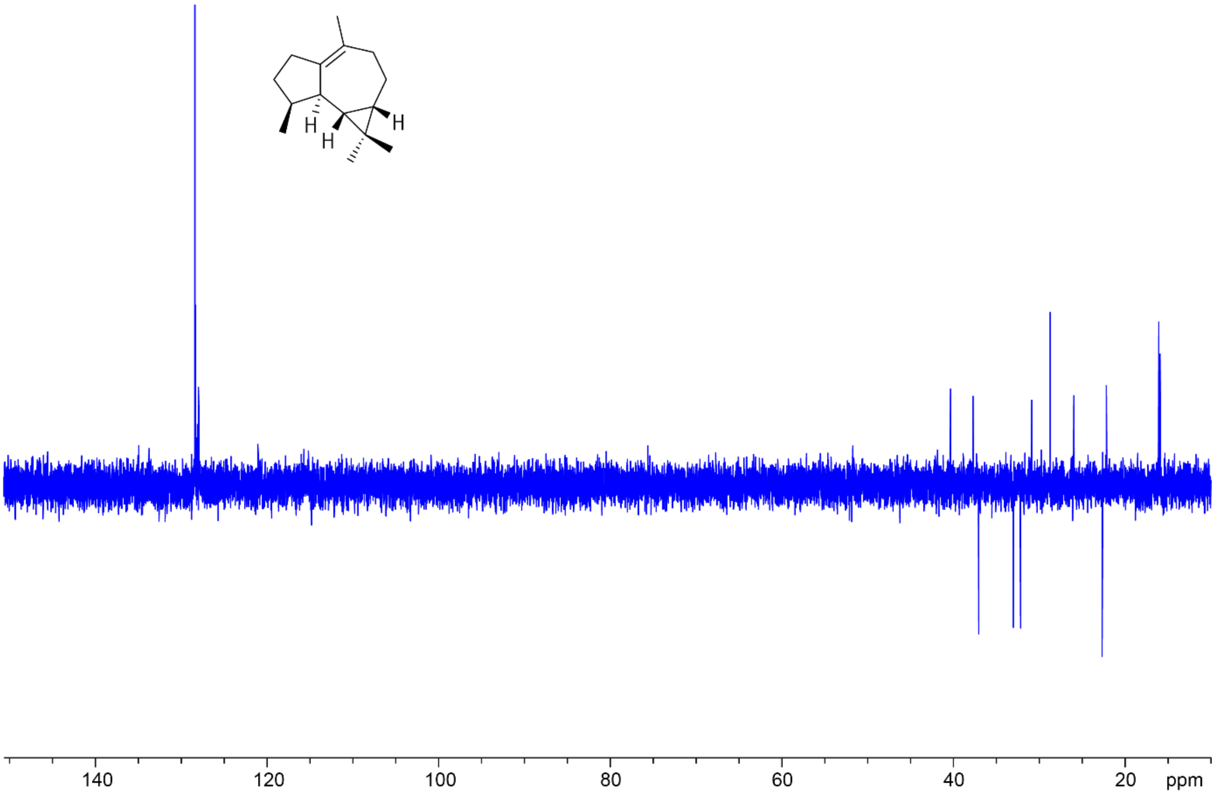

Figure S32. ^{13}C-DEPT135 spectrum of **15** (176 MHz, C_6D_6).

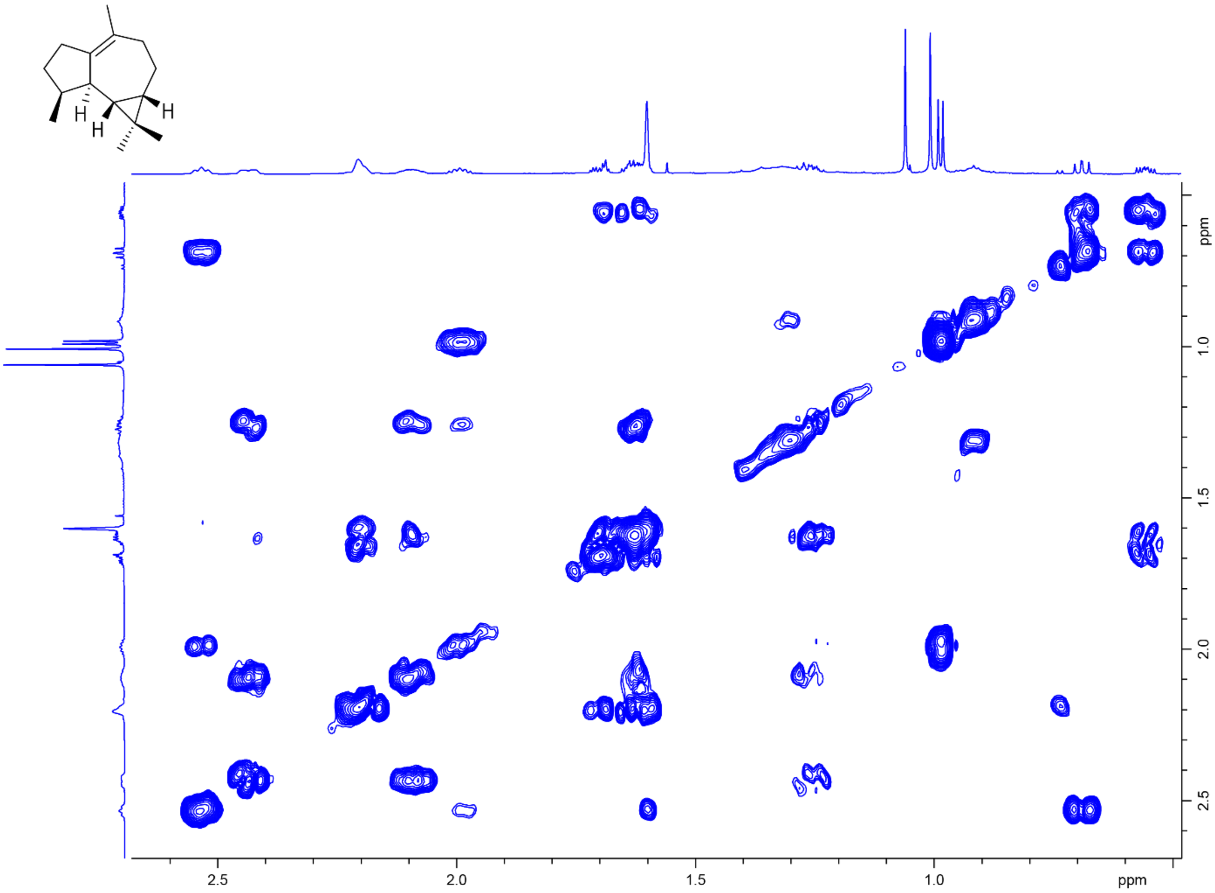

Figure S33. ^{1}H,^{1}H-COSY spectrum (C_6D_6) of **15**.

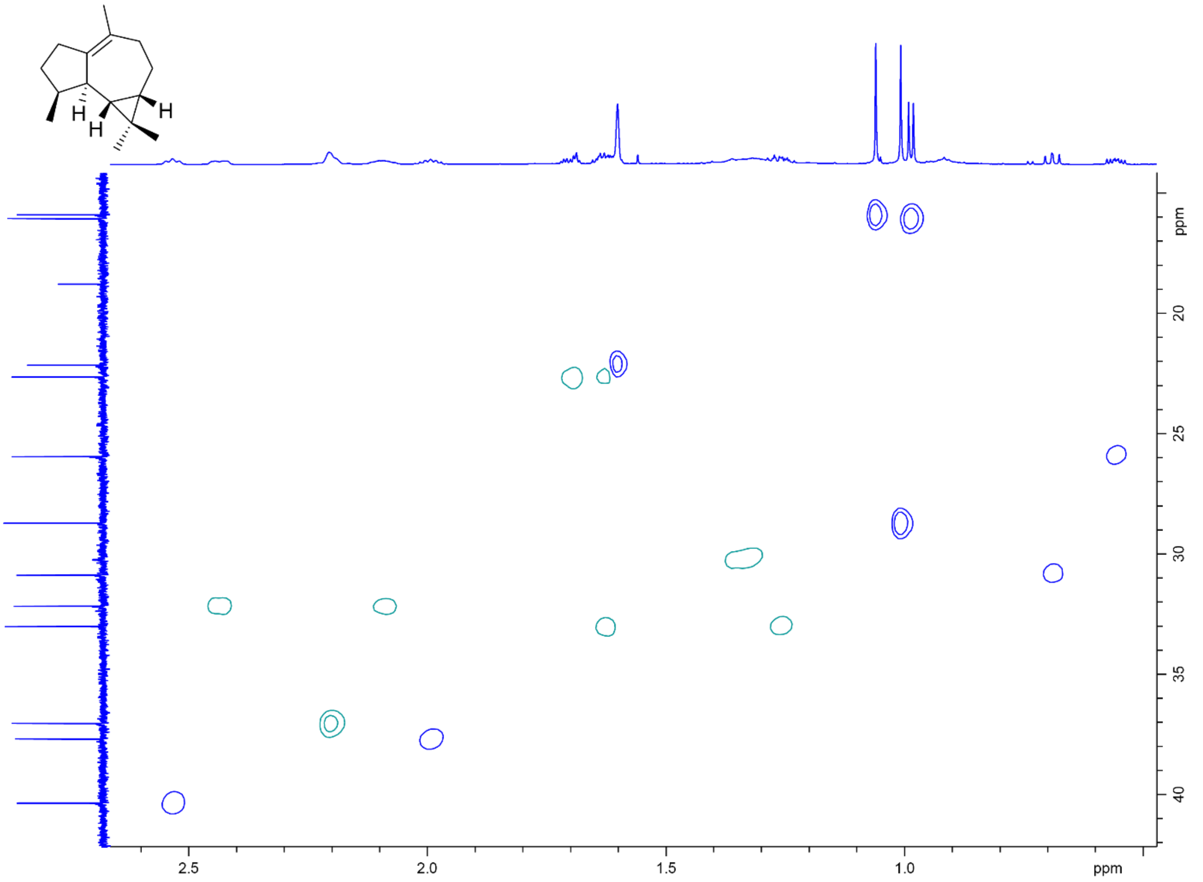

Figure S34. HSQC spectrum (C_6D_6) of **15**.

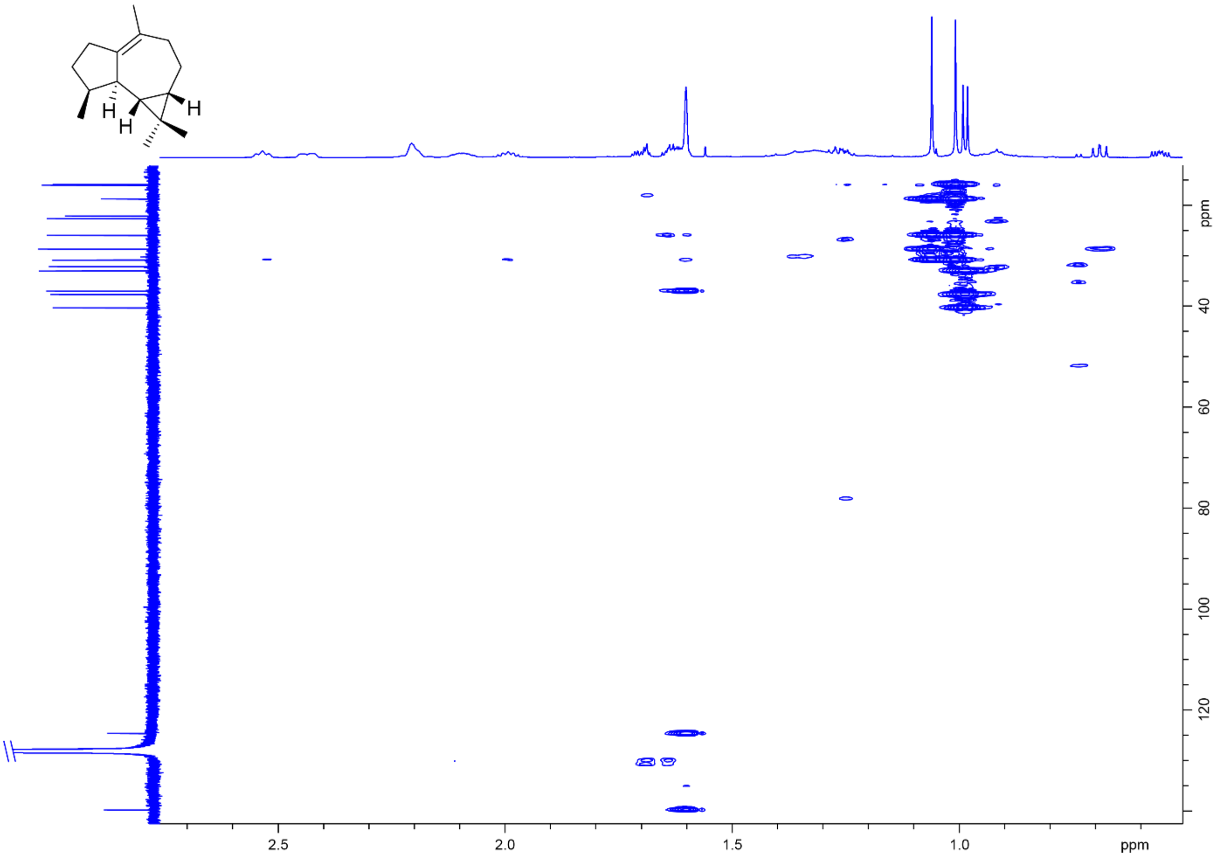

Figure S35. HMBC spectrum (C_6D_6) of **15**.

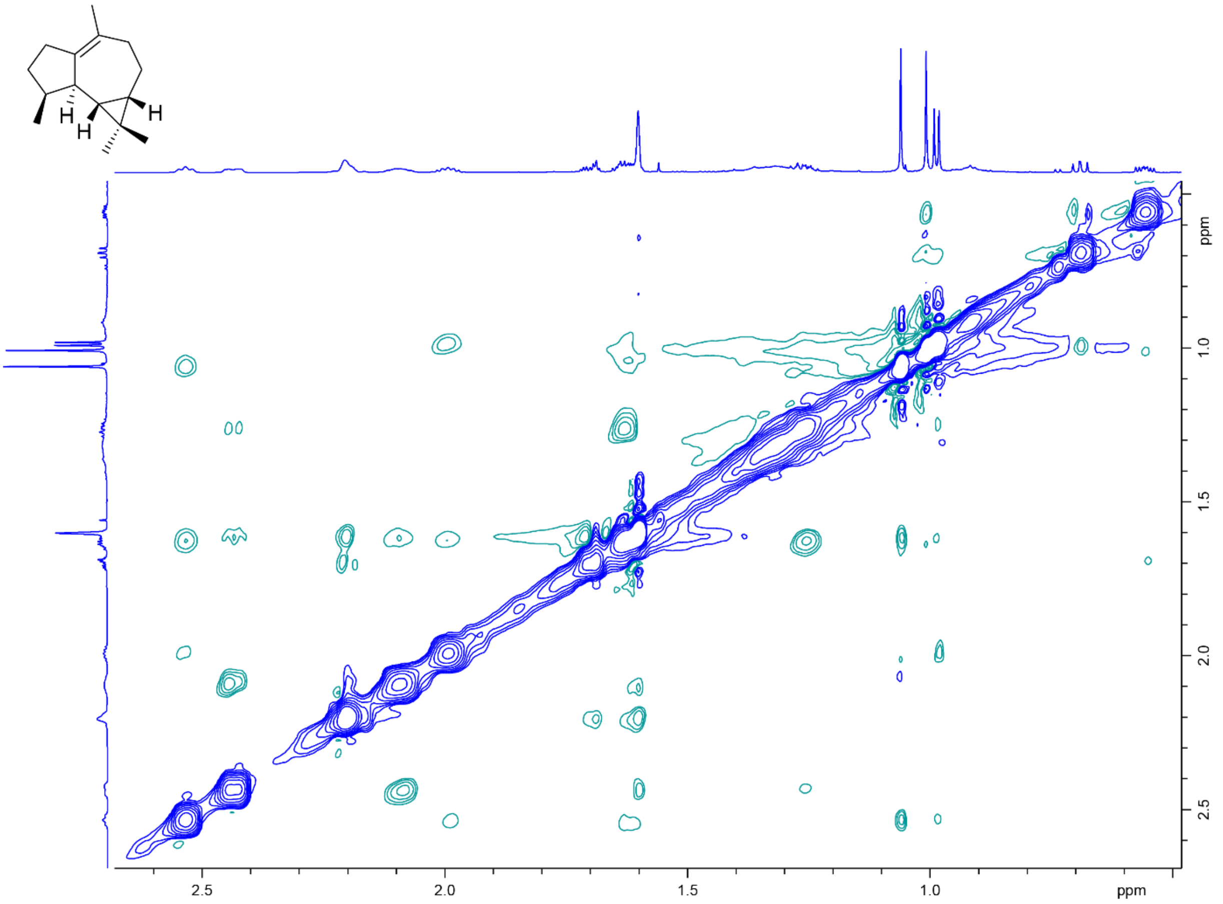

Figure S36. NOESY spectrum (C_6D_6) of **15**.

Molecular simulation

AlphaFold3 was used to construct a structural model of BgPgS and to predict its substrate pocket (Figure S37).[34] The structural model of the ligand FPP was downloaded from the PubChem database (https://pubchem.ncbi.nlm.nih.gov/) and docked into the cofactor-binding site of BgPgS using AutoDock Vina v1.5.6.[35] The three Mg^{2+} cations were taken from the aligned crystal structure of AhCS (PDB: 7XKW). PyMOL 2.1 (http://www.pymol.org) was used to view the molecular interactions and to process the image.

Site-directed mutagenesis of BgPgS

Site-directed mutagenesis was performed through PCR using pairs of reverse complement mutational primers (Table S1) to amplify the entire expression plasmids pESC-BgPgS. Through the same strategy the in vitro expression plasmids pMAL-BgPgS-G299A were derived from the templates pMAL-BgPgS. The resulting PCR product was incubated with *Dpn*I to digest the parent vector, followed by transformation into *E. coli* DH5α*.* All mutated plasmids were isolated and mutations were verified by DNA sequencing. The pESC-derived plasmids were introduced into *S. cerevisiae* for in vivo production and the pMAL-c6T-derived plasmids were introduced into *E. coli* BL21(DE3) by electroporation for heterologous expression and in vitro incubations.

Heterologous expression of BgPgS enzyme variants in the *S. cerevisiae* production strain

Gene integrations for the construction of engineered yeast strains were conducted using the LiOAc/salmon sperm carrier DNA/polyethyleneglycol method[36] and verified using diagnostic polymerase chain reaction. Seed cultures of *S. cerevisiae* containing the pESC-derived plasmids with the mutated *BgPgS* gene were cultured overnight at 30 °C and 220 rpm. The seed cultures were used to inoculate production cultures in YPD medium (50 mL) with an initial OD_{600} = 0.1. Fermentation was performed at 30 °C and 220 rpm for 3 days. The cultures were extracted with hexane (2 x 50 mL), the extracts were combined and concentrated. The residue was redissolved in hexane (1 mL) and the obtained solution was analysed by GC/MS. All experiments were performed in triplicates for the determination of relative productions (mean ± standard deviation). The relative production of all compounds is shown in Figure S38 and summarised in Table S6.

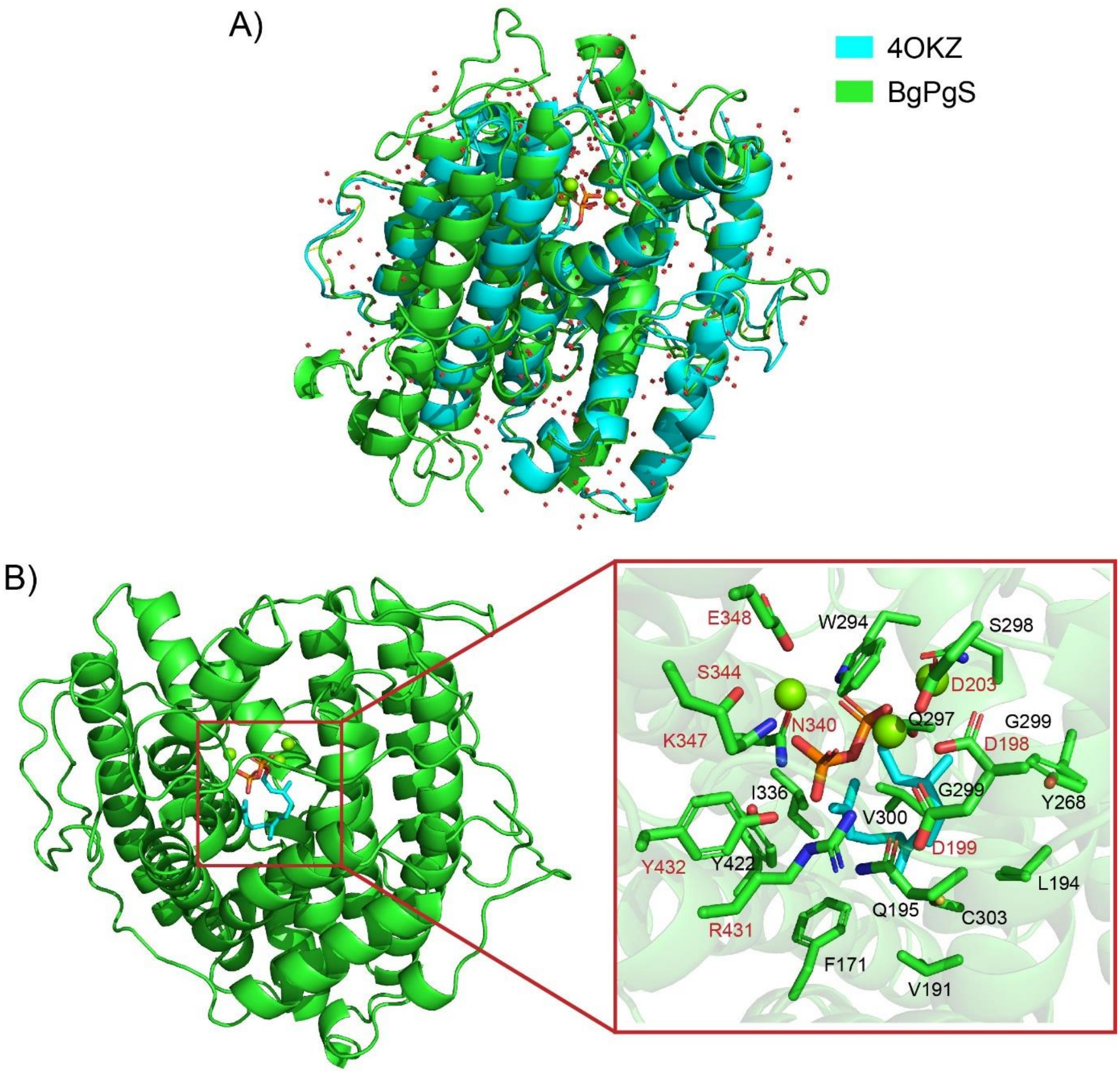

Figure S37. A) AlphaFold3 model of BgPgS superimposed with 4OKZ. The three Mg^{2+} cations (green spheres) were added from an aligned structure of 4OKZ. The structural model of the ligand FPP (cyan) was downloaded from the PubChem database (https://pubchem.ncbi.nlm.nih.gov/) and docked into the cofactor-binding site of BgPgS using AutoDock Vina v1.5.6. B) Active site architecture of BgPgS docked with FPP (cyan). Active site amino acid residues are shown in green.

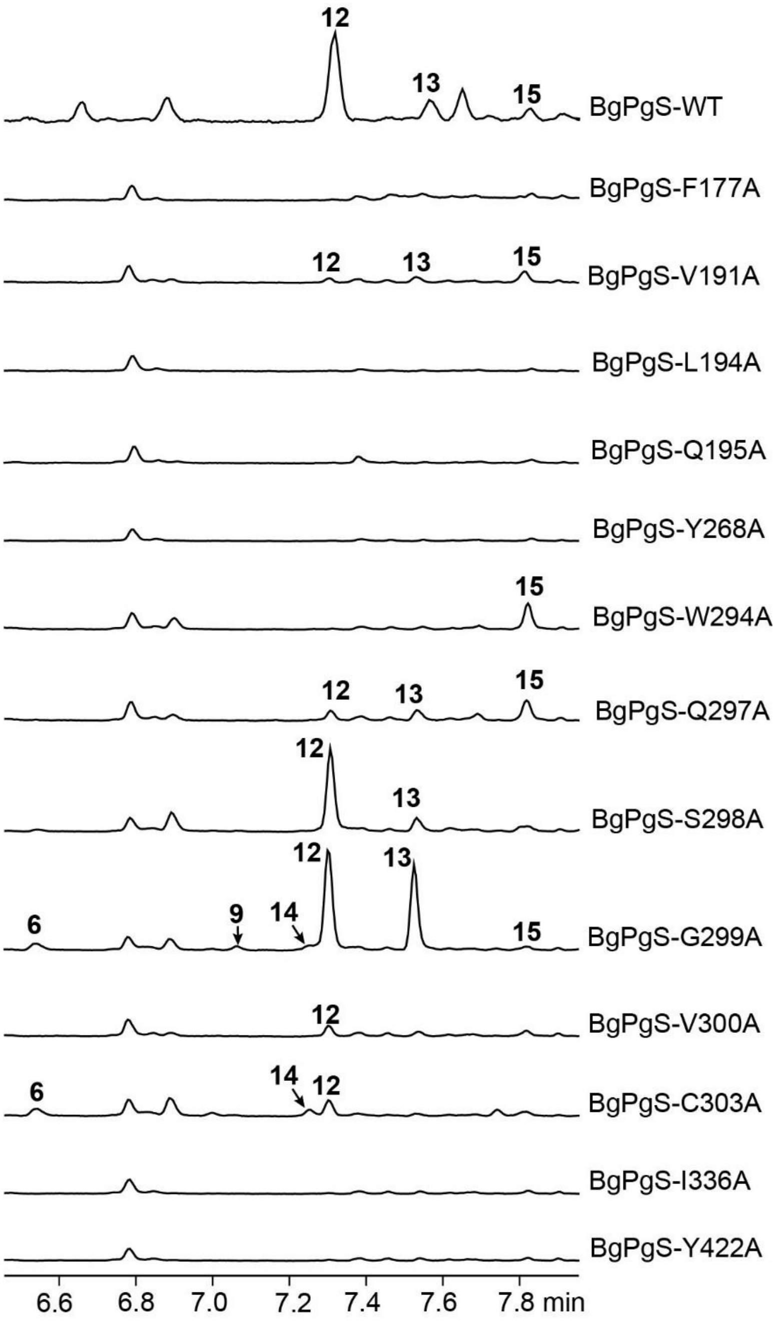

Figure S38. Alanine scanning of BgPgS active site residues. Total ion chromatograms of extracts from *S. cerevisiae* cultures expressing BgPgS and its variants.

Table S6. Relative compound production by BgPgS and its enzyme variants in vivo.

Variant	6	9	12	13	14	15
BgPgS-WT	–	–	100.0±1.6%	21.1±0.9%	–	8.9±0.4%
BgPgS-F177A	–	–	–	–	–	–
BgPgS-V191A	–	–	2.6±0.2%	5.2±0.3%	–	9.7±0.5%
BgPgS-L194A	–	–	–	–	–	–
BgPgS-Q195A	–	–	–	–	–	–
BgPgS-Y268A	–	–	–	–	–	–
BgPgS-W294A	–	–	–	–	–	24.7±0.8%
BgPgS-Q297A	–	–	6.3±0.3%	8.5±0.4%	–	17.0±0.6%
BgPgS-S298A	–	–	77.7±2.3%	12.5±0.6%	–	–
BgPgS-G299A	9.5±0.3%	4.6±0.2%	96.8±6.9%	78.6±2.1%	8.8±0.5%	4.3±0.2%
BgPgS-V300A	–	–	8.6±0.3%	–	–	–
BgPgS-C303A	8.0±0.2%	–	8.7±0.3%	–	4.7±0.2%	–
BgPgS-I336A	–	–	–	–	–	–
BgPgS-Y422A	–	–	–	–	–	–

[a] The production of **12** by wildtype BgPgS was arbitrarily set to 100%. The data are mean and standard deviations from triplicates.

Table S7. NMR data of (–)-pacifigorgia-1(6),10-diene (**9**) in C_6D_6 recorded at 298 K.

C[a]	type	^{13}C[b]	^{1}H[b]
1	C_q	124.94	–
2	CH	40.35	2.91 (m)
3	CH	33.39	1.81 (m)
4	CH_2	27.88	1.51 (m)
5	CH_2	24.04	2.10 (m)<br>1.85 (m)
6	C_q	137.99	–
7	CH	42.19	2.60 (m)
8	CH_2	32.23	2.10 (m)<br>1.37 (m)
9	CH_2	33.53	2.43 (m)<br>2.10 (m)
10	C_q	137.60	–
11	C_q	131.87	–
12	CH_3	26.18	1.68 (d, J = 1.3)
13	CH_3	18.26	1.62 (d, J = 1.3)
14	CH_3	19.83	1.02 (d, J = 7.0)
15	CH_3	18.77	0.96 (d, J = 7.0)

[a] Carbon numbering as shown in Figure S39 indicates the origin of each carbon from FPP by same number. [b] Chemical shifts δ in ppm. Multiplicity: s = singlet, d = doublet, m = multiplet. Coupling constants J are given in Hertz.

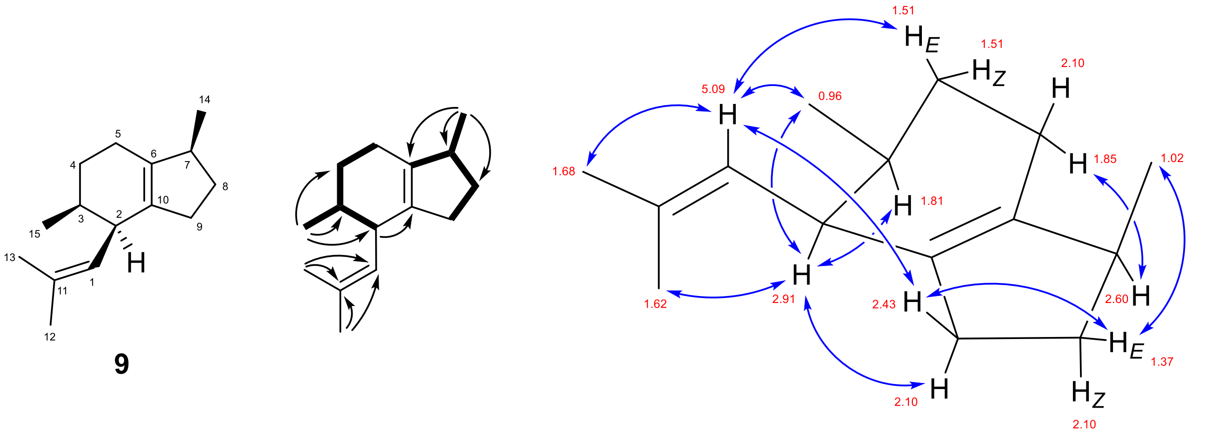

Figure S39. Structure elucidation of (–)-pacifigorgia-1(6),10-diene (**9**). Bold: $^{1}H,^{1}H$-COSY, single headed arrows: key HMBC, and blue double headed arrows: NOESY correlations. H_E and H_Z indicate the results from stereoselective labelling experiments (Figure S59).

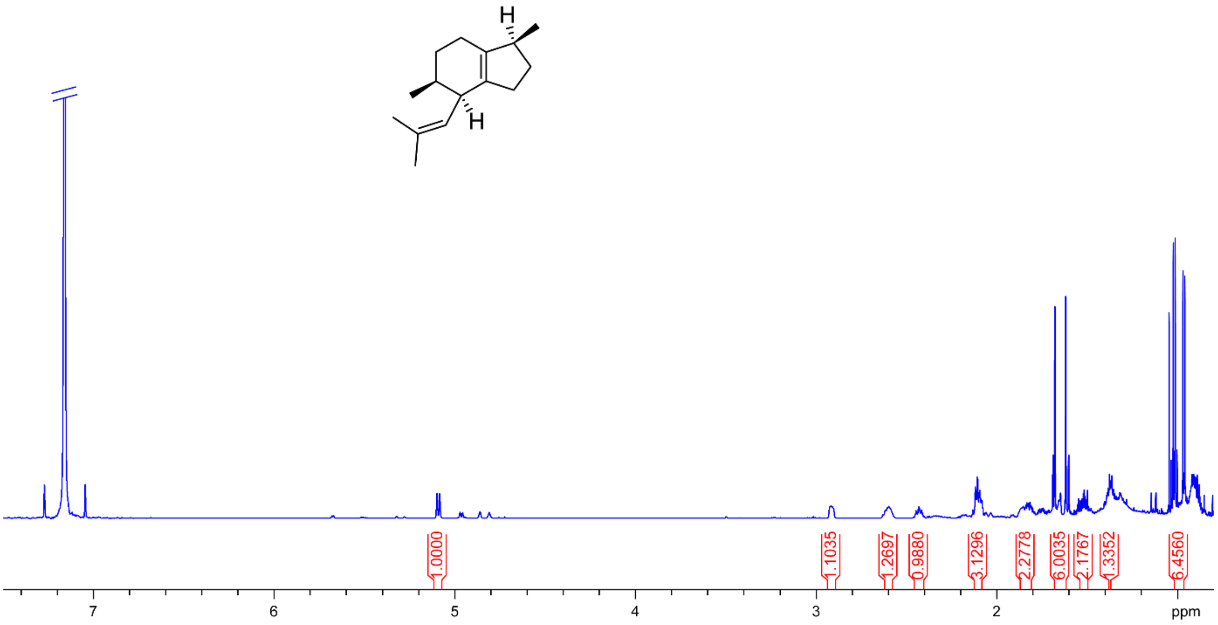

Figure S40. ^{1}H-NMR spectrum of **9** (700 MHz, C_6D_6).

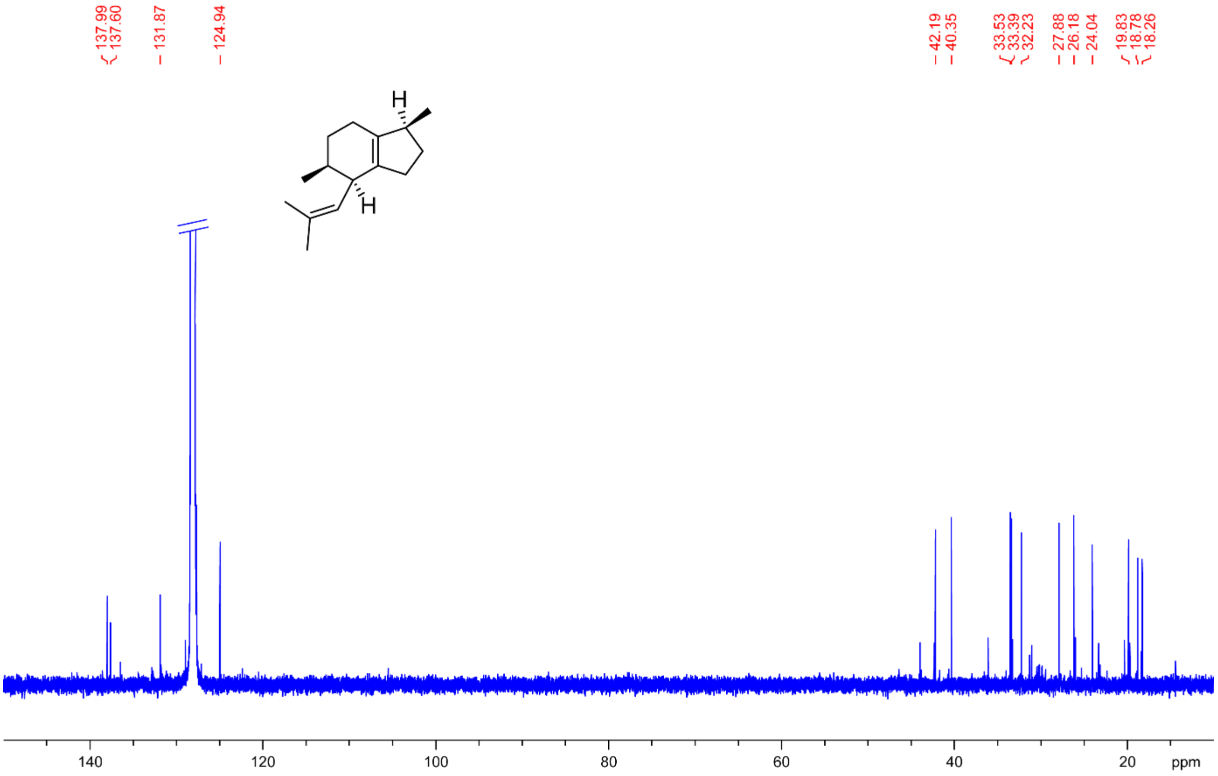

Figure S41. ^{13}C-NMR spectrum of **9** (176 MHz, C_6D_6).

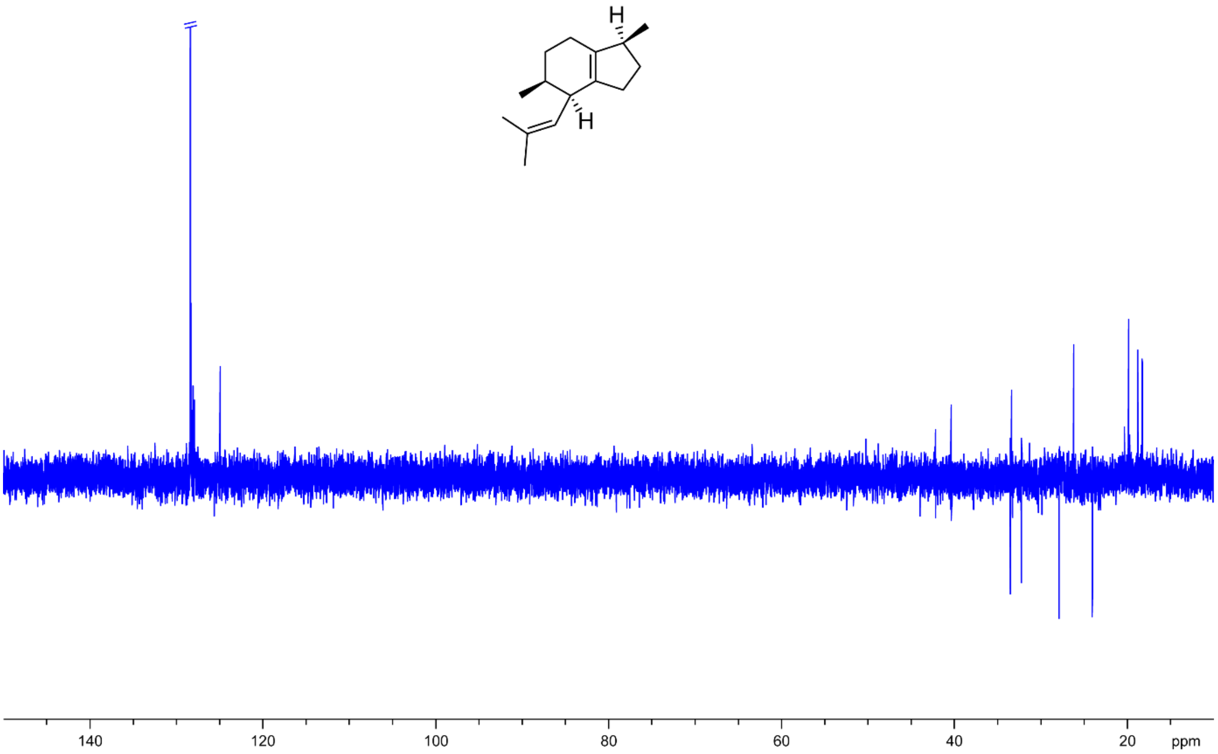

Figure S42. ^{13}C-DEPT135 spectrum of **9** (176 MHz, C_6D_6).

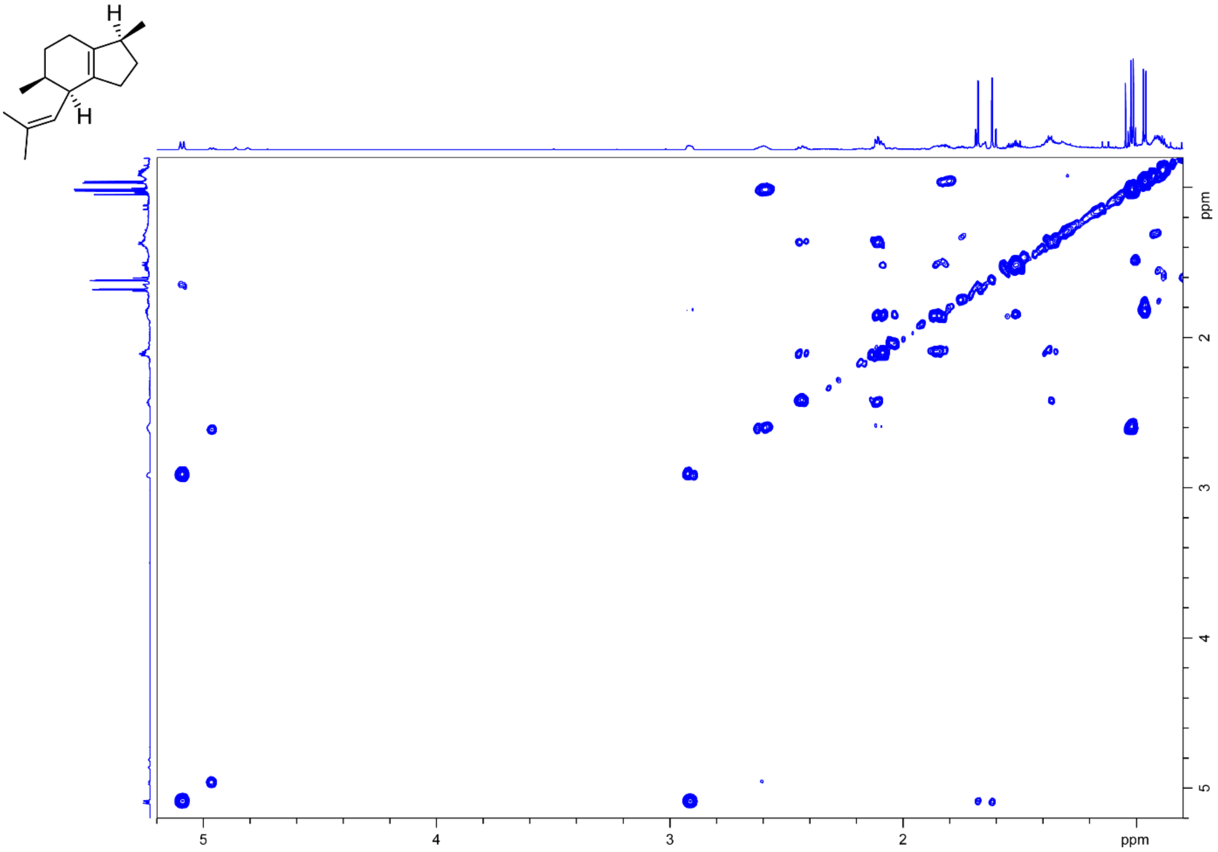

Figure S43. ^{1}H,^{1}H-COSY spectrum (C_6D_6) of **9**.

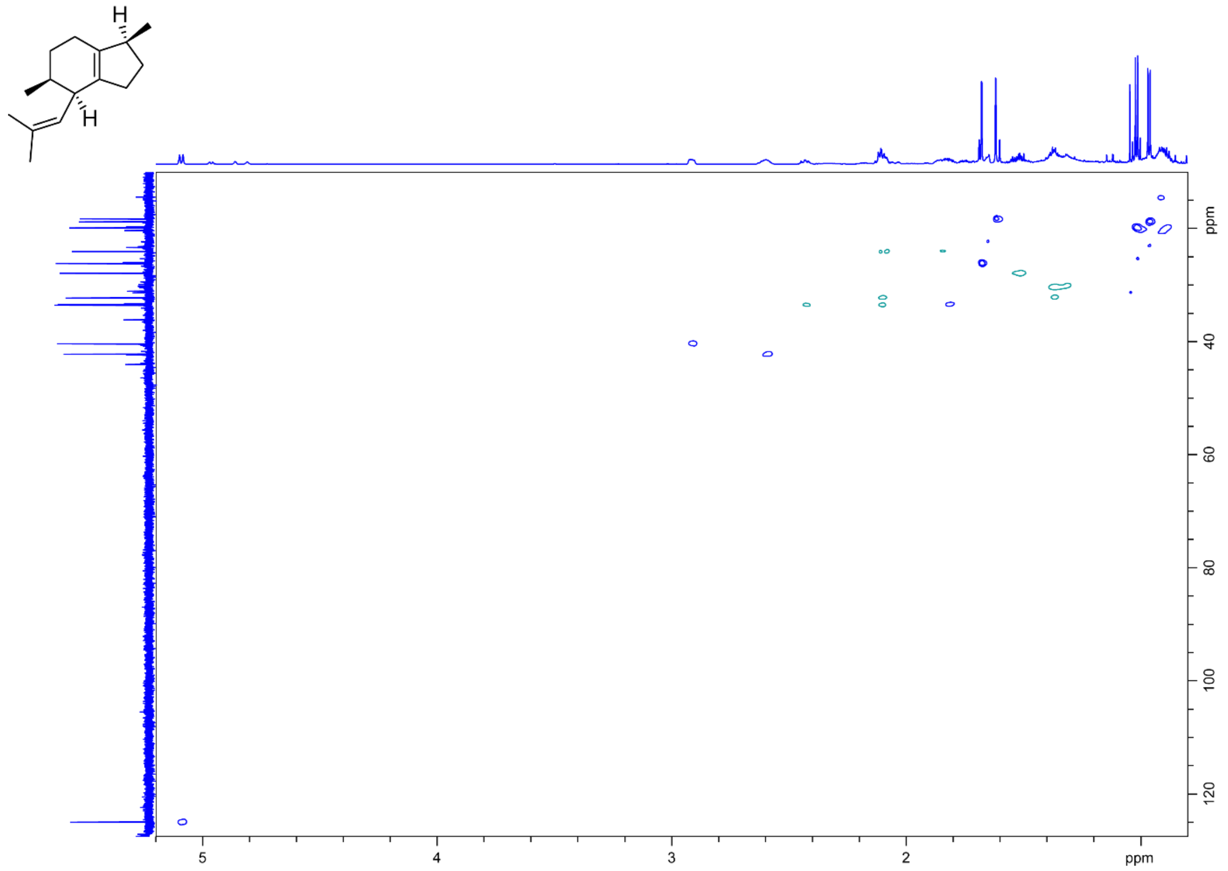

Figure S44. HSQC spectrum (C_6D_6) of **9**.

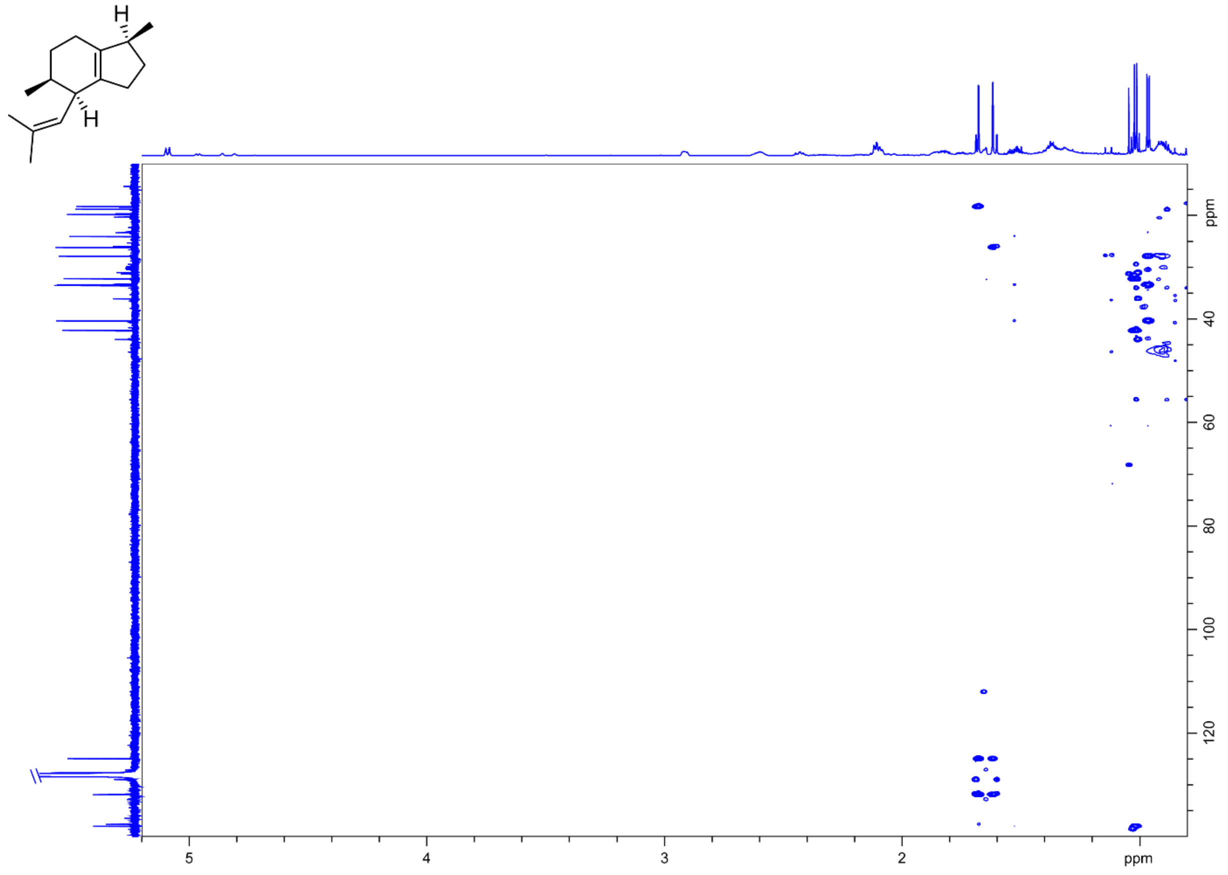

Figure S45. HMBC spectrum (C_6D_6) of **9**.

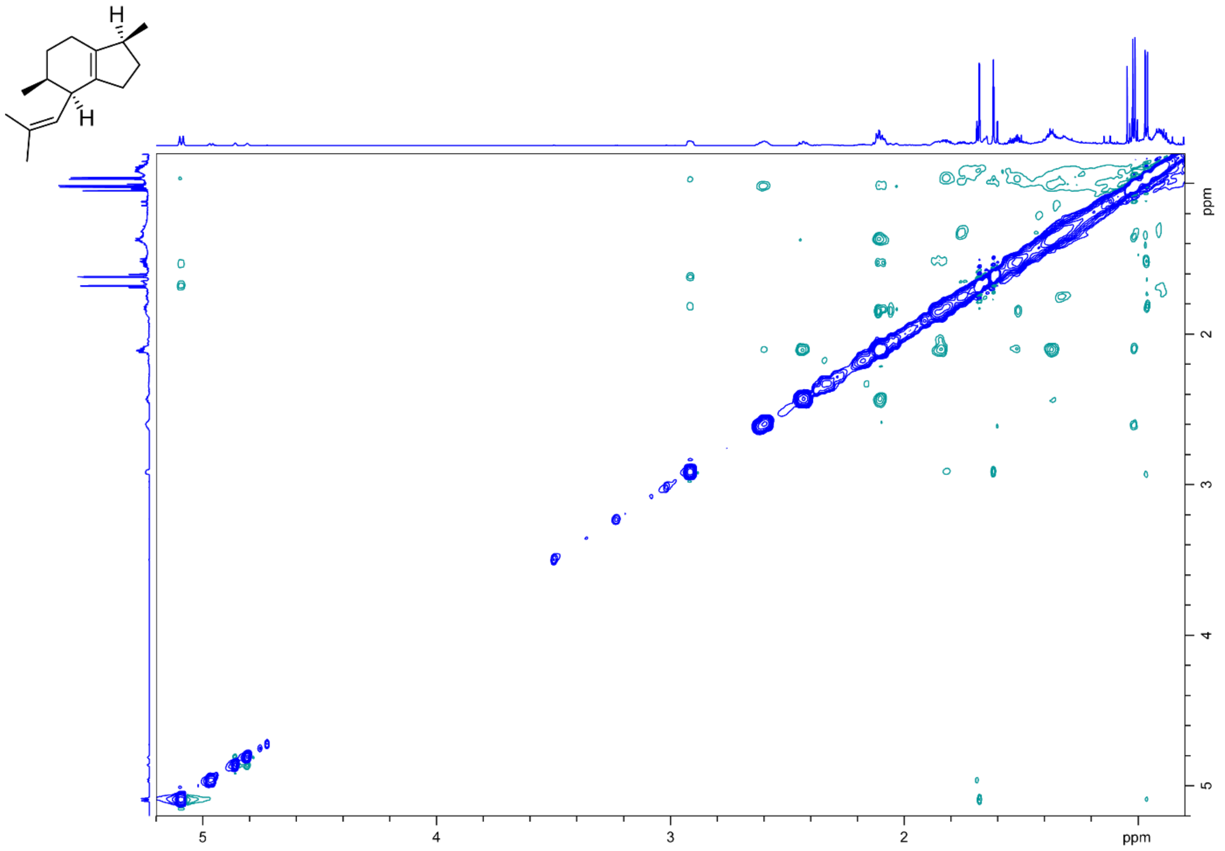

Figure S46. NOESY spectrum (C_6D_6) of **9**.

Table S8. NMR data of (–)-tamariscene (**6**) in C_6D_6 recorded at 298 K.

C[a]	type	^{13}C[b]	^{1}H[b]
1	CH	33.55	0.95 (m)
2	CH	30.06	1.07 (dd, *J* = 5.8, 5.8)
3	CH	28.95	1.81 (m)
4	CH_2	29.04	1.37 (m)<br>0.54 (m)
5	CH_2	30.31	1.69 (m)<br>0.72 (m)
6	CH	50.16	1.16 (m)
7	CH	44.66	1.51 (m)
8	CH_2	35.55	0.95 (m)
9	CH_2	30.40	1.62 (m)<br>1.50 (m)
10	C_q	34.14	–
11	C_q	144.69	–
12	CH_3	109.52	4.90 (br s)<br>4.71 (br s)
13	CH_3	24.42	1.74 (s)
14	CH_3	19.17	1.04 (d, *J* = 6.6)
15	CH_3	21.33	1.02 (d, *J* = 6.6)

[a] Carbon numbering as shown in Figure S47 indicates the origin of each carbon from FPP by same number (C12 and C13 are distributed over both positions). [b] Chemical shifts δ in ppm. Multiplicity: s = singlet, d = doublet, m = multiplet, br = broad. Coupling constants *J* are given in Hertz.

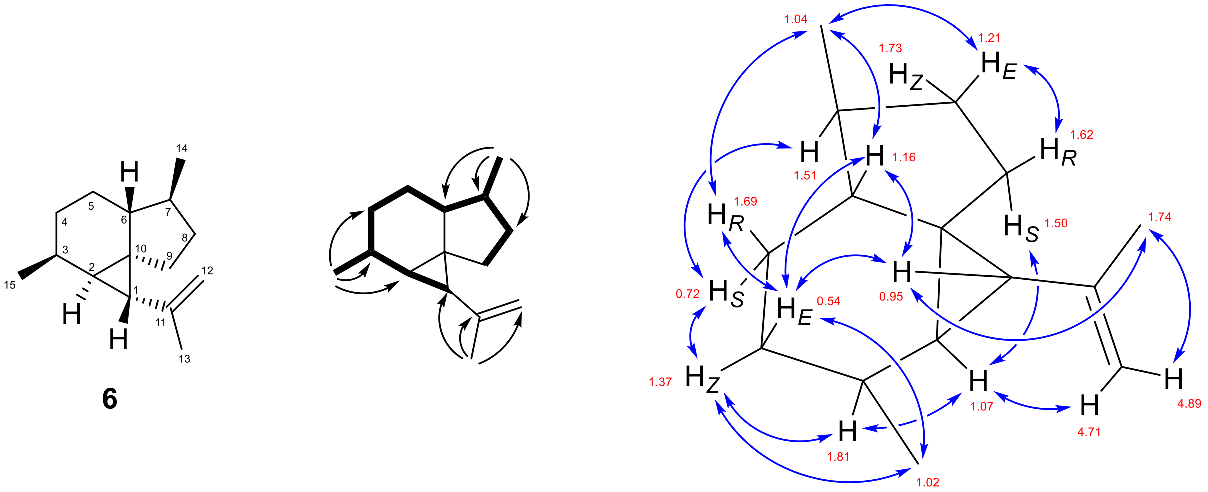

Figure S47. Structure elucidation of (–)-tamariscene (**6**). Bold: ^{1}H,^{1}H-COSY, single headed arrows: key HMBC, and blue double headed arrows: NOESY correlations. H_R, H_S, H_E and H_Z indicate the results from stereoselective labelling experiments (Figures S60 and S65).

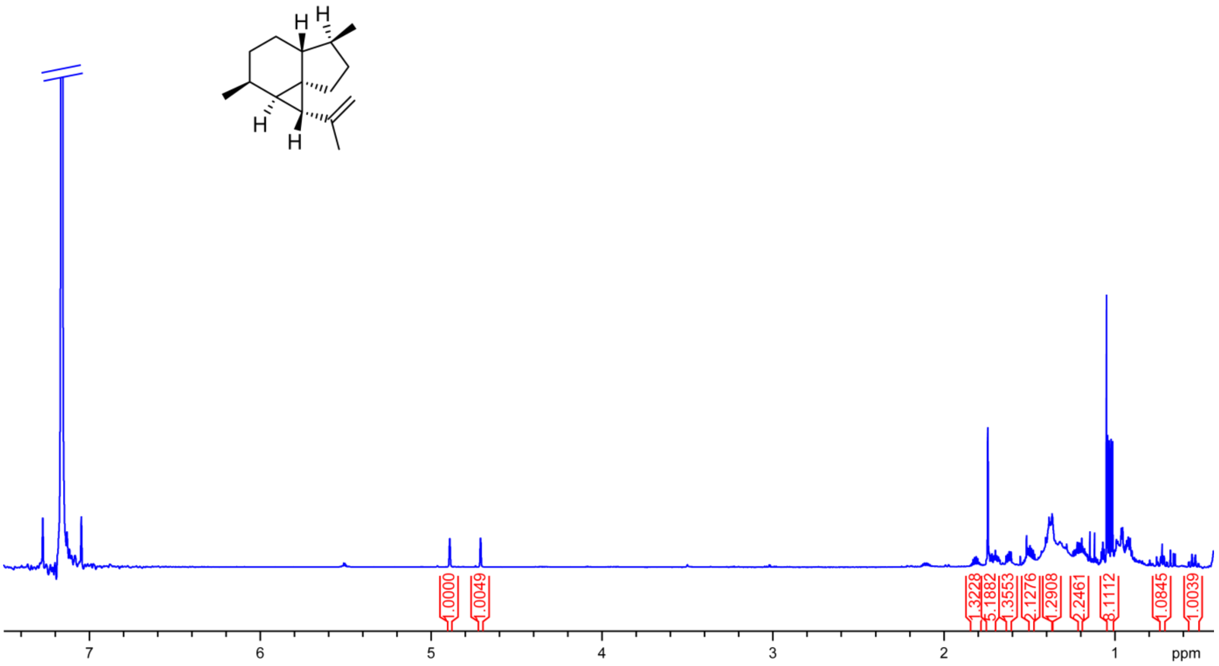

Figure S48. ^{1}H-NMR spectrum of **6** (700 MHz, C_6D_6).

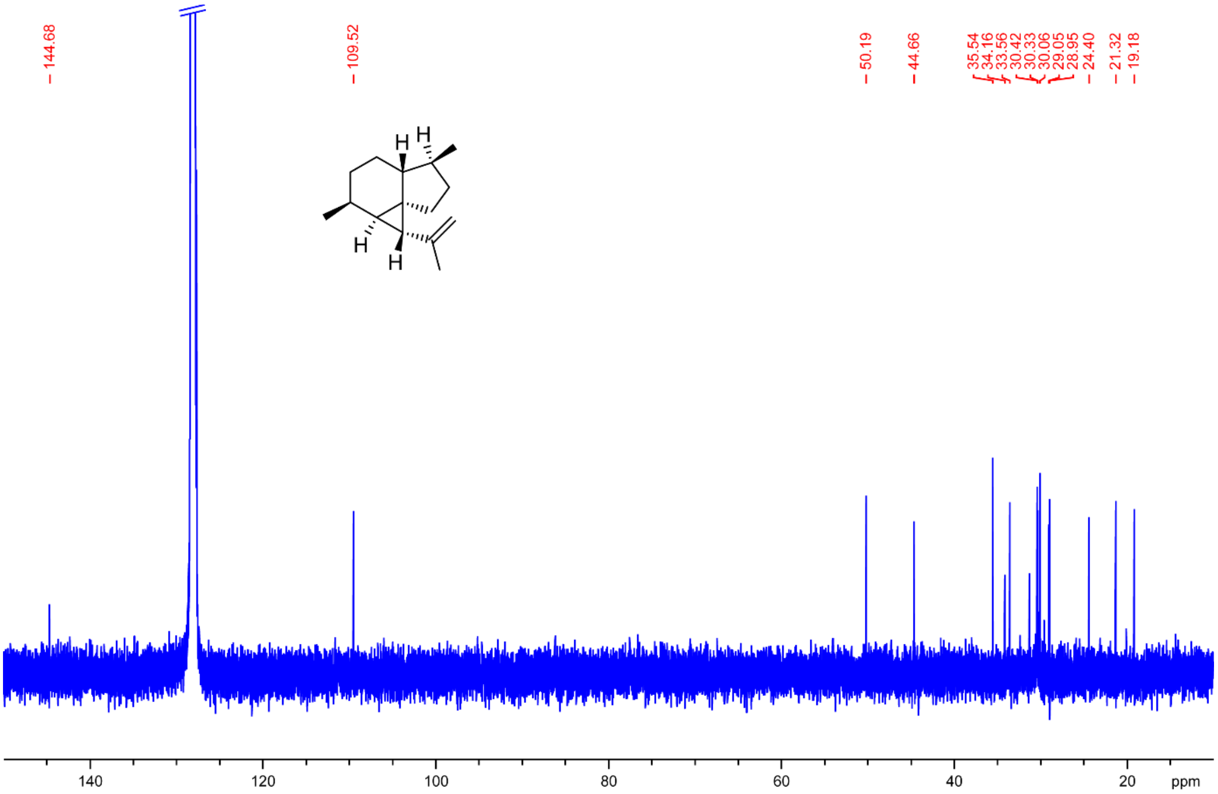

Figure S49. ^{13}C-NMR spectrum of **6** (176 MHz, C_6D_6).

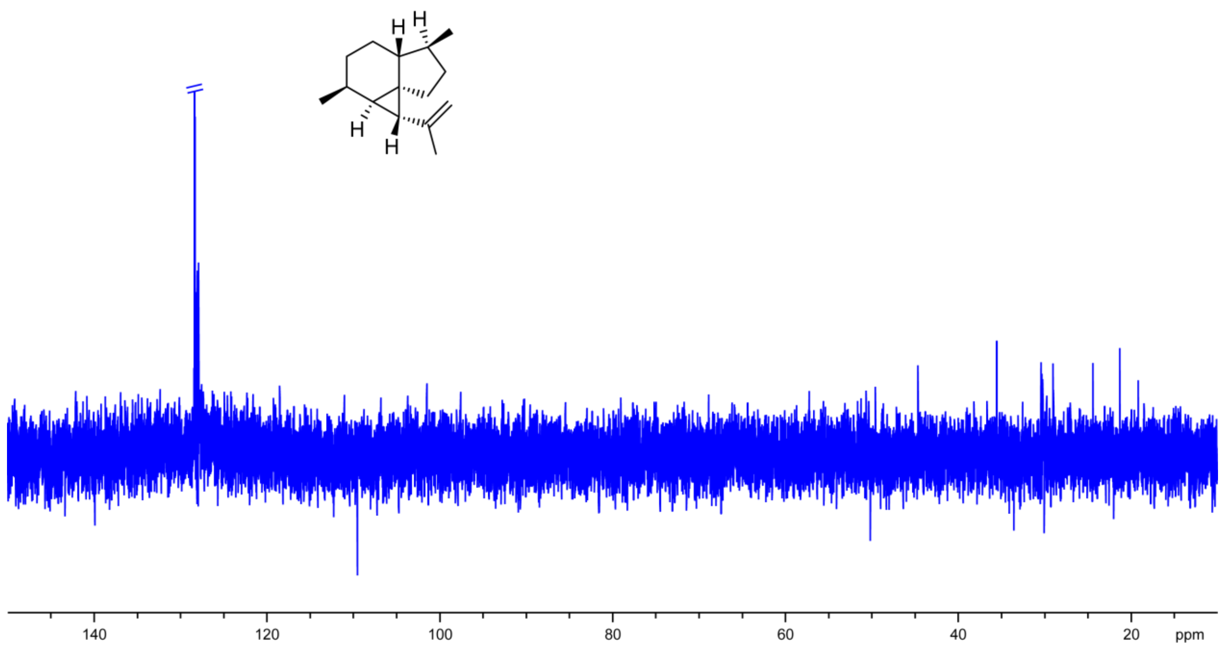

Figure S50. ^{13}C-DEPT135 spectrum of **6** (176 MHz, C_6D_6).

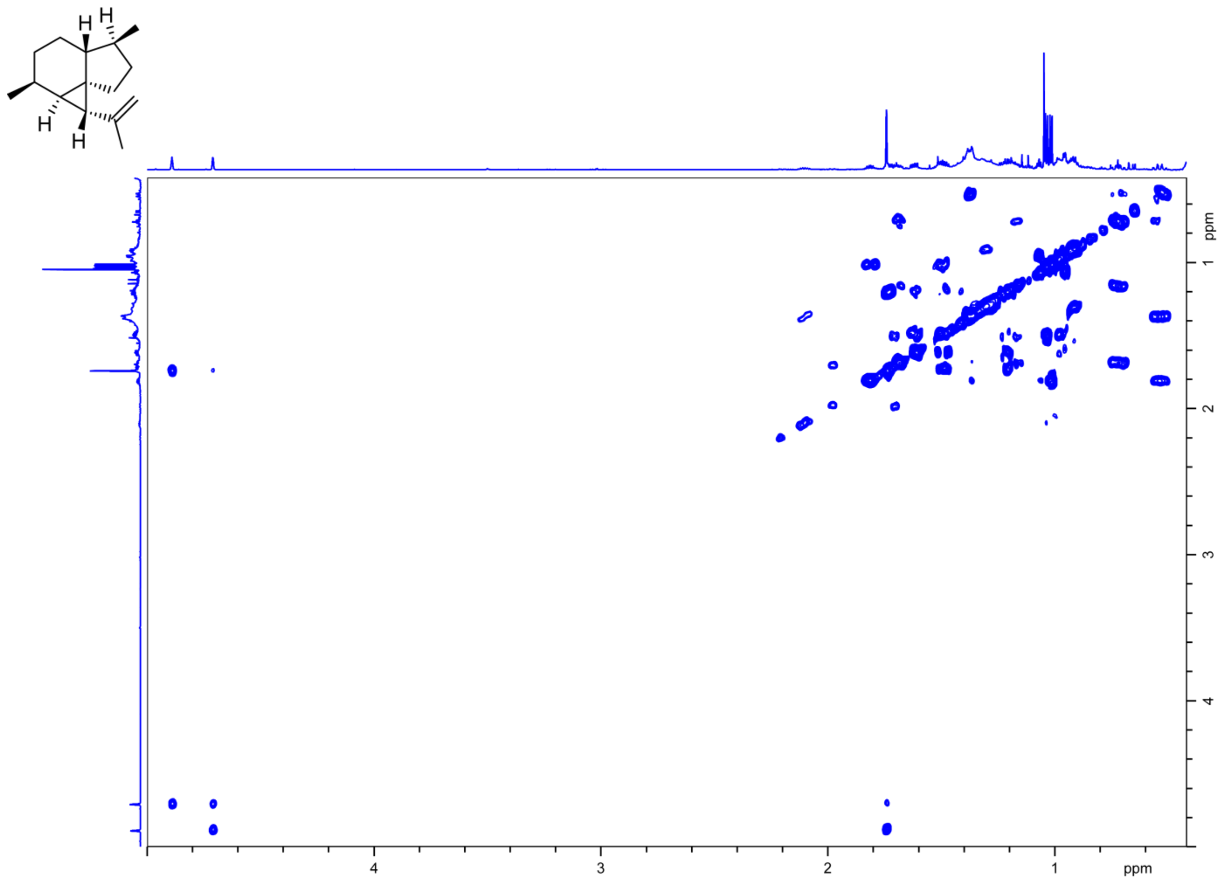

Figure S51. $^1H,^1H$-COSY spectrum (C_6D_6) of **6**.

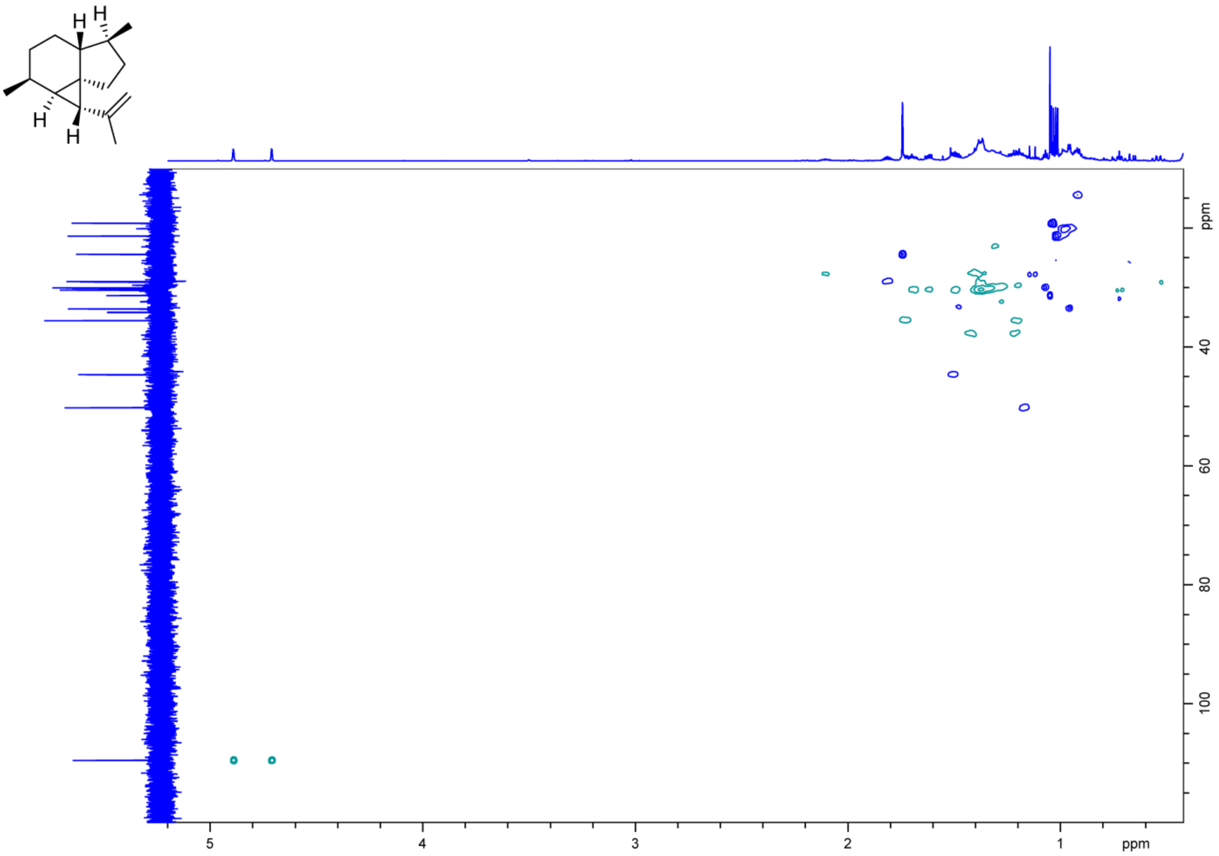

Figure S52. HSQC spectrum (C_6D_6) of **6**.

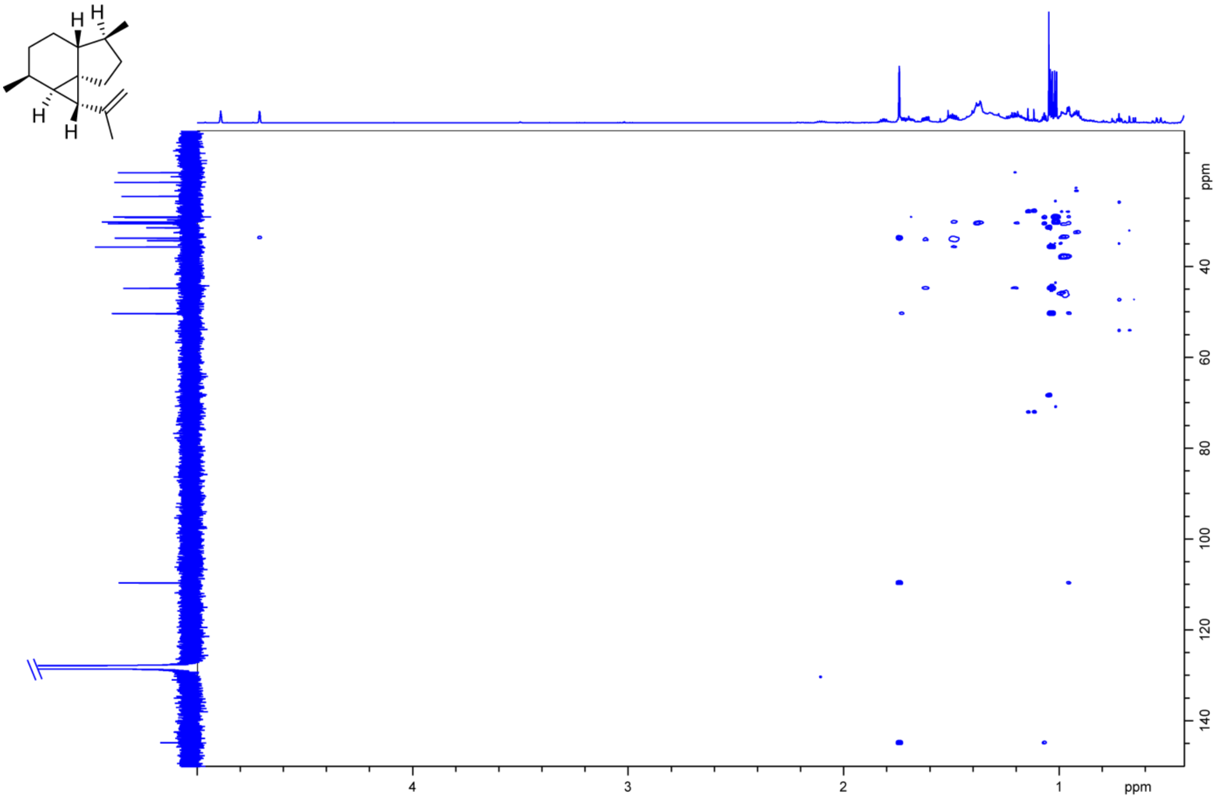

Figure S53. HMBC spectrum (C_6D_6) of **6**.

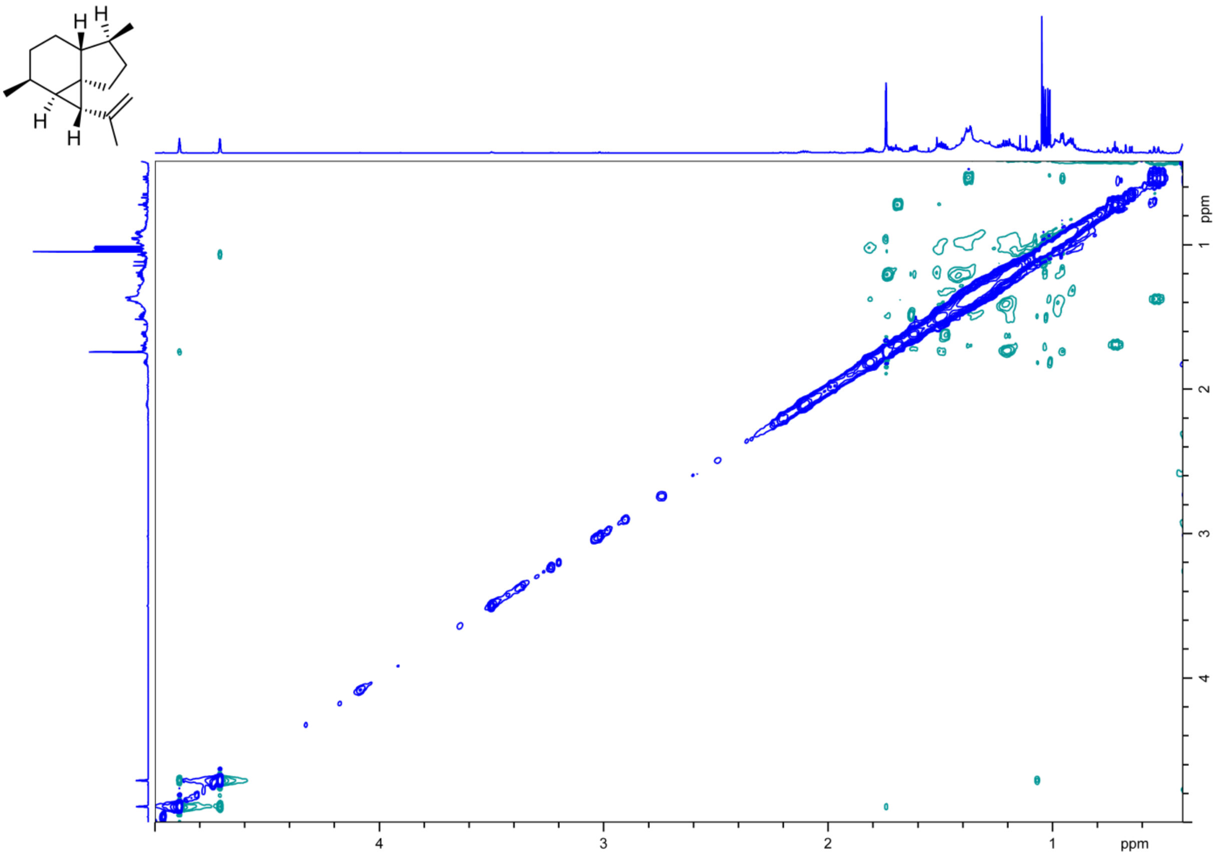

Figure S54. NOESY spectrum (C_6D_6) of **6**.

Incubation experiments with labelled substrates

Isotopic labelling experiments were performed with labelled FPP or its precursors (ca. 1.0 mg, in 100 µL 25 mM NH_4HCO_3), incubation buffer (850 µL) and preparations of purified enzymes (each 50 µL) as listed in Table S9. After incubation at 30 °C overnight, the products were extracted with C_6D_6 (600 µL) or n-hexane (200 µL), the extracts were dried with $MgSO_4$ and analysed by NMR and/or GC/MS.

Table S9. Labelling experiments with BgPgS.

entry	substrates	enzymes	results shown in
1	(*E*)-(4-^{13}C,4-^{2}H)IPP[18] + DMAPP	FPPS,[19] BgPgS	Figures S55 – S60
2	(*Z*)-(4-^{13}C,4-^{2}H)IPP[18] + DMAPP	FPPS, BgPgS	Figures S55 – S60
3	(*S*)-(1-^{13}C,1-^{2}H)IPP[20]	IDI,[21] FPPS, BgPgS	Figures S61 – S65 and S74
4	(*R*)-(1-^{13}C,1-^{2}H)IPP[20]	IDI, FPPS, BgPgS	Figures S61 – S65 and S75
5	(1-^{13}C)FPP[23]	BgPgS	Figures S66 – S71
6	(2-^{13}C)FPP[23]	BgPgS	Figures S66 – S71
7	(3-^{13}C)FPP[23]	BgPgS	Figures S66 – S71
8	(4-^{13}C)FPP[23]	BgPgS	Figures S66 – S71
9	(5-^{13}C)FPP[23]	BgPgS	Figures S66 – S71
10	(6-^{13}C)FPP[23]	BgPgS	Figures S66 – S71
11	(3-^{13}C)GPP[37] + IPP	FPPS, BgPgS	Figures S66 – S71
12	(8-^{13}C)FPP[23]	BgPgS	Figures S66 – S71
13	(1-^{13}C)DMAPP[38] + IPP	FPPS, BgPgS	Figures S66 – S71
14	(10-^{13}C)FPP[23]	BgPgS	Figures S66 – S71
15	(11-^{13}C)FPP[23]	BgPgS	Figures S66 – S71
16	(12-^{13}C)FPP[23]	BgPgS	Figures S66 – S71
17	(13-^{13}C)FPP[23]	BgPgS	Figures S66 – S71
18	(14-^{13}C)FPP[23]	BgPgS	Figures S66 – S71
19	(15-^{13}C)FPP[23]	BgPgS	Figures S66 – S71
20	(3-^{13}C)-FPP[23] + D_2O	BgPgS	Figures S72 – S73
21	(3-^{13}C,2-^{2}H)FPP[24]	BgPgS	Figures S76, S79, S82 and S90
22	(2-^{2}H)DMAPP[25] + (2-^{13}C)IPP[26]	FPPS, BgPgS	Figures S77 and S80
23	(2-^{2}H)GPP[27] + (2-^{13}C)IPP	FPPS, BgPgS	Figures S78 and S81
24	(2-^{2}H)GPP + IPP	FPPS, BgPgS	Figure S83
25	(3-^{13}C,2-^{2}H)FPP	BgPgS-G299A	Figures S84 and S87
26	(2-^{2}H)GPP + (2-^{13}C)IPP	FPPS, BgPgS-G299A	Figures S85 and S88
27	(2-^{2}H)DMAPP + (3-^{13}C)IPP[28]	FPPS, BgPgS-G299A	Figure S86
28	(2-^{2}H)DMAPP + (2-^{13}C)IPP	FPPS, BgPgS-G299A	Figure S89

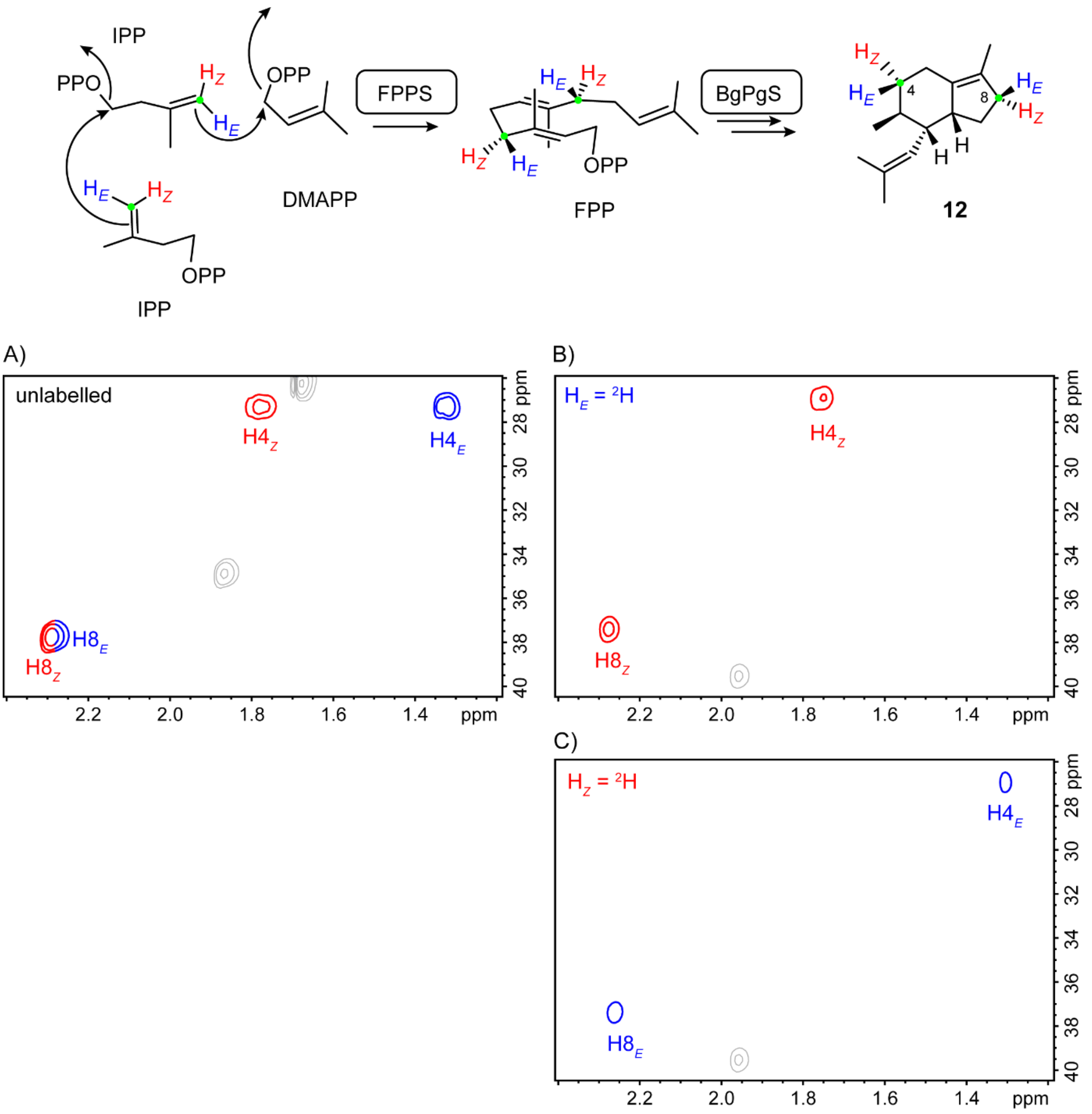

Figure S55. The absolute configuration of **12**. Partial HSQC spectra of A) unlabelled **12**, B) labelled **12** obtained from DMAPP and (*E*)-(4-^{13}C,4-^{2}H)IPP (blue H = ^{2}H) incubated with FPPS and BgPgS and C) labelled **12** obtained from DMAPP and (*Z*)-(4-^{13}C,4-^{2}H)IPP (red H = ^{2}H) incubated with FPPS and BgPgS. The specific incorporation at C4 and C8 with known configuration at these carbons in experiments B) and C) together with the NOESY based assignments of relative orientations of $H4_E$, $H4_Z$, $H8_E$, and $H8_Z$ (Figure S5) with respect to the naturally present stereogenic centers in **12** allows to assign the shown absolute configuration. Green dots represent ^{13}C-labelled carbons.

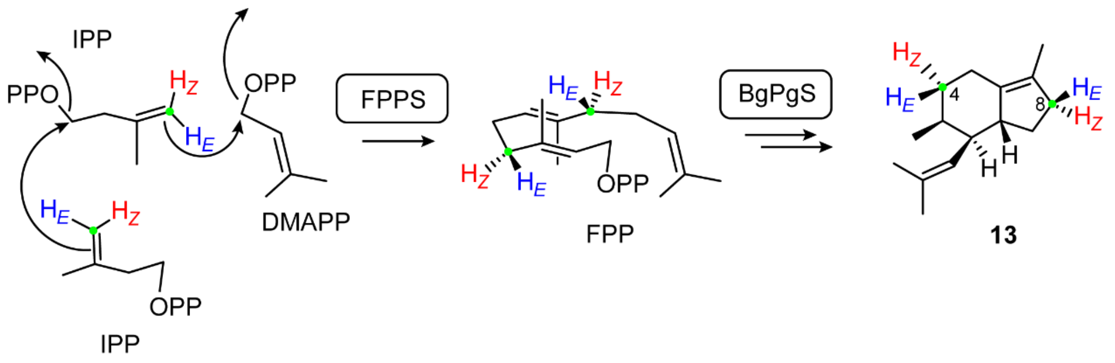

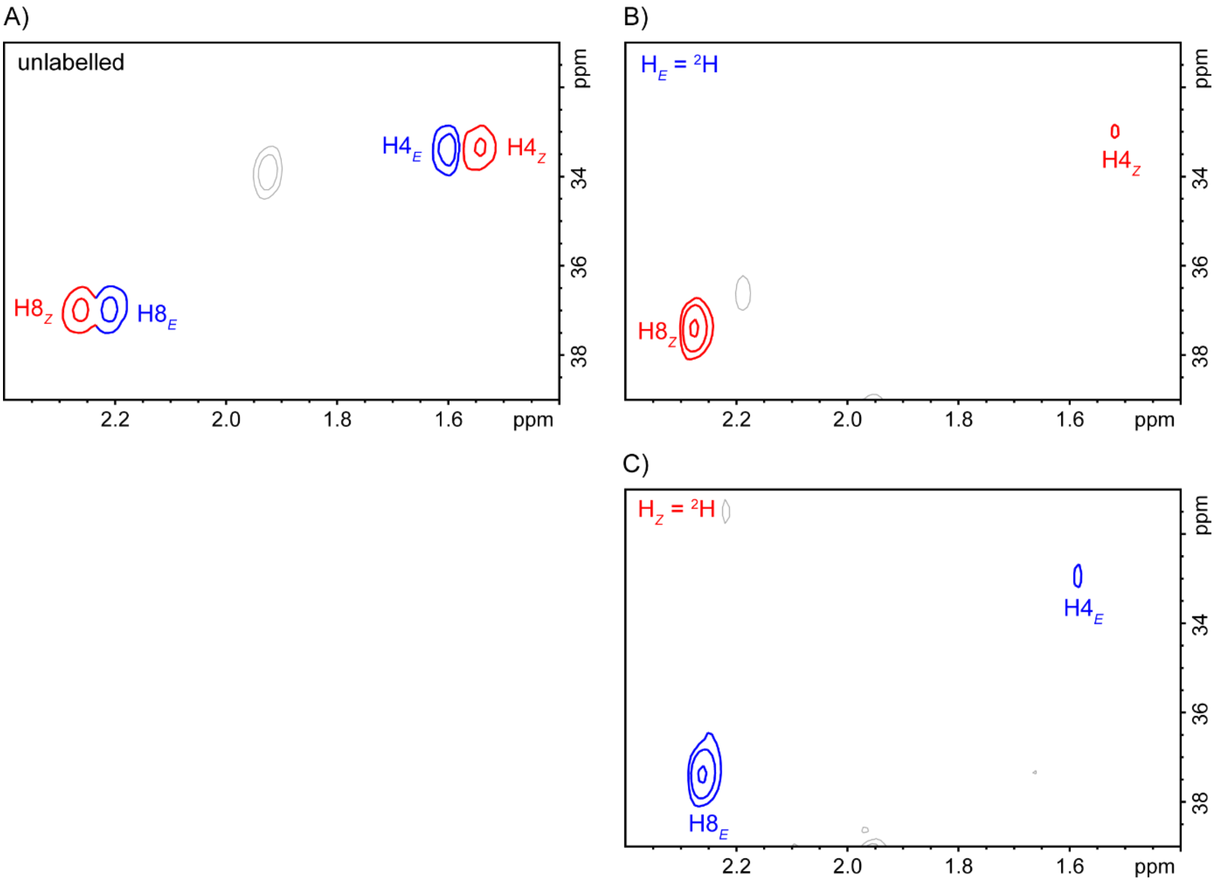

Figure S56. The absolute configuration of **13**. Partial HSQC spectra of A) unlabelled **13**, B) labelled **13** obtained from DMAPP and (*E*)-(4-^{13}C,4-^{2}H)IPP (blue H = ^{2}H) incubated with FPPS and BgPgS and C) labelled **13** obtained from DMAPP and (*Z*)-(4-^{13}C,4-^{2}H)IPP (red H = ^{2}H) incubated with FPPS and BgPgS. The specific incorporation at C4 and C8 with known configuration at these carbons in experiments B) and C) together with the NOESY based assignments of relative orientations of H4$_E$, H4$_Z$, H8$_E$, and H8$_Z$ (Figure S13) with respect to the naturally present stereogenic centers in **13** allows to assign the shown absolute configuration. Green dots represent ^{13}C-labelled carbons.

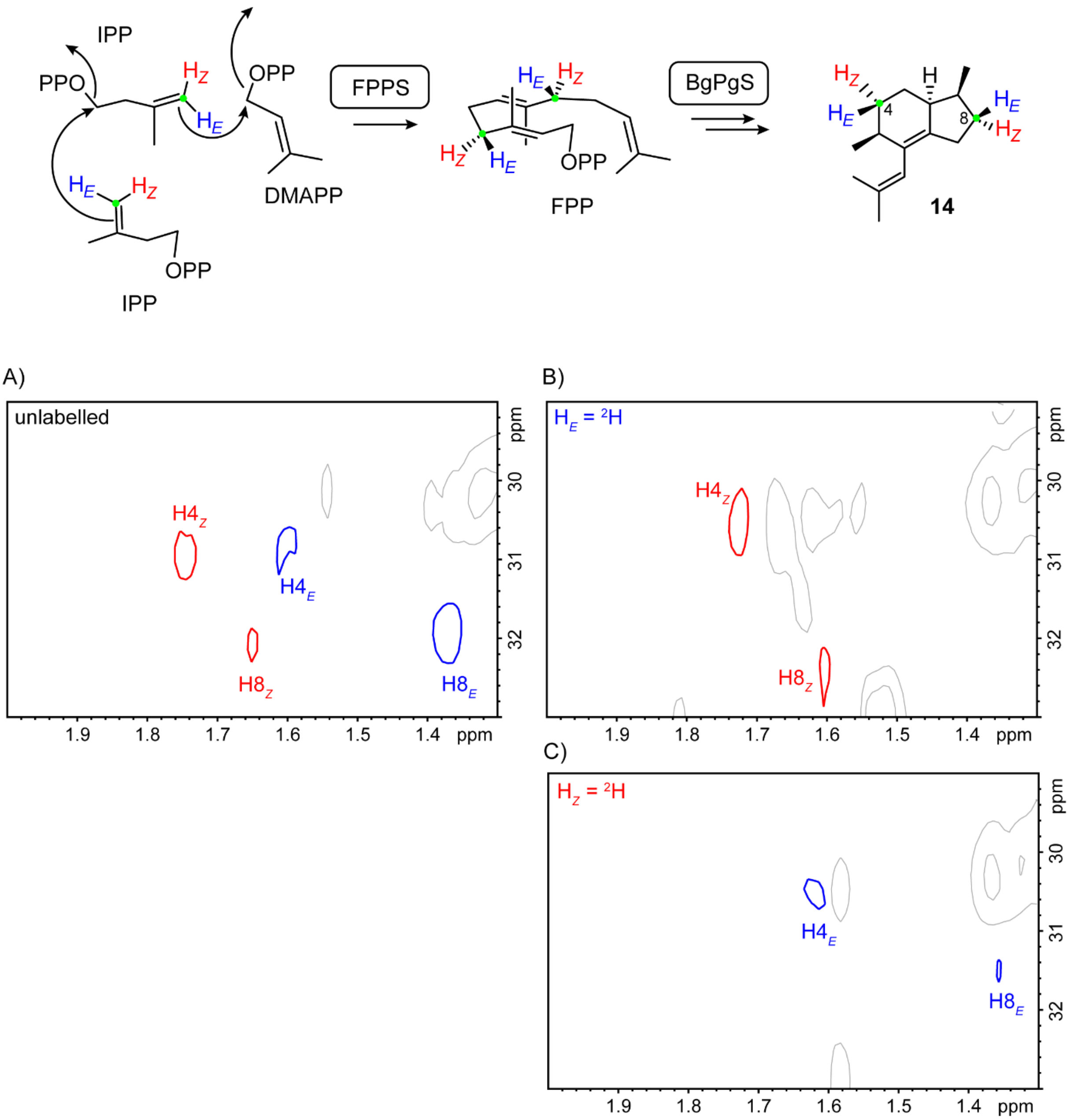

Figure S57. The absolute configuration of **14**. Partial HSQC spectra of A) unlabelled **14**, B) labelled **14** obtained from DMAPP and (*E*)-(4-^{13}C,4-^{2}H)IPP (blue H = ^{2}H) incubated with FPPS and BgPgS and C) labelled **14** obtained from DMAPP and (*Z*)-(4-^{13}C,4-^{2}H)IPP (red H = ^{2}H) incubated with FPPS and BgPgS. The specific incorporation at C4 and C8 with known configuration at these carbons in experiments B) and C) together with the NOESY based assignments of relative orientations of H4$_E$, H4$_Z$, H8$_E$, and H8$_Z$ (Figure S21) with respect to the naturally present stereogenic centers in **14** allows to assign the shown absolute configuration. Green dots represent ^{13}C-labelled carbons.

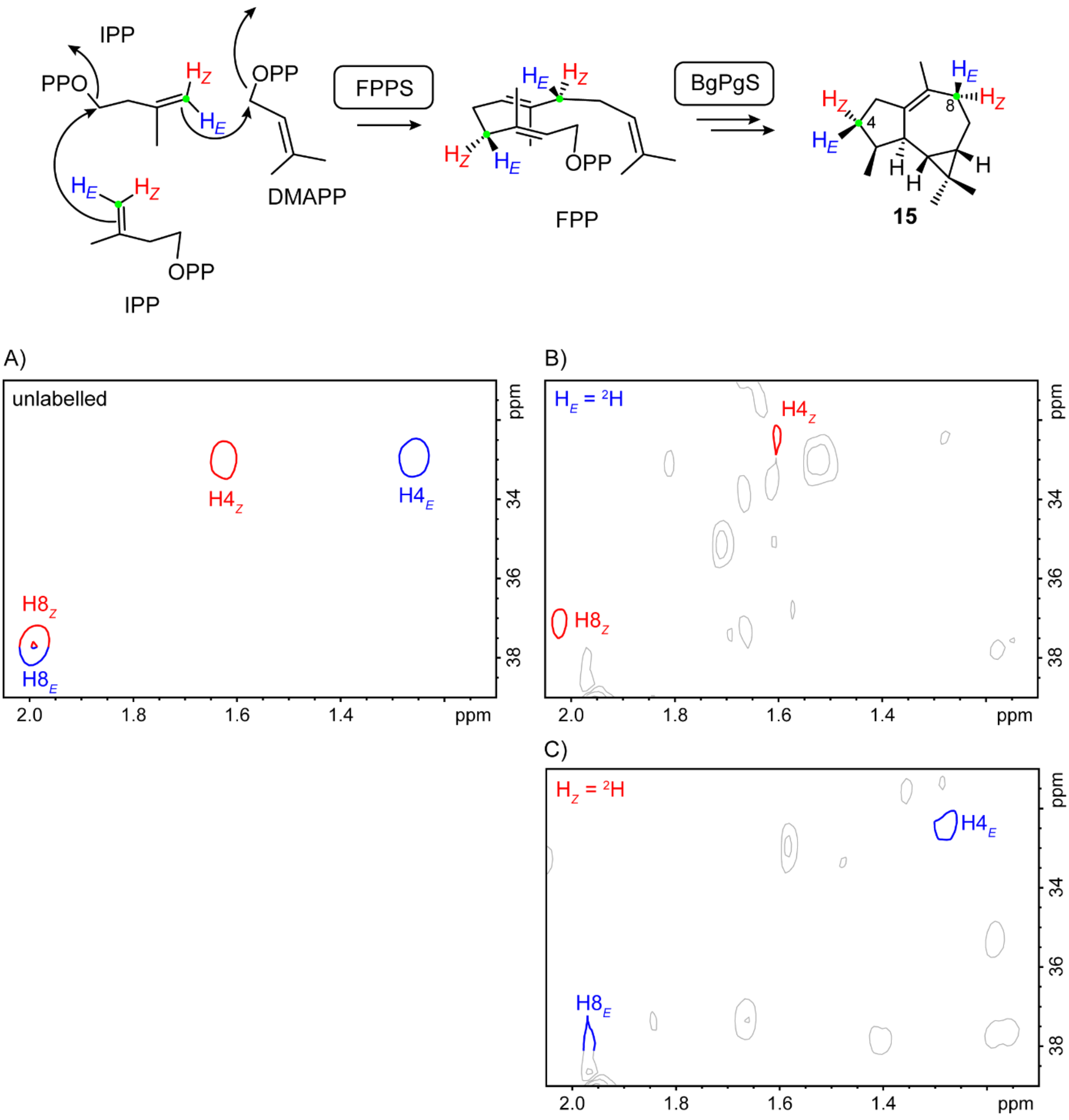

Figure S58. The absolute configuration of **15**. Partial HSQC spectra of A) unlabelled **15**, B) labelled **15** obtained from DMAPP and (*E*)-(4-^{13}C,4-^{2}H)IPP (blue H = ^{2}H) incubated with FPPS and BgPgS and C) labelled **15** obtained from DMAPP and (*Z*)-(4-^{13}C,4-^{2}H)IPP (red H = ^{2}H) incubated with FPPS and BgPgS. The specific incorporation at C4 and C8 with known configuration at these carbons in experiments B) and C) together with the NOESY based assignments of relative orientations of H4$_E$, H4$_Z$, H8$_E$, and H8$_Z$ (Figure S29) with respect to the naturally present stereogenic centers in **15** allows to assign the shown absolute configuration. Green dots represent ^{13}C-labelled carbons.

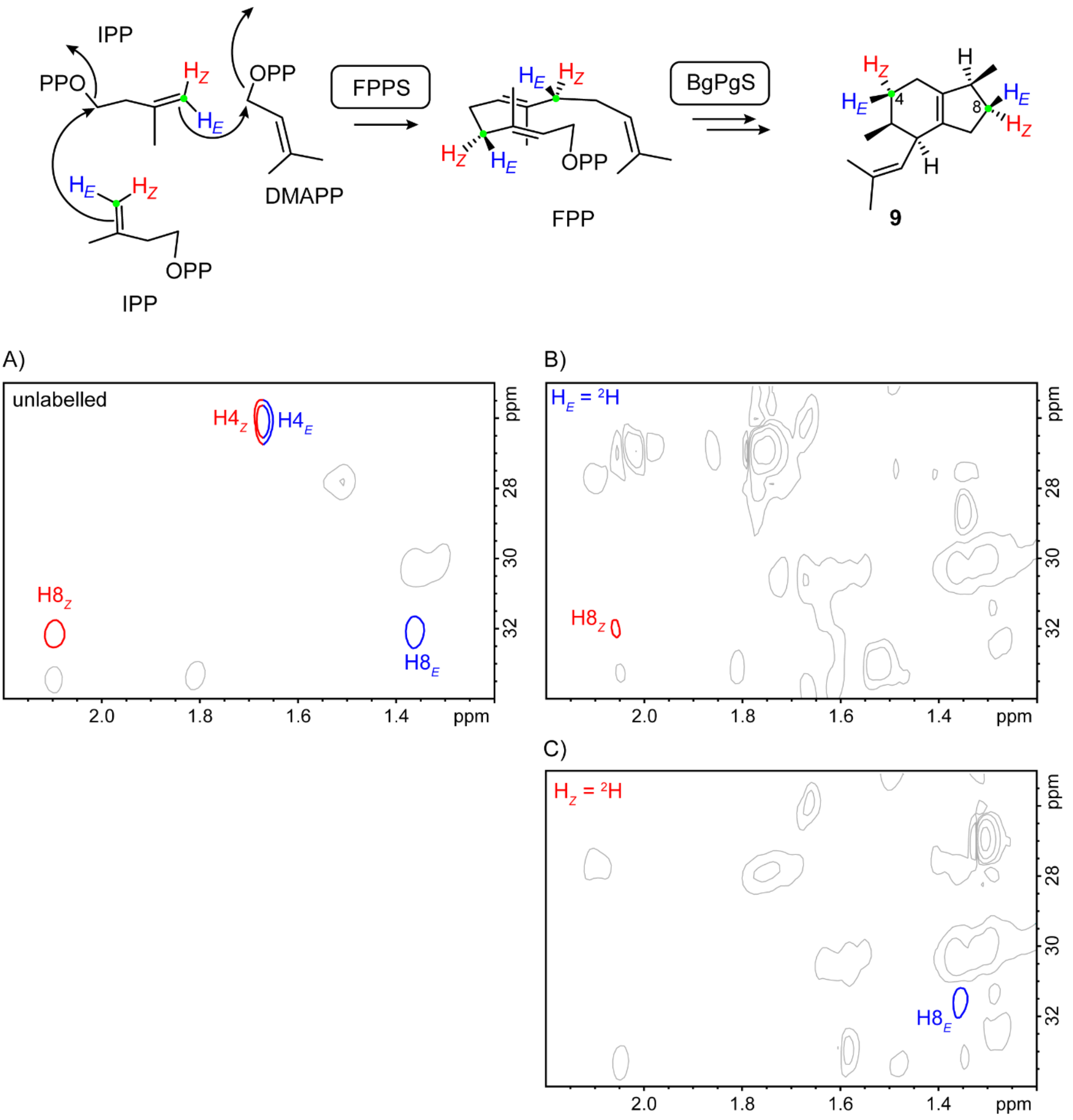

Figure S59. The absolute configuration of **9**. Partial HSQC spectra of A) unlabelled **9**, B) labelled **9** obtained from DMAPP and (*E*)-(4-^{13}C,4-^{2}H)IPP (blue H = ^{2}H) incubated with FPPS and BgPgS and C) labelled **9** obtained from DMAPP and (*Z*)-(4-^{13}C,4-^{2}H)IPP (red H = ^{2}H) incubated with FPPS and BgPgS. The specific incorporation at C4 and C8 with known configuration at these carbons in experiments B) and C) together with the NOESY based assignments of relative orientations of H8$_E$, and H8$_Z$ (Figure S39) with respect to the naturally present stereogenic centers in **9** allows to assign the shown absolute configuration. Green dots represent ^{13}C-labelled carbons.

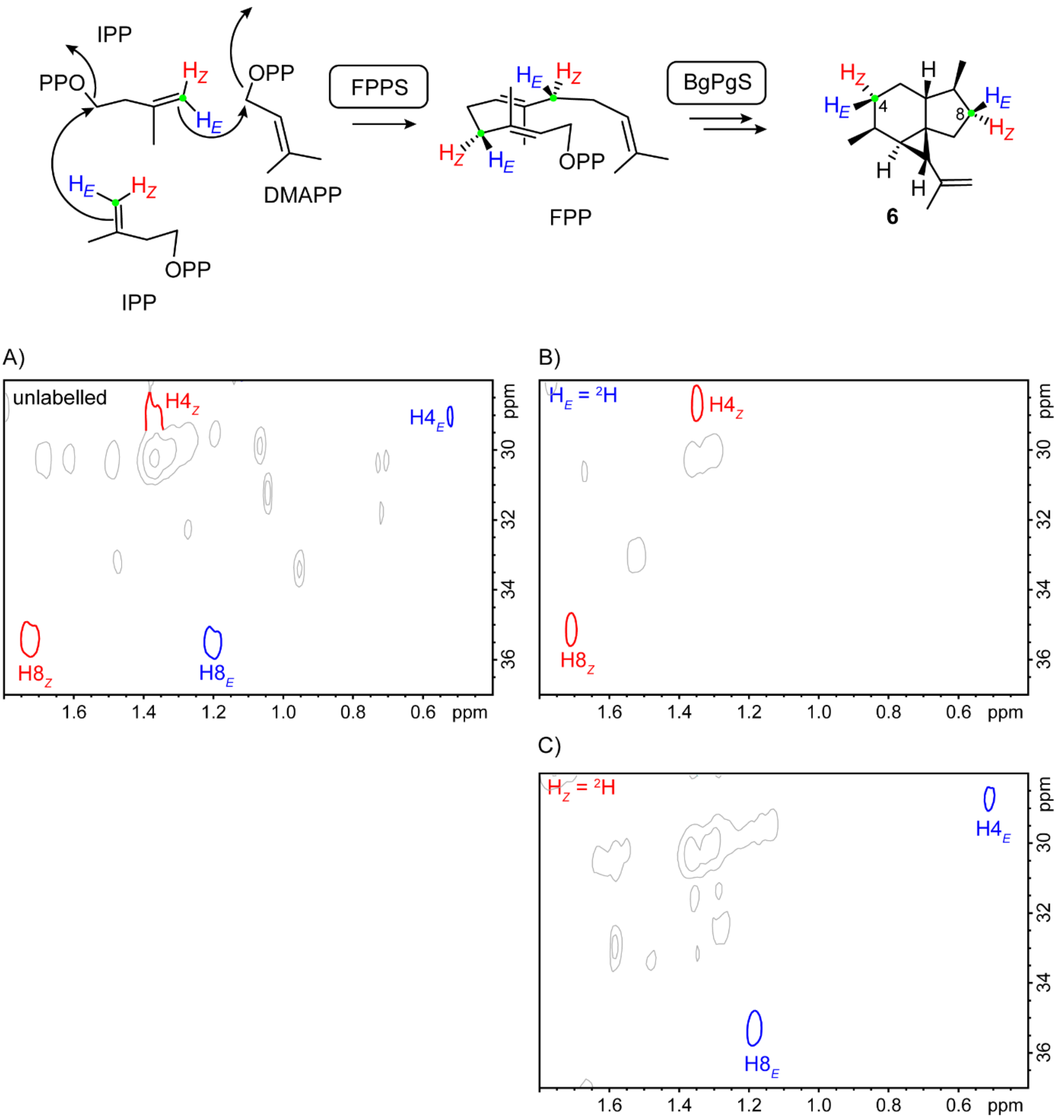

Figure S60. The absolute configuration of **6**. Partial HSQC spectra of A) unlabelled **6**, B) labelled **6** obtained from DMAPP and (*E*)-(4-^{13}C,4-^{2}H)IPP (blue H = ^{2}H) incubated with FPPS and BgPgS and C) labelled **6** obtained from DMAPP and (*Z*)-(4-^{13}C,4-^{2}H)IPP (red H = ^{2}H) incubated with FPPS and BgPgS. The specific incorporation at C4 and C8 with known configuration at these carbons in experiments B) and C) together with the NOESY based assignments of relative orientations of H4$_E$, H4$_Z$, H8$_E$, and H8$_Z$ (Figure S47) with respect to the naturally present stereogenic centers in **6** allows to assign the shown absolute configuration. Green dots represent ^{13}C-labelled carbons.

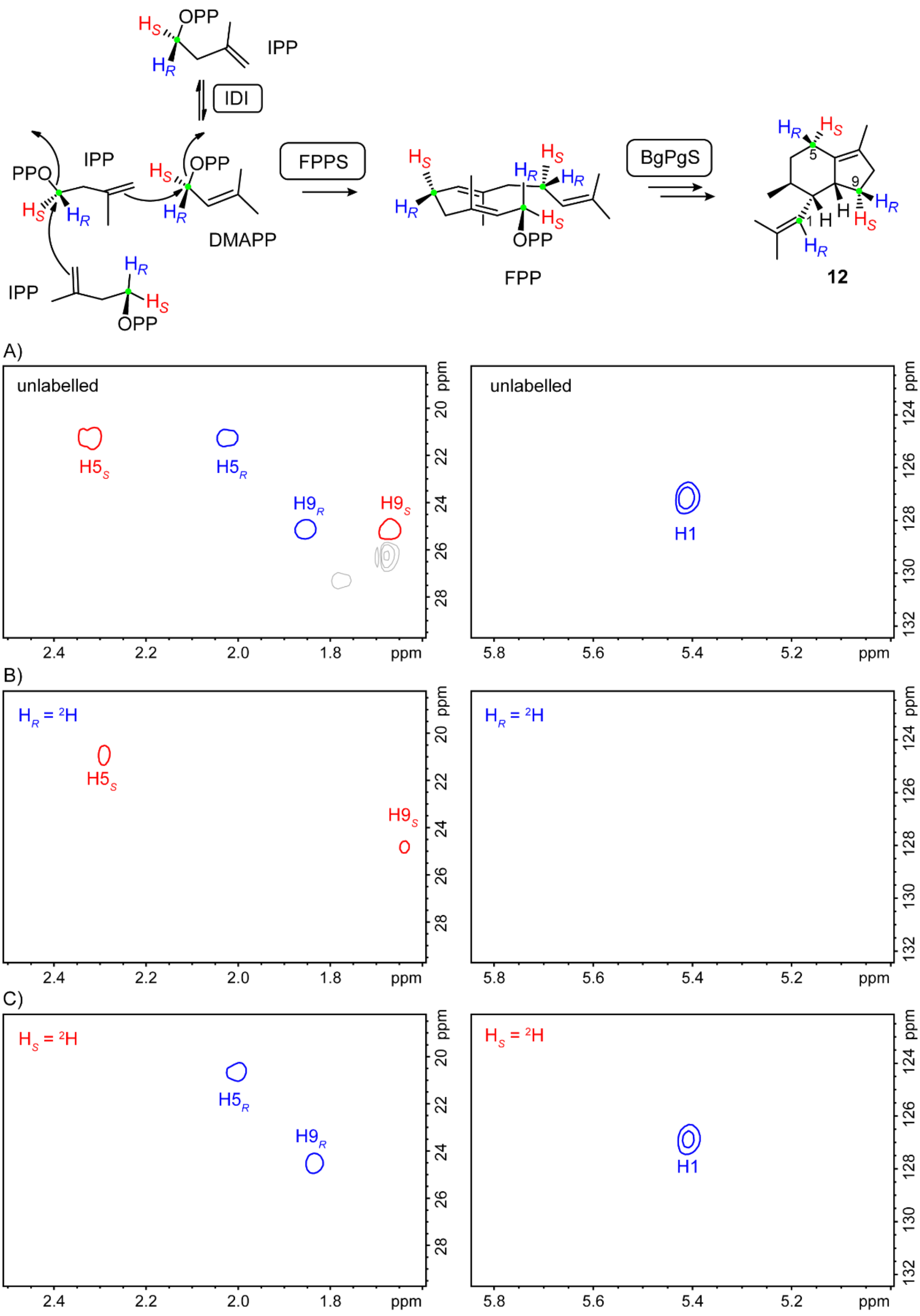

Figure S61. The absolute configuration of **12**. Partial HSQC spectra of A) unlabelled **12**, B) labelled **12** obtained from (*R*)-(1-^{13}C,1-^{2}H)IPP (blue H = ^{2}H) incubated with IDI, FPPS and BgPgS and C) labelled **1** obtained from (*S*)-(1-^{13}C,1-^{2}H)IPP (red H = ^{2}H) incubated with IDI, FPPS and BgPgS. The specific incorporation at C1, C5 and C9 with known configuration at these carbons in experiments B) and C) together with the NOESY based assignments of relative orientations of H5$_R$, H5$_S$, H9$_R$ and H9$_S$ (Figure S5) with respect to the naturally present stereogenic centers in **12** allows to assign the shown absolute configuration. Green dots represent ^{13}C-labelled carbons.

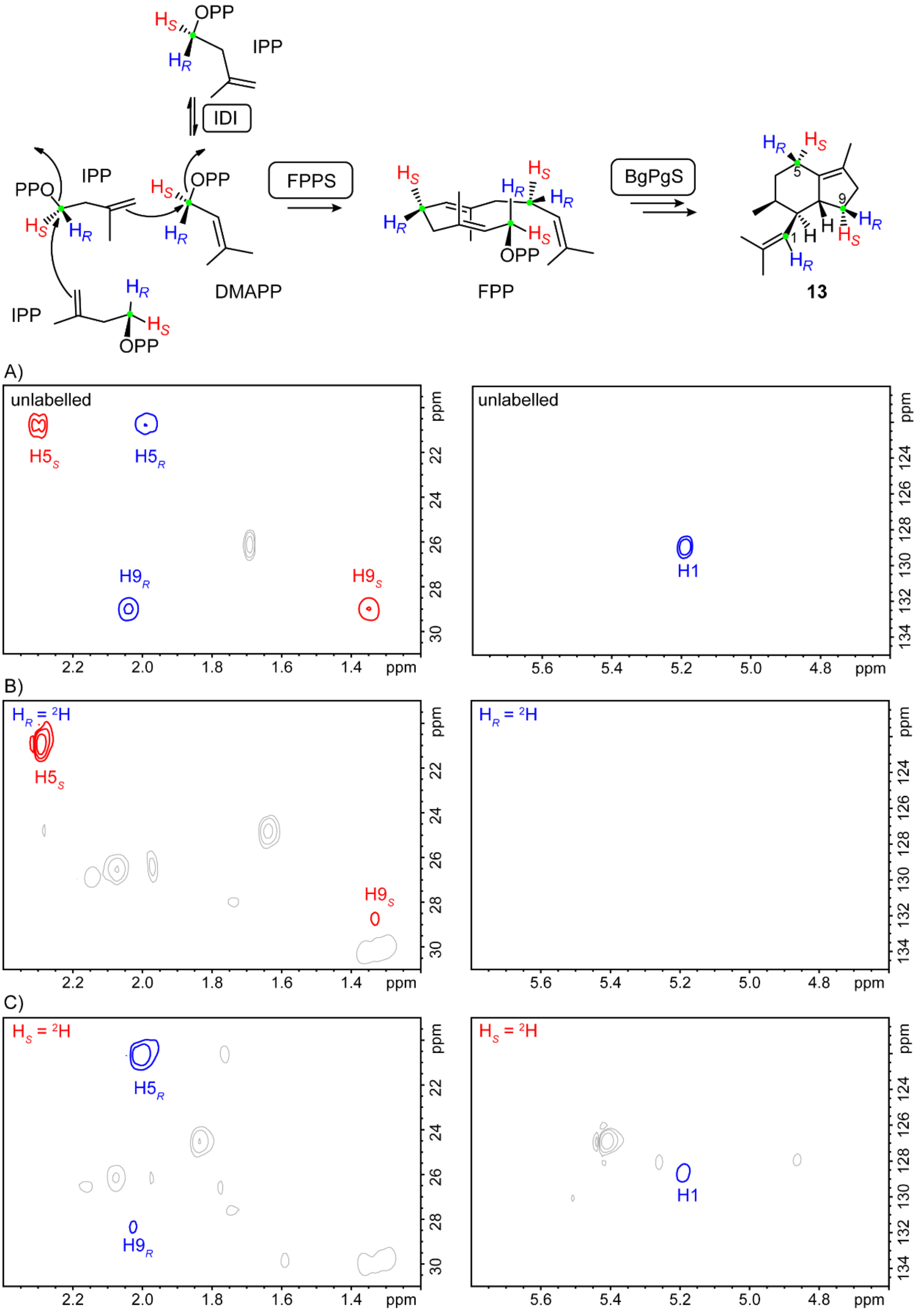

Figure S62. The absolute configuration of **13**. Partial HSQC spectra of A) unlabelled **13**, B) labelled **13** obtained from (*R*)-(1-^{13}C,1-^{2}H)IPP (blue H = ^{2}H) incubated with IDI, FPPS and BgPgS and C) labelled **13** obtained from (*S*)-(1-^{13}C,1-^{2}H)IPP (red H = ^{2}H) incubated with IDI, FPPS and BgPgS. The specific incorporation at C1, C5 and C9 with known configuration at these carbons in experiments B) and C) together with the NOESY based assignments of relative orientations of H5$_R$, H5$_S$, H9$_R$ and H9$_S$ (Figure S13) with respect to the naturally present stereogenic centers in **13** allows to assign the shown absolute configuration. Green dots represent ^{13}C-labelled carbons.

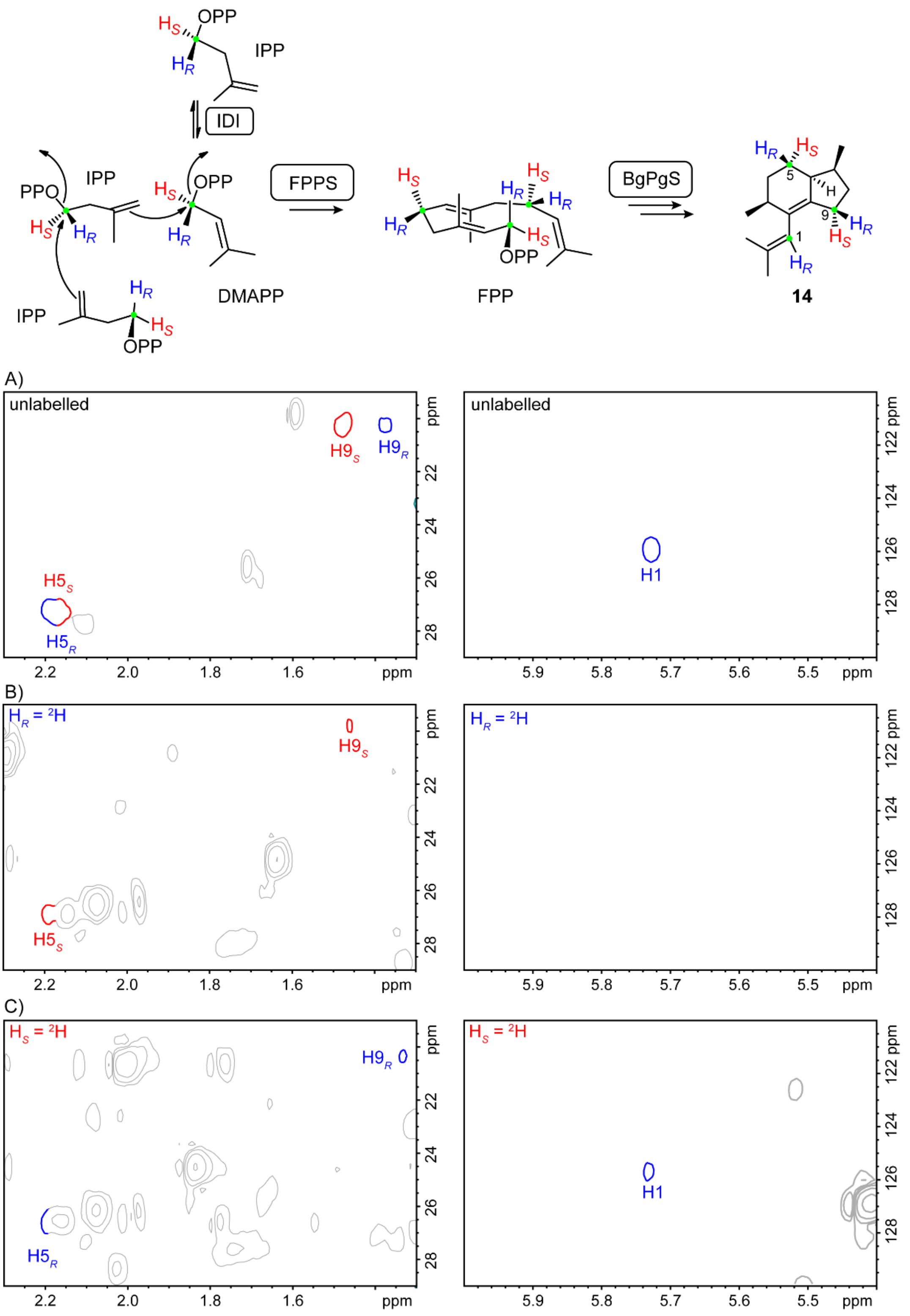

Figure S63. The absolute configuration of **14**. Partial HSQC spectra of A) unlabelled **14**, B) labelled **14** obtained from (*R*)-(1-^{13}C,1-^{2}H)IPP (blue H = ^{2}H) incubated with IDI, FPPS and BgPgS and C) labelled **14** obtained from (*S*)-(1-^{13}C,1-^{2}H)IPP (red H = ^{2}H) incubated with IDI, FPPS and BgPgS. The specific incorporation at C1, C5 and C9 with known configuration at these carbons in experiments B) and C) together with the NOESY based assignments of relative orientations of $H5_R$, $H5_S$, $H9_R$ and $H9_S$ (Figure S21) with respect to the naturally present stereogenic centers in **14** allows to assign the shown absolute configuration. Green dots represent ^{13}C-labelled carbons.

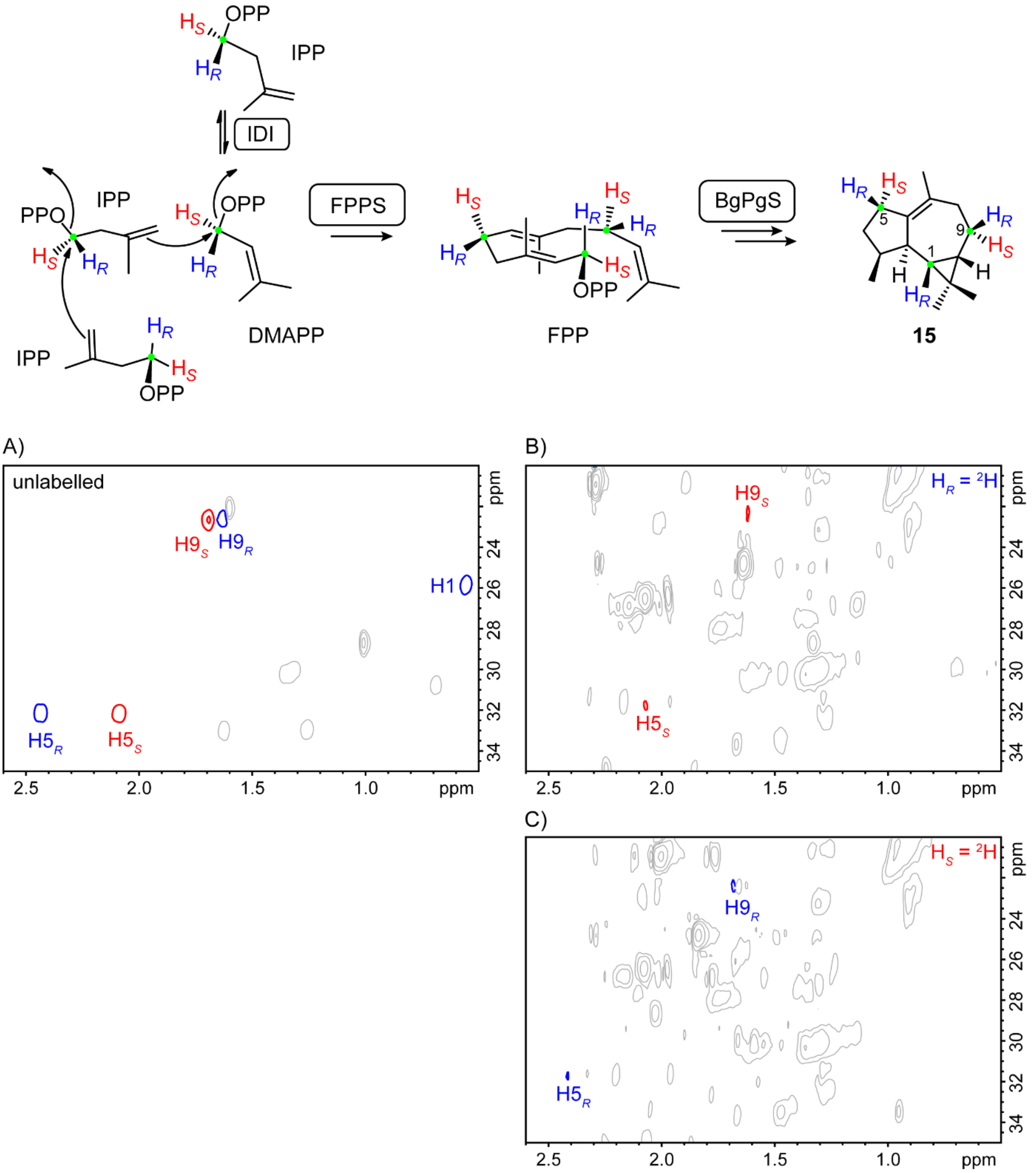

Figure S64. The absolute configuration of **15**. Partial HSQC spectra of A) unlabelled **15**, B) labelled **15** obtained from (*R*)-(1-^{13}C,1-^{2}H)IPP (blue H = ^{2}H) incubated with IDI, FPPS and BgPgS and C) labelled **15** obtained from (*S*)-(1-^{13}C,1-^{2}H)IPP (red H = ^{2}H) incubated with IDI, FPPS and BgPgS. The specific incorporation at C1, C5 and C9 with known configuration at these carbons in experiments B) and C) together with the NOESY based assignments of relative orientations of H5$_R$, H5$_S$, H9$_R$ and H9$_S$ (Figure S29) with respect to the naturally present stereogenic centers in **15** allows to assign the shown absolute configuration. Green dots represent ^{13}C-labelled carbons.

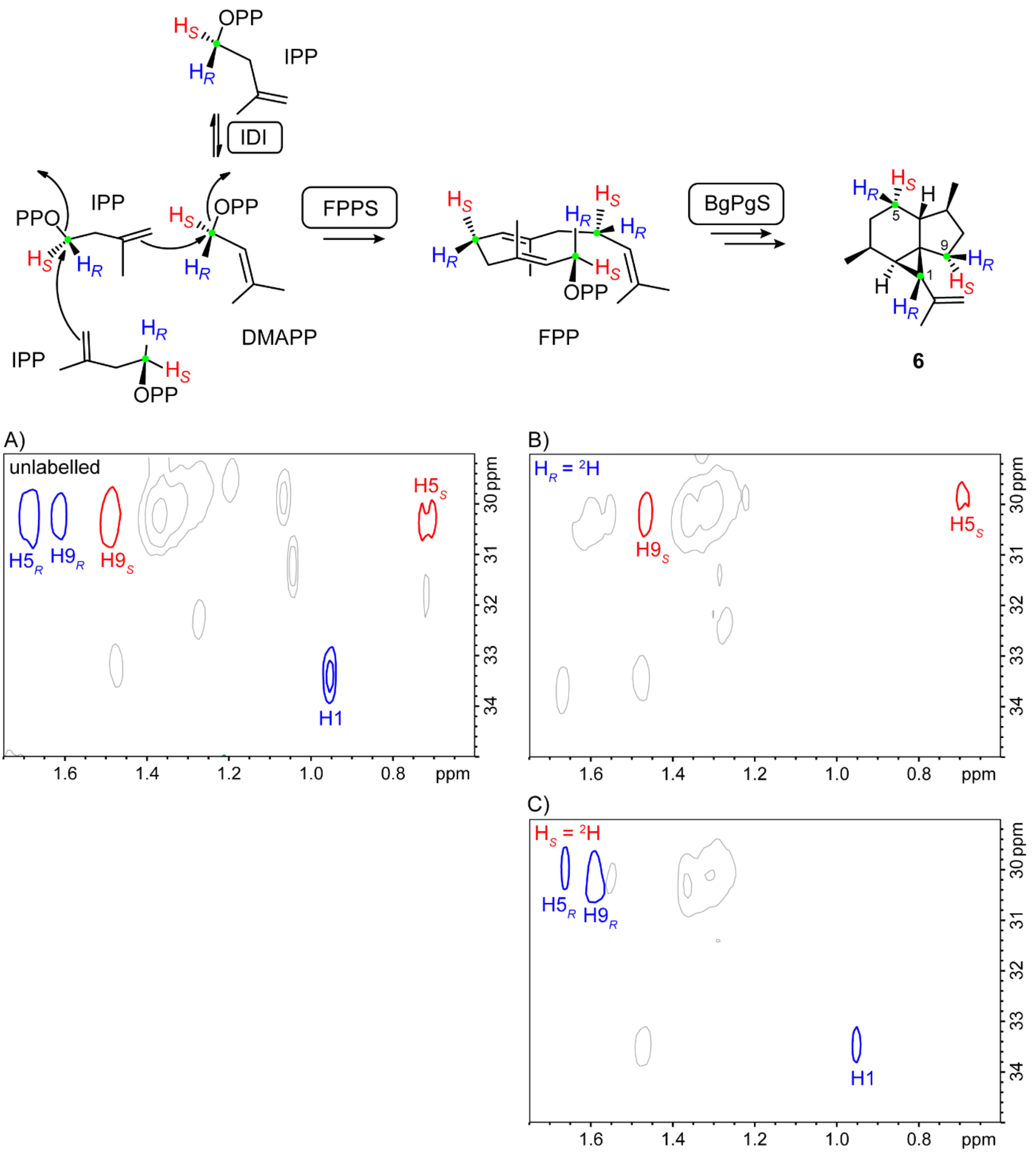

Figure S65. The absolute configuration of **6**. Partial HSQC spectra of A) unlabelled **6**, B) labelled **6** obtained from (*R*)-(1-^{13}C,1-^{2}H)IPP (blue H = ^{2}H) incubated with IDI, FPPS and BgPgS and C) labelled **6** obtained from (*S*)-(1-^{13}C,1-^{2}H)IPP (red H = ^{2}H) incubated with IDI, FPPS and BgPgS. The specific incorporation at C1, C5 and C9 with known configuration at these carbons in experiments B) and C) together with the NOESY based assignments of relative orientations of H5$_R$, H5$_S$, H9$_R$ and H9$_S$ (Figure S47) with respect to the naturally present stereogenic centers in **6** allows to assign the shown absolute configuration. Green dots represent ^{13}C-labelled carbons.

Single ^{13}C labellings

Approach: All compounds included in the investigation are first isolated from an in vitro reaction of the substrate (here: FPP) with purified enzyme and their structures are rigorously determined by NMR spectroscopy. The labelling experiments then make use of the whole series of singly ^{13}C-labelled substrates, from (1-^{13}C)FPP to (15-^{13}C)FPP in 15 individual reactions. These reactions are carried out in small scale (1 mg of labelled substrate). Each enzyme product will show up by a specific signal for its labelled carbon, with the signal intensity reflecting the amount formed.

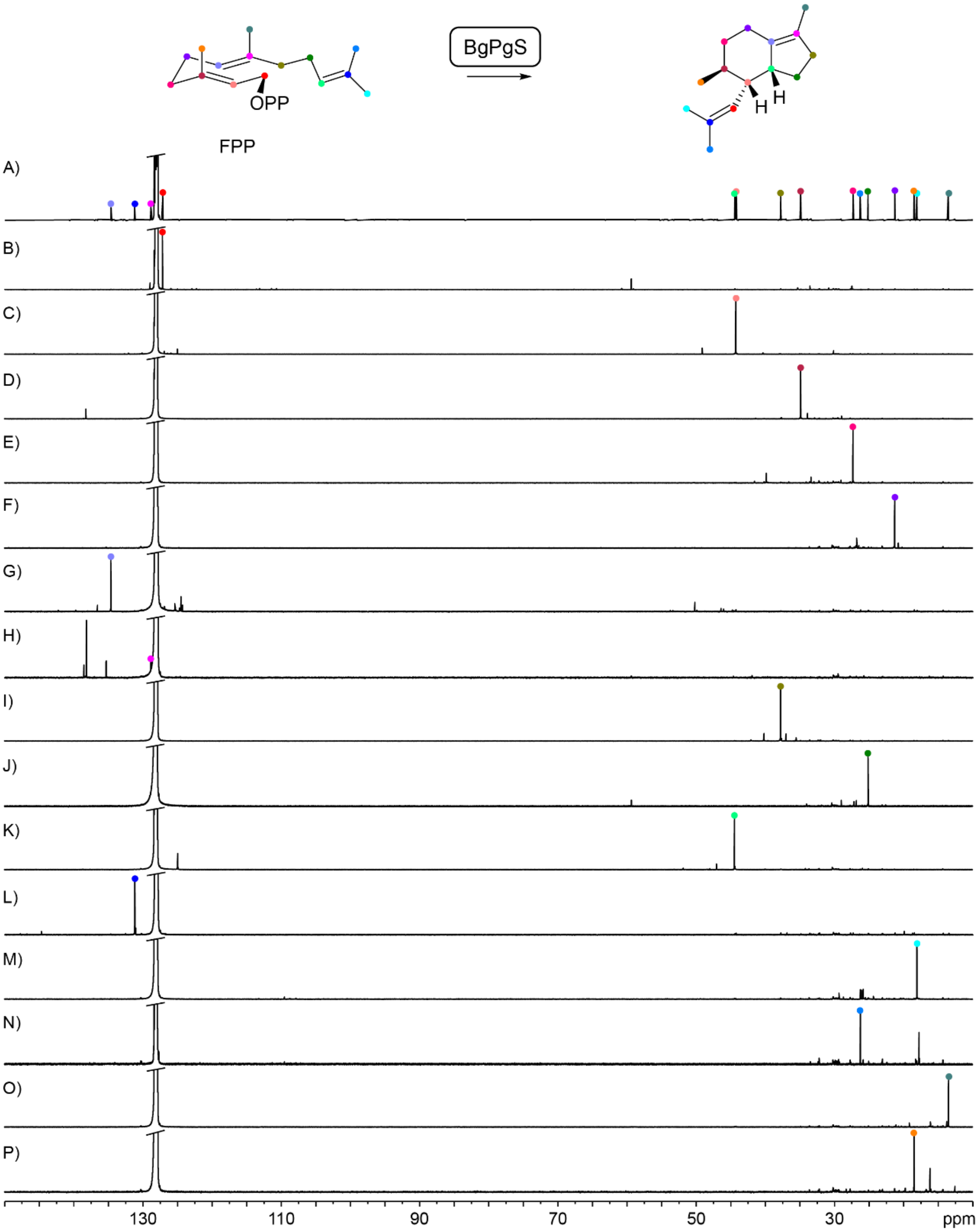

Figure S66. ^{13}C-NMR spectra of A) unlabelled **12**, and B) – P) the mixture of products obtained with BgPgS from (1-^{13}C)FPP – (15-^{13}C)FPP. The coloured dots show the site of incorporation into **12** and indicate the corresponding signal in the ^{13}C-NMR spectra.

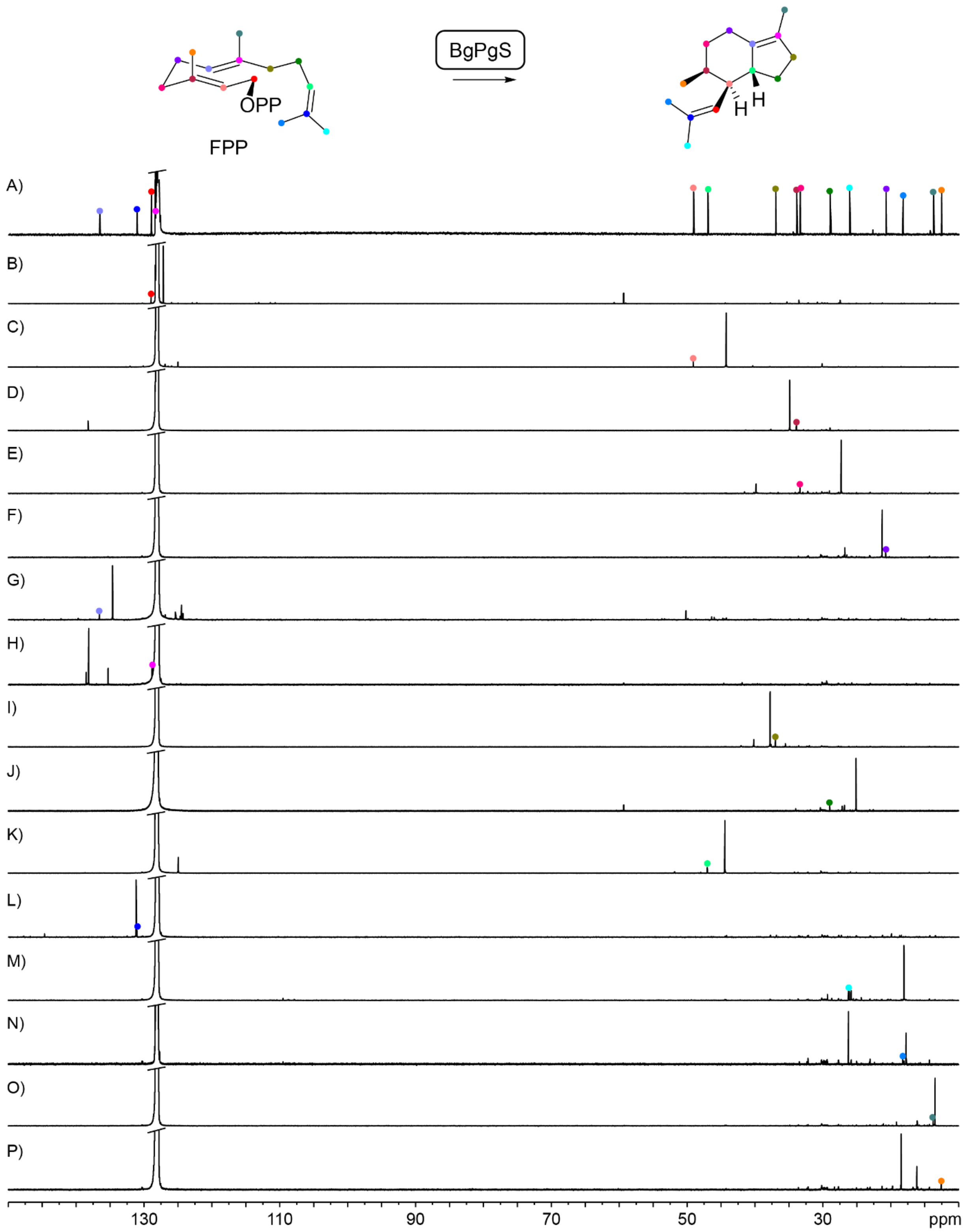

Figure S67. ^{13}C-NMR spectra of A) unlabelled **13**, and B) – P) the mixture of products obtained with BgPgS from (1-^{13}C)FPP – (15-^{13}C)FPP. The coloured dots show the site of incorporation into **13** and indicate the corresponding signal in the ^{13}C-NMR spectra.

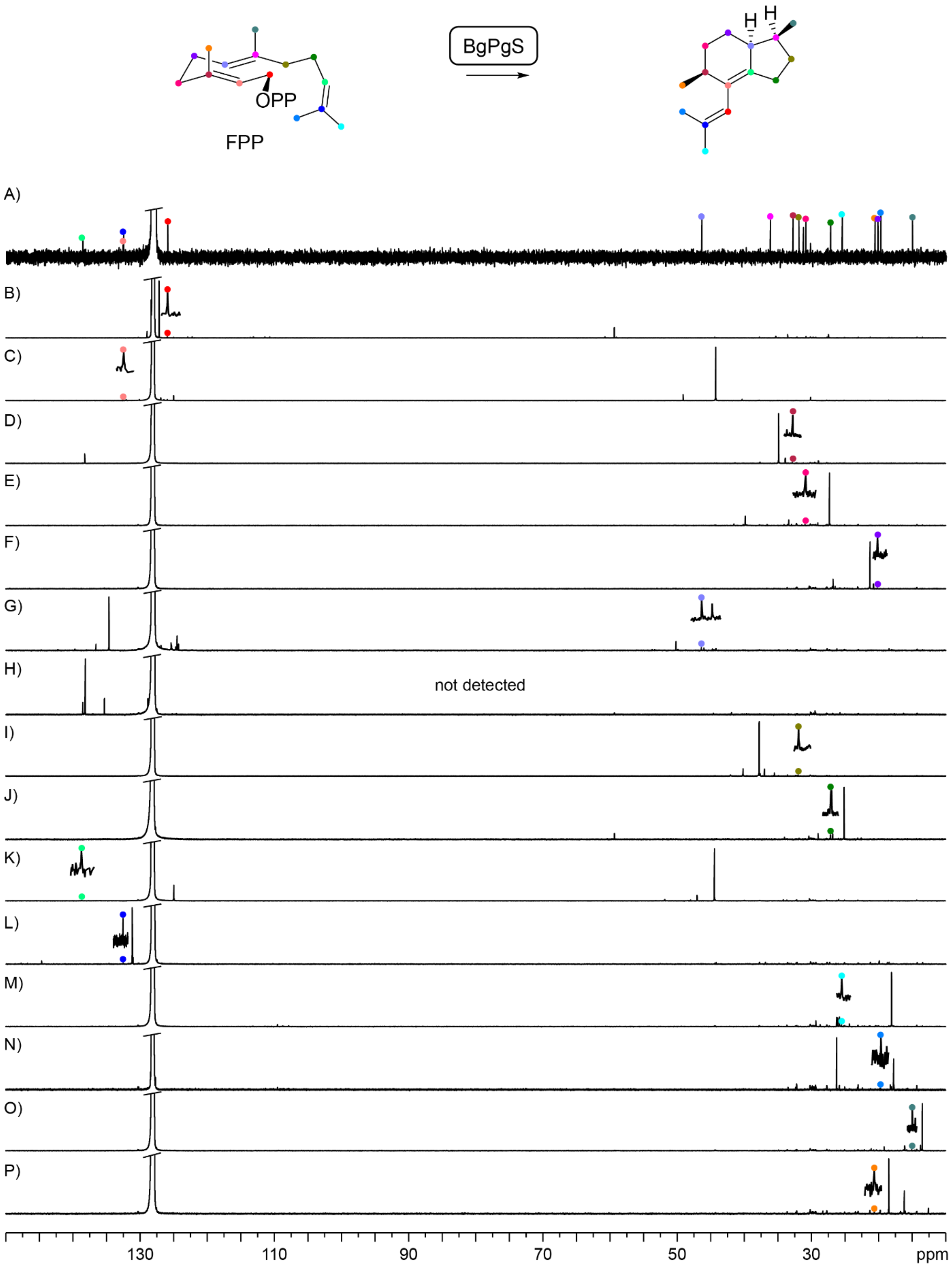

Figure S68. ^{13}C-NMR spectra of A) unlabelled **14**, and B) – P) the mixture of products obtained with BgPgS from (1-^{13}C)FPP – (15-^{13}C)FPP. The coloured dots show the site of incorporation into **14** and indicate the corresponding signal in the ^{13}C-NMR spectra. Incorporation for C7 was below the limit of detection.

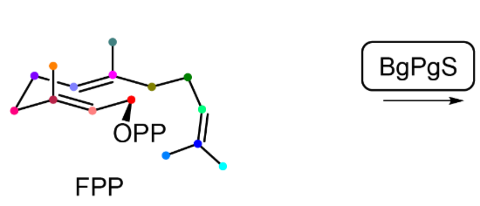

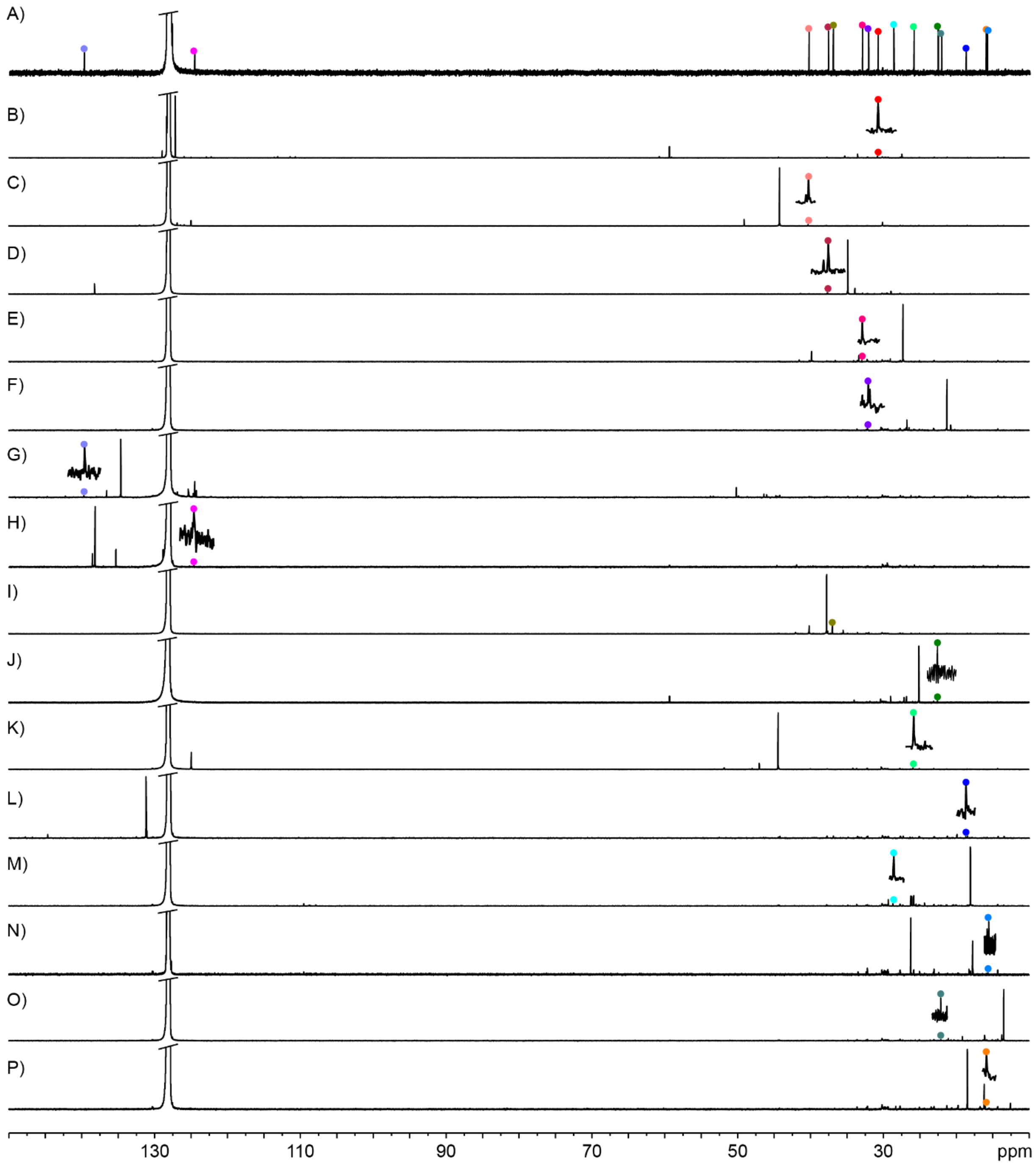

Figure S69. ^{13}C-NMR spectra of A) unlabelled **15**, and B) – P) the mixture of products obtained with BgPgS from (1-^{13}C)FPP – (15-^{13}C)FPP. The coloured dots show the site of incorporation into **15** and indicate the corresponding signal in the ^{13}C-NMR spectra.

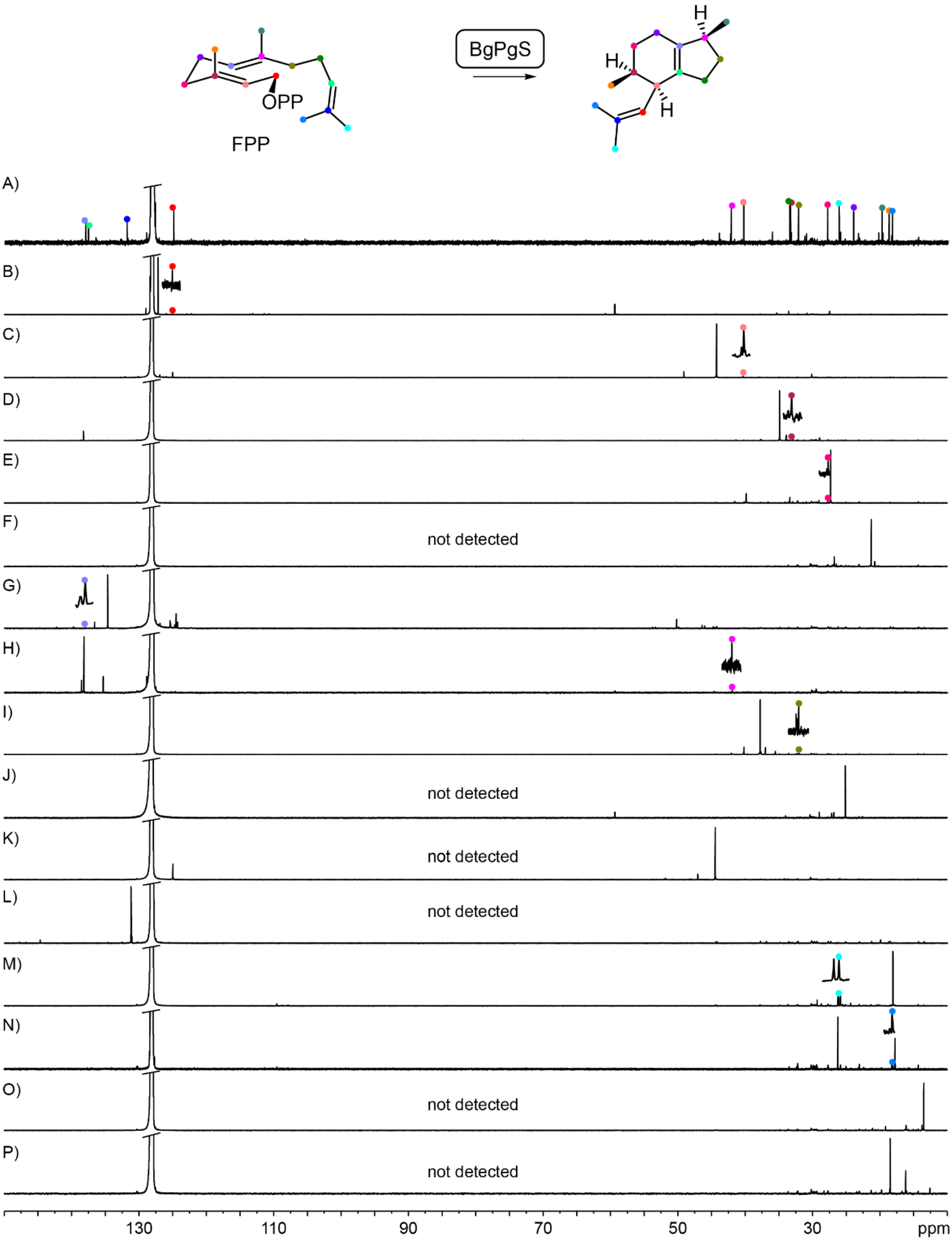

Figure S70. ^{13}C-NMR spectra of A) unlabelled **9**, and B) – P) the mixture of products obtained with BgPgS from (1-^{13}C)FPP – (15-^{13}C)FPP. The coloured dots show the site of incorporation into **9** and indicate the corresponding signal in the ^{13}C-NMR spectra. Incorporations for C5, C9, C10, C11, C14 and C15 were below the limit of detection.

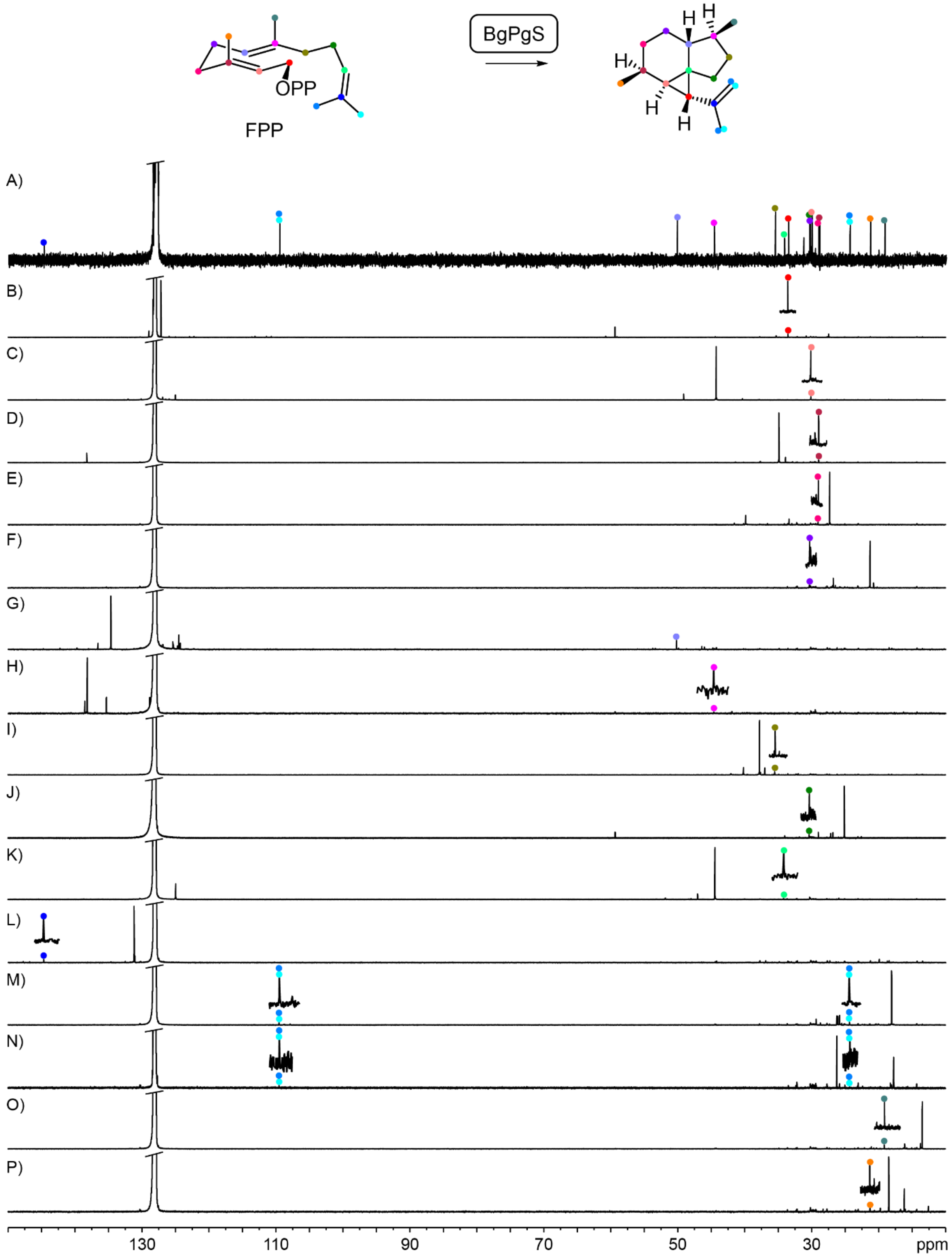

Figure S71. ^{13}C-NMR spectra of A) unlabelled **6**, and B) – P) the mixture of products obtained with BgPgS from (1-^{13}C)FPP – (15-^{13}C)FPP. The coloured dots show the site of incorporation into **6** and indicate the corresponding signal in the ^{13}C-NMR spectra.

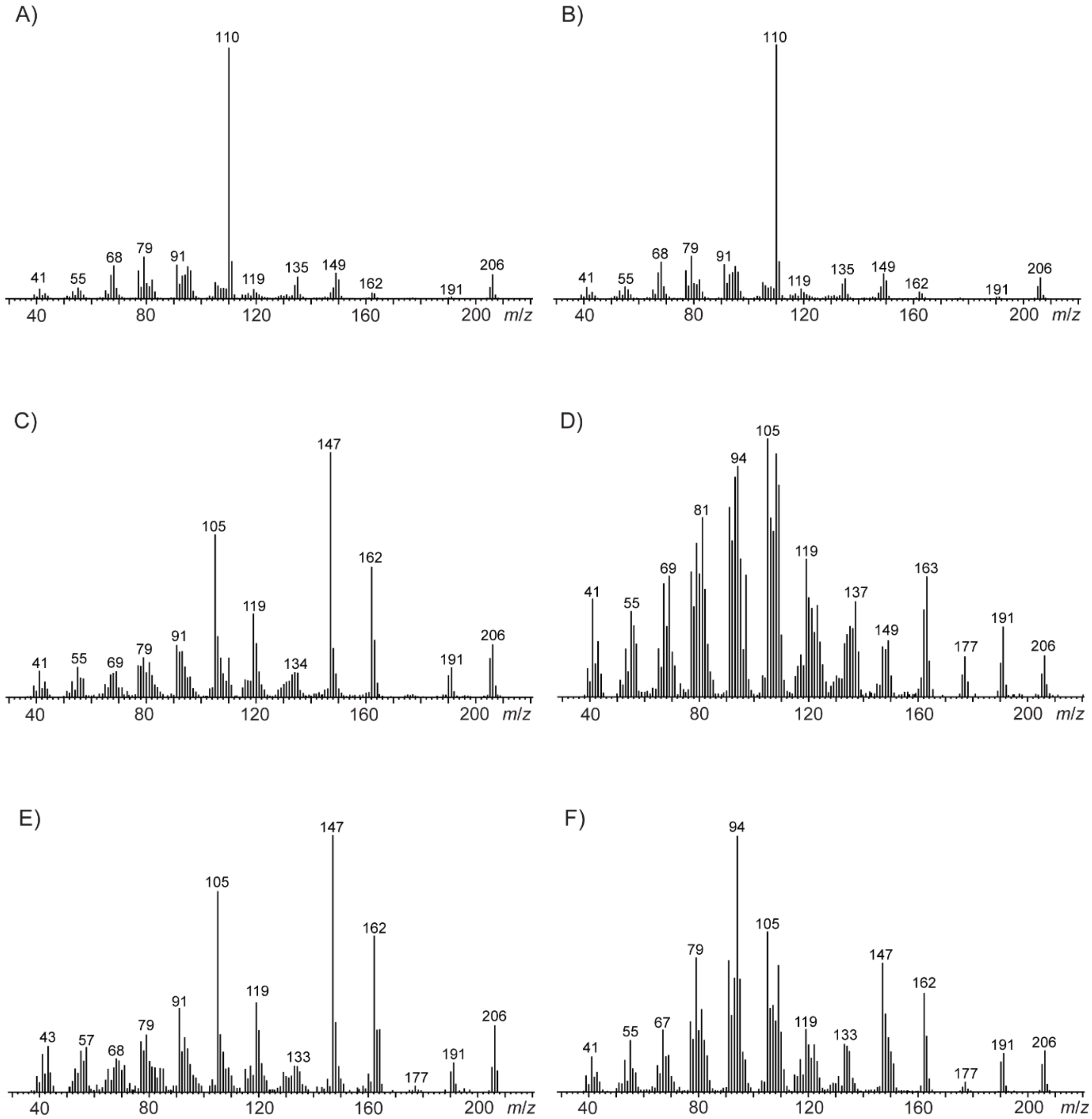

Figure S72. The reprotonation step in the formation of BgPgS products. EI mass spectra of labelled A) 1-*epi*-pacifigorgia-6,10-diene (**12**), B) 1,2-*diepi*-pacifigorgia-6,10-diene (**13**), C) 6-*epi*-pacifigorgia-1,10-diene (**14**), D) ledene (**15**), E) pacifigorgia-1(6),10-diene (**9**) and F) tamariscene (**6**) obtained from an incubation of (3-^{13}C)FPP in a deuterium oxide buffer with BgPgS. The molecular ions at *m*/*z* 206 indicate incorporation of one 13C atom and one deuterium atom from the buffer.

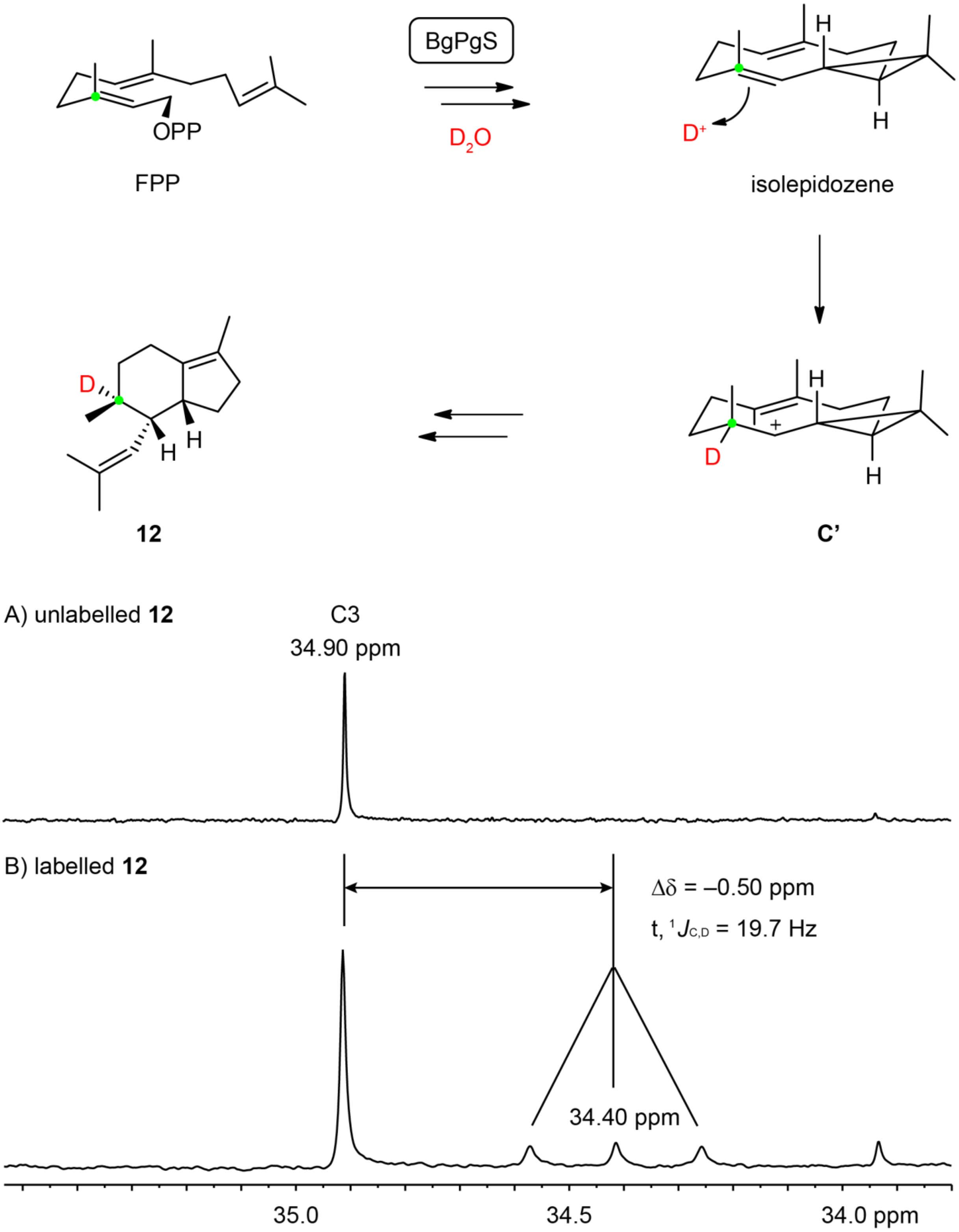

Figure S73. The reprotonation of isolepidozene in the formation of **12**. ^{13}C-NMR spectra showing the region for C3 of **12** for A) unlabelled **12**, and B) labelled **12** obtained from (3-^{13}C)FPP in a deuterium oxide buffer with BgPgS. The upfield shifted triplet is indicative for a direct ^{13}C-^{2}H bond and supports the proposed reprotonation of isolepidozene at C3. Green dots represent ^{13}C-labelled carbons.

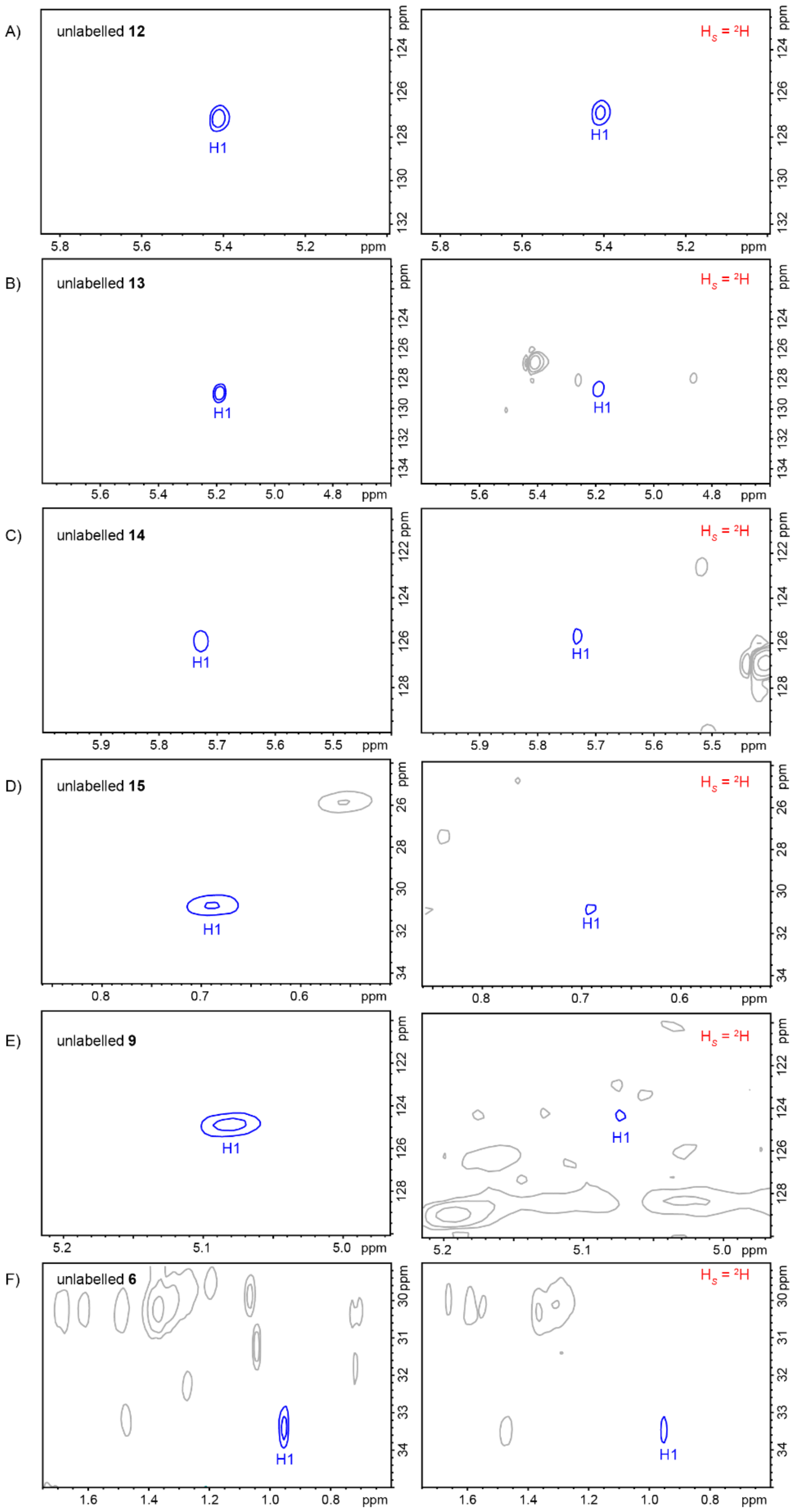

Figure S74. The deprotonation from bicyclogermacrene to **B** and isolepidozene to **B'**. Partial HSQC spectra of unlabelled compounds (left) and labelled compounds obtained from (*S*)-(1-^{13}C,1-^{2}H)IPP (H_S = ^{2}H) after incubation with IDI, FPPS and BgPgS (right). A) (–)-1-*epi*-Pacifigorgia-6,10-diene (**12**), B) (–)-1,2-*diepi*-pacifigorgia-6,10-diene (**13**), C) (–)-6-*epi*-pacifigorgia-1,10-diene (**14**), D) (–)-ledene (**15**), E) (–)-pacifigorgia-1(6),10-diene (**9**) and F) (–)-tamariscene (**6**). The observation of a crosspeak for H1 in all cases is in line with retainment of the 1-*pro*-*R* hydrogen and loss of the 1-*pro*-*S* hydrogen.

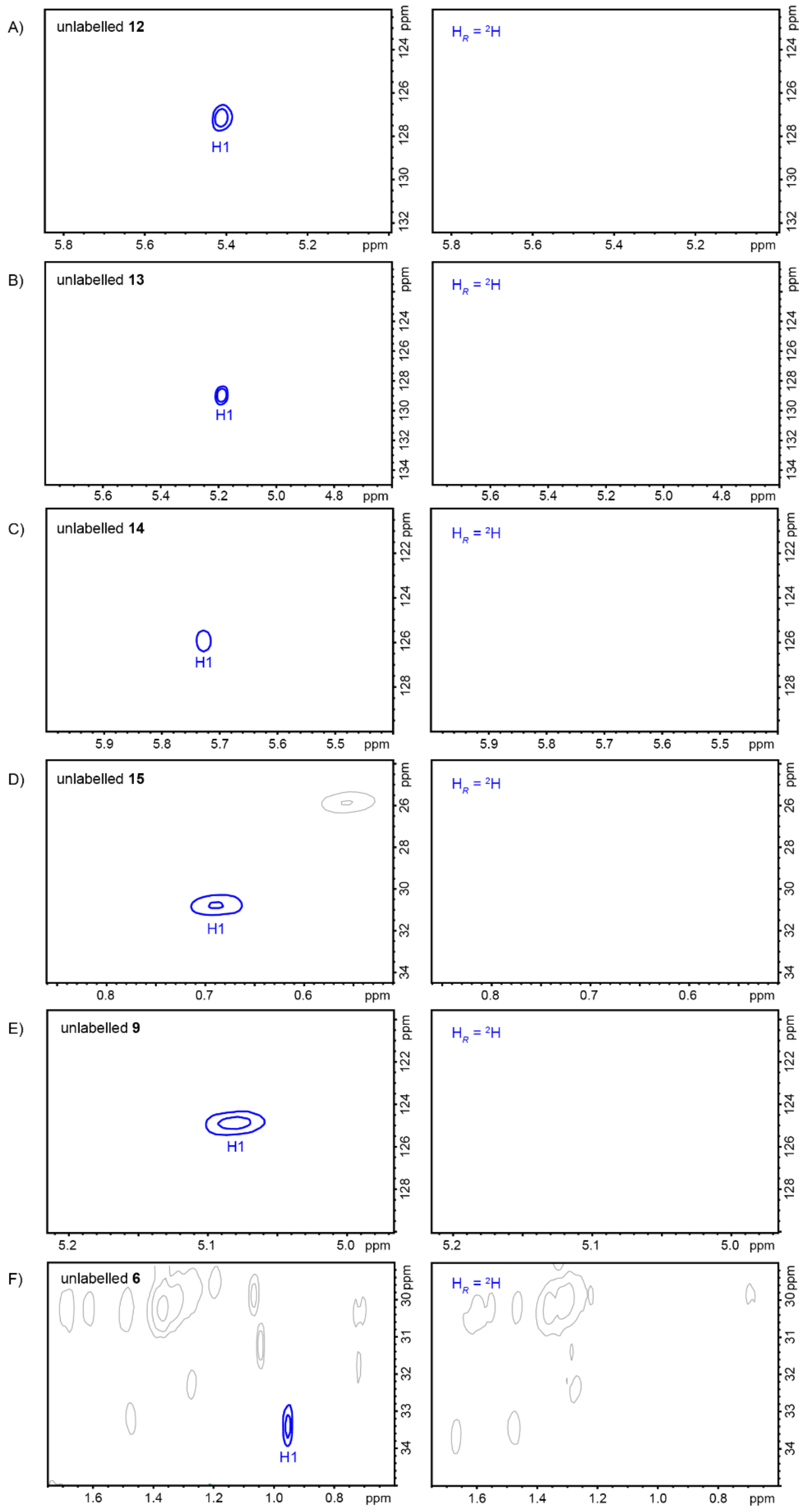

Figure S75. The deprotonation from bicyclogermacrene to **B** and isolepidozene to **B'**. Partial HSQC spectra of unlabelled compounds (left) and labelled compounds obtained from (*R*)-(1-^{13}C,1-^{2}H)IPP (H_R = ^{2}H) after incubation with IDI, FPPS and BgPgS (right). A) (–)-1-*epi*-Pacifigorgia-6,10-diene (**12**), B) (–)-1,2-*diepi*-pacifigorgia-6,10-diene (**13**), C) (–)-6-*epi*-pacifigorgia-1,10-diene (**14**), D) (–)-ledene (**15**), E) (–)-pacifigorgia-1(6),10-diene (**9**) and F) (–)-tamariscene (**6**). The lack of a crosspeak for H1 in all cases is in line with retainment of the 1-*pro*-*R* hydrogen and loss of the 1-*pro*-*S* hydrogen.

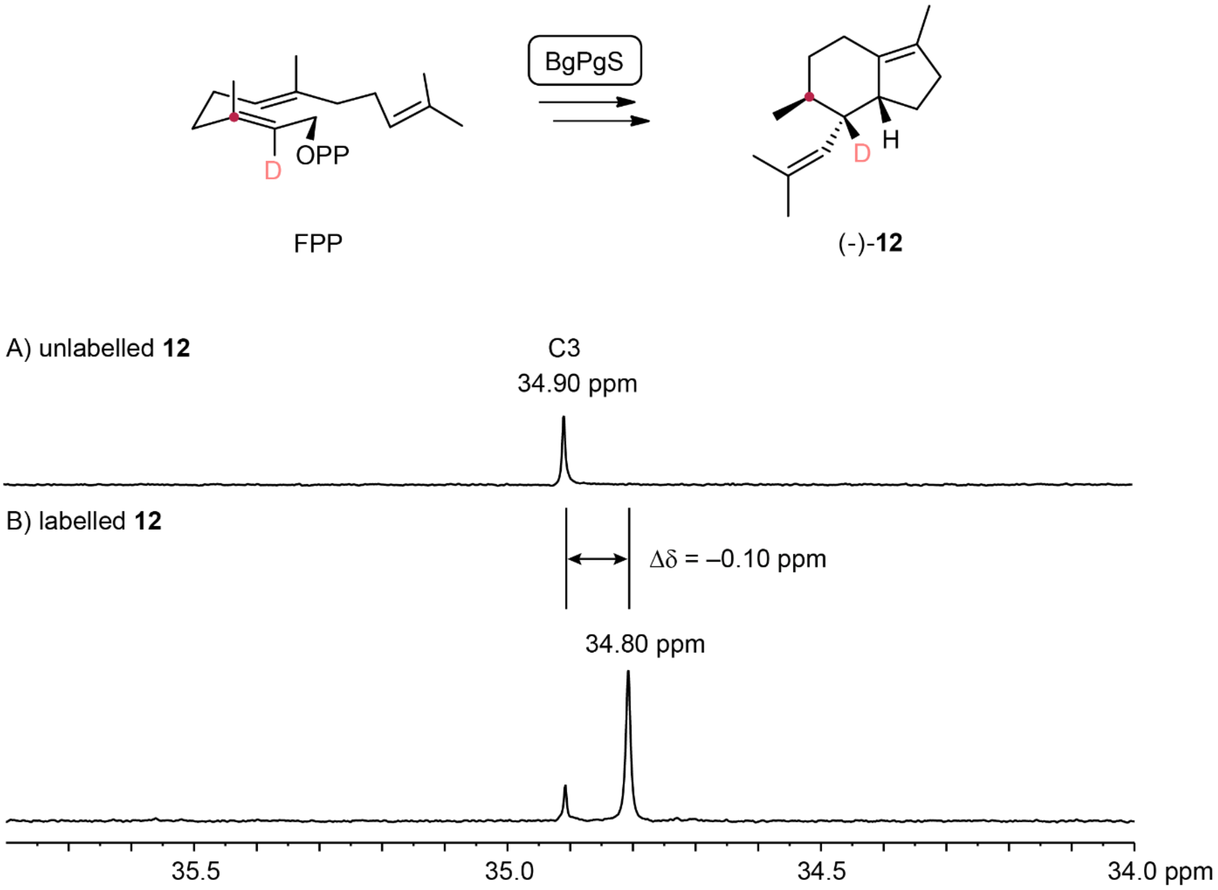

Figure S76. The site of incorporation of H2 in **12**. ^{13}C-NMR spectra showing the region for C3 of A) unlabelled **12** and B) labelled **12** obtained from (3-^{13}C,2-^{2}H)FPP with BgPgS (red dots represent ^{13}C-labelled carbons). The slightly upfield shifted peak in B) is characteristic of a deuterium substitution at a neighbouring position, indicating that H2 remains at C2 in **12**.

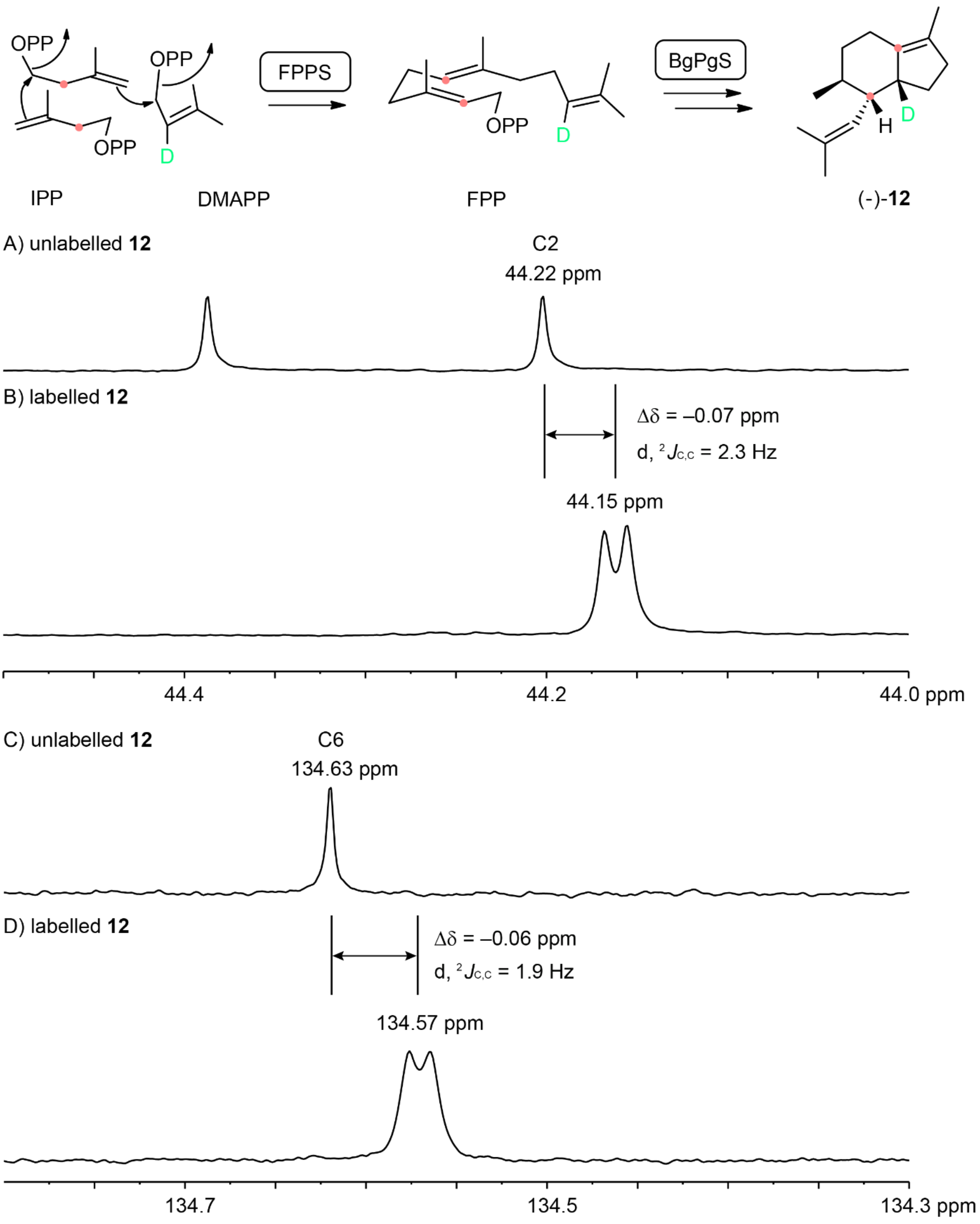

Figure S77. The site of incorporation of H10 in **12**. ^{13}C-NMR spectra showing the region of C2 of A) unlabelled **12** and B) labelled **12** obtained from the conversion of (2-^{2}H)DMAPP and (2-^{13}C)IPP with FPPS and BgPgS, and showing the region for C6 of C) unlabelled **12** and D) labelled **12** obtained from the conversion of (2-^{2}H)DMAPP and (2-^{13}C)IPP with FPPS and BgPgS (red dots represent ^{13}C-labelled carbons). The slightly upfield shifted doublets observed in B) and in D) are consistent with the presence of a neighbouring deuterium atom, suggesting that H10 is retained at C10 in **12**.

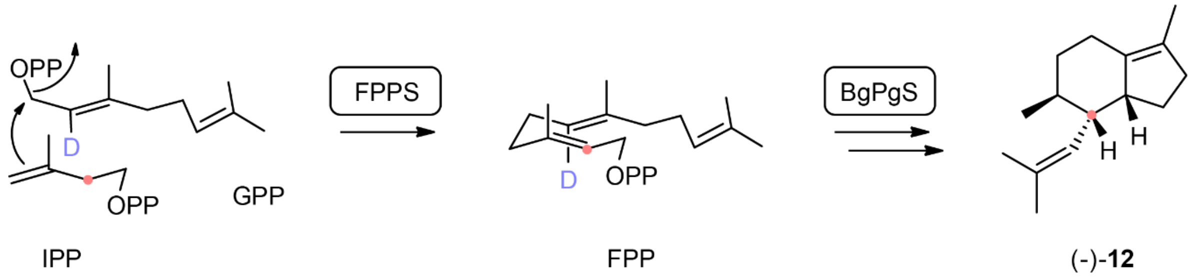

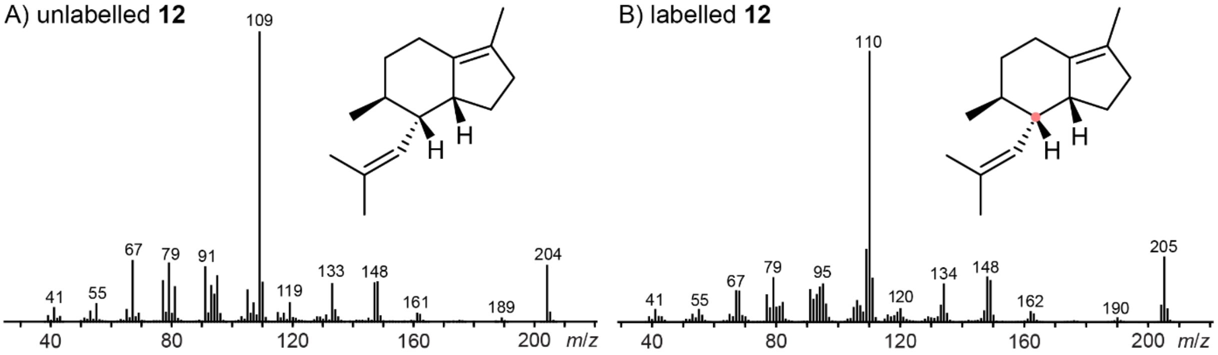

Figure S78. The loss of H6 in the biosynthesis of **12**. EI mass spectra of A) unlabelled **12** and B) labelled **12** obtained from the conversion of (2-^{2}H)GPP and (2-^{13}C)IPP with FPPS and BgPgS (red dots represent ^{13}C-labelled carbons). The molecular ion in B) at *m*/*z* 205 confirms the loss of H6 in the biosynthesis of **12**.

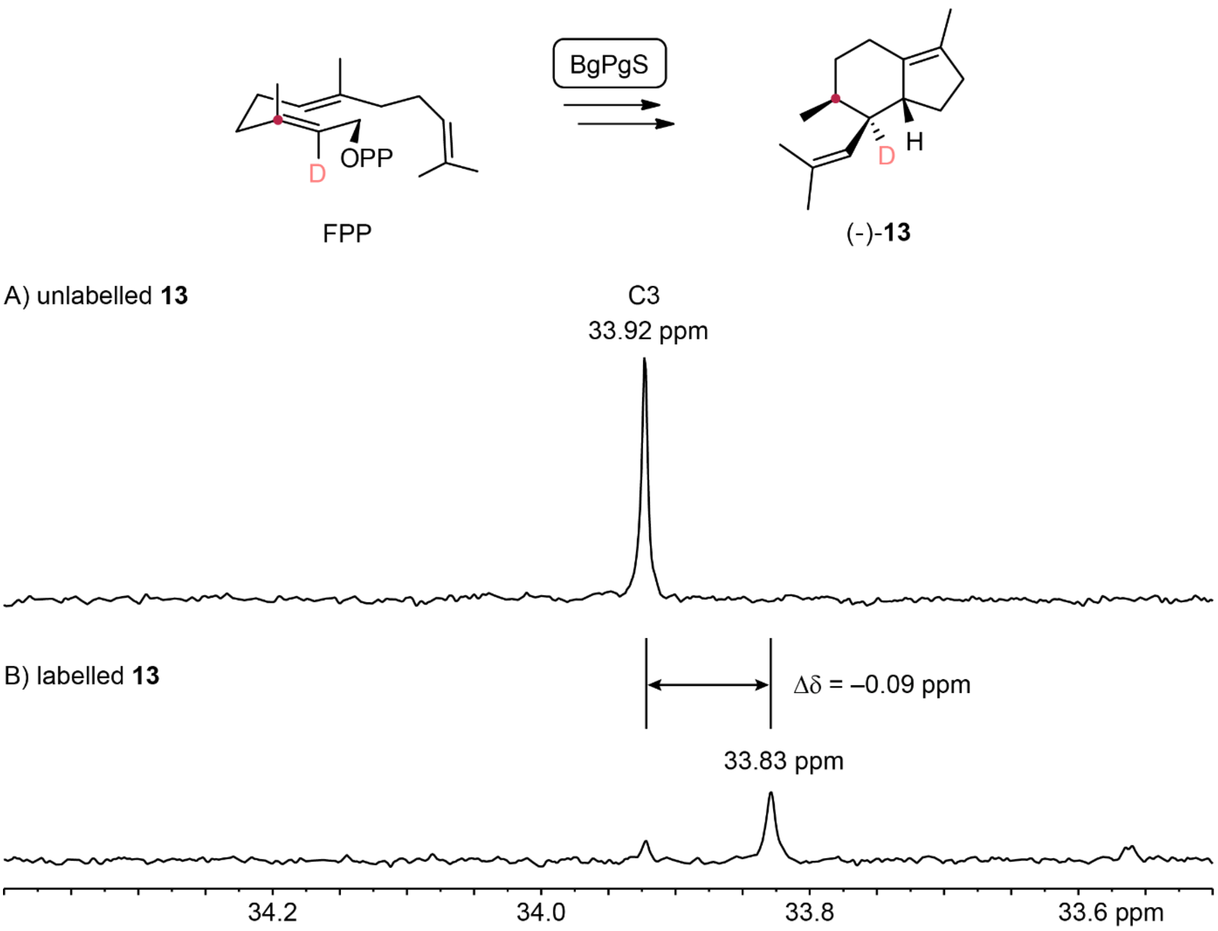

Figure S79. The site of incorporation of H2 in **13**. ^{13}C-NMR spectra showing the region for C3 of A) unlabelled **13** and B) labelled **13** obtained from (3-^{13}C,2-^{2}H)FPP with BgPgS (red dots represent ^{13}C-labelled carbons). The slightly upfield shifted peak in B) is characteristic of a deuterium substitution at a neighbouring position, indicating that H2 remains at C2 in **13**.

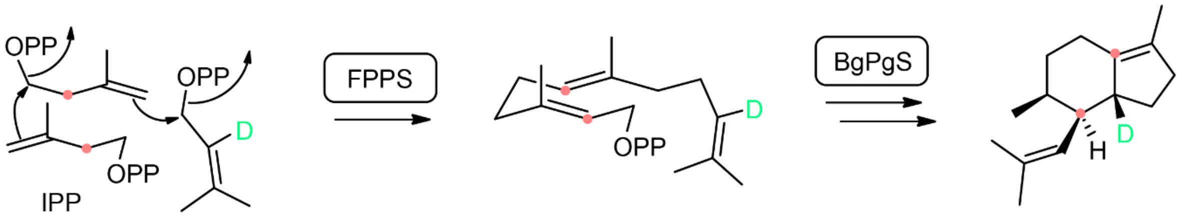

DMAPP FPP (-)-**13**

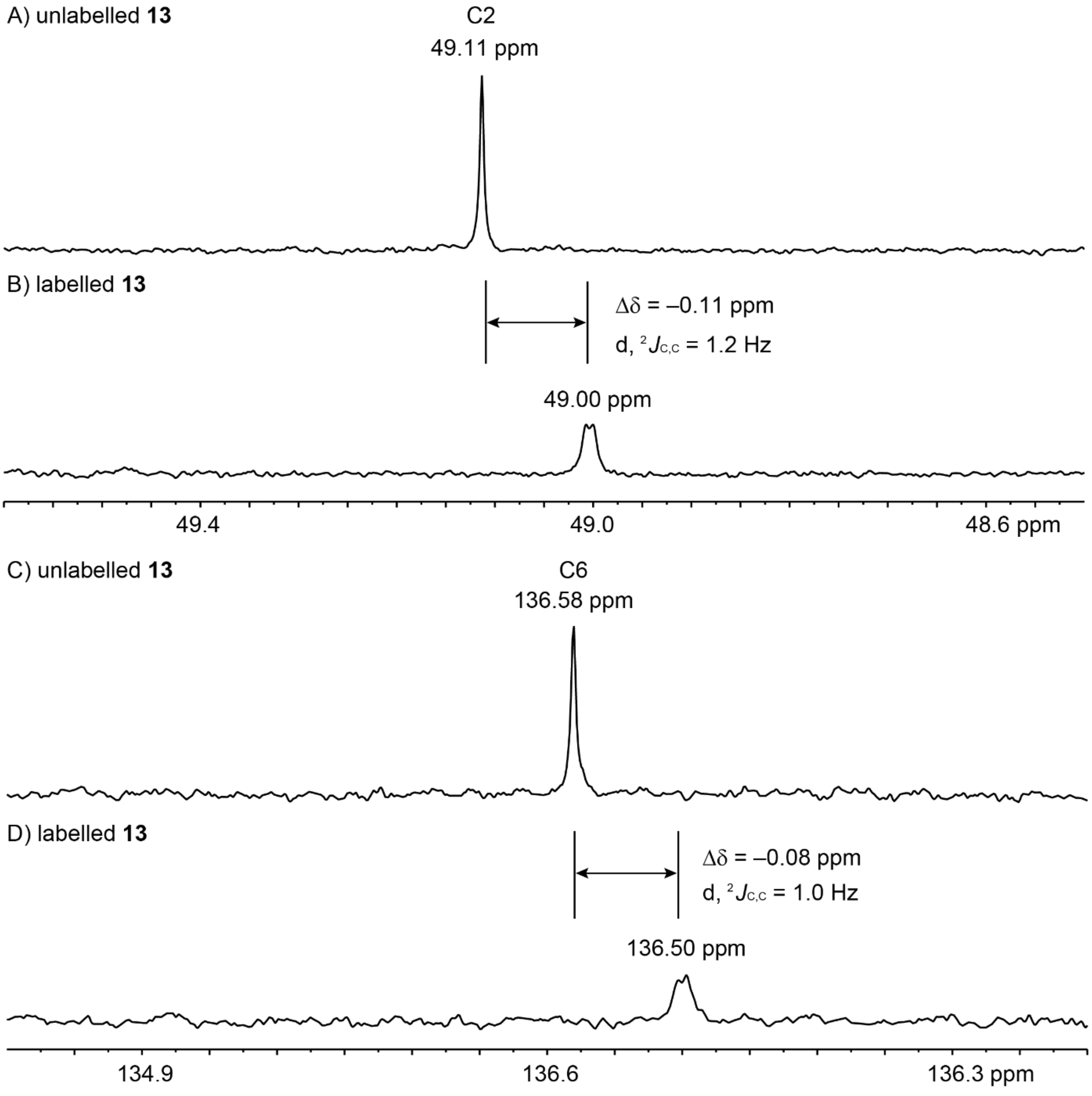

Figure S80. The site of incorporation of H10 in **13**. ^{13}C-NMR spectra showing the region of C2 of A) unlabelled **13** and B) labelled **13** obtained from the conversion of (2-^{2}H)DMAPP and (2-^{13}C)IPP with FPPS and BgPgS, and showing the region for C6 of C) unlabelled **13** and D) labelled **13** obtained from the conversion of (2-^{2}H)DMAPP and (2-^{13}C)IPP with FPPS and BgPgS (red dots represent ^{13}C-labelled carbons). The slightly upfield shifted doublets observed in B) and in D) are consistent with the presence of a neighbouring deuterium atom, suggesting that H10 is retained at C10 in **13**.

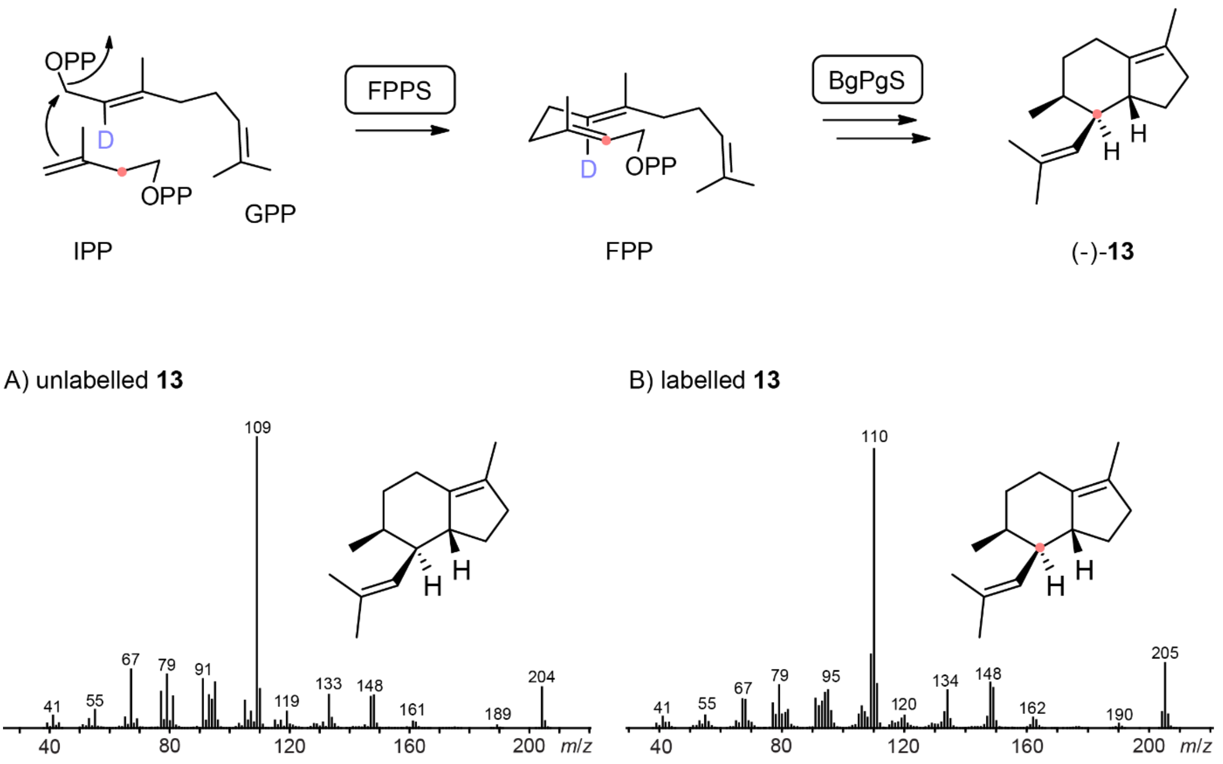

Figure S81. The loss of H6 in the biosynthesis of **13**. EI mass spectra of A) unlabelled **13** and B) labelled **13** obtained from the conversion of (2-^{2}H)GPP and (2-^{13}C)IPP with FPPS and BgPgS (red dots represent ^{13}C-labelled carbons). The molecular ion in B) at *m*/*z* 205 confirms the loss of H6 in the biosynthesis of **13**.

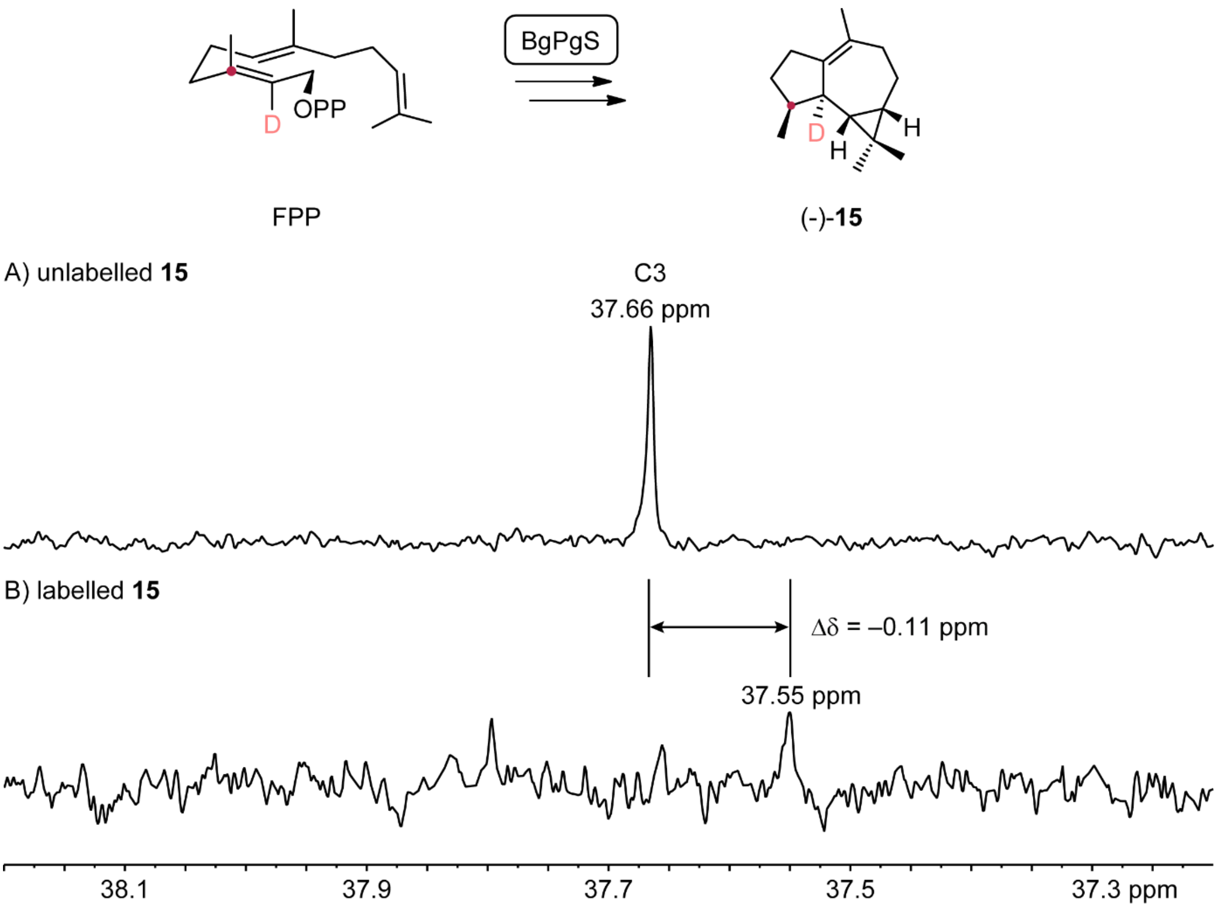

Figure S82. The site of incorporation of H2 in **15**. ^{13}C-NMR spectra showing the region for C3 of A) unlabelled **15** and B) labelled **15** obtained from (3-^{13}C,2-^{2}H)FPP with BgPgS (red dots represent ^{13}C-labelled carbons). The slightly upfield shifted peak in B) is characteristic of a deuterium substitution at a neighbouring position, indicating that H2 remains at C2 in **15**.

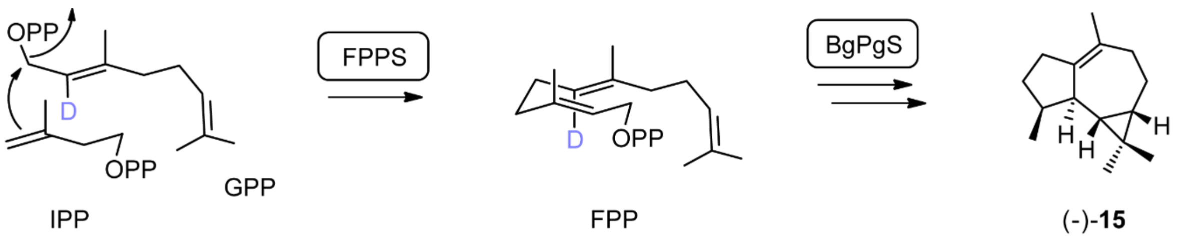

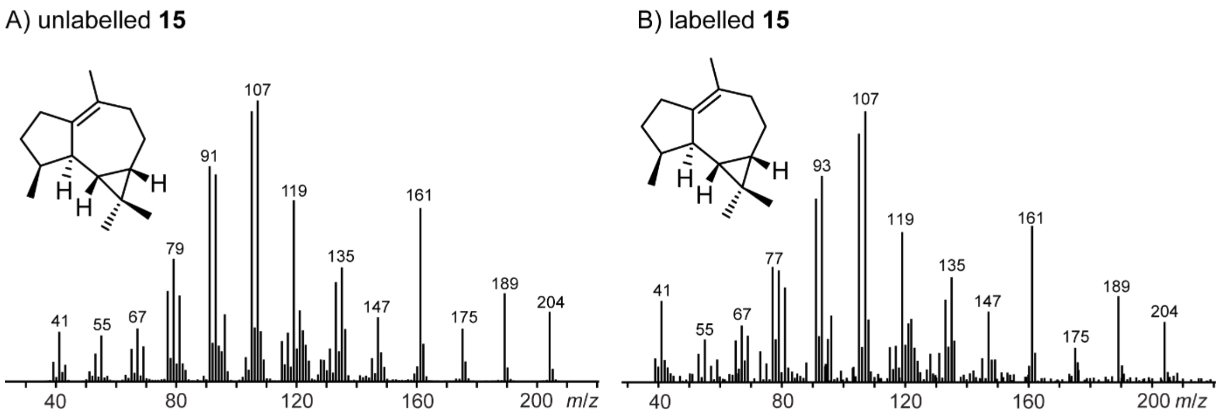

Figure S83. The loss of H6 in the biosynthesis of **15**. EI mass spectra of A) unlabelled **15** and B) labelled **15** obtained from the conversion of (2-^{2}H)GPP and IPP with FPPS and BgPgS. The molecular ion in B) at *m*/*z* 204 confirms the loss of H6 in the biosynthesis of **15**. To separate the trace product 15 from another unknown compound, the analysis of the sample in B) was performed using a chiral Cyclosil-B GC column.

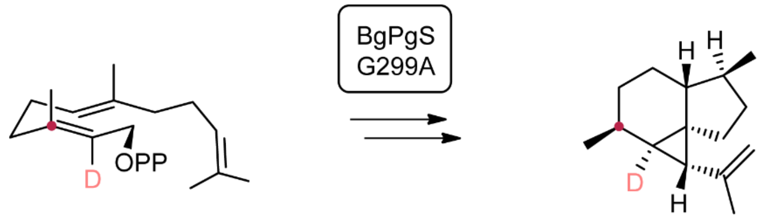

FPP (-)-**6**

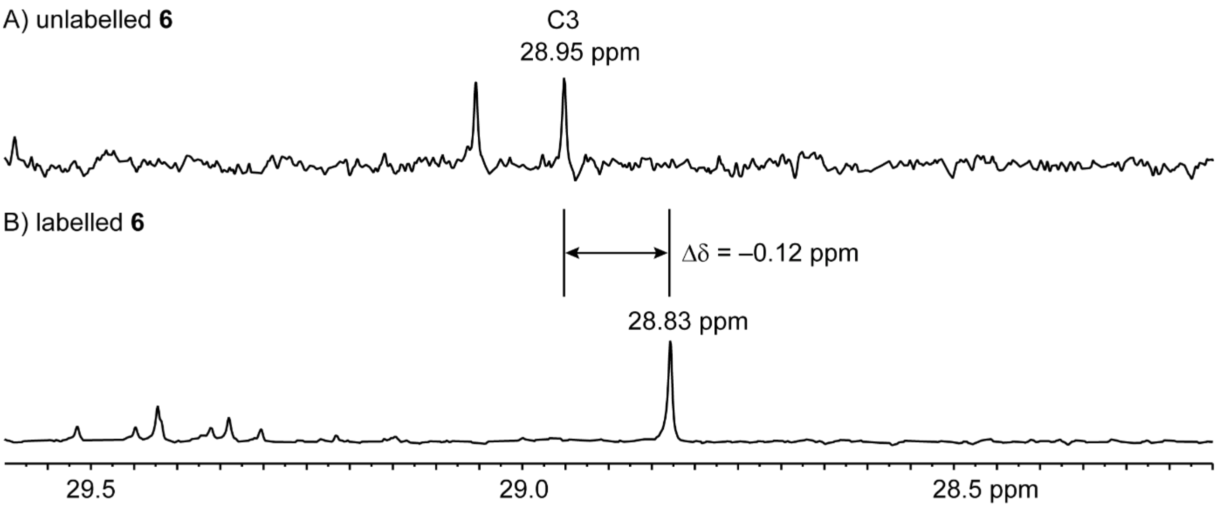

Figure S84. The site of incorporation of H2 in **6**. ^{13}C-NMR spectra showing the region for C3 of A) unlabelled **6** and B) labelled **6** obtained from (3-^{13}C,2-^{2}H)FPP with BgPgS-G299A (red dots represent ^{13}C-labelled carbons). The slightly upfield shifted peak in B) is characteristic of a deuterium substitution at a neighbouring position, indicating that H2 remains at C2 in **6**.

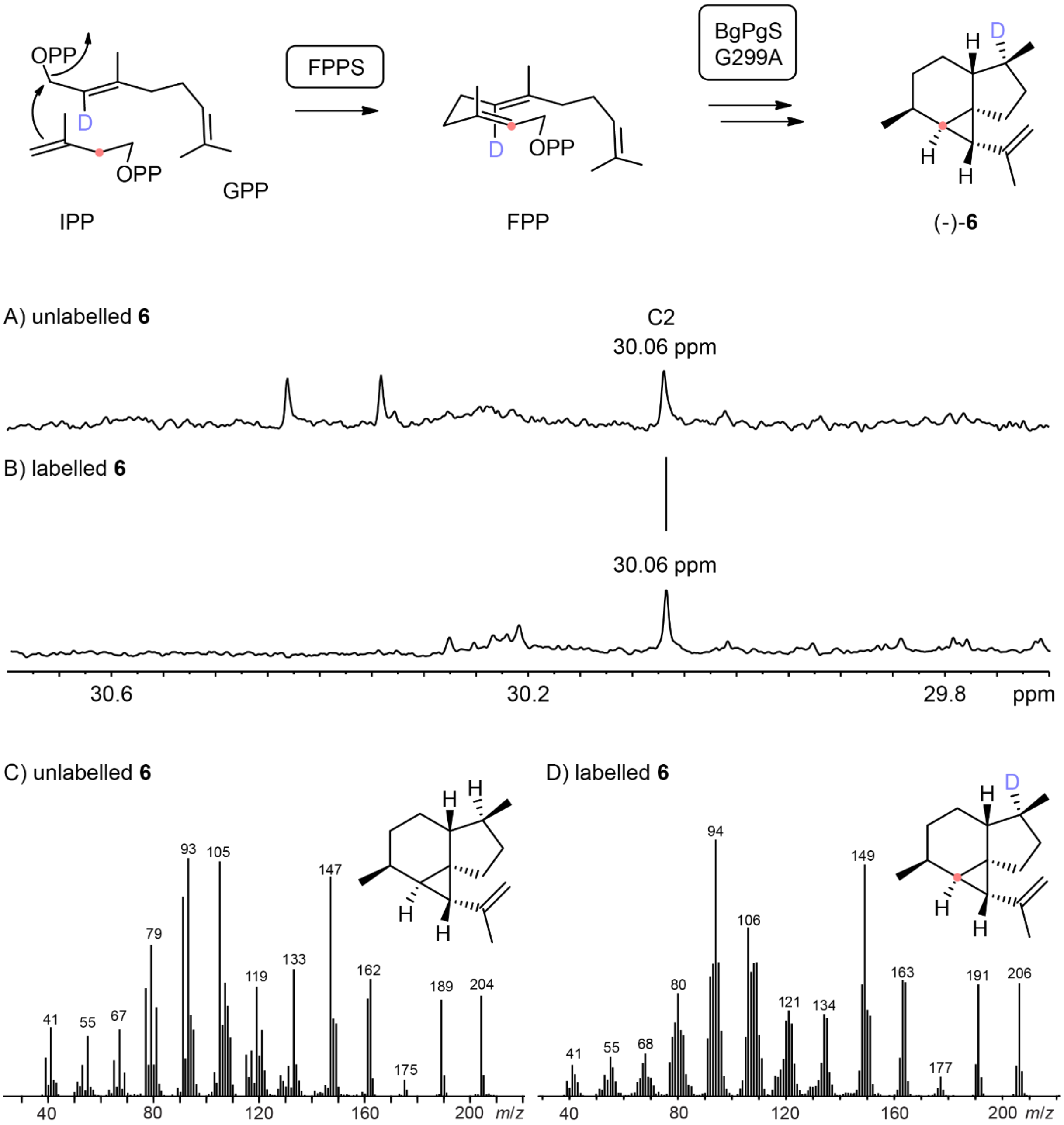

Figure S85. The site of incorporation of H6 in **6**. ^{13}C-NMR spectra showing the region for C2 of A) unlabelled **6** and B) labelled **6** obtained from (2-^{2}H)GPP and (2-^{13}C)IPP with FPPS and BgPgS-G299A (red dots represent ^{13}C-labelled carbons). The unchanged peak in B) indicates deuterium residing in a distant position (C7). EI mass spectra of C) unlabelled **6** and D) labelled **6** obtained from (2-^{2}H)GPP and (2-^{13}C)IPP with FPPS and BgPgS-G299A. The molecular ion at *m*/*z* 206 indicates retainment of deuterium (loss of deuterium could also explain the unchanged signal in B).

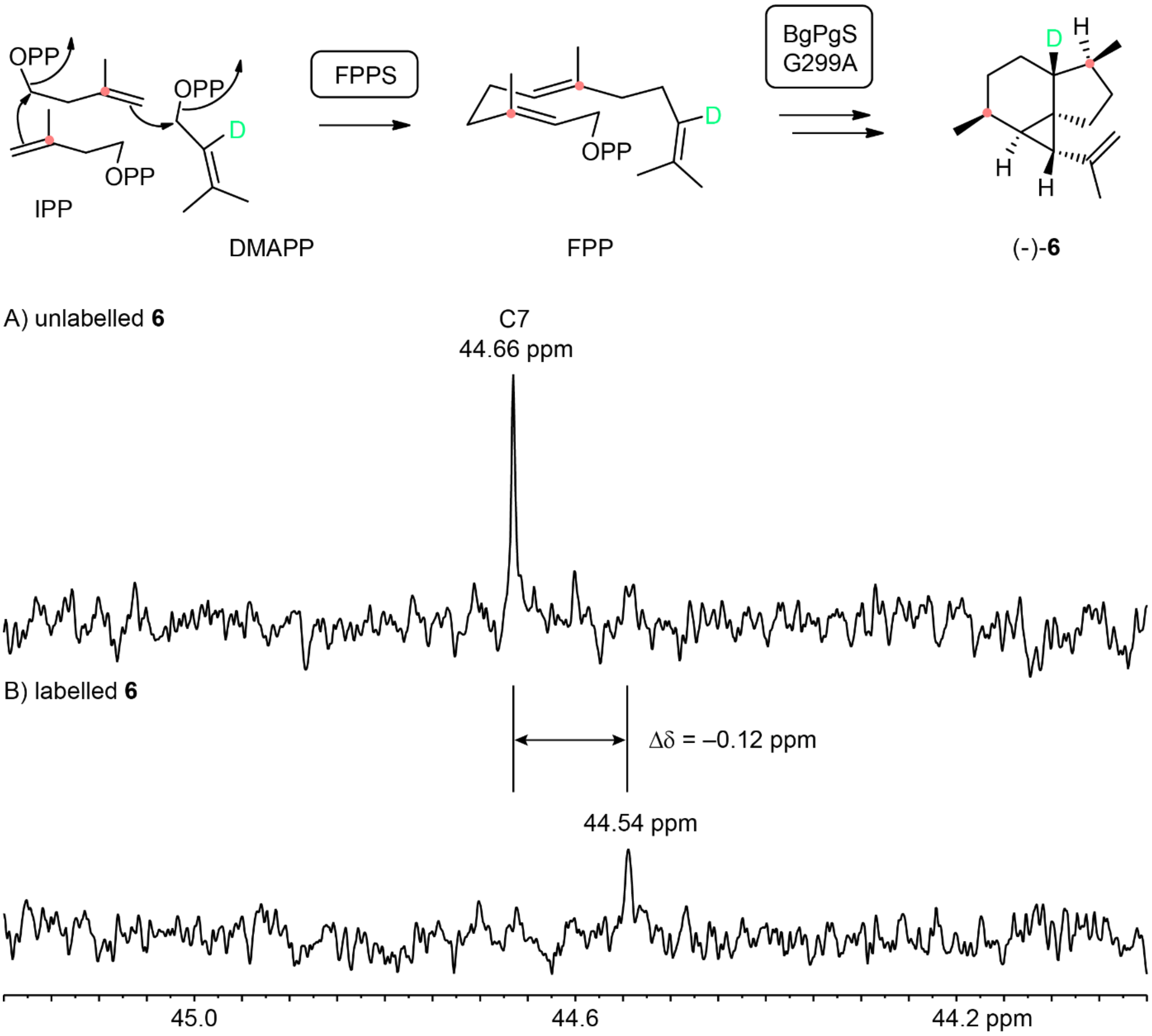

Figure S86. The site of incorporation of H10 in **6**. ^{13}C-NMR spectra showing the region for C7 of A) unlabelled **6** and B) labelled **6** obtained from (2-^{2}H)DMAPP and (3-^{13}C)IPP with FPPS and BgPgS-G299A (red dots represent ^{13}C-labelled carbons). The slightly upfield shifted peak in B) is characteristic of a deuterium substitution at a neighbouring position, indicating that H10 resides at C6 of **6**.

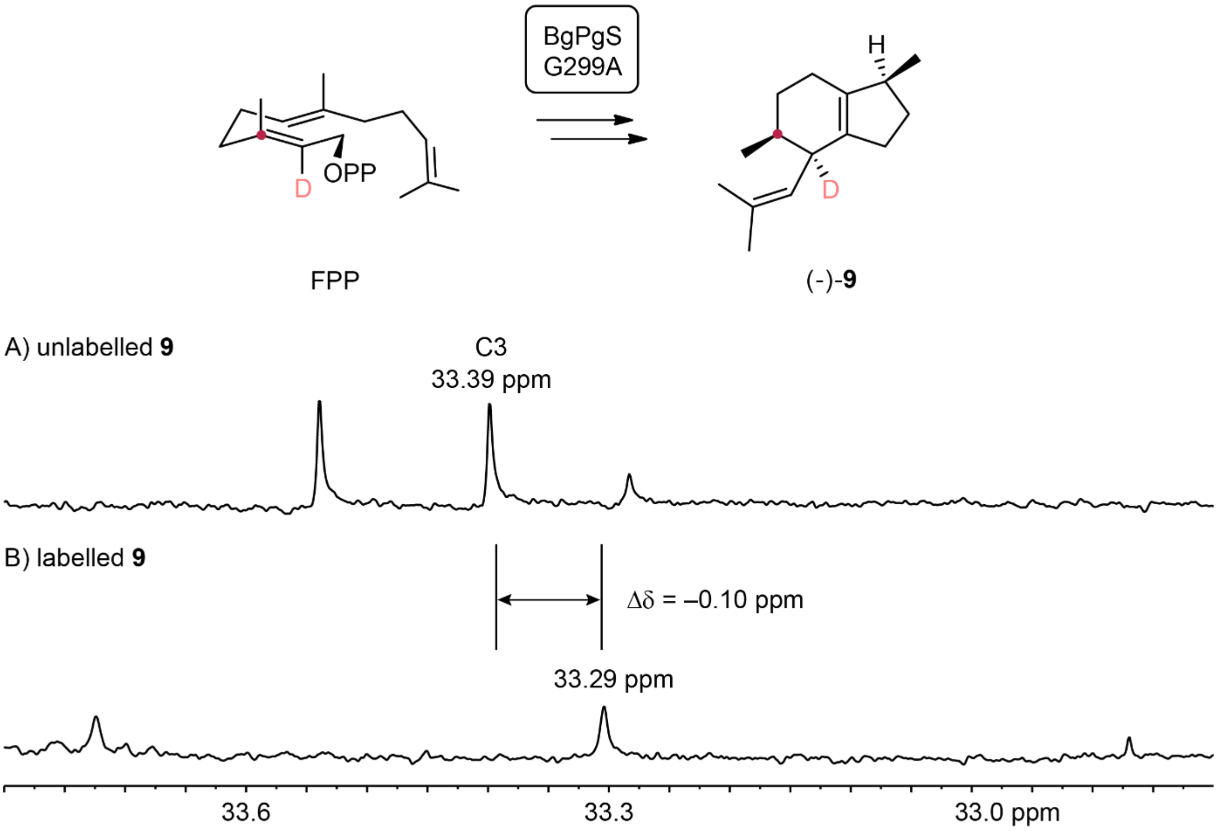

Figure S87. The site of incorporation of H2 in **9**. ^{13}C-NMR spectra showing the region for C3 of A) unlabelled **9** and B) labelled **9** obtained from (3-^{13}C,2-^{2}H)FPP with BgPgS-G299A (red dots represent ^{13}C-labelled carbons). The slightly upfield shifted peak in B) is characteristic of a deuterium substitution at a neighbouring position, indicating that H2 remains at C2 in **9**.

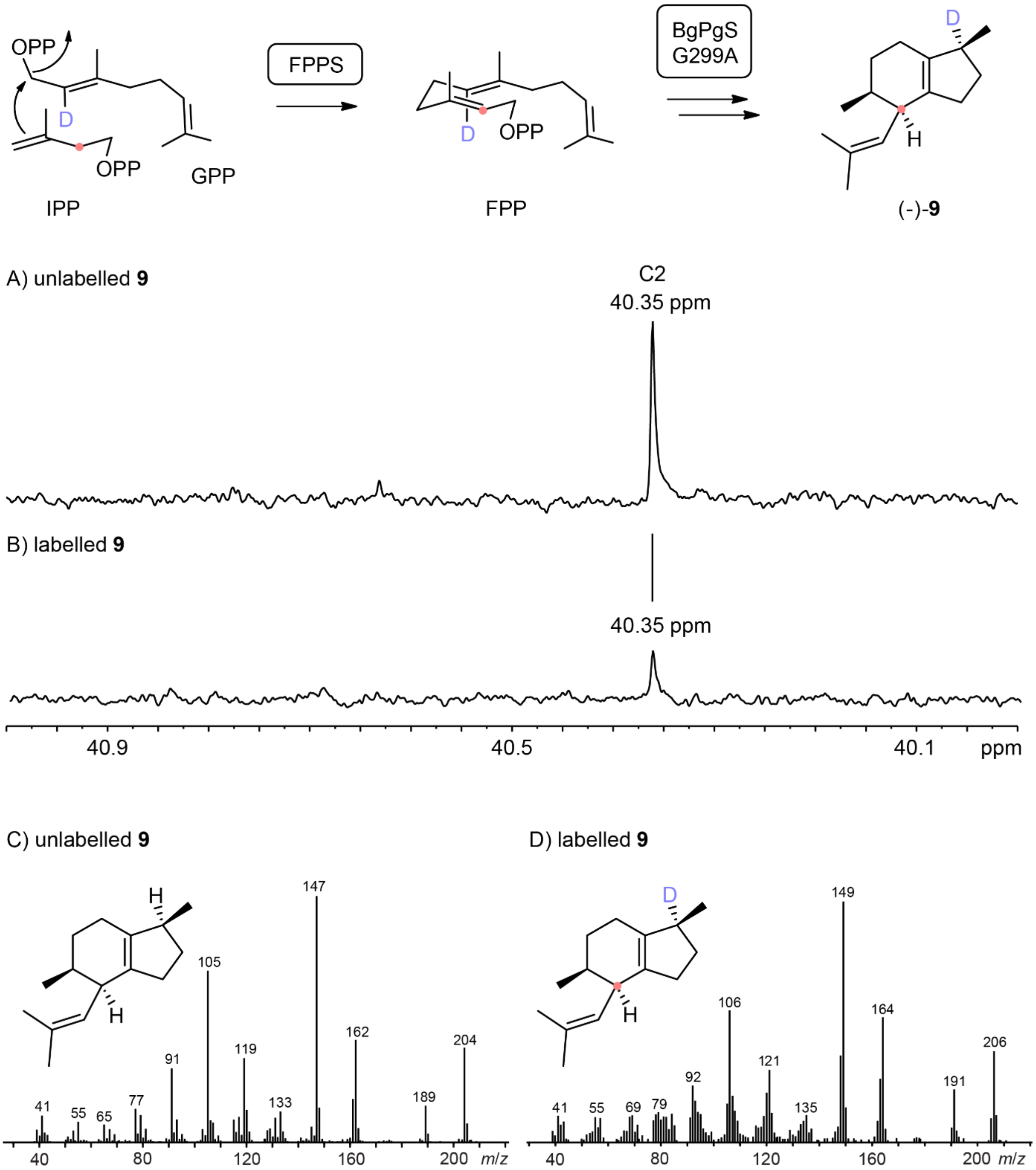

Figure S88. The site of incorporation of H6 in **9**. ^{13}C-NMR spectra showing the region for C2 of A) unlabelled **6** and B) labelled **6** obtained from (2-^{2}H)GPP and (2-^{13}C)IPP with FPPS and BgPgS-G299A (red dots represent ^{13}C-labelled carbons). The unchanged peak in B) indicates deuterium residing in a distant position (C7). EI mass spectra of C) unlabelled **9** and D) labelled **9** obtained from (2-^{2}H)GPP and (2-^{13}C)IPP with FPPS and BgPgS-G299A. The molecular ion at *m*/*z* 206 indicates retainment of deuterium.

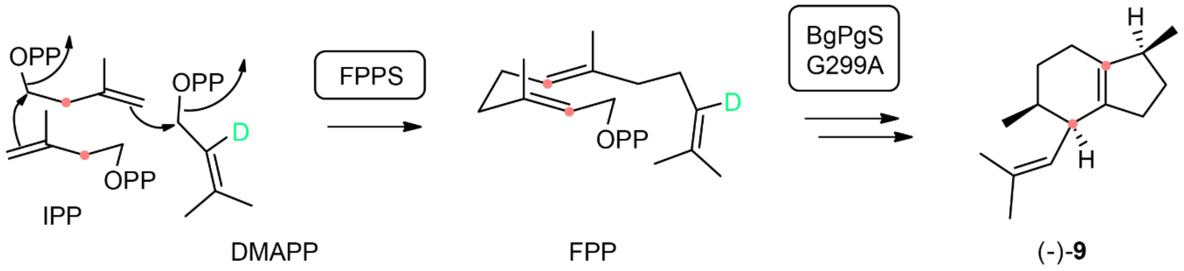

A) unlabelled **9**

B) labelled **9**

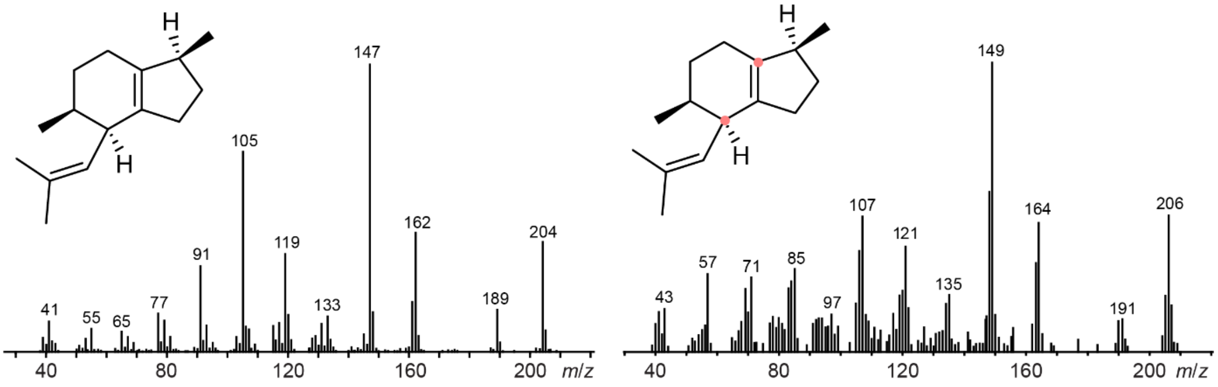

Figure S89. The loss of H10 in the biosynthesis of **9**. EI mass spectra of A) unlabelled **9** and B) labelled **9** obtained from the conversion of (2-^{2}H)DMAPP and (2-^{13}C)IPP with FPPS and BgPgS (red dots represent ^{13}C-labelled carbons). The molecular ion in B) at *m*/*z* 206 confirms the loss of H10 in the biosynthesis of **9**.

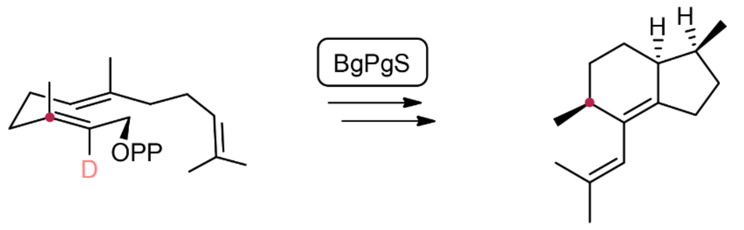

FPP (-)-**14**

A) unlabelled **14**

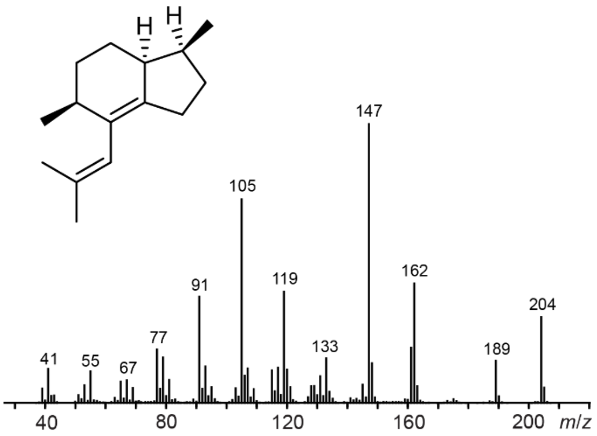

B) labelled **14**

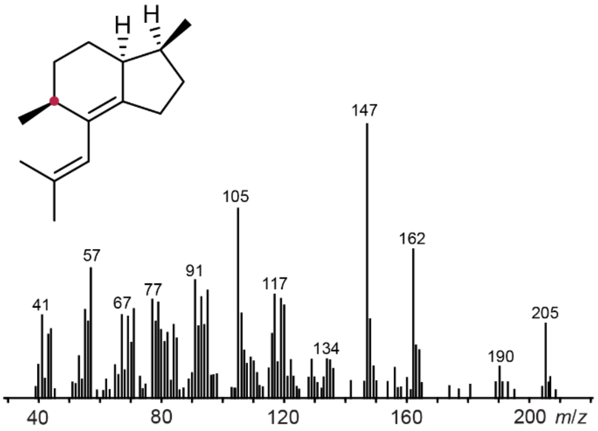

Figure S90. The loss of H2 in the biosynthesis of **14**. EI mass spectra of A) unlabelled **14** and B) labelled **14** obtained from the conversion of (3-^{13}C,2-^{2}H)FPP with BgPgS (red dots represent ^{13}C-labelled carbons). The molecular ion in B) at *m*/*z* 205 confirms the loss of H2 in the biosynthesis of **14**.

Computational Methods

All computed structures were geometry optimized without restrictions and were characterized as minima or as transition state structures by frequency analyses using the B97D3/6-31g(d,p) method with the density fitting approximation for s- and p-functions, including Grimme's empirical D3-dispersion correction[39] in Gaussian16.[40] Frequency computations also provided Gibbs corrections, which include Grimme's quasi-RRHO approach with a frequency cut-off value of 100.0 wave numbers using GoodVibes.[41,42] For single-point energies, Head-Gordon’s range-separated hybrid meta-GGA functional ωB97M-V was applied with the Def2-TZVPPD basis set in Orca (6.1.0), due to its general suitability to describe reaction energies and barriers, also for the chemistry of carbocations.[43-48] Conformational analyses were performed with xTB-GFN2 in the CREST 2.12 program (github.com/crest-lab), developed by the Grimme group.[49-53]

Table S10. Results of DFT calculations (wB97M-V/Def2-TZVPPD//B97D3/6-31G(d,p)) for the cyclisation cascade from FPP to **6**, **9**, **13** and **15** (Scheme 2B of main text).

Structure[a]	Gibbs energy (298.15K) in Hartree	energy relative to C in kcal/mol	free energy of activation / kcal/mol	free reaction energy / kcal/mol
**C**	–585.951890	0.0		
**C-E-TS**	–585.949308	4.1	4.1	
**E**	–585.956506	–2.1		–2.1
**E**	–585.956509	–2.1		
**E-F-TS**	–585.955177	–0.1	1.9	
**F**	–585.971462	–13.1		–11.1
**F**	–585.975564	–15.9		
**F-G-TS**	–585.947317	3.4	19.3	
**G**	–585.968152	–10.1		5.8
**G**	–585.974572	–13.7		
**G-I-TS**	–585.945650	6.0	19.7	
**I**	–585.976540	–15.3		–1.6
**I**	–585.981942	–19.0		
**I-J-TS**	–585.961725	–4.4	14.6	
**J**	–585.971196	–10.9		8.1
**J**	–585.974583	–14.2		
**J-K-TS**	–585.973288	–13.4	0.8	
**K**	–585.975307	–14.7		–0.5
**K**	–585.975296	–14.7		
**K-L-TS**	–585.973128	–13.3	1.4	
**L**	–585.982691	–19.3		–4.6

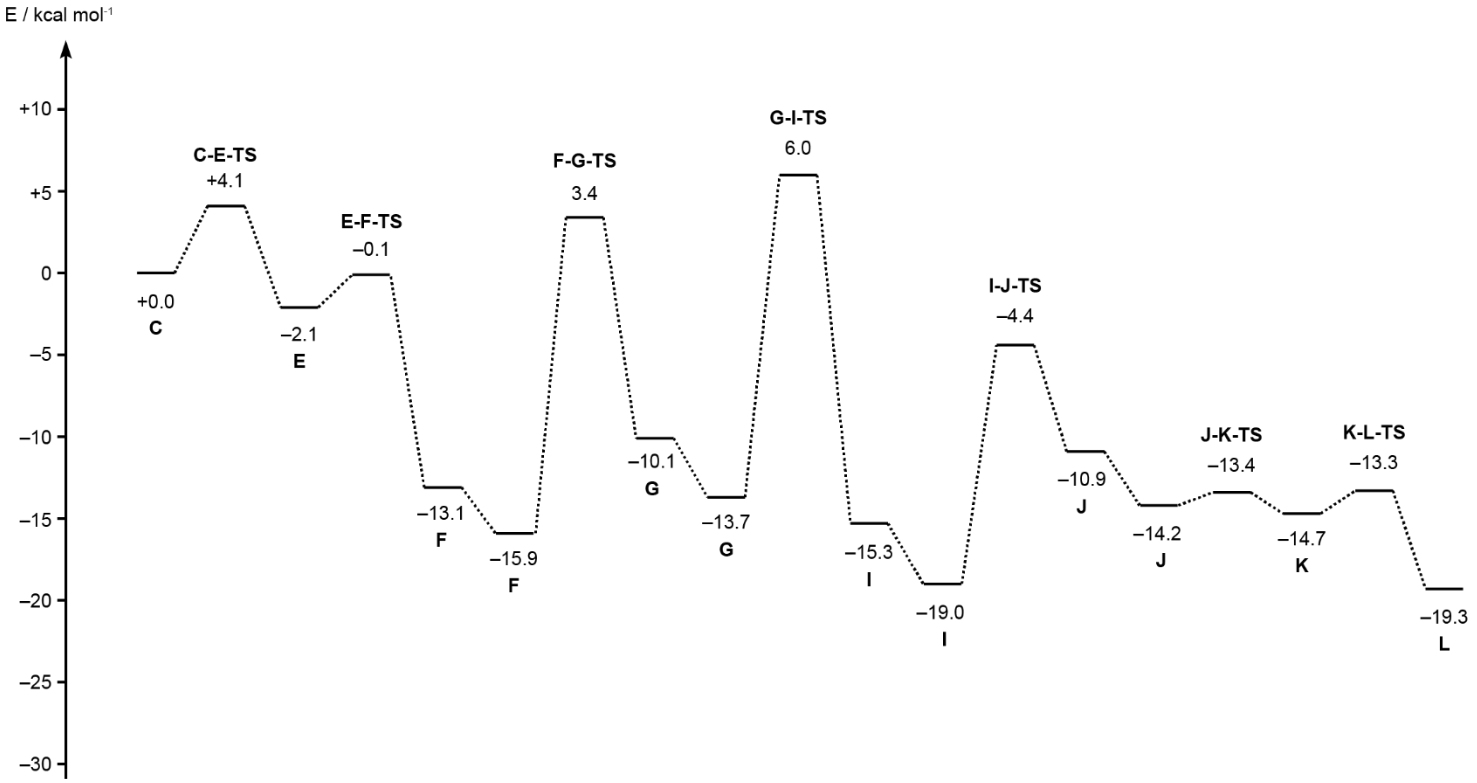

Figure S91. Computed energy profile for the cyclisation cascade from FPP to **6**, **9**, **13** and **15** (Scheme 2B of main text, wB97M-V/Def2-TZVPPD//B97D3/6-31G(d,p), 298 K).

Table S11. Results of DFT calculations (wB97M-V/Def2-TZVPPD//B97D3/6-31G(d,p)) for the cyclisation cascade from FPP to **12** (Scheme 3 of main text).

Structure[a]	Gibbs energy (298.15K) in Hartree	energy relative to C' in kcal/mol	free energy of activation / kcal/mol	free reaction energy / kcal/mol
**C'**	–585.946860	0.0		
**C'-D'-TS**	–585.934255	7.9	7.9	
**D'**	–585.939871	4.4		4.4
**D'**	–585.935890	6.9		
**D'-E'-TS**	–585.926062	13.1	6.2	
**E'**	–585.938292	5.4		–1.5
**E'**	–585.939868	4.4		
**E'-F'-TS**	–585.935382	7.2	2.8	
**F'**	–585.946063	0.5		–3.9
**F'**	–585.946058	0.5		
**F'-G'-TS**	–585.941274	3.5	3.0	
**G'**	–585.963589	–10.5		–11.0
**G'**	–585.968107	–13.3		
**G'-I'-TS**	–585.927982	11.8	25.2	
**I'**	–585.979207	–20.3		–7.0
**I'**	–585.978674	–20.0		
**I'-J'-TS**	–585.958551	–7.3	12.6	
**J'**	–585.963490	–10.4		9.5
**J'**	–585.971196	–15.3		
**J'-M'-TS**	–585.967139	–12.7	2.5	
**M'**	–585.963209	–10.3		5.0
**M'**	–585.963210	–10.3		
**M'-N'-TS**	–585.954616	–4.9	5.4	
**N'**	–585.967430	–12.9		–2.6
**N'**	–585.967416	–12.9		
**N'-O'-TS**	–585.968525	–13.6	–0.7	
**O'**	–585.970157	–14.6		–1.7

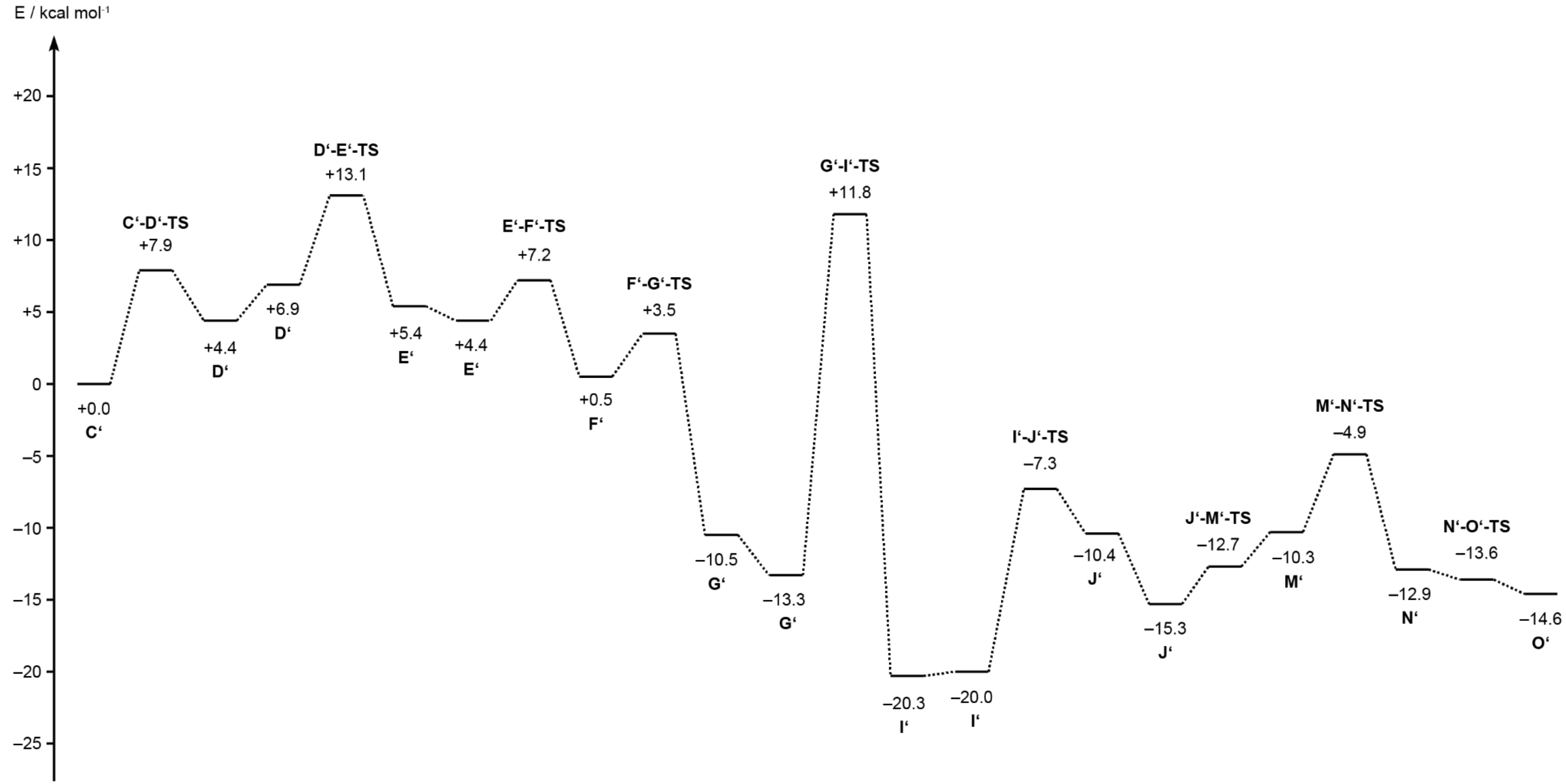

Figure S92. Computed energy profile for the cyclisation cascade from FPP to **12** (Scheme 3 of main text, wB97M-V/Def2-TZVPPD//B97D3/6-31G(d,p), 298 K).

Table S12. Results of DFT calculations (wB97M-V/Def2-TZVPPD//B97D3/6-31G(d,p)) for the cyclisation cascade from **J** to **14** (Scheme 4 of main text; the steps until **J** are the same as in Scheme 2, the step **J**-**M** is the same as **J'**-**M'** in Scheme 3, only with different Me12/Me13 labelling).

Structure[a]	Gibbs energy (298.15K) in Hartree	energy relative to C in kcal/mol	free energy of activation / kcal/mol	free reaction energy / kcal/mol
**J**	–585.971196	–15.3		
**J-M-TS**	–585.967139	–12.7	2.5	
**M**	–585.963209	–10.3		5.0
**M**	–585.967219	–12.8		
**M-P-TS**	–585.948664	–1.1	11.6	
**P**	–585.963761	–10.6		2.2
**P**	–585.963393	–10.4		
**P-Q-TS**	–585.967384	–12.9	–2.5	
**Q**	–585.975369	–17.9		–7.5
**Q**	–585.975812	–18.2		
**Q-R-TS**	–585.973480	–16.7	1.5	
**R**	–585.977649	–19.3		–1.2

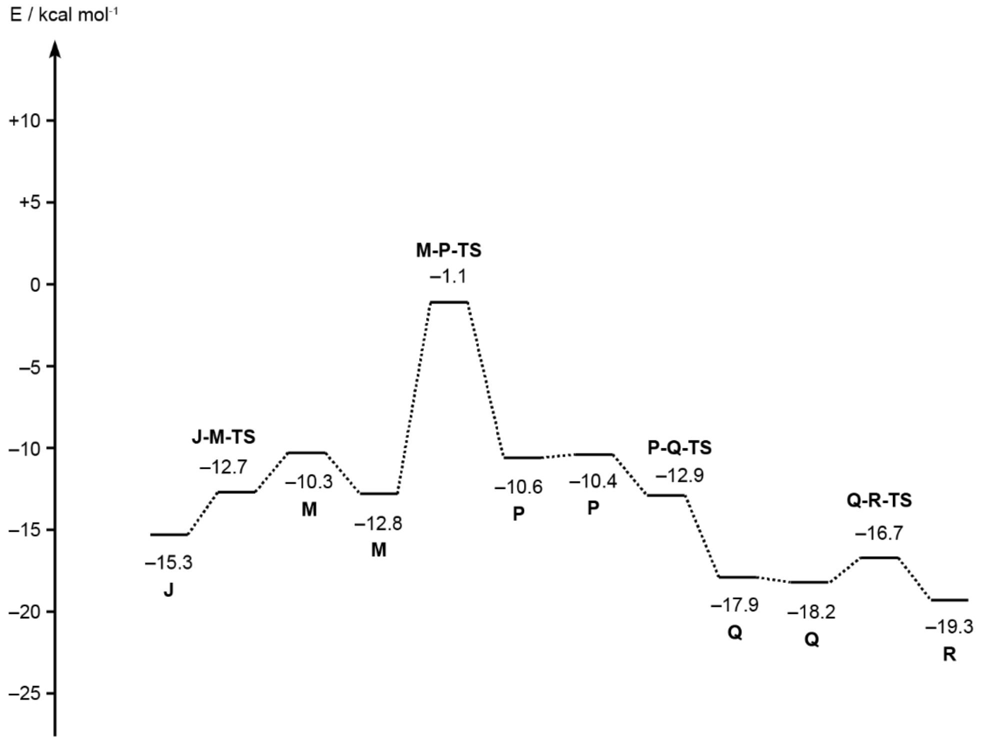

Figure S93. Computed energy profile for the cyclisation cascade from **J** to **14** (Scheme 4 of main text, wB97M-V/Def2-TZVPPD//B97D3/6-31G(d,p), 298 K).

Development of the mechanism for the biosynthesis of 12

The subsequent discussion is a summary of mechanistic hypotheses that were considered as alternatives to the mechanism for the biosynthesis of **12** presented in Scheme 3 of the main text. As will be demonstrated, any of the mechanistic alternatives shown here is excluded, either because it does not fit with the results from isotopic labelling experiments, or a key step has a reaction barrier that is way too high to be realistic for an enzyme catalysed reaction at 30 °C. The mechanism presented in the main text, although it has one step with a comparably high reaction barrier of 25.2 kcal/mol, is in line with all labelling experiments and is according to the computational results the best possible option that was found. Before the detailed mechanistic alternatives are discussed (Schemes S6 – S23), a few general considerations are explained in Schemes S2 – S5.

1. The terminal deprotonation to **12**

In the terminal deprotonation to **12** information about the configuration at C7 in the direct precursor is lost. Therefore, both possibilities must be considered for the late-stage intermediates **J**/**L** (Schemes S6 – S21). This traces back to different starting conformations of FPP with respect to the orientation of the plane of the olefinic C6=C7 double bond.

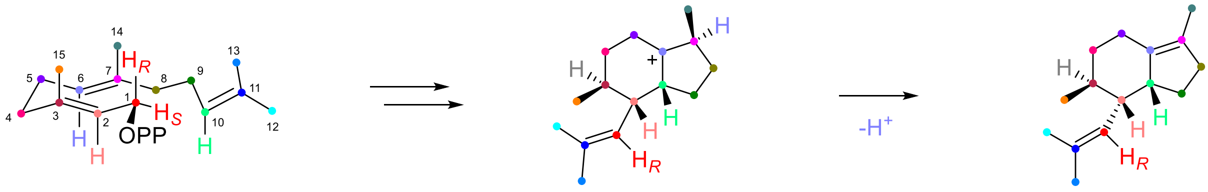

FPP | **L** | (-)-**12**

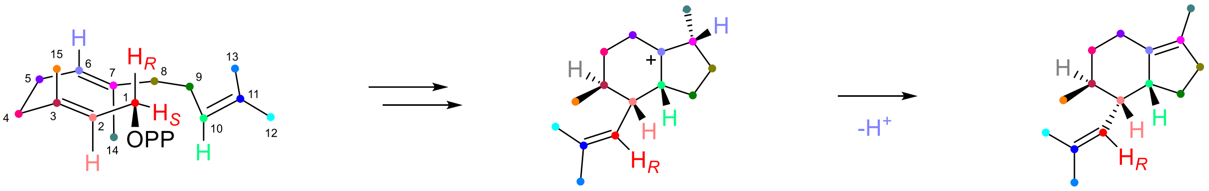

FPP | **L** | (-)-**12**

Scheme S2. The terminal deprotonation to **12**.

2. The reprotonation of a neutral intermediate at C3

Bicyclogermacrene is observed as an enzyme product of BgPgS and it can logically be considered as a neutral intermediate to other enzyme products. Its stereoisomer isolepidozene, although not being detected among the enzyme products, may also play a role. In all enzyme products including **12** a 3*S* configuration is observed, which requires an orientation of the C2=C3 olefinic plane with H2 (light red) pointing down and Me15 (orange) pointing up. Theoretically, two more possibilities may arise, if NPP is considered as an intermediate, which could lead to neutral intermediates with a 2*Z* configured double bond, i. e. isobicyclogermacrene and lepidozene. In these cases, H2 and Me15 may both point up. An argument against the latter two possibilities is the observation that both enantiomers of NPP are only sluggishly converted by BgPgS (Figure S94, next page). Further stereoisomers of the neutral intermediate are excluded by the fact that the 1-*pro*-*S* hydrogen is lost in the deprotonation with cyclopropanation to the neutral intermediate.

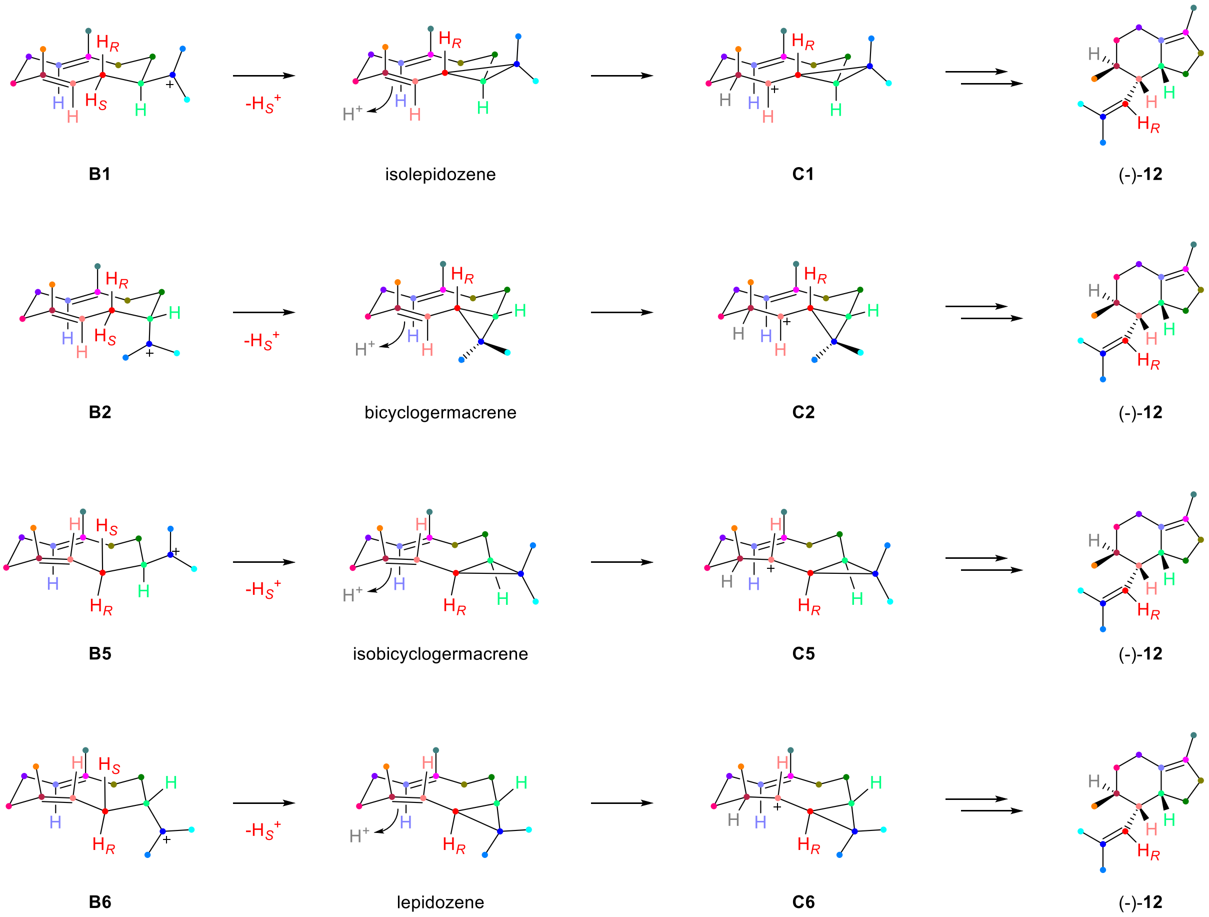

Scheme S3. The reprotonation of a neutral intermediate at C3.

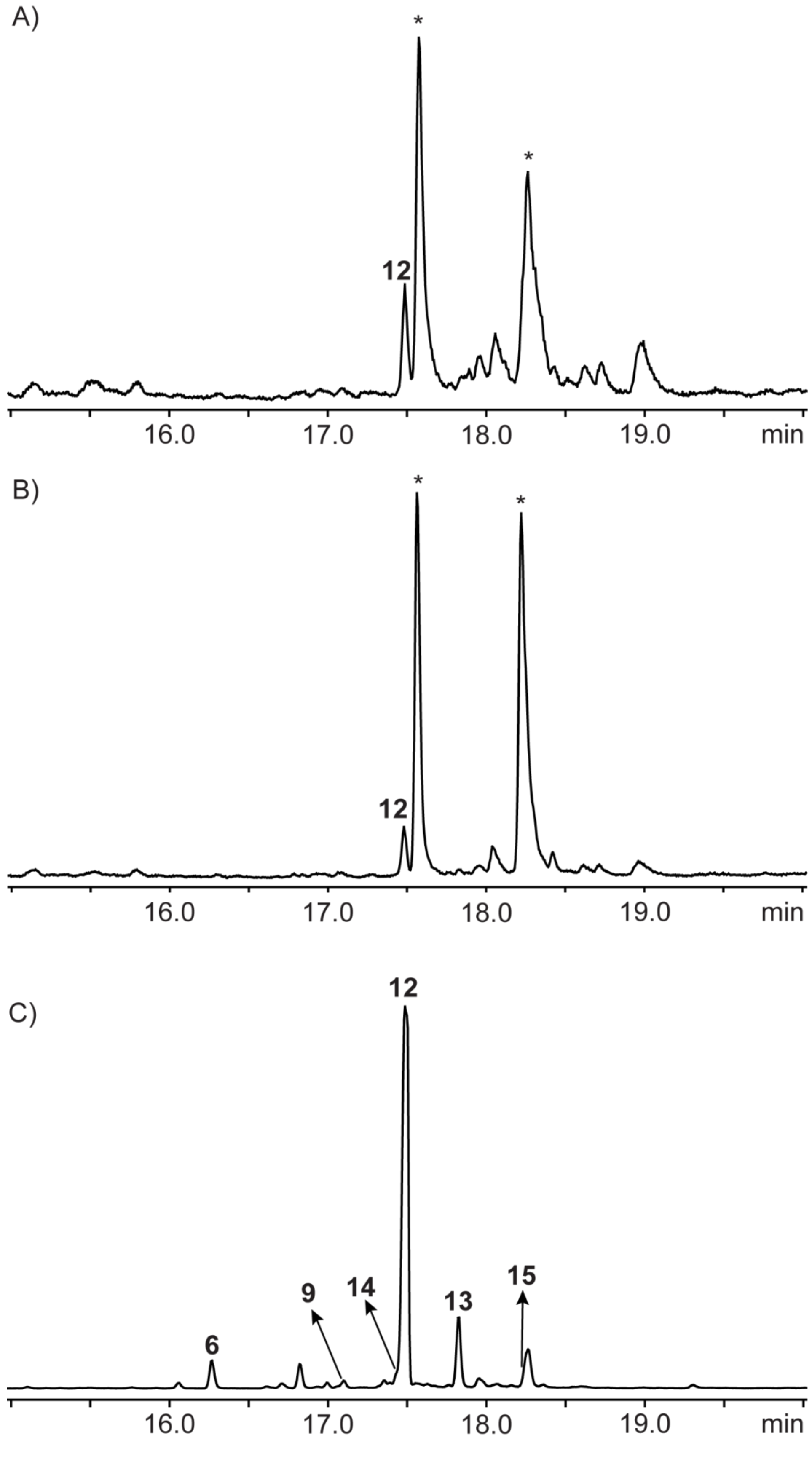

Figure S94. Total ion chromatograms of extracts from enzyme incubations of A) (*R*)-NPP with BgPgS, B) (*S*)-NPP with BgPgS, and C) FPP with BgPgS.

3. The „break-flip-cyclise“ (B-F-C) sequence

In our development of a mechanistic model for the biosynthesis of **12** we realised that it is very difficult to explain how H2 can end up at C2 (light red) and H10 at C10 (light green), with the correct configurations being set at C2 and C10. As can be seen in the mechanistic alternatives discussed below, either a key rearrangement step or a subsequent 1,3-hydride shift shows a too high reaction barrier to be realised for an enzymatic reaction at 30 °C, or the wrong configurations at C2 and/or C10 are obtained, or the positions of H2 and H10 are exchanged, contradicting the results of labelling experiments. We considered the situation may be resolved through the following “break-flip-cyclise” (B-F-C) sequence through which the configurations at C2 and/or C10 can be inverted. As a consequence, neither the configuration at C2 nor at C10 in the late-stage intermediates **J**/**L** (Schemes S6 – S21) is known, which allows for an orientation of H2 up (cyclisation of FPP to the (*E*,*E*)-germacradienyl cation, **B1** or **B2** in Scheme S3) or down (cyclisation of FPP via NPP to the (*Z*,*E*)-germacradienyl cation, **B5** or **B6** in Scheme S3) in the initial pathway intermediates. Analogously, H10 may point up or down, which is explainable through different orientations of the olefinic C10=C11 plane in the starting conformation of FPP.

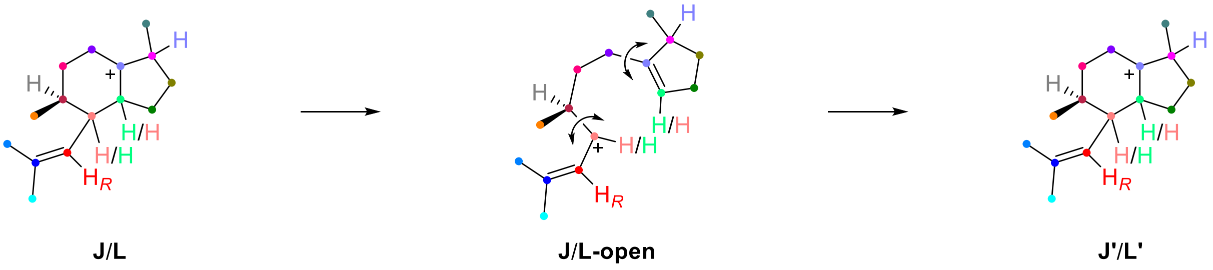

Scheme S4. The “break-flip-cyclise” (B-F-C) sequence.

4. Configurational inversion at C1 in **G**

The intermediates **G** do not show a fully closed cyclopropane ring. As exemplified for the stereoisomer **G1**, the ring can fully open to **M1** in which a rotation of the dimethylvinyl group is possible. This can be followed by partial ring closure to **N1**, which leads to another series of possible stereoisomers for subsequent steps.

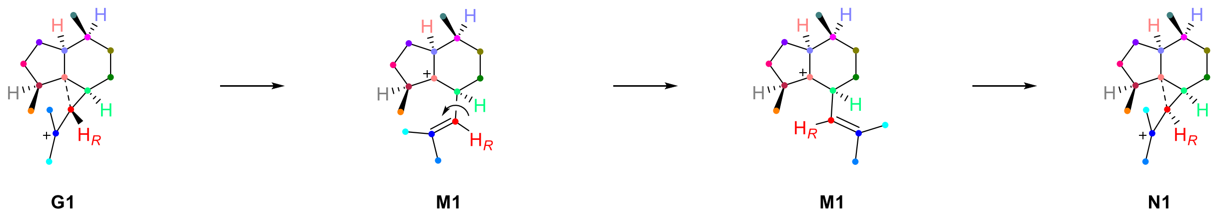

Scheme S5. The configurational inversion at C1 in **G**, exemplified for the stereoisomer **G1**.

5. Mechanistic alternative I

This mechanism starts with a conformation of FPP in which H2, H6 and H10 are oriented down (2D-6D-10D). In intermediate **C1** H1$_R$ (red) and C13 (dark blue) are *cis*, which matches the situation in the product **12**. DFT computations show a comparably low reaction barrier of 16.5 kcal/mol for the rearrangement to **I1** with skipping of **H1**, but the subsequent 1,3-hydride shift to **J1** is impossible (reaction barrier: 58.4 kcal/mol). A sequence of two 1,2-hydride shifts will have much lower reaction barriers, but this will lead to the wrong positions for H2 and H10. Conclusively, mechanistic alternative I cannot explain the formation of **12**. The B-F-C mechanism (Scheme S4) explains required configurational inversions in **J1**/**L1**.

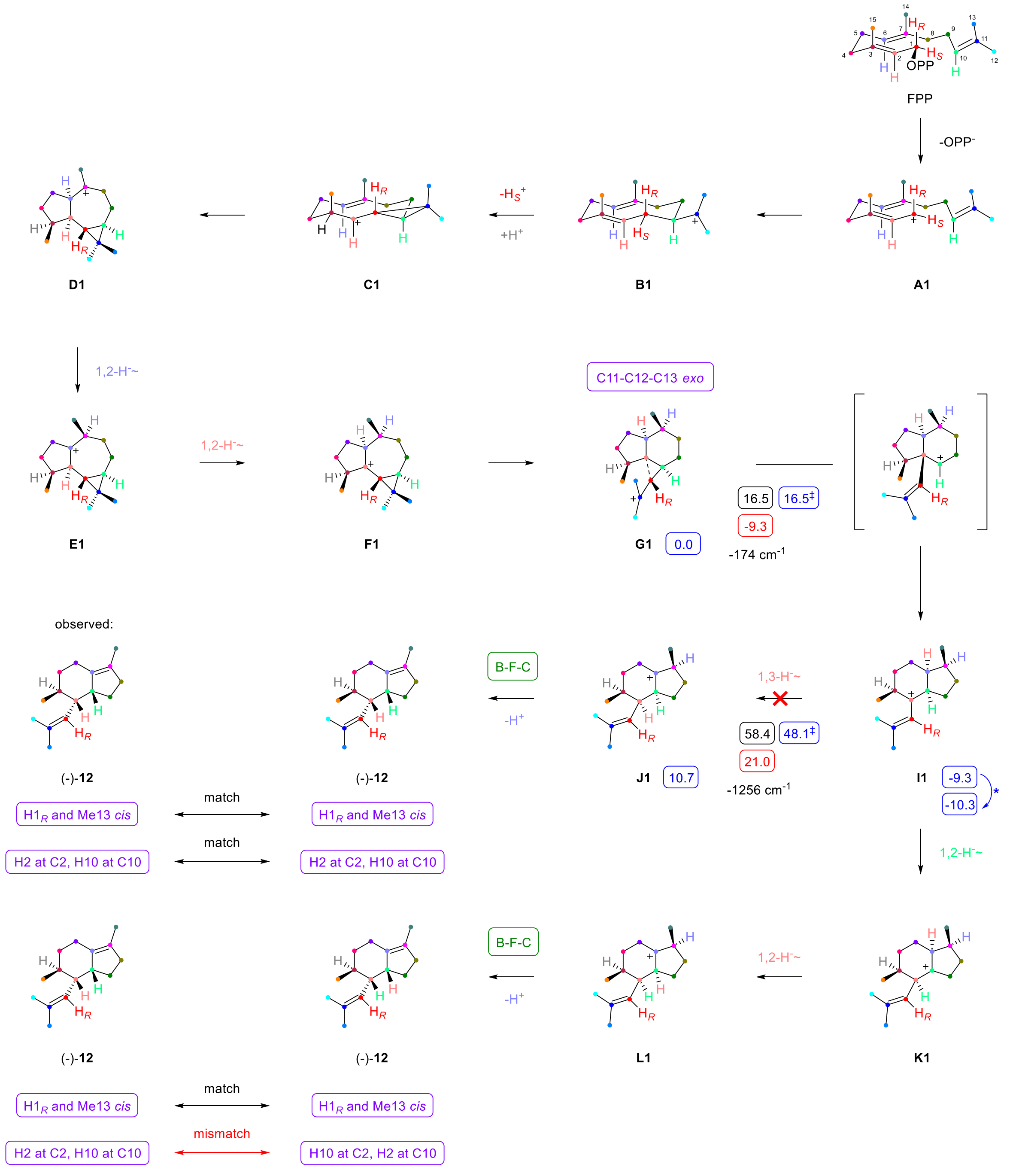

Scheme S6. Mechanism for the biosynthesis of **12** starting from FPP with H2, H6 and H10 down (2D-6D-10D).

6. Mechanistic alternative II

This mechanism is a modification of mechanistic alternative I with same steps until **G1** (Scheme S6) and starts with a conformation of FPP in which H2, H6 and H10 are oriented down (2D-6D-10D). In intermediate **C1** $H1_R$ (red) and C13 (dark blue) are *cis*, which matches the situation in the product **12**. In **G1** a configurational inversion according to the mechanism of Scheme S5 may happen, leading to **N1**. For the rearrangement of **N1** with C11-C12-C13 in *endo* orientation no transition state could be localised through DFT computations, and also the subsequent 1,3-hydride shift to **J1** is impossible (reaction barrier: 58.4 kcal/mol). A sequence of two 1,2-hydride shifts will have much lower reaction barriers, but this will lead to the wrong positions for H2 and H10. Conclusively, mechanistic alternative II cannot explain the formation of **12**. The B-F-C mechanism (Scheme S4) explains required configurational inversions in **J1**/**L1**.

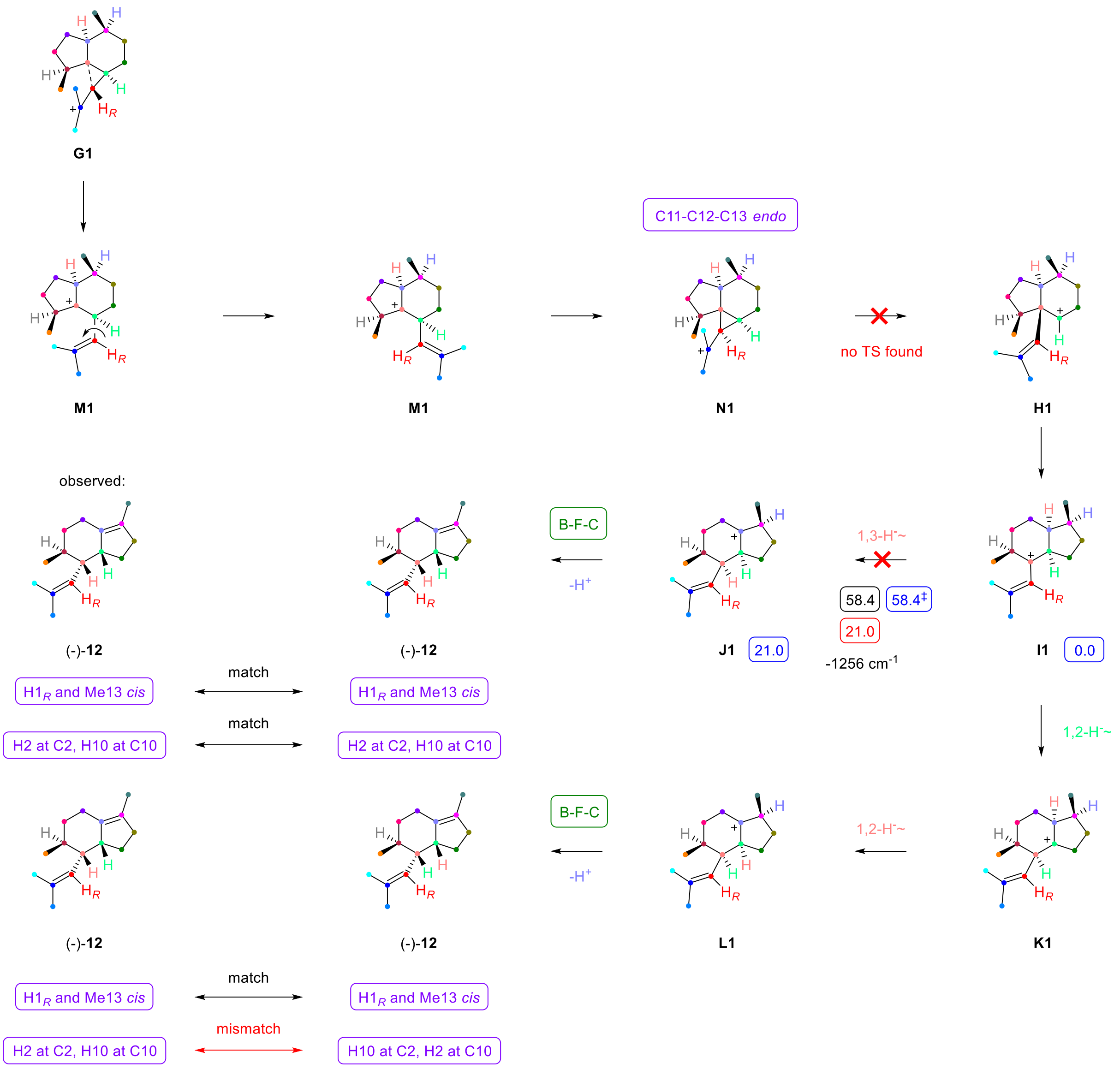

Scheme S7. Mechanism for the biosynthesis of **12** starting from FPP with H2, H6 and H10 down (2D-6D-10D) and configurational change in **G1**.

7. Mechanistic alternative III

This mechanism starts with a conformation of FPP in which H2 and H6 are oriented down and H10 is oriented up (2D-6D-10U). In intermediate **C2** H1$_R$ (red) and C12 (light blue) are *cis*, which mismatches the situation in the product **12**. For the rearrangement of **G2** with C11-C12-C13 in *endo* orientation no transition state could be localised through DFT computations. The subsequent 1,3-hydride shift to **J2** is possible (reaction barrier: 12.7 kcal/mol) and can explain the correct positioning of H2 and H10. A sequence of two 1,2-hydride shifts will lead to the wrong positions for H2 and H10. Because of the mismatching situation for the relative orientation of H1$_R$ with respect to Me12/Me13, mechanistic alternative III cannot explain the formation of **12**. The B-F-C mechanism (Scheme S4) explains required configurational inversions in **J2**/**L2**.

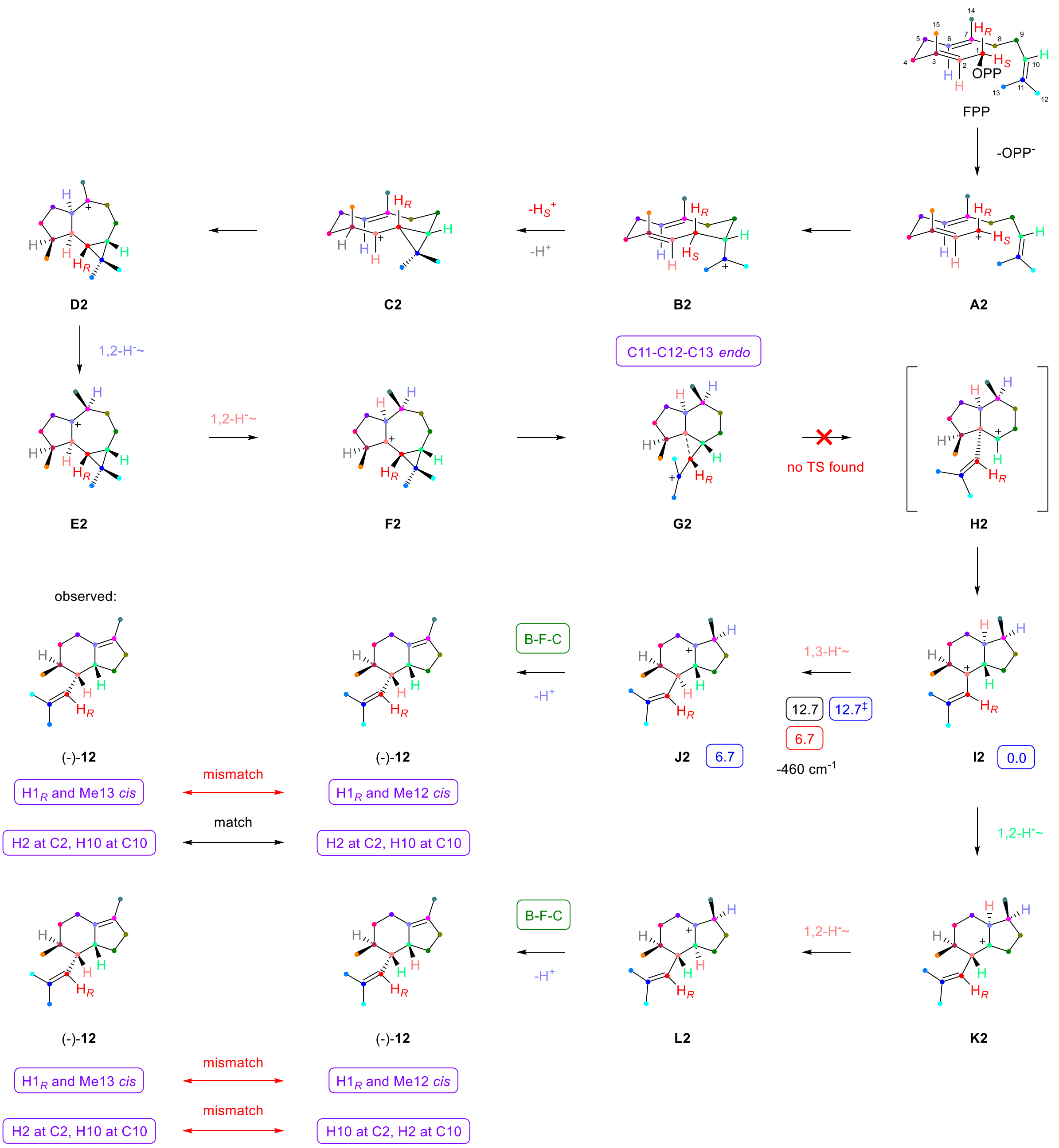

Scheme S8. Mechanism for the biosynthesis of **12** starting from FPP with H2 and H6 down and H10 up (2D-6D-10U).

8. Mechanistic alternative IV

This mechanism is a modification of mechanistic alternative III with same steps until **G2** (Scheme S8) and starts with a conformation of FPP in which H2 and H6 are oriented down and H10 is oriented up (2D-6D-10D). In intermediate **C2** $H1_R$ (red) and C12 (light blue) are *cis*, which mismatches the situation in the product **12**. In **G2** a configurational inversion according to the mechanism of Scheme S5 may happen, leading to **N2**. DFT computations show a comparably low reaction barrier of 18.1 kcal/mol for the rearrangement to **I2** with **H2** representing the transition state. The subsequent 1,3-hydride shift to **J2** is possible (reaction barrier: 12.7 kcal/mol). A sequence of two 1,2-hydride shifts will lead to the wrong positions for H2 and H10. Because of the mismatching situation for the relative orientation of $H1_R$ with respect to Me12/Me13, mechanistic alternative IV cannot explain the formation of **12**. The B-F-C mechanism (Scheme S4) explains required configurational inversions in **J2**/**L2**.

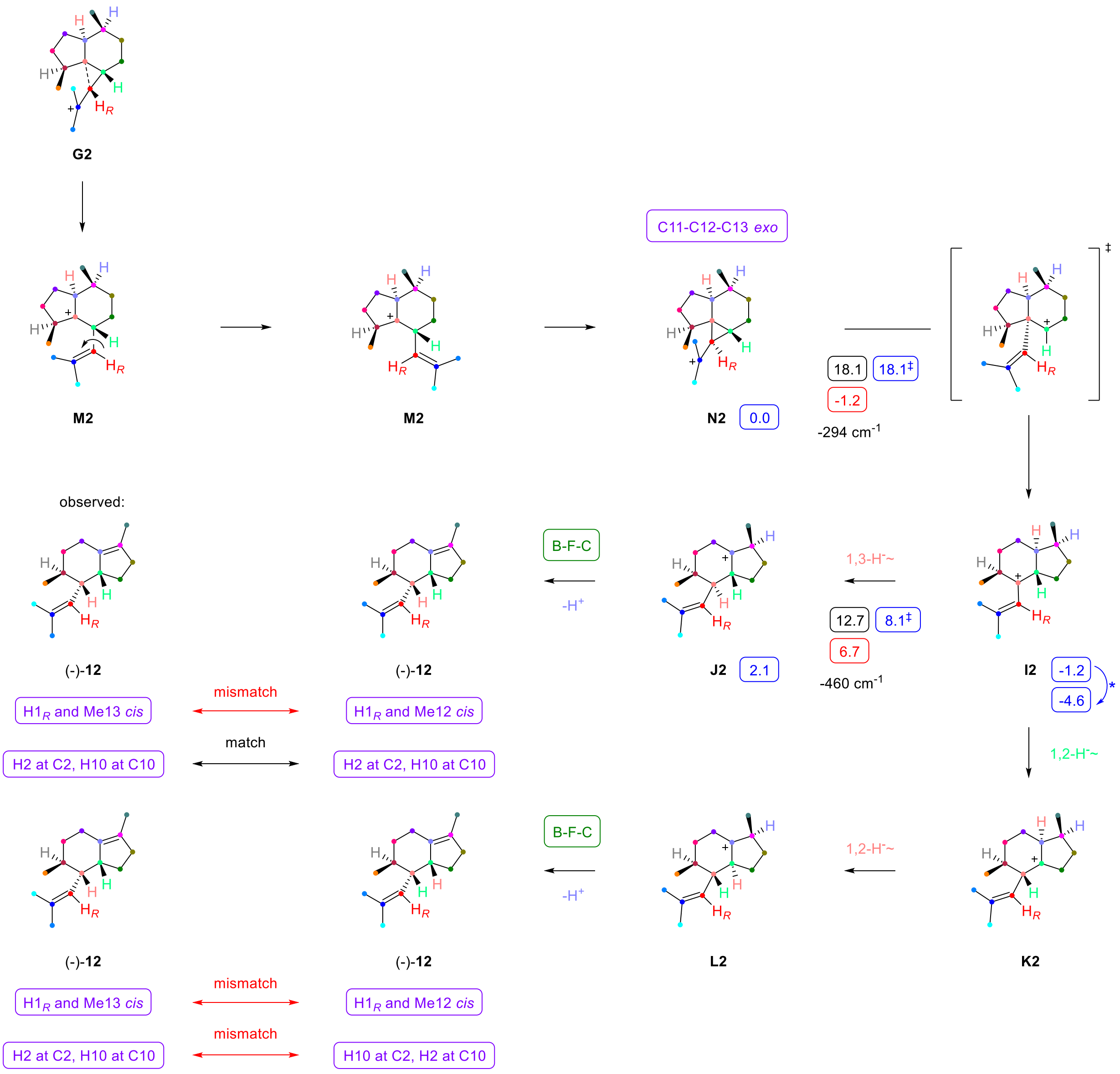

Scheme S9. Mechanism for the biosynthesis of **12** starting from FPP with H2 and H6 down and H10 up (2D-6D-10U) and configurational change in **G1**.

9. Mechanistic alternative V

This mechanism starts with a conformation of FPP in which H2 and H10 are oriented down and H6 is oriented up (2D-6U-10D). In intermediate **C3** H1$_R$ (red) and C13 (dark blue) are *cis*, which matches the situation in the product **12**. DFT computations show a comparably low reaction barrier of 16.1 kcal/mol for the rearrangement to **I3** with skipping of **H3**, but the subsequent 1,3-hydride shift to **J3** is impossible (reaction barrier: 59.8 kcal/mol). A sequence of two 1,2-hydride shifts will have lower reaction barriers, but will lead to the wrong positions for H2 and H10. Conclusively, mechanistic alternative V cannot explain the formation of **12**. The B-F-C mechanism (Scheme S4) explains required configurational inversions in **J3**/**L3**.

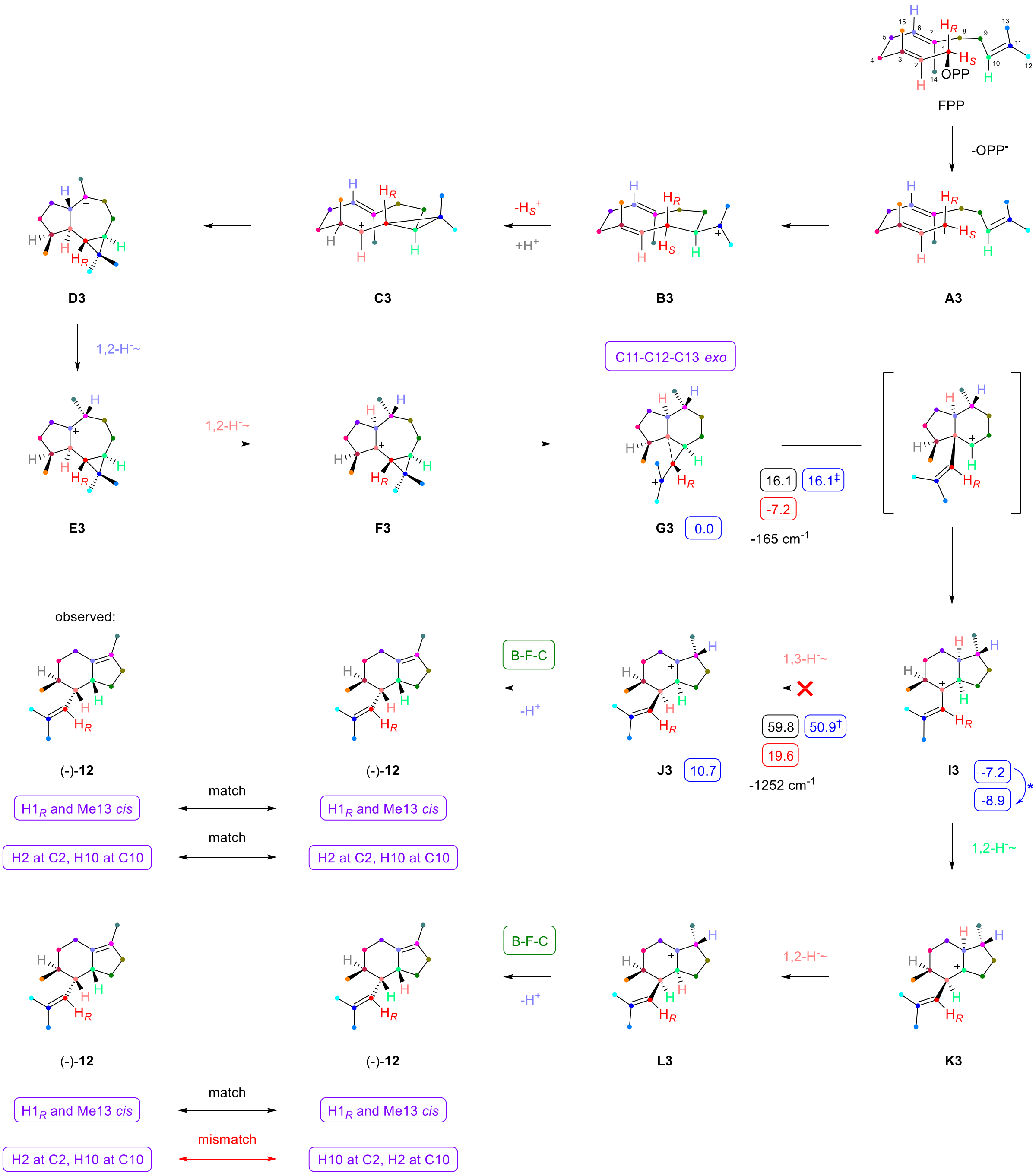

Scheme S10. Mechanism for the biosynthesis of **12** starting from FPP with H2 and H10 down and H6 up (2D-6U-10D).

10. Mechanistic alternative VI

This mechanism is a modification of mechanistic alternative V with same steps until **G3** (Scheme S10) and starts with a conformation of FPP in which H2 and H10 are oriented down and H6 is oriented up (2D-6U-10D). In intermediate **C3** H1$_R$ (red) and C13 (dark blue) are *cis*, which matches the situation in the product **12**. In **G3** a configurational inversion according to the mechanism of Scheme S5 may happen, leading to **N3**. For the rearrangement of **N3** with C11-C12-C13 in *endo* orientation no transition state could be localised through DFT computations, and also the subsequent 1,3-hydride shift to **J3** is impossible (reaction barrier: 59.8 kcal/mol). A sequence of two 1,2-hydride shifts will have much lower reaction barriers, but this will lead to the wrong positions for H2 and H10. Conclusively, mechanistic alternative VI cannot explain the formation of **12**. The B-F-C mechanism (Scheme S4) explains required configurational inversions in **J3**/**L3**.

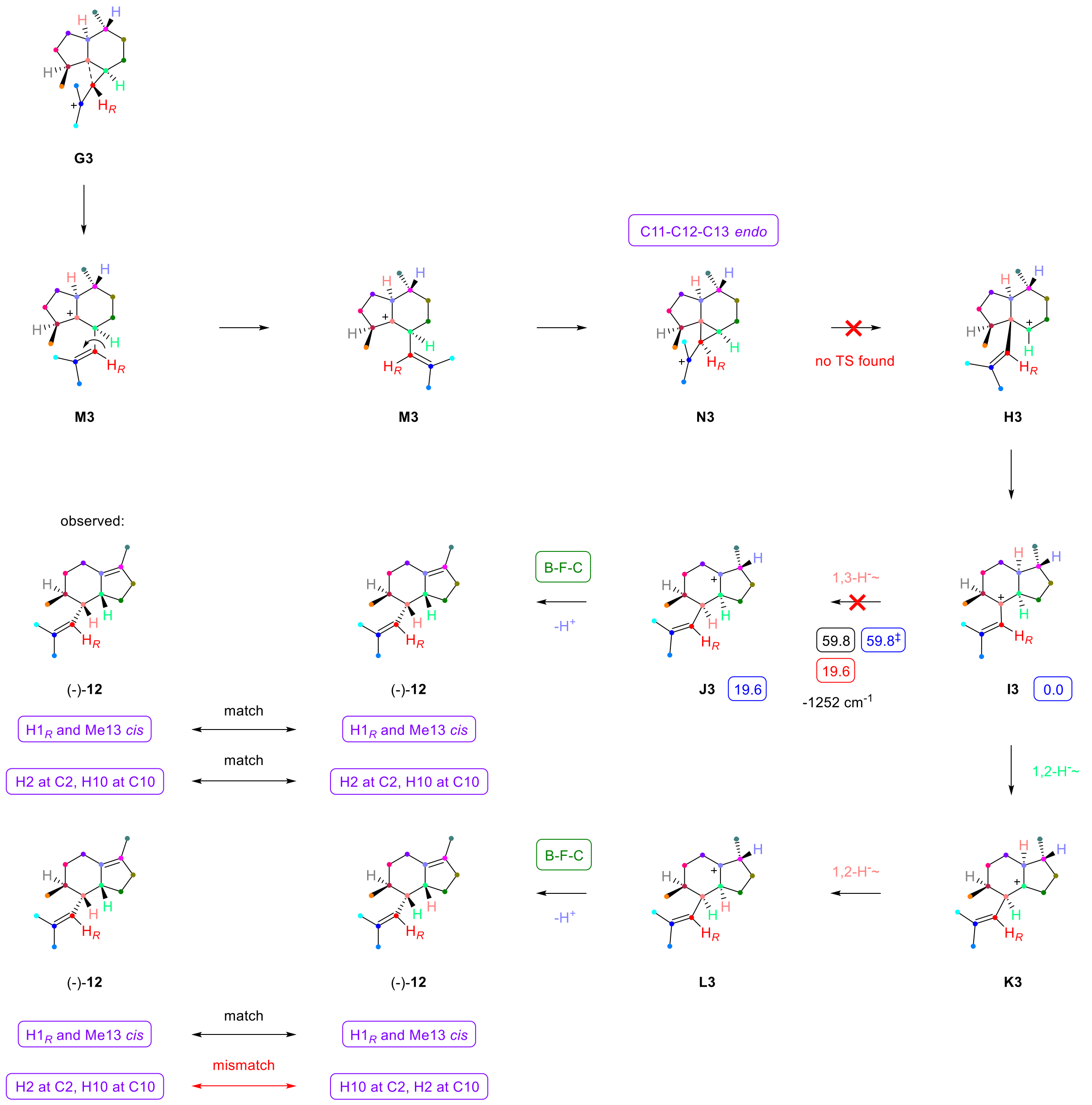

Scheme S11. Mechanism for the biosynthesis of **12** starting from FPP with H2 and H10 down and H6 up (2D-6U-10D) and configurational change in **G3**.

11. Mechanistic alternative VII

This mechanism starts with a conformation of FPP in which H2 is oriented down and H6 and H10 are oriented up (2D-6U-10U). In intermediate **C4** H1$_R$ (red) and C12 (light blue) are *cis*, which mismatches the situation in the product **12**. DFT computations show a reaction barrier of 21.2 kcal/mol for the rearrangement to **I4** with skipped **H4**. The subsequent 1,3-hydride shift to **J4** is possible (reaction barrier: 12.9 kcal/mol) and can explain the correct positioning of H2 and H10. A sequence of two 1,2-hydride shifts will lead to the wrong positions for H2 and H10. Because of the mismatching situation for the relative orientation of H1$_R$ with respect to Me12/Me13, mechanistic alternative VII cannot explain the formation of **12**. The B-F-C mechanism (Scheme S4) explains required configurational inversions in **J4**/**L4**.

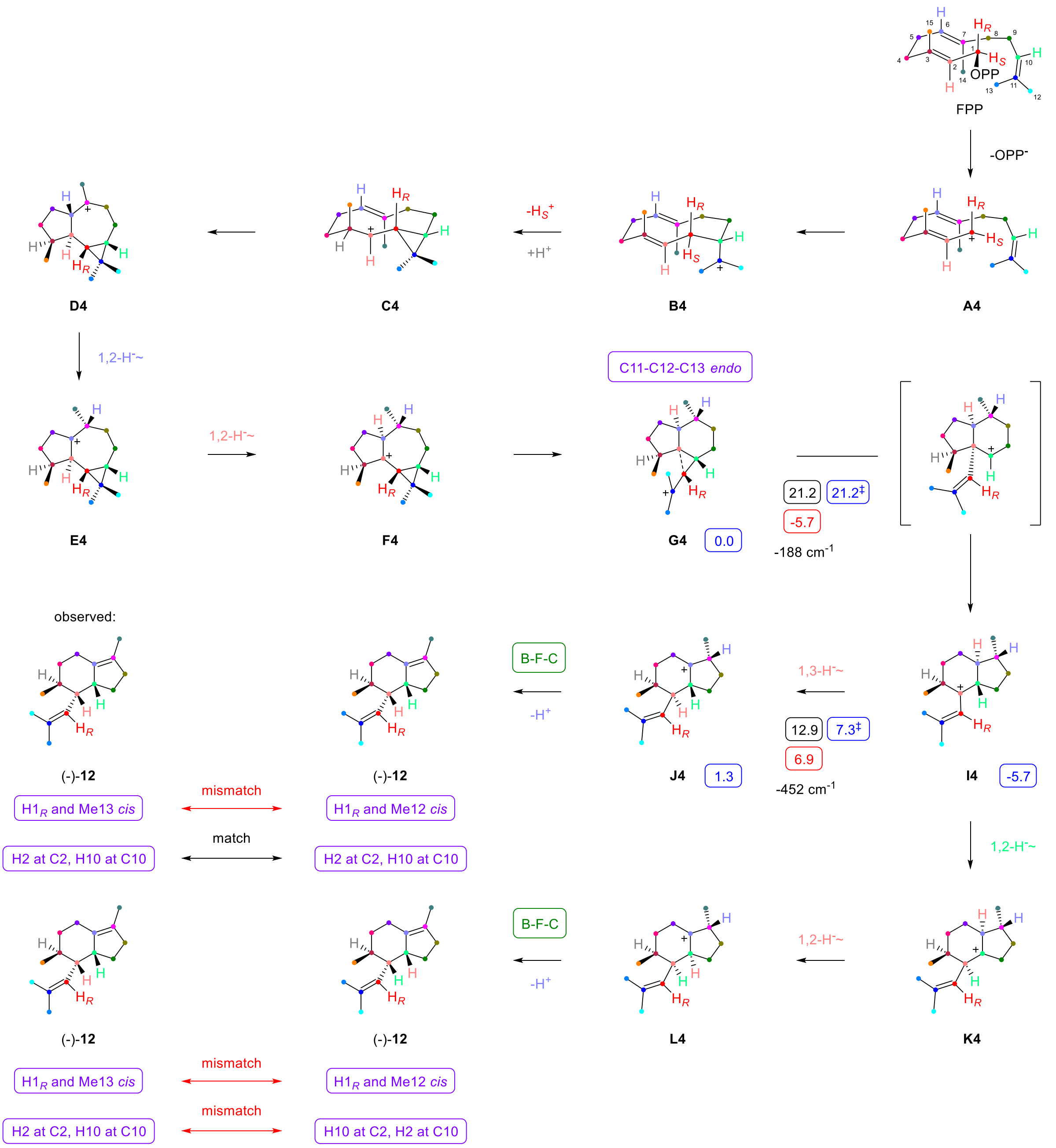

Scheme S12. Mechanism for the biosynthesis of **12** starting from FPP with H2 down and H6 and H10 up (2D-6U-10U).

12. Mechanistic alternative VIII

This mechanism is a modification of mechanistic alternative VII with same steps until **G4** (Scheme S12) and starts with a conformation of FPP in which H2 is oriented down and H6 and H10 are oriented up (2D-6U-10U). In intermediate **C4** H1$_R$ (red) and C12 (light blue) are *cis*, which mismatches the situation in the product **12**. In **G4** a configurational inversion according to the mechanism of Scheme S5 may happen, leading to **N4**. DFT computations show a reaction barrier of 20.4 kcal/mol for the rearrangement to **I4** with **H4** localised as intermediate. The subsequent 1,3-hydride shift to **J4** is possible (reaction barrier: 12.9 kcal/mol). A sequence of two 1,2-hydride shifts will lead to the wrong positions for H2 and H10. Because of the mismatching situation for the relative orientation of H1$_R$ with respect to Me12/Me13, mechanistic alternative VIII cannot explain the formation of **12**. The B-F-C mechanism (Scheme S4) explains required configurational inversions in **J4**/**L4**.

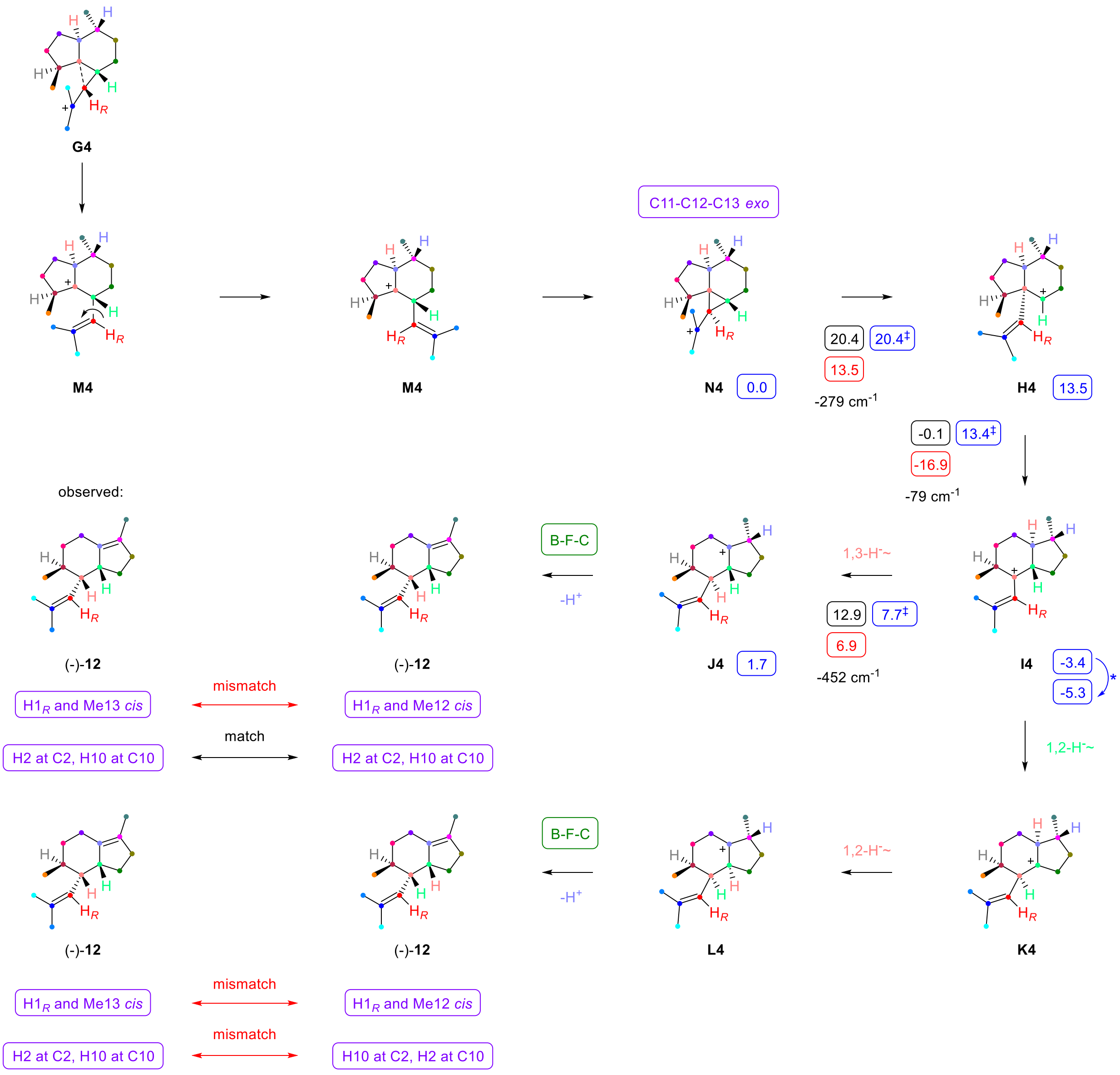

Scheme S13. Mechanism for the biosynthesis of **12** starting from FPP with H2 down and H6 and H10 up (2D-6U-10U) and configurational change in **G4**.

13. Mechanistic alternative IX

This mechanism starts with a conformation of FPP with H6 and H10 down, and proceeding through NPP to turn H2 up (2U-6D-10D). In intermediate **C5** H1$_R$ (red) and C12 (light blue) are *cis*, which mismatches the situation in the product **12**. DFT computations show a comparably low reaction barrier of 13.8 kcal/mol for the rearrangement to **I6** with **H5** localised as intermediate. Due to a conformational change during the rearrangement **I6** with H10 up was obtained instead of the expected **I5** with H10 down. The subsequent 1,3-hydride shift to **J6** is impossible (reaction barrier: 60.7 kcal/mol). A sequence of two 1,2-hydride shifts will lead to the wrong positions for H2 and H10. Because of the mismatching situation for the relative orientation of H1$_R$ with respect to Me12/Me13, mechanistic alternative IX cannot explain the formation of **12**. The B-F-C mechanism (Scheme S4) explains a required configurational inversion in **L5**.

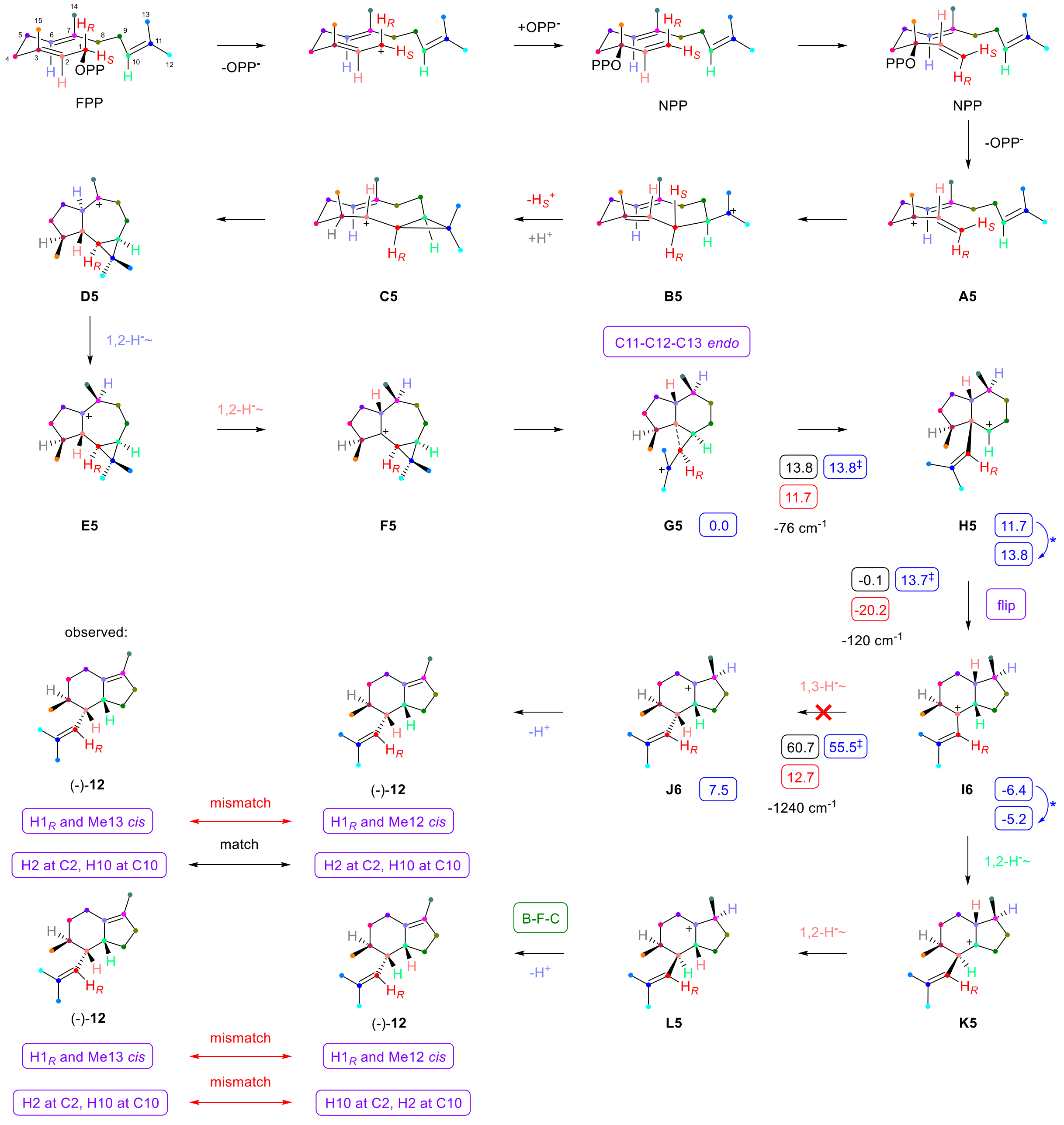

Scheme S14. Mechanism for the biosynthesis of **12** starting from FPP with H6 and H10 down, and proceeding through NPP to turn H2 up (2U-6D-10D).

14. Mechanistic alternative X

This mechanism is a modification of mechanistic alternative IX with same steps until **G5** (Scheme S14) and starts with a conformation of FPP with H6 and H10 down, and proceeding through NPP to turn H2 up (2U-6D-10D). In intermediate **C5** $H1_R$ (red) and C12 (light blue) are *cis*, which mismatches the situation in the product **12**. In **G5** a configurational inversion according to the mechanism of Scheme S5 may happen, leading to **N5**. DFT computations show a reaction barrier of 21.2 kcal/mol for the rearrangement to **I5** with **H5** localised as intermediate. The subsequent 1,3-hydride shift to **J5** is possible (reaction barrier: 12.2 kcal/mol). A sequence of two 1,2-hydride shifts will lead to the wrong positions for H2 and H10. Because of the mismatching situation for the relative orientation of $H1_R$ with respect to Me12/Me13, mechanistic alternative X cannot explain the formation of **12**. The B-F-C mechanism (Scheme S4) explains required configurational inversions in **J5**/**L5**.

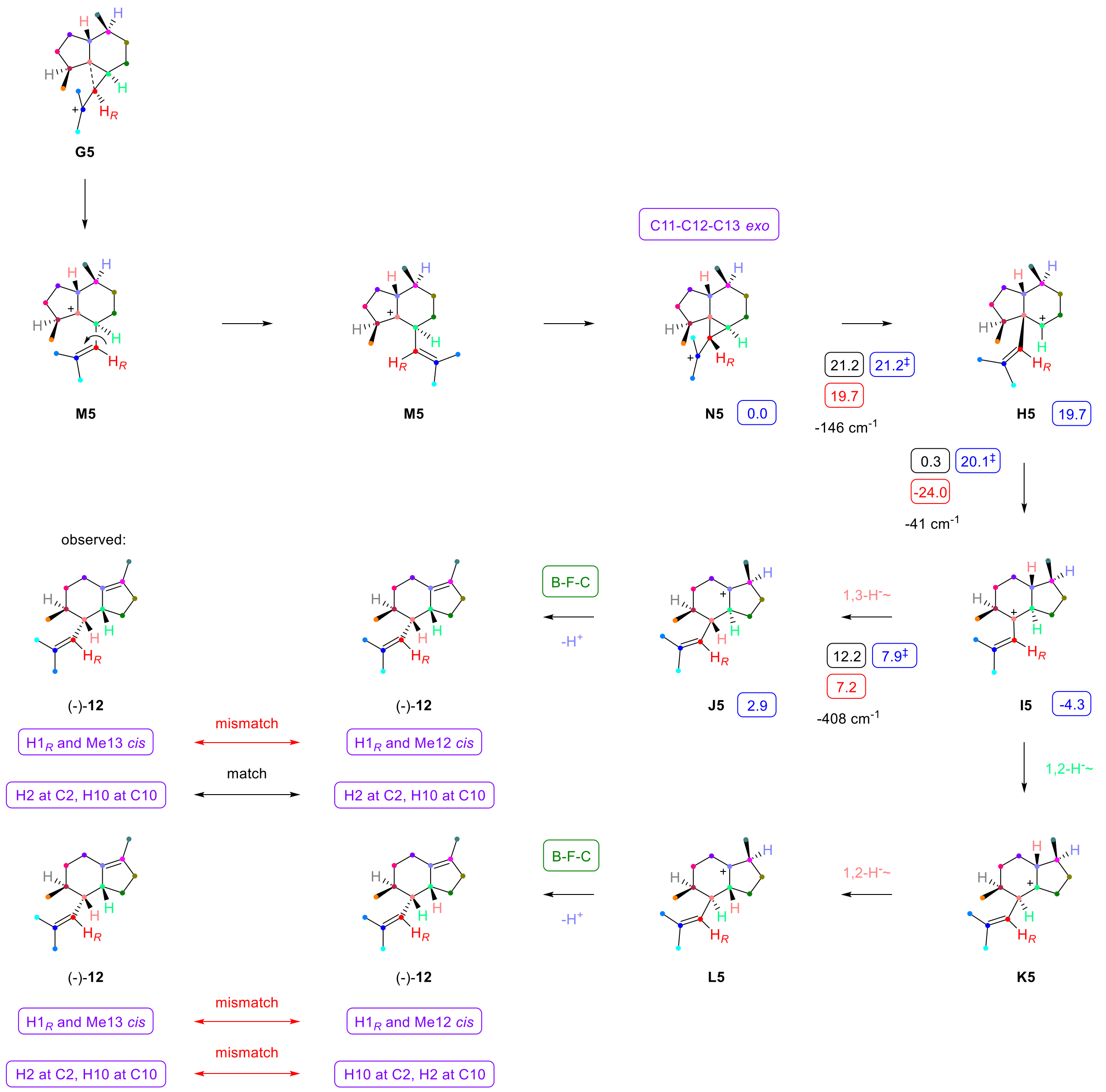

Scheme S15. Mechanism for the biosynthesis of **12** starting from FPP with H6 and H10 down, and proceeding through NPP to turn H2 up (2U-6D-10D) and configurational change in **G5**.

15. Mechanistic alternative XI

This mechanism starts with a conformation of FPP with H6 down and H10 up, and proceeding through NPP to turn H2 up (2U-6D-10U). In intermediate **C6** H1$_R$ (red) and C13 (dark blue) are *cis*, which matches the situation in the product **12**. DFT computations show a comparably low reaction barrier of 19.4 kcal/mol for the rearrangement to **I6** with skipping of **H6**, but the subsequent 1,3-hydride shift to **J3** is impossible (reaction barrier: 60.7 kcal/mol). A sequence of two 1,2-hydride shifts will have lower reaction barriers, but will lead to the wrong positions for H2 and H10. Conclusively, mechanistic alternative XI cannot explain the formation of **12**. Configurational inversions through the B-F-C mechanism (Scheme S4) are not required.

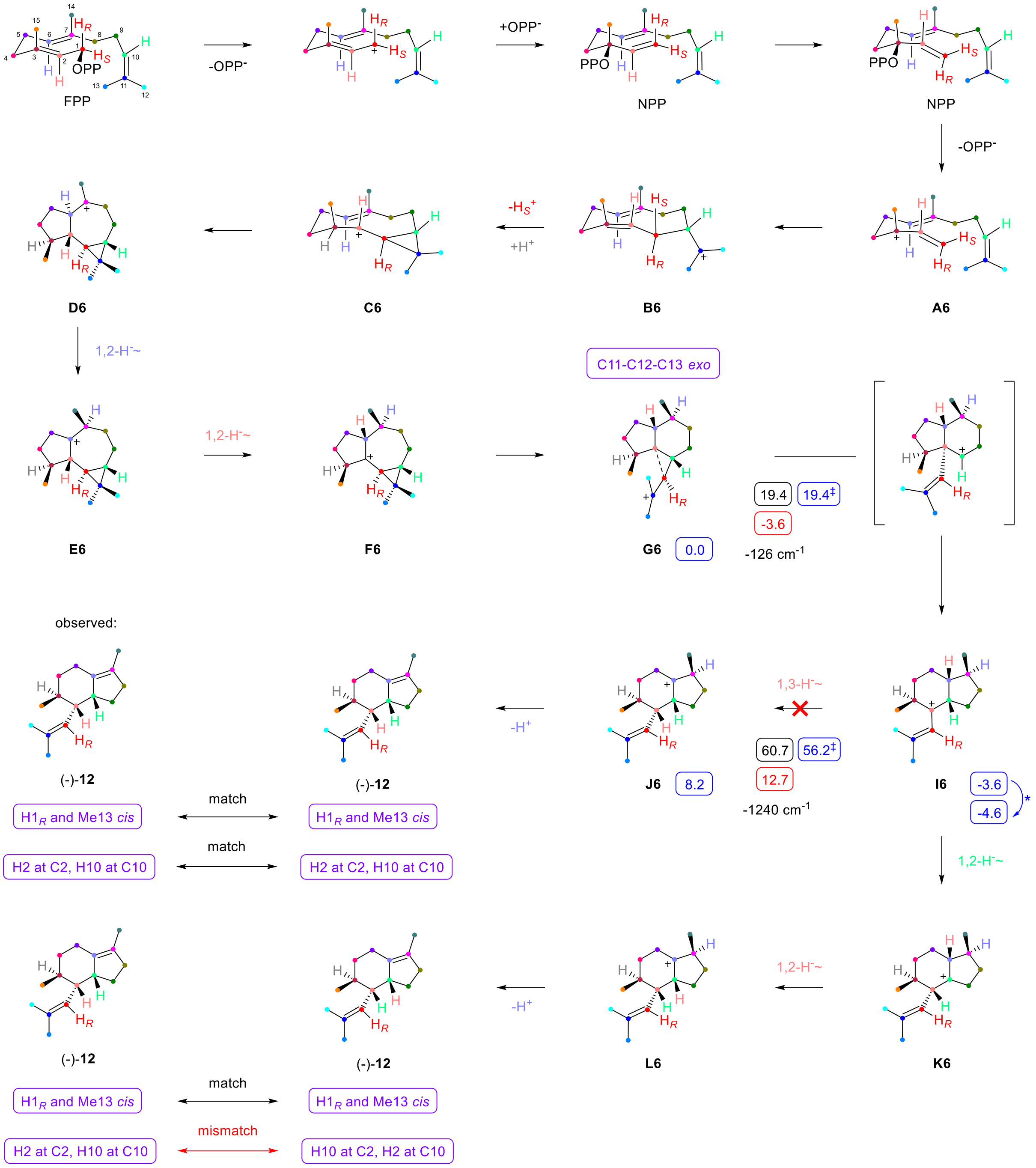

Scheme S16. Mechanism for the biosynthesis of **12** starting from FPP with H6 down and H10 up, and proceeding through NPP to turn H2 up (2U-6D-10U).

16. Mechanistic alternative XII

This mechanism is a modification of mechanistic alternative XI with same steps until **G6** (Scheme S16) and starts with a conformation of FPP with H6 down and H10 up, and proceeding through NPP to turn H2 up (2U-6D-10U). In intermediate **C6** H1*R* (red) and C13 (dark blue) are *cis*, which matches the situation in the product **12**. In **G6** a configurational inversion according to the mechanism of Scheme S5 may happen, leading to **N6**. For the rearrangement of **N6** with C11-C12-C13 in *endo* orientation no transition state could be localised through DFT computations, and also the subsequent 1,3-hydride shift to **J6** is impossible (reaction barrier: 60.7 kcal/mol). A sequence of two 1,2-hydride shifts will have much lower reaction barriers, but this will lead to the wrong positions for H2 and H10. Conclusively, mechanistic alternative XII cannot explain the formation of **12**. The B-F-C mechanism (Scheme S4) explains required configurational inversions in **J6**/**L6**.

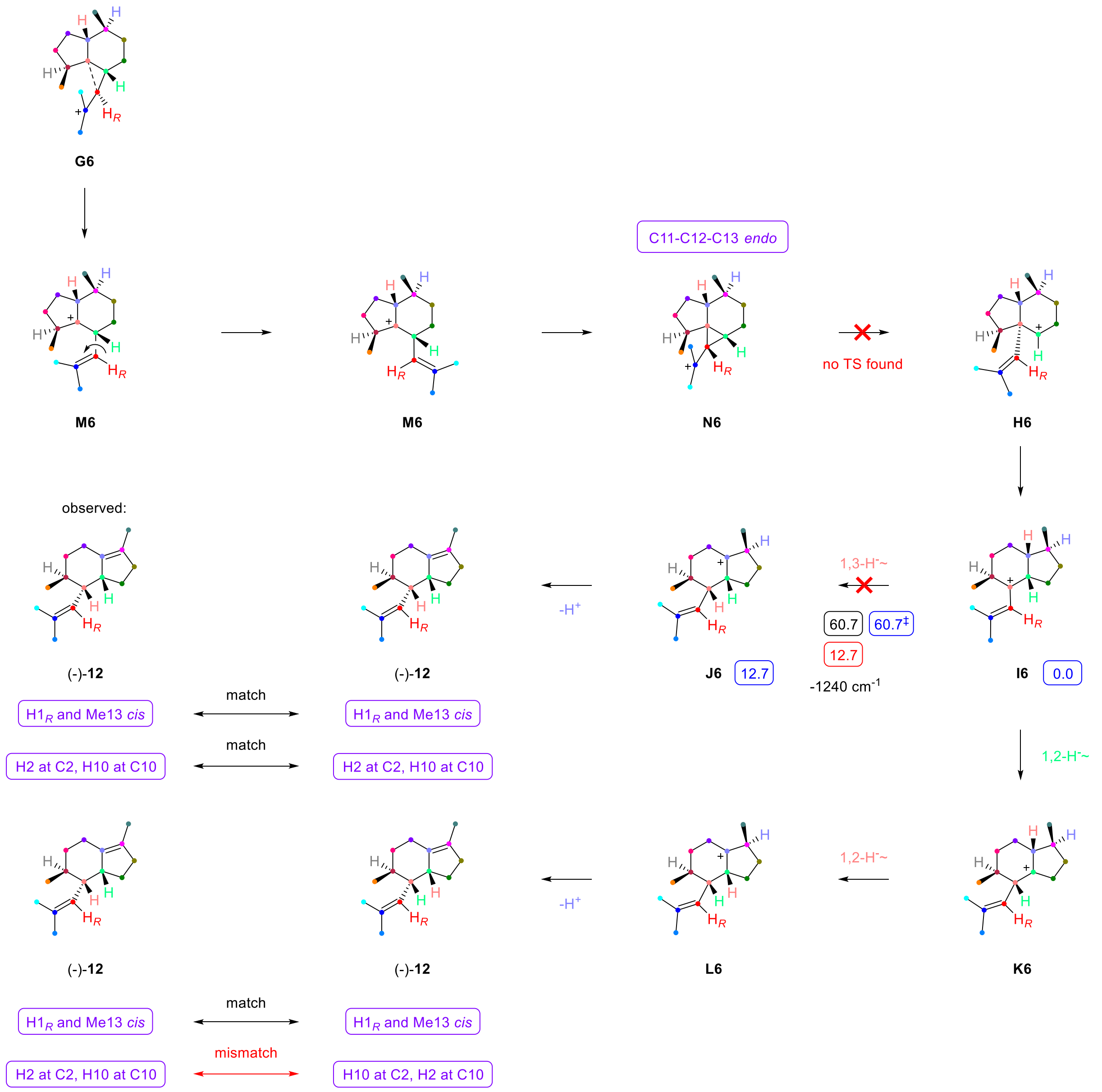

Scheme S17. Mechanism for the biosynthesis of **12** starting from FPP with H6 down and H10 up, and proceeding through NPP to turn H2 up (2U-6D-10U) and configurational change in **G6**.

17. Mechanistic alternative XIII

This mechanism starts with a conformation of FPP with H6 up and H10 down, and proceeding through NPP to turn H2 up (2U-6U-10D). In intermediate **C7** H1$_R$ (red) and C12 (light blue) are *cis*, which mismatches the situation in the product **12**. For the rearrangement of **G7** with C11-C12-C13 in *endo* orientation no transition state could be localised through DFT computations. The subsequent 1,3-hydride shift to **J6** is possible (reaction barrier: 6.3 kcal/mol), while a sequence of two 1,2-hydride shifts will lead to the wrong positions for H2 and H10. Because of the mismatching situation for the relative orientation of H1$_R$ with respect to Me12/Me13, mechanistic alternative XIII cannot explain the formation of **12**. The B-F-C mechanism (Scheme S4) explains required configurational inversions in **J7**/**L7**.

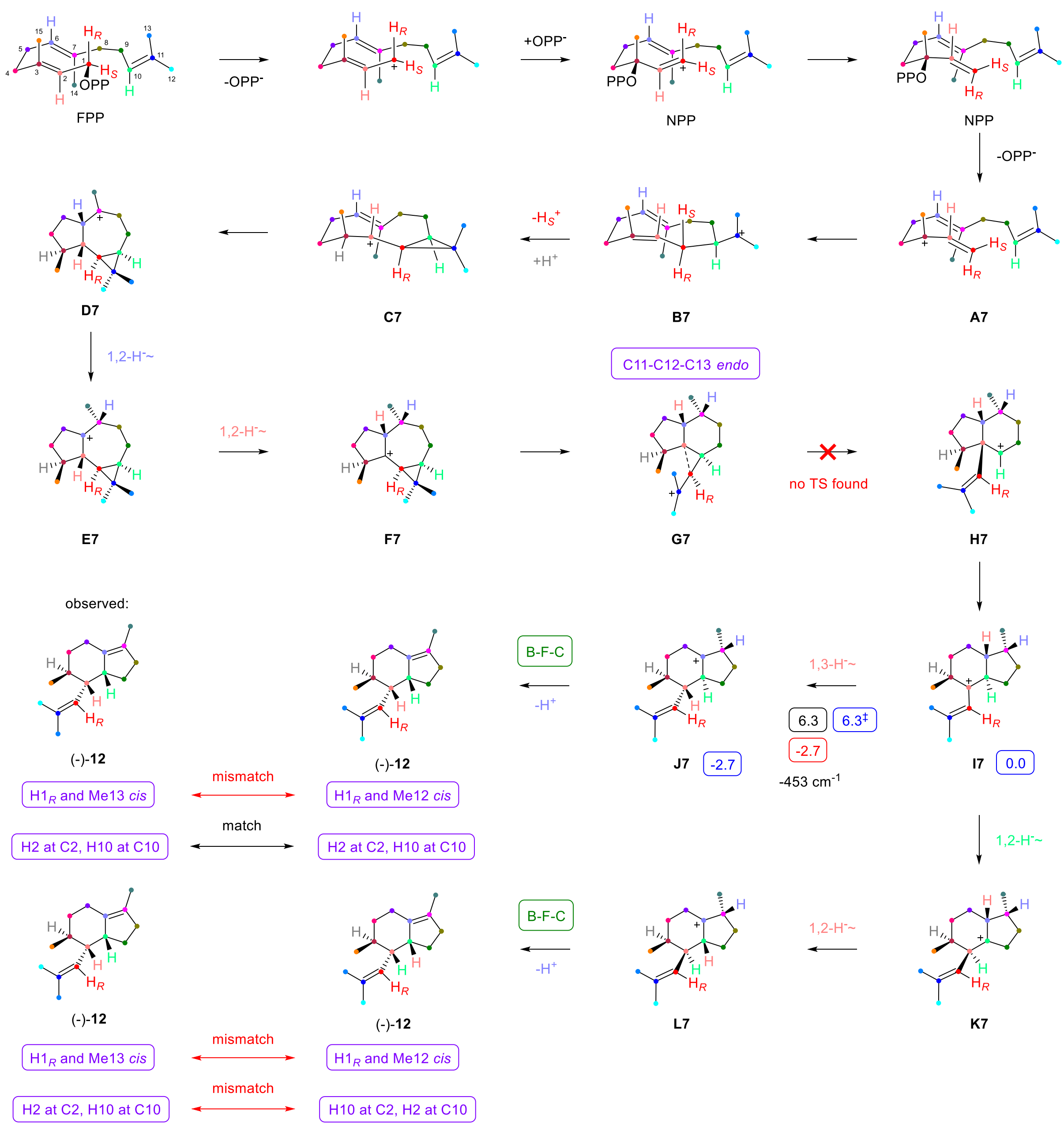

Scheme S18. Mechanism for the biosynthesis of **12** starting from FPP with H6 up and H10 down, and proceeding through NPP to turn H2 up (2U-6U-10D).

18. Mechanistic alternative XIV

This mechanism is a modification of mechanistic alternative XIII with same steps until **G7** (Scheme S18) and starts with a conformation of FPP with H6 up and H10 down, and proceeding through NPP to turn H2 up (2U-6U-10D). In intermediate **C7** H1$_R$ (red) and C12 (light blue) are *cis*, which mismatches the situation in the product **12**. In **G7** a configurational inversion according to the mechanism of Scheme S5 may happen, leading to **N7**. DFT computations show a reaction barrier of 20.6 kcal/mol for the rearrangement to **I7** with skipped **H7**. The subsequent 1,3-hydride shift to **J7** is possible (reaction barrier: 6.3 kcal/mol, or 14.7 kcal/mol from the conformer of **I7** obtained from **N7**). A sequence of two 1,2-hydride shifts will lead to the wrong positions for H2 and H10. Because of the mismatching situation for the relative orientation of H1$_R$ with respect to Me12/Me13, mechanistic alternative XIV cannot explain the formation of **12**. The B-F-C mechanism (Scheme S4) explains required configurational inversions in **J7**/**L7**. Alternatively, from **N7** a two-step rearrangement via **H7** to **I8** with conformational flip turning H10 from bottom to top was found (highest barrier: 31.5 kcal/mol), similar to the conformational flip shown in Scheme 3 of main text.

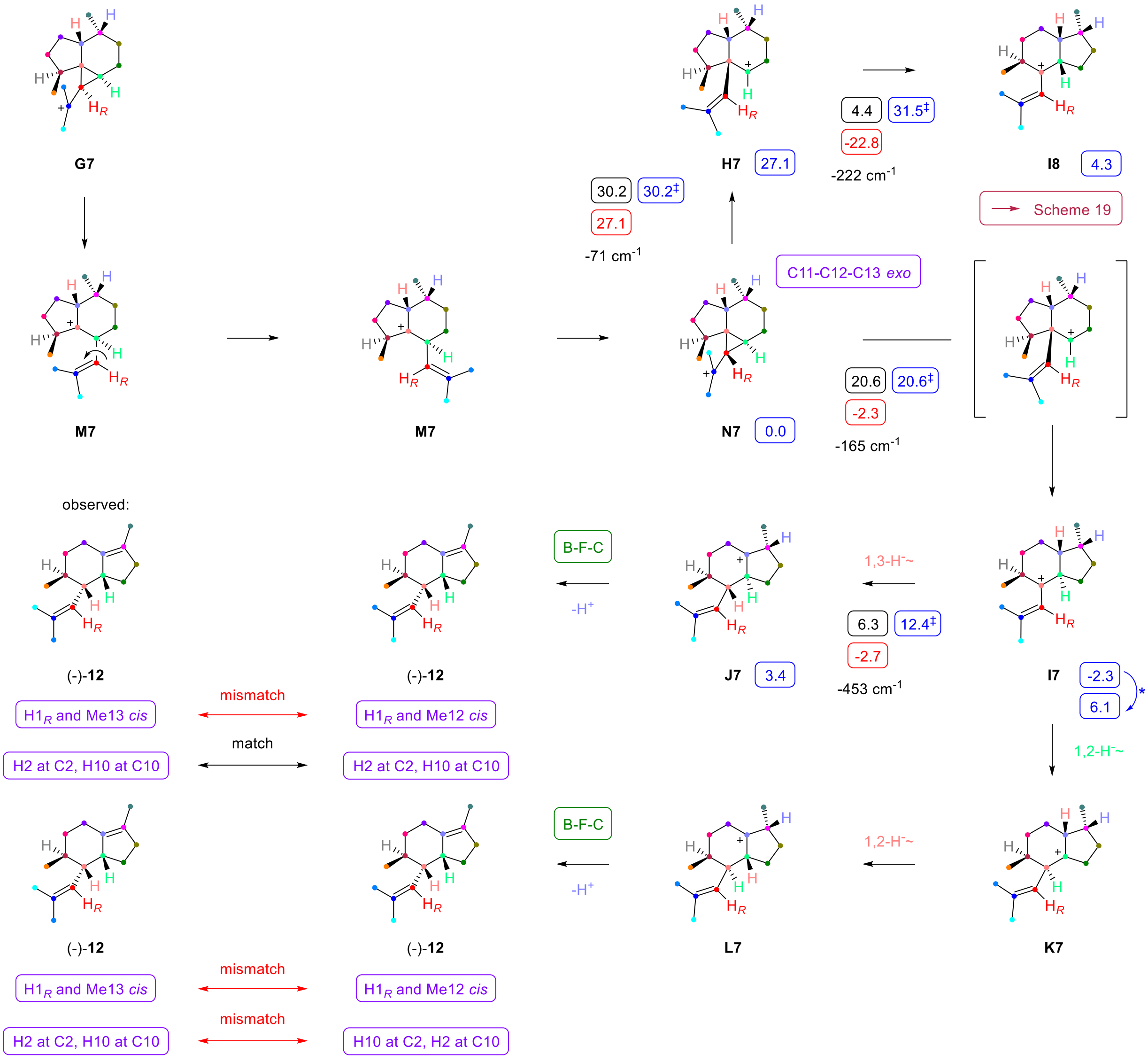

Scheme S19. Mechanism for the biosynthesis of **12** starting from FPP with H6 up and H10 down, and proceeding through NPP to turn H2 up (2U-6U-10D) and configurational change in **G7**.

19. Mechanistic alternative XV

This mechanism starts with a conformation of FPP with H6 and H10 up, and proceeding through NPP to turn H2 up (2U-6U-10U). In intermediate **C8** H1$_R$ (red) and C13 (dark blue) are *cis*, which matches the situation in the product **12**. DFT computations show a comparably low reaction barrier of 18.7 kcal/mol for the rearrangement to **I8** with skipping of **H8**, but the subsequent 1,3-hydride shift to **J8** is impossible (reaction barrier: 59.2 kcal/mol). A sequence of two 1,2-hydride shifts will have lower reaction barriers, but will lead to the wrong positions for H2 and H10. Conclusively, mechanistic alternative XV cannot explain the formation of **12**. Configurational inversions through the B-F-C mechanism (Scheme S4) are not required.

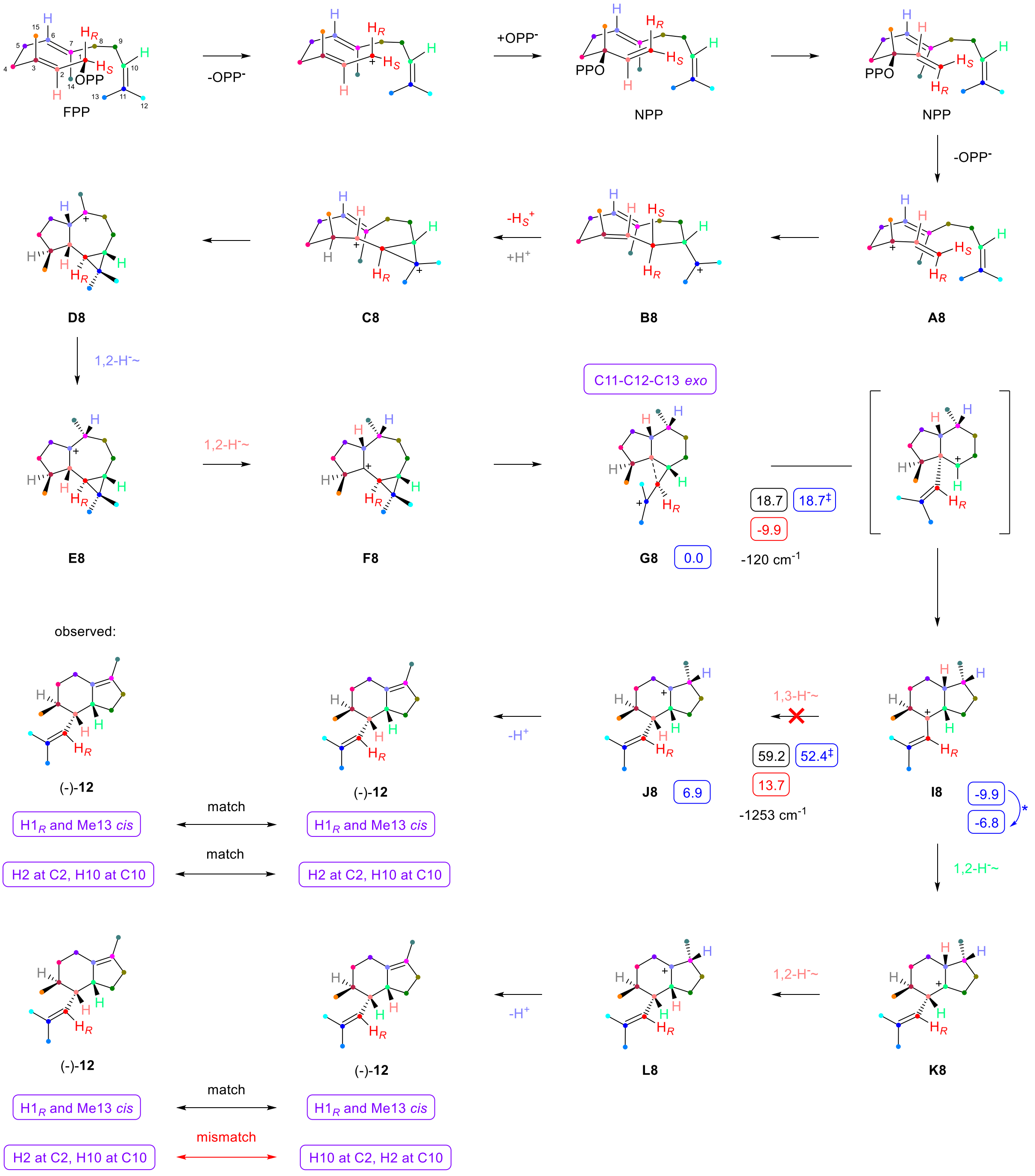

Scheme S20. Mechanism for the biosynthesis of **12** starting from FPP with H6 and H10 up, and proceeding through NPP to turn H2 up (2U-6U-10U).

20. Mechanistic alternative XVI

This mechanism is a modification of mechanistic alternative XV with same steps until **G8** (Scheme S20) and starts with a conformation of FPP with H6 and H10 up, and proceeding through NPP to turn H2 up (2U-6U-10U). In intermediate **C8** H1$_R$ (red) and C13 (dark blue) are *cis*, which matches the situation in the product **12**. In **G8** a configurational inversion according to the mechanism of Scheme S5 may happen, leading to **N8**. For the rearrangement of **N8** with C11-C12-C13 in *endo* orientation no transition state could be localised through DFT computations, and also the subsequent 1,3-hydride shift to **J3** is impossible (reaction barrier: 59.2 kcal/mol). A sequence of two 1,2-hydride shifts will have much lower reaction barriers, but this will lead to the wrong positions for H2 and H10. Conclusively, mechanistic alternative XVI cannot explain the formation of **12**. Configurational inversions through the B-F-C mechanism (Scheme S4) are not required.

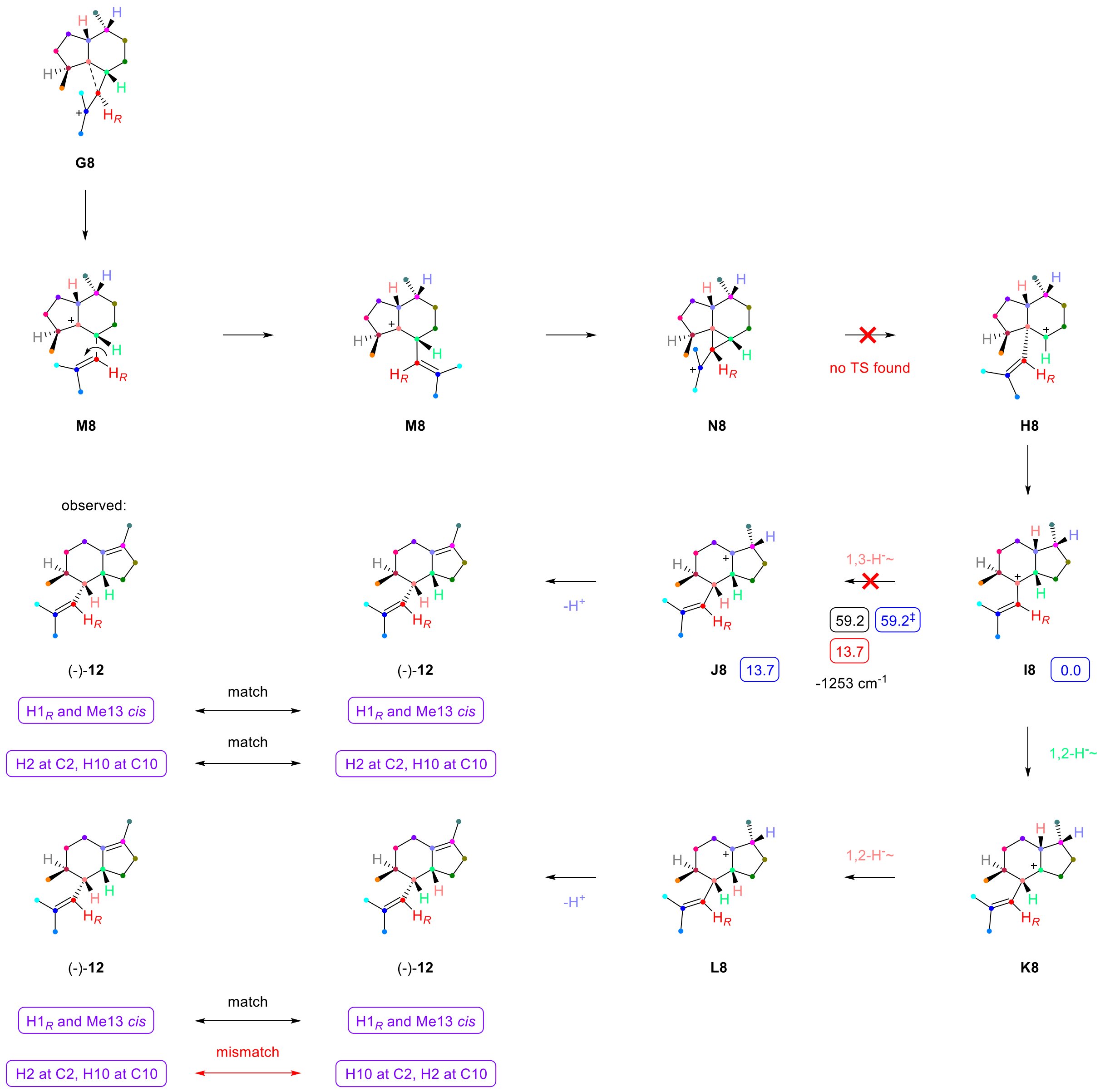

Scheme S21. Mechanism for the biosynthesis of **12** starting from FPP with H6 and H10 up, and proceeding through NPP to turn H2 up (2U-6U-10U) and configurational change in **G8**.

21. Mechanistic alternative XVII

The above mechanistic alternatives are stereochemical permutations of the same basic mechanistic principle. Another alternative that was considered to explain the change of hydrogens from the down to the top side proceeds from **G1** through opening to **H9**, a 1,2-hydride shift to **I9**, ring opening to **J9**, conformational change to **K9** turning H2 (light red) from bottom to top, and ring closure to **L9**. DFT computations revealed that the ring opening in **I9** is impossible, because of the high stability of the allyl cation. Any trials to open the ring always immediately resulted in ring closure. Therefore, mechanistic alternative XVII is excluded.

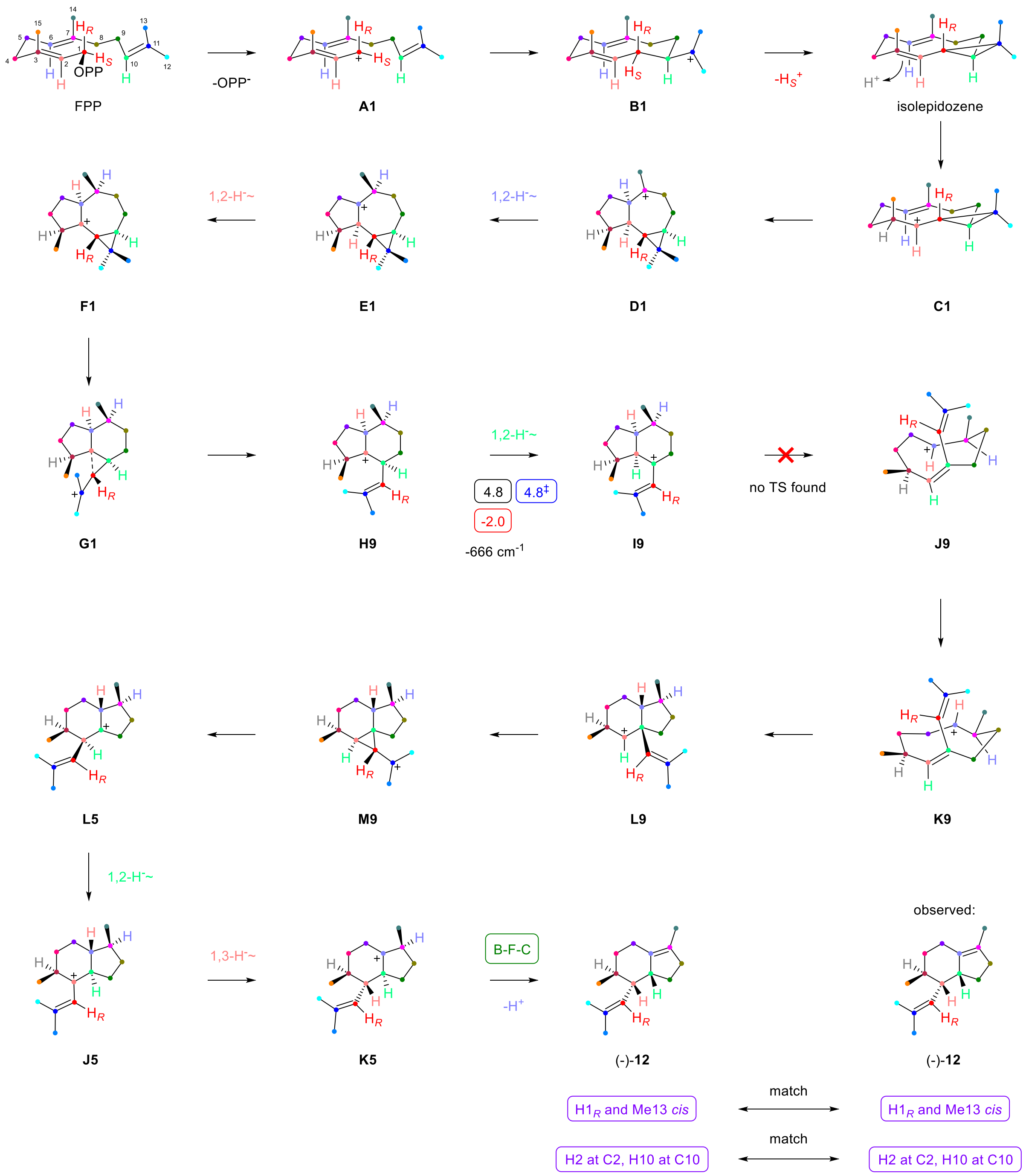

Scheme S22. First mechanistic alternative through ring opening, conformational change and ring closure to explain turning of H2 from bottom to top.

22. Mechanistic alternative XVIII

Another alternative following a similar idea as in mechanistic alternative XVII proceeds from **G1** through opening to **H10**, another ring opening to **I10**, conformational change to **J10** turning H2 (light red) from bottom to top, and ring closure to **K10**. DFT computations revealed that the ring opening in **G1** is impossible. Any trials to open the ring always immediately resulted in ring closure. Therefore, mechanistic alternative XVIII is excluded.

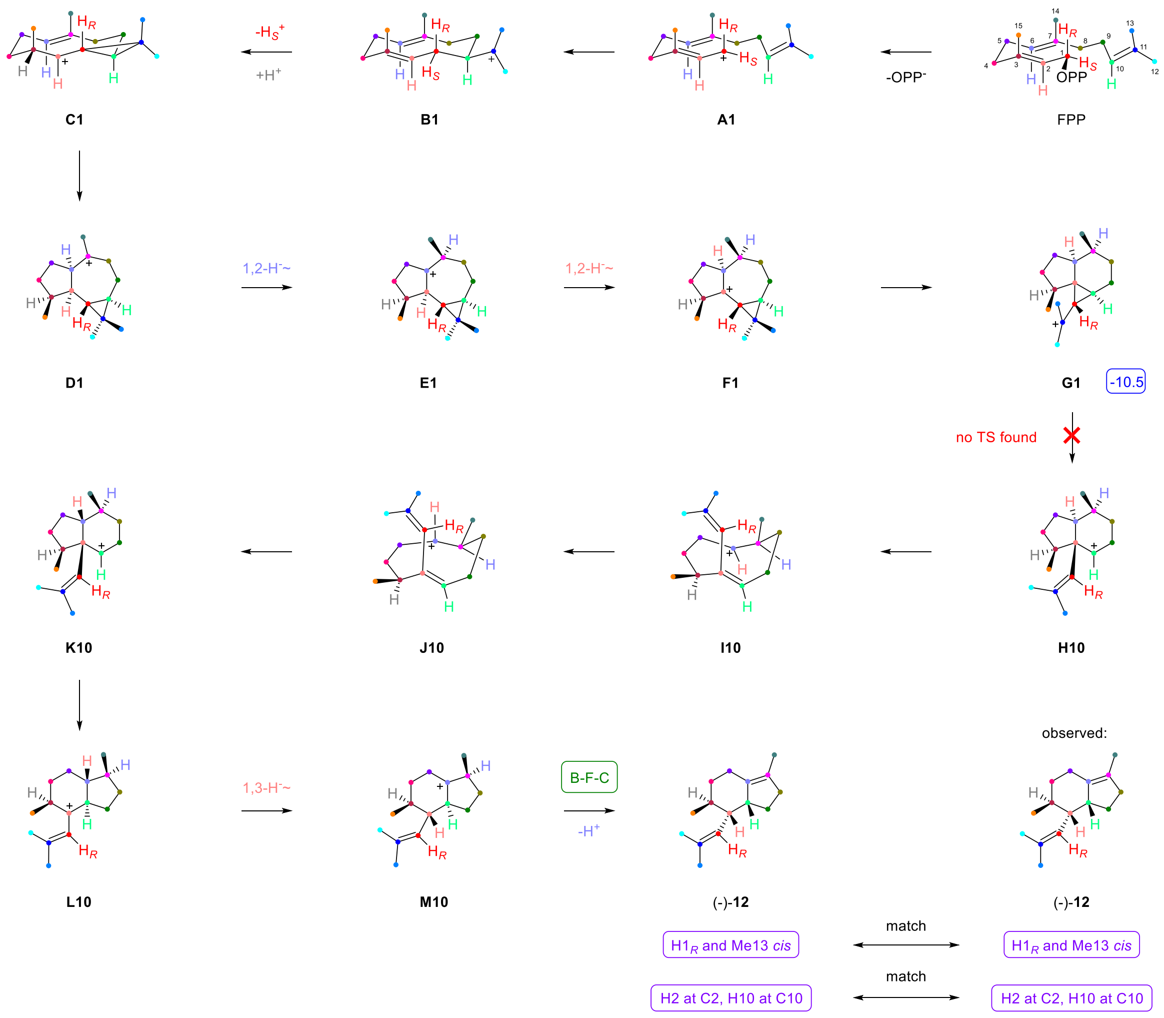

Scheme S23. Second mechanistic alternative through ring opening, conformational change and ring closure to explain turning of H2 from bottom to top.

Cartesian coordinates of computed structures (Scheme 2B of main text, Table S10)

Gibbs energies (G in Hartree) and imagininary frequencies of TS (T in cm^{-1}) (wB97M-V/Def2-TZVPPD//B97D3/6-31G(d,p)-sp-density-fitting, 1 bar, 298.15 K)

Step C – E

**C**	G = -585.951890		
C	1.234156	-1.875330	0.646597
C	1.746901	-0.836764	-0.327398
C	2.449907	0.409520	0.031439
C	0.943630	0.521168	-0.492432
C	-0.061413	-2.589416	0.168263
C	-0.103385	0.817423	0.412021
C	-1.868756	-0.960636	0.765784
C	-1.192874	-1.643891	-0.209467
H	0.051211	0.558269	1.460594
H	-0.392907	-3.260002	0.974308
H	1.078666	-1.447250	1.645439
C	-1.484367	-1.545081	-1.678909
H	-0.591079	-1.197051	-2.222563
H	-1.704098	-2.549155	-2.072828
H	-2.323112	-0.889658	-1.928497
H	0.181342	-3.222512	-0.697119
H	0.838420	0.873929	-1.517110
H	1.988911	-1.241314	-1.315849
H	-1.593636	-1.182794	1.803899
H	2.015673	-2.640796	0.763886
C	-1.190930	1.815531	0.180661
C	-2.971960	0.040312	0.595464
H	-3.816600	-0.224060	1.249333
H	-3.356599	0.059152	-0.431889
C	-2.454507	1.442933	0.998517
H	-2.203585	1.442819	2.071172
H	-3.231091	2.204707	0.848651
H	-0.773505	2.717718	0.683551
C	-1.477113	2.184399	-1.283049
H	-1.798979	1.316415	-1.870001
H	-0.596030	2.617216	-1.772962
H	-2.277434	2.932583	-1.325034
C	2.776523	0.772942	1.472069
H	2.802135	1.862852	1.601925
H	3.777117	0.385960	1.710770
H	2.085612	0.352115	2.209875
C	3.440730	0.976867	-0.977901
H	4.439907	0.566803	-0.770416
H	3.501687	2.070139	-0.898309
H	3.178715	0.714064	-2.010772

C-E-TS G = -585.949308, T = -707

C	0.796837	-2.204964	0.495170
C	1.498130	-1.248492	-0.449204
C	2.402315	-0.108313	-0.023961
C	0.992564	0.179108	-0.531728
C	-0.613468	-2.549449	-0.016034
C	-0.118449	0.634944	0.399748
C	-1.464610	-0.075036	0.143352
C	-1.674839	-1.466429	-0.086631
H	0.163711	0.411325	1.441172
H	-1.049178	-3.385244	0.554889
H	0.736115	-1.809875	1.519152
C	-3.036058	-1.952362	-0.534324
H	-3.064558	-1.863395	-1.632173
H	-3.191249	-3.004964	-0.276511
H	-3.866883	-1.357257	-0.143780
H	-0.549929	-2.924550	-1.055322
H	0.925566	0.606525	-1.534843
H	1.751350	-1.710840	-1.408907
H	-1.764440	-0.857318	1.135461
H	1.359695	-3.146174	0.575470
C	-0.468743	2.168015	0.371573
C	-2.559491	0.975241	0.025923
H	-3.537358	0.653540	0.403502
H	-2.687570	1.177556	-1.051691
C	-1.966822	2.198115	0.750168
H	-2.098298	2.077991	1.837089
H	-2.461357	3.131418	0.456420
H	0.134194	2.669513	1.141515
C	-0.198625	2.866546	-0.971021
H	-0.751648	2.412933	-1.807186
H	0.867487	2.849668	-1.227692
H	-0.506354	3.918053	-0.905595
C	2.775437	0.106866	1.437164
H	3.011081	1.164780	1.622344
H	3.671161	-0.479470	1.685970
H	1.996045	-0.190939	2.150527
C	3.537386	0.248230	-0.979131
H	4.421253	-0.375309	-0.782689
H	3.834733	1.299724	-0.855367
H	3.247051	0.100190	-2.028148

E G = -585.956506

C	0.870367	-2.230034	0.390860
C	1.613412	-1.213718	-0.459406
C	2.410768	-0.031072	0.047342
C	1.044845	0.182560	-0.602694
C	-0.510924	-2.523873	-0.226577
C	-0.146968	0.644625	0.212077
C	-1.425561	-0.090101	0.183384
C	-1.654766	-1.552829	0.148914

H	0.071489	0.445068	1.300818
H	-0.859351	-3.520766	0.079369
H	0.758314	-1.900111	1.435453
C	-2.977168	-1.940590	-0.573729
H	-2.895208	-1.723291	-1.646519
H	-3.137825	-3.017682	-0.456527
H	-3.856709	-1.424229	-0.175066
H	-0.425144	-2.555630	-1.324469
H	1.041522	0.607728	-1.609196
H	1.977977	-1.644072	-1.397451
H	-1.859157	-1.695601	1.242970
H	1.452569	-3.161308	0.437174
C	-0.505825	2.173625	0.185230
C	-2.541261	0.866793	0.376508
H	-3.386693	0.473192	0.956324
H	-2.935595	0.998541	-0.656997
C	-1.896362	2.184800	0.858633
H	-1.801061	2.158900	1.954898
H	-2.499386	3.060009	0.593017
H	0.233470	2.715723	0.790132
C	-0.518470	2.773193	-1.230519
H	-1.189071	2.229806	-1.913266
H	0.483324	2.773796	-1.675974
H	-0.860522	3.814960	-1.187980
C	2.597051	0.209512	1.538598
H	2.772648	1.275335	1.743994
H	3.470586	-0.347495	1.905500
H	1.746914	-0.117711	2.156130
C	3.621657	0.390170	-0.779352
H	4.510355	-0.186475	-0.486378
H	3.847462	1.455863	-0.629034
H	3.454336	0.227088	-1.852531

Step E – F

E G = -585.956509

C	-0.870080	2.230124	0.391093
C	-1.613280	1.213974	-0.459237
C	-2.410708	0.031296	0.047326
C	-1.044811	-0.182336	-0.602715
C	0.511200	2.523835	-0.226417
C	0.146898	-0.644573	0.212068
C	1.425625	0.089956	0.183389
C	1.654981	1.552671	0.148937
H	-0.071483	-0.444982	1.300804
H	0.859749	3.520688	0.079530
H	-0.758012	1.900091	1.435650
C	2.977315	1.940366	-0.573870
H	2.895026	1.723445	-1.646711
H	3.138266	3.017378	-0.456337

H	3.856821	1.423647	-0.175601
H	0.425338	2.555652	-1.324302
H	-1.041420	-0.607361	-1.609283
H	-1.977830	1.644464	-1.397223
H	1.859537	1.695417	1.242950
H	-1.452154	3.161473	0.437521
C	0.505496	-2.173619	0.185238
C	2.541150	-0.867121	0.376427
H	3.386789	-0.473719	0.956058
H	2.935195	-0.999049	-0.657178
C	1.896043	-2.185031	0.858614
H	1.800784	-2.159082	1.954878
H	2.498929	-3.060330	0.592985
H	-0.233886	-2.715584	0.790156
C	0.517985	-2.773201	-1.230512
H	1.188722	-2.229992	-1.913269
H	-0.483815	-2.773523	-1.675955
H	0.859754	-3.815062	-1.187978
C	-2.596964	-0.209597	1.538539
H	-2.771991	-1.275548	1.743775
H	-3.470843	0.346861	1.905450
H	-1.747051	0.118044	2.156145
C	-3.621597	-0.389733	-0.779485
H	-4.510234	0.187013	-0.486532
H	-3.847558	-1.455408	-0.629261
H	-3.454164	-0.226602	-1.852638

E-F-TS G = -585.955177, T = -657

C	-1.004149	2.242086	0.242916
C	-1.724224	1.138730	-0.523586
C	-2.361340	-0.083063	0.111091
C	-1.073295	-0.209717	-0.729975
C	0.389925	2.520703	-0.350081
C	0.211043	-0.628068	-0.127069
C	1.330604	0.163060	0.279288
C	1.534819	1.660253	0.226342
H	0.261968	-0.184633	1.086227
H	0.675571	3.566477	-0.167423
H	-0.918766	2.010701	1.316375
C	2.856285	1.940370	-0.544130
H	2.803155	1.550059	-1.569437
H	2.999381	3.025827	-0.604242
H	3.733327	1.512225	-0.046383
H	0.357338	2.396857	-1.444340
H	-1.217900	-0.706181	-1.694304
H	-2.240778	1.507547	-1.414851
H	1.708119	1.968661	1.275147
H	-1.616295	3.153021	0.192382
C	0.565836	-2.117200	-0.029696
C	2.436549	-0.760951	0.759773
H	2.970409	-0.370865	1.635704

H	3.168417	-0.811486	-0.063933
C	1.747498	-2.124971	0.972583
H	1.363637	-2.186158	2.002789
H	2.426471	-2.970987	0.818647
H	-0.298223	-2.685914	0.340972
C	0.952290	-2.646867	-1.428963
H	1.784575	-2.075728	-1.863683
H	0.107806	-2.606098	-2.127653
H	1.267795	-3.693953	-1.345009
C	-2.313357	-0.300302	1.616758
H	-2.415854	-1.367213	1.859902
H	-3.143571	0.232238	2.101052
H	-1.393882	0.066373	2.098517
C	-3.630834	-0.630848	-0.528875
H	-4.514823	-0.131756	-0.107137
H	-3.734506	-1.709484	-0.342798
H	-3.639278	-0.468811	-1.614779

F G = -585.971462

C	-1.315998	2.099610	-0.055113
C	-1.882164	0.823251	-0.674789
C	-2.417688	-0.305490	0.107074
C	-0.983844	-0.432062	-0.806757
C	0.131939	2.385492	-0.491371
C	0.254208	-0.630327	-0.186485
C	0.948000	0.247470	0.813097
C	1.237272	1.740068	0.375883
H	0.241067	0.324020	1.660820
H	0.306749	3.470409	-0.480269
H	-1.388350	2.083311	1.040982
C	2.586698	1.925547	-0.340009
H	2.646313	1.324904	-1.258848
H	2.695586	2.978188	-0.633458
H	3.444119	1.679215	0.295728
H	0.261705	2.070792	-1.540222
H	-1.196588	-1.108527	-1.635771
H	-2.397187	0.987285	-1.628296
H	1.283245	2.299492	1.324653
H	-1.967860	2.920677	-0.384662
C	1.039136	-1.890942	-0.420767
C	2.165872	-0.593480	1.303489
H	2.339505	-0.468189	2.378609
H	3.084802	-0.285370	0.792133
C	1.813766	-2.043426	0.915769
H	1.155600	-2.499414	1.671016
H	2.695714	-2.686746	0.807204
H	0.360383	-2.732029	-0.629983
C	1.984017	-1.731840	-1.647496
H	2.708795	-0.923212	-1.500774
H	1.417201	-1.527845	-2.564456
H	2.539169	-2.668506	-1.781159

C	-2.367769	-0.371603	1.614566
H	-2.224118	-1.401137	1.965424
H	-3.347277	-0.033492	1.986397
H	-1.621398	0.278110	2.078136
C	-3.480265	-1.196142	-0.509765
H	-4.459888	-0.857519	-0.138241
H	-3.358362	-2.243757	-0.204937
H	-3.498008	-1.137414	-1.603970

Step F – G

F G = -585.975564

C	-1.291951	-2.074978	0.291467
C	-1.857387	-0.726196	0.725216
C	-2.418448	0.289498	-0.170205
C	-0.962924	0.564225	0.629400
C	0.154731	-2.290866	0.780390
C	0.269047	0.610957	-0.027726
C	0.855753	-0.418758	-0.935563
C	1.270225	-1.732467	-0.135931
H	0.107469	-0.764675	-1.660873
H	0.342026	-3.366458	0.906220
H	-1.365059	-2.219025	-0.794663
C	2.570831	-1.603664	0.670645
H	2.477727	-0.859529	1.474497
H	2.796360	-2.568099	1.145315
H	3.432897	-1.339878	0.047819
H	0.255873	-1.848864	1.785245
H	-1.127386	1.315964	1.400512
H	-2.304700	-0.743070	1.725330
H	1.442394	-2.460450	-0.946183
H	-1.944195	-2.838448	0.738807
C	1.199471	1.789812	0.113611
C	2.030741	0.306442	-1.642523
H	1.654561	0.779095	-2.562176
H	2.830498	-0.384176	-1.934411
C	2.489756	1.387568	-0.643802
H	2.951654	2.251425	-1.136127
H	3.225647	0.989692	0.068618
H	0.678295	2.570282	-0.486462
C	1.410702	2.355354	1.529887
H	1.823647	1.586180	2.195589
H	0.484714	2.742175	1.973284
H	2.126894	3.184495	1.484551
C	-2.444151	0.158572	-1.676644
H	-2.303830	1.132439	-2.162225
H	-3.443811	-0.205852	-1.957083
H	-1.724386	-0.552994	-2.087127
C	-3.468280	1.245856	0.373410
H	-4.459080	0.852294	0.100137

H	-3.373681	2.243842	-0.073805
H	-3.433223	1.335502	1.465411

F-G-TS G = -585.947317, T = -202

C	-0.705012	-1.043163	1.549624
C	-1.144133	0.215629	0.737657
C	-2.343384	-0.161931	-0.436459
C	-1.392333	0.860298	-0.794430
C	0.800218	-1.287473	1.502502
C	-0.015926	0.650442	-0.399817
C	0.933201	-0.466031	-0.942147
C	1.261276	-1.621934	0.075817
H	0.464107	-0.922259	-1.823901
H	1.024060	-2.136050	2.165087
H	-1.232781	-1.941921	1.214264
C	2.724670	-2.090149	0.061004
H	3.411816	-1.319168	0.435281
H	2.832700	-2.969172	0.709878
H	3.050614	-2.382734	-0.945193
H	1.348090	-0.420623	1.905316
H	-1.750633	1.896909	-0.727726
H	-1.442973	1.024433	1.402561
H	0.656963	-2.491412	-0.240026
H	-1.047318	-0.831597	2.571282
C	0.816481	1.948772	-0.157391
C	2.149024	0.385058	-1.402534
H	1.913616	0.836004	-2.379501
H	3.056917	-0.210227	-1.533640
C	2.274158	1.490967	-0.351722
H	2.922848	2.318877	-0.663508
H	2.680210	1.094541	0.593095
H	0.569529	2.626723	-0.994230
C	0.548599	2.691219	1.155862
H	0.754503	2.056188	2.028932
H	-0.483159	3.065062	1.232372
H	1.208033	3.565772	1.221887
C	-2.275912	-1.582627	-0.967766
H	-2.817266	-1.593767	-1.924151
H	-2.792458	-2.275613	-0.292687
H	-1.264143	-1.947744	-1.150664
C	-3.749897	0.301728	-0.093104
H	-4.176440	-0.346347	0.683971
H	-4.397692	0.221174	-0.976636
H	-3.775321	1.338122	0.265358

G G = -585.968152

C	0.011063	-1.204354	1.934882
C	-0.488813	0.134972	1.397079
C	-2.459451	-0.522746	-0.318199
C	-1.719645	0.357543	0.459962
C	1.437575	-1.427202	1.409517

C	-0.014682	0.607034	0.105628
C	0.775860	-0.304465	-0.852637
C	1.471763	-1.530722	-0.126032
H	0.094243	-0.739016	-1.597371
H	1.854403	-2.347247	1.841608
H	-0.639195	-2.046048	1.664714
C	2.897885	-1.817262	-0.620488
H	3.599745	-1.023900	-0.327636
H	3.258704	-2.753684	-0.174923
H	2.942064	-1.932051	-1.711320
H	2.085066	-0.598529	1.746299
H	-2.178764	1.333665	0.618411
H	-0.562080	0.913222	2.163201
H	0.870231	-2.418759	-0.388307
H	-0.003039	-1.155037	3.031626
C	0.278725	2.080526	-0.196704
C	1.691497	0.715861	-1.607417
H	1.223761	0.967172	-2.570484
H	2.684355	0.316486	-1.829409
C	1.729678	1.968229	-0.721793
H	2.036434	2.876320	-1.256234
H	2.418948	1.831798	0.127840
H	-0.351050	2.377727	-1.055432
C	0.083717	3.078600	0.945940
H	0.729213	2.838259	1.802100
H	-0.955981	3.131274	1.299620
H	0.353908	4.084245	0.600767
C	-2.074390	-1.906816	-0.720415
H	-1.864843	-1.911779	-1.804543
H	-2.933824	-2.581228	-0.589636
H	-1.208483	-2.319097	-0.204951
C	-3.788231	-0.054151	-0.836556
H	-4.586454	-0.606796	-0.312017
H	-3.902061	-0.304206	-1.901804
H	-3.960601	1.016922	-0.688731

Step G – I

G G = -585.974572

C	-0.444475	1.838425	-0.322819
C	-0.038037	0.397108	-0.011471
C	-2.451166	-0.933633	-0.357392
C	0.957250	2.513232	-0.280087
C	-0.381137	-0.366446	1.179343
C	1.248354	0.091071	-0.793015
C	1.992792	1.463163	-0.756769
H	-1.101930	0.103825	1.854249
H	0.959256	3.428484	-0.885084
H	-0.803735	1.864643	-1.366061
H	1.169212	2.812853	0.757296

H	2.843913	1.422648	-0.066353
C	0.652462	-1.282829	1.856290
C	2.049442	-1.126465	-0.265106
H	1.519936	-2.043979	-0.584378
C	2.065934	-1.118157	1.273124
H	2.709784	-1.924379	1.650396
H	2.507766	-0.172475	1.629817
H	0.647835	-1.042756	2.929286
C	-2.978358	-1.821729	-1.444079
H	-3.591385	-2.621636	-0.995636
H	-3.660598	-1.257862	-2.098432
H	-2.189899	-2.283654	-2.046825
C	-3.478611	-0.151396	0.392184
H	-3.959263	0.577764	-0.278653
H	-4.282315	-0.838531	0.700280
H	-3.098799	0.369552	1.272110
C	-1.088103	-0.968680	-0.083594
H	-0.527496	-1.743322	-0.607040
H	0.960760	-0.104033	-1.840957
H	2.398699	1.698562	-1.747722
C	-1.437074	2.565287	0.588309
H	-1.382837	3.641943	0.381660
H	-2.477180	2.266742	0.431813
H	-1.184167	2.429595	1.649218
H	0.322688	-2.329321	1.773954
C	3.457173	-1.197199	-0.877390
H	3.948331	-2.136524	-0.592651
H	3.417532	-1.155206	-1.974491
H	4.095204	-0.373456	-0.530962

G-I-TS G = -585.945650, T = -294

C	-0.334870	1.881795	-0.413592
C	-0.134535	0.363506	-0.122603
C	-2.443133	-0.865736	-0.383161
C	1.040864	2.455511	-0.021426
C	-0.128470	-0.030962	1.276304
C	1.310425	0.097836	-0.770995
C	2.086587	1.414260	-0.492675
H	-0.536877	0.661915	2.023483
H	1.205482	3.456478	-0.438718
H	-0.446046	1.948092	-1.509709
H	1.085708	2.561644	1.077901
H	2.869260	1.271395	0.264062
C	0.503030	-1.268652	1.771020
C	1.978730	-1.209238	-0.305841
H	1.376647	-2.050183	-0.695578
C	1.978471	-1.317955	1.223317
H	2.419516	-2.264865	1.562299
H	2.565938	-0.507392	1.679426
H	0.462485	-1.326969	2.864523
C	-3.282109	-1.749582	-1.276489

H	-3.663113	-2.610888	-0.705197
H	-4.166815	-1.197751	-1.628646
H	-2.733922	-2.122141	-2.148716
C	-3.155160	-0.353304	0.838763
H	-4.120914	0.097109	0.569845
H	-3.381229	-1.196705	1.510128
H	-2.580854	0.381106	1.414373
C	-1.152206	-0.625186	-0.737013
H	-0.735095	-1.209622	-1.563312
H	1.105974	0.025235	-1.852320
H	2.592815	1.741296	-1.410093
C	-1.522631	2.601521	0.224938
H	-1.494880	3.664006	-0.049611
H	-2.481516	2.197676	-0.120254
H	-1.498733	2.562972	1.324446
H	-0.012096	-2.152116	1.365150
C	3.401606	-1.352293	-0.883768
H	3.823470	-2.331712	-0.625264
H	3.388304	-1.266538	-1.978046
H	4.076898	-0.581158	-0.490848

I G = -585.976540

C	-0.344980	2.101704	-0.128910
C	-0.677739	0.631189	-0.013227
C	-2.774751	-0.782386	-0.258668
C	1.119123	2.421644	0.214678
C	0.384708	-0.222584	0.534266
C	1.518652	0.043919	-0.605797
C	2.127400	1.445608	-0.443168
H	0.807150	0.215609	1.455220
H	1.332991	3.459211	-0.074220
H	-0.562434	2.395893	-1.172611
H	1.238201	2.386900	1.309090
H	3.031190	1.411009	0.177595
C	0.328634	-1.748447	0.636409
C	2.437483	-1.239560	-0.520001
H	2.330344	-1.755177	-1.487055
C	1.815278	-2.134569	0.577553
H	1.968572	-3.203560	0.383192
H	2.288906	-1.910620	1.547574
H	-0.154131	-2.104769	1.553139
C	-3.951966	-1.074281	-1.141060
H	-4.070631	-2.163478	-1.251303
H	-4.880658	-0.722148	-0.663034
H	-3.870586	-0.617062	-2.132614
C	-2.766631	-1.483318	1.065727
H	-3.765744	-1.393654	1.518632
H	-2.599062	-2.562616	0.926262
H	-2.031622	-1.089147	1.769631
C	-1.876221	0.197446	-0.645062
H	-2.194518	0.812199	-1.494993

H	1.002509	0.005108	-1.578392
H	2.434816	1.819499	-1.430002
C	-1.326973	2.906050	0.776103
H	-1.094001	3.974207	0.689329
H	-2.370801	2.748620	0.480475
H	-1.212267	2.614020	1.828423
H	-0.215621	-2.168898	-0.224307
C	3.928221	-0.965042	-0.289906
H	4.475626	-1.917720	-0.292329
H	4.359964	-0.330157	-1.074337
H	4.110371	-0.487509	0.683021

Step I – J

I G = -585.981942

C	-0.713310	1.539035	0.167285
C	-0.615464	0.037727	0.270418
C	-3.016728	-0.694284	-0.206114
C	0.641767	2.195892	-0.196819
C	0.757256	-0.449997	0.462773
C	1.546886	-0.082282	-0.876779
C	1.382563	1.397034	-1.285323
H	1.231109	0.182007	1.237397
H	0.446474	3.225757	-0.526784
H	-1.444500	1.795473	-0.613711
H	1.271691	2.279058	0.701779
H	2.355906	1.862305	-1.488538
C	1.142340	-1.914923	0.678027
C	2.951904	-0.654799	-0.535303
H	3.459477	-0.888908	-1.484809
C	2.644440	-1.970063	0.258315
H	2.838194	-2.862466	-0.349725
H	3.295364	-2.040694	1.139917
H	0.972895	-2.248272	1.710329
C	-3.916311	-1.887923	-0.212988
H	-4.348344	-2.018129	-1.219578
H	-3.424983	-2.816576	0.093908
H	-4.779829	-1.696304	0.446311
C	-3.655733	0.595419	-0.635026
H	-4.707523	0.446177	-0.899684
H	-3.604117	1.355202	0.155885
H	-3.139185	1.015235	-1.511632
C	-1.684509	-0.880736	0.164834
H	-1.421352	-1.912995	0.402360
H	1.110127	-0.727570	-1.656545
H	0.806964	1.455520	-2.221591
C	-1.244988	2.098576	1.522290
H	-2.209760	1.663079	1.807142
H	-0.529455	1.887807	2.327405
H	-1.358109	3.186164	1.438915

H	0.550611	-2.566639	0.017934
C	3.842342	0.304181	0.266179
H	3.389663	0.579964	1.231331
H	4.800485	-0.181357	0.493623
H	4.063470	1.229026	-0.282255

I-J-TS G = -585.961725, T = -460

C	-0.646868	1.905223	-0.034170
C	-0.393458	0.431427	0.353280
C	-2.452949	-0.895349	-0.307266
C	0.703118	2.540279	-0.465051
C	0.926840	0.224265	1.110775
C	1.362765	0.162297	-0.335080
C	1.644184	1.458746	-1.058199
H	1.243856	1.092141	1.693543
H	0.535308	3.350174	-1.185304
H	-1.346840	1.901117	-0.883144
H	1.185129	2.996725	0.410647
H	2.696789	1.728528	-0.884378
C	1.206511	-1.162038	1.710551
C	1.913392	-1.224702	-0.648782
H	1.602493	-1.570920	-1.645405
C	1.332578	-2.106582	0.490298
H	0.341073	-2.481559	0.199821
H	1.967891	-2.976584	0.694916
H	2.153599	-1.108930	2.265337
C	-3.602361	-1.769267	0.124188
H	-3.579303	-2.728000	-0.416942
H	-3.594844	-1.975406	1.200528
H	-4.560391	-1.292908	-0.134486
C	-2.423720	-0.543332	-1.773466
H	-2.521961	-1.455430	-2.380166
H	-3.288083	0.089007	-2.028877
H	-1.518703	-0.016742	-2.103715
C	-1.522155	-0.499613	0.597209
H	-1.596916	-0.867213	1.622863
H	0.064354	0.006708	-0.755511
H	1.520305	1.325834	-2.142624
C	-1.313155	2.661014	1.128150
H	-2.283799	2.217720	1.381900
H	-0.680825	2.651461	2.026829
H	-1.477103	3.707955	0.843697
H	0.436650	-1.486209	2.421777
C	3.458514	-1.135863	-0.619410
H	3.825548	-0.746015	0.339567
H	3.872387	-2.142858	-0.755280
H	3.843964	-0.497625	-1.423749

J G = -585.971196

C	-0.295168	2.121829	-0.379696
C	-0.512813	0.588217	-0.201077

C	-2.685752	-0.735320	-0.206662
C	1.209588	2.510538	-0.288773
C	0.677241	0.026698	0.892840
C	1.732560	0.083784	-0.080547
C	2.154469	1.363142	-0.689675
H	0.652628	0.792692	1.677057
H	1.412160	3.392506	-0.909344
H	-0.642321	2.336053	-1.403617
H	1.437742	2.806167	0.746647
H	3.183574	1.579337	-0.342813
C	0.538315	-1.440828	1.320389
C	2.181635	-1.278331	-0.491108
H	2.286316	-1.362177	-1.584381
C	1.103916	-2.224852	0.114564
H	0.312940	-2.404346	-0.628003
H	1.522516	-3.198838	0.393851
H	1.148912	-1.603680	2.220866
C	-3.991076	-1.040236	0.484653
H	-4.087791	-2.123796	0.657955
H	-4.091315	-0.522481	1.445592
H	-4.840319	-0.755790	-0.155583
C	-2.483573	-1.437154	-1.525105
H	-2.455418	-2.527072	-1.368307
H	-3.341328	-1.247884	-2.187816
H	-1.573816	-1.146260	-2.061647
C	-1.807609	0.135288	0.361153
H	-2.063809	0.544090	1.342197
H	-0.331617	0.097736	-1.168114
H	2.257534	1.230085	-1.780821
C	-1.115253	2.987246	0.588852
H	-2.192769	2.835258	0.453474
H	-0.867672	2.769601	1.638882
H	-0.898569	4.049396	0.416006
H	-0.495283	-1.710147	1.564981
C	3.599509	-1.508168	0.132743
H	3.577838	-1.411530	1.225514
H	3.918112	-2.527284	-0.117587
H	4.335831	-0.802753	-0.270301

Step J – K

J G = -585.974583

C	-0.400504	1.974366	-0.313042
C	-0.688373	0.454308	-0.190226
C	-3.012673	-0.600984	-0.182400
C	0.985702	2.188529	-0.938262
C	0.474327	-0.254088	0.709052
C	1.764686	0.099877	0.128059
C	2.131683	1.501677	-0.098363
H	0.356407	0.216208	1.701212

H	1.015295	1.778730	-1.958502
H	-1.137581	2.344459	-1.046639
H	1.233512	3.255967	-1.005183
H	2.161526	2.017017	0.878003
C	0.411224	-1.802329	0.730338
C	2.512473	-1.105506	-0.312292
H	2.170760	-1.223722	-1.372208
C	1.876193	-2.248716	0.524895
H	1.967621	-3.222218	0.029465
H	2.406841	-2.315832	1.487406
H	-0.032800	-2.189522	1.653689
C	-4.288970	-0.868049	0.576374
H	-4.493317	-1.950008	0.612133
H	-4.257499	-0.485596	1.603358
H	-5.147786	-0.411504	0.060392
C	-2.994564	-1.122856	-1.597285
H	-3.101823	-2.218922	-1.595647
H	-3.863029	-0.734016	-2.150635
H	-2.095137	-0.867075	-2.167952
C	-1.997290	0.078686	0.413548
H	-2.138540	0.371076	1.459066
H	-0.588753	0.010134	-1.193031
H	3.107466	1.619502	-0.581879
C	-0.602622	2.769791	0.986399
H	-1.634886	2.688457	1.346423
H	0.056673	2.425435	1.798258
H	-0.388581	3.833337	0.822002
H	-0.219101	-2.147846	-0.102389
C	4.047920	-1.022426	-0.321793
H	4.429013	-0.819299	0.687394
H	4.459224	-1.982845	-0.653699
H	4.419220	-0.245590	-1.001049

J-K-TS G = -585.973288, T = -576

C	-0.594601	1.775346	-0.017754
C	-0.770414	0.257130	-0.356067
C	-3.151829	-0.579201	0.025364
C	0.641529	2.343482	-0.748980
C	0.540352	-0.479415	-0.246901
C	1.801882	0.168870	-0.227573
C	1.954043	1.659895	-0.308373
H	1.110060	-0.145043	0.891184
H	0.518953	2.203279	-1.834364
H	-1.489081	2.276138	-0.417560
H	0.721211	3.424568	-0.573628
H	2.315644	2.038754	0.661432
C	0.733060	-1.977177	-0.359203
C	2.924509	-0.849644	-0.288755
H	3.203501	-0.851615	-1.362783
C	2.210874	-2.188457	0.048973
H	2.674194	-3.046588	-0.449396

H	2.274314	-2.364083	1.135278
H	0.002080	-2.550061	0.221882
C	-4.160889	-1.259976	0.920051
H	-4.613121	-2.122350	0.406036
H	-3.718667	-1.610502	1.860669
H	-4.988485	-0.574948	1.160808
C	-3.697225	-0.087109	-1.294437
H	-4.115119	-0.925178	-1.873174
H	-4.530424	0.610639	-1.119615
H	-2.963821	0.429616	-1.925758
C	-1.869758	-0.435053	0.422637
H	-1.596023	-0.808046	1.415138
H	-0.996279	0.184310	-1.437638
H	2.775459	1.862212	-1.013873
C	-0.555044	2.043676	1.497583
H	-1.483483	1.715752	1.978621
H	0.276272	1.526928	2.006404
H	-0.427923	3.116374	1.691343
H	0.565766	-2.222029	-1.423100
C	4.165509	-0.527053	0.550799
H	3.912208	-0.479542	1.619431
H	4.922382	-1.309683	0.419131
H	4.618400	0.429207	0.259353

K G = -585.975307

C	-0.635461	1.763035	-0.043942
C	-0.757758	0.223126	-0.262427
C	-3.150063	-0.616834	0.007840
C	0.594588	2.283388	-0.818006
C	0.527905	-0.477702	-0.163221
C	1.800054	0.201235	0.073618
C	1.925205	1.691796	-0.303880
H	1.722090	0.135367	1.201057
H	0.474455	2.029169	-1.884538
H	-1.540491	2.199115	-0.493225
H	0.635572	3.379297	-0.760824
H	2.292700	2.255731	0.562801
C	0.742901	-1.941189	-0.292074
C	2.912106	-0.826983	-0.291978
H	3.047842	-0.772311	-1.388221
C	2.231634	-2.168335	0.066916
H	2.662386	-3.027567	-0.458802
H	2.326292	-2.355807	1.148527
H	-0.008492	-2.551521	0.227284
C	-4.211181	-1.260411	0.867930
H	-4.652588	-2.125223	0.348693
H	-3.820711	-1.597693	1.835370
H	-5.036314	-0.555686	1.052399
C	-3.616257	-0.145703	-1.347013
H	-3.976085	-0.996880	-1.945374
H	-4.471821	0.536960	-1.236042

H	-2.849835	0.377690	-1.934117
C	-1.887805	-0.478243	0.471922
H	-1.654284	-0.844914	1.474959
H	-0.952905	0.072074	-1.354155
H	2.701534	1.777802	-1.078247
C	-0.626184	2.147374	1.445733
H	-1.547722	1.816950	1.939884
H	0.220611	1.708878	1.994174
H	-0.553144	3.236946	1.553471
H	0.576805	-2.138374	-1.374273
C	4.252017	-0.568458	0.396658
H	4.145866	-0.606811	1.490846
H	4.990232	-1.325229	0.102602
H	4.657815	0.415462	0.127289

Step K – L

K G = -585.975296

C	-0.635656	1.762560	-0.043047
C	-0.757776	0.222665	-0.262192
C	-3.150274	-0.616689	0.007564
C	0.593902	2.283546	-0.817432
C	0.528058	-0.477923	-0.163400
C	1.800049	0.201333	0.072813
C	1.924938	1.691887	-0.304382
H	1.721673	0.135227	1.200324
H	0.473257	2.029873	-1.884032
H	-1.540994	2.198657	-0.491686
H	0.634708	3.379432	-0.759701
H	2.293077	2.255594	0.562179
C	0.743350	-1.941400	-0.291745
C	2.912357	-0.826720	-0.292332
H	3.048115	-0.772401	-1.388590
C	2.232206	-2.168104	0.067060
H	2.663035	-3.027427	-0.458449
H	2.327036	-2.355232	1.148718
H	-0.007829	-2.551807	0.227821
C	-4.211489	-1.260877	0.867057
H	-4.652903	-2.125254	0.347092
H	-3.821099	-1.598983	1.834244
H	-5.036603	-0.556272	1.052043
C	-3.616439	-0.144059	-1.346790
H	-3.976743	-0.994501	-1.945908
H	-4.471687	0.538882	-1.235069
H	-2.849879	0.379556	-1.933511
C	-1.887909	-0.479014	0.471608
H	-1.654400	-0.846731	1.474271
H	-0.952639	0.072240	-1.354069
H	2.700710	1.778133	-1.079282
C	-0.625783	2.146162	1.446806

H	-1.546961	1.815145	1.941228
H	0.221440	1.707730	1.994647
H	-0.553084	3.235709	1.555044
H	0.577212	-2.138861	-1.373896
C	4.252173	-0.567577	0.396259
H	4.145991	-0.605510	1.490460
H	4.990594	-1.324277	0.102545
H	4.657727	0.416336	0.126502

K-L-TS G = -585.973128, T = -27

C	0.530950	1.915229	0.244182
C	0.764791	0.381758	0.410800
C	3.070947	-0.615314	-0.122198
C	-0.810330	2.286330	0.913558
C	-0.470551	-0.411549	0.295576
C	-1.753466	0.150377	-0.130758
C	-2.018080	1.637058	0.208531
H	-1.564823	0.072865	-1.240430
H	-0.787581	1.958334	1.967540
H	1.346684	2.404526	0.798361
H	-0.934725	3.377376	0.925782
H	-2.277004	2.186828	-0.704822
C	-0.609842	-1.862936	0.566215
C	-2.823634	-0.942505	0.168832
H	-3.109347	-0.825603	1.230847
C	-2.004390	-2.243441	0.012082
H	-2.443636	-3.099254	0.536374
H	-1.919699	-2.508620	-1.053997
H	0.253486	-2.467690	0.261450
C	4.039106	-1.144640	-1.153563
H	4.401676	-2.141975	-0.859257
H	3.593603	-1.216726	-2.152688
H	4.927277	-0.497499	-1.213511
C	3.610799	-0.528062	1.282704
H	3.770356	-1.537006	1.694696
H	4.593905	-0.035924	1.280451
H	2.965267	0.021167	1.980563
C	1.826507	-0.237264	-0.497336
H	1.542835	-0.342657	-1.547212
H	1.092517	0.183582	1.454175
H	-2.905373	1.676475	0.857217
C	0.616044	2.392374	-1.216882
H	1.615058	2.214731	-1.631955
H	-0.108790	1.888433	-1.872984
H	0.412540	3.469337	-1.272082
H	-0.636146	-1.910487	1.679054
C	-4.074758	-0.844558	-0.703880
H	-3.820999	-0.952734	-1.768748
H	-4.790620	-1.635152	-0.445755
H	-4.581015	0.120528	-0.570240

**L**	G = -585.982691		
C	-0.299533	2.123165	-0.856137
C	-0.687087	0.628121	-0.907310
C	-2.452506	-0.848078	0.351573
C	1.140445	2.301951	-0.320985
C	0.124790	-0.411247	-0.299380
C	1.280684	-0.056567	0.633652
C	1.416420	1.448062	0.924274
H	1.147218	-0.606775	1.583879
H	1.855104	2.027365	-1.112683
H	-0.306652	2.428317	-1.915606
H	1.311802	3.364115	-0.097745
H	0.729820	1.738096	1.736208
C	0.388622	-1.783395	-0.932058
C	2.466405	-0.754018	-0.099423
H	2.695552	-0.172462	-1.010821
C	1.849174	-2.106656	-0.519334
H	2.407932	-2.597376	-1.325677
H	1.853765	-2.788781	0.344705
H	-0.304480	-2.560169	-0.587286
C	-3.302979	-0.986766	1.575615
H	-3.487307	-2.047894	1.801608
H	-2.880723	-0.489486	2.454714
H	-4.295578	-0.550268	1.371251
C	-2.859804	-1.664192	-0.830827
H	-2.723226	-2.732710	-0.594213
H	-3.938874	-1.539725	-1.007955
H	-2.314305	-1.444939	-1.751249
C	-1.365216	0.018067	0.380388
H	-1.208495	0.580505	1.299712
H	-1.232675	0.332005	-1.808350
H	2.426035	1.645378	1.309497
C	-1.334518	3.001679	-0.131523
H	-2.354348	2.802236	-0.488182
H	-1.319289	2.847061	0.956143
H	-1.114752	4.060978	-0.312682
H	0.273938	-1.709840	-2.023145
C	3.733642	-0.881227	0.747007
H	3.531087	-1.431327	1.677675
H	4.509298	-1.427606	0.193895
H	4.147906	0.099912	1.013119

Cartesian coordinates of computed structures (Scheme 3 of main text, Table S11)

Gibbs energies (G in Hartree) and imagininary frequencies of TS (T in cm^{-1}) (wB97M-V/Def2-TZVPPD//B97D3/6-31G(d,p)-sp-density-fitting, 1 bar, 298.15 K)

Step C' – D'

**C'**	G = -585.946860		
C	-0.973557	-2.242439	-0.148386
C	-1.467503	-0.897539	-0.615169
C	-2.377528	0.030543	0.058495
C	-0.829341	0.390960	0.178828
C	0.446593	-2.596830	-0.690886
C	-0.148838	1.270374	-0.654304
C	2.012094	-0.666289	-0.861976
C	1.521687	-1.694660	-0.126140
H	1.690378	-0.597075	-1.907395
H	0.427559	-2.533984	-1.789329
H	-1.683167	-2.984373	-0.551765
C	1.918265	-1.961385	1.306255
H	2.756962	-1.346776	1.648502
H	1.077112	-1.787140	1.998634
H	2.197552	-3.018083	1.433799
H	0.646229	-3.646202	-0.426911
H	-0.390620	0.138100	1.143129
H	-1.389263	-0.759887	-1.698389
H	-0.593017	1.465929	-1.636114
H	-1.015919	-2.333718	0.944700
C	1.018960	2.126010	-0.297142
C	2.928171	0.417928	-0.350952
H	3.411932	0.928116	-1.196769
H	3.738906	-0.000088	0.263621
C	2.158455	1.457007	0.499455
H	2.843936	2.247051	0.838975
H	1.754209	0.982515	1.406757
H	1.434627	2.529329	-1.233843
C	0.400877	3.331221	0.495119
H	1.189375	4.078588	0.647410
H	0.030758	2.998027	1.472895
H	-0.423049	3.809448	-0.049108
C	-3.251003	0.940018	-0.796769
H	-4.225489	0.454671	-0.945376
H	-2.827474	1.130363	-1.790011
H	-3.425281	1.900371	-0.294829
C	-2.983411	-0.313375	1.414423
H	-3.267888	0.600317	1.950988
H	-2.309290	-0.895012	2.052858
H	-3.892594	-0.909744	1.252461

C‘-D‘-TS G = -585.934255, T = -356

C	-1.145867	-2.298240	0.020000
C	-1.521965	-0.949665	-0.522475
C	-2.312674	0.213295	0.019319
C	-0.786128	0.225434	0.134419
C	0.326500	-2.616392	-0.503785
C	0.261653	0.824767	-0.706680
C	1.663543	-0.469894	-0.867010
C	1.330084	-1.602410	-0.061741
H	1.523315	-0.678454	-1.934788
H	0.276830	-2.662488	-1.599878
H	-1.769961	-3.112725	-0.373584
C	1.922629	-1.825673	1.286872
H	2.380608	-0.944303	1.739999
H	1.193560	-2.275490	1.974808
H	2.717852	-2.585862	1.165292
H	0.611989	-3.605016	-0.115847
H	-0.460015	0.016075	1.159516
H	-1.452837	-0.925185	-1.616515
H	-0.054511	0.918911	-1.751926
H	-1.187809	-2.341737	1.116545
C	1.093635	2.033000	-0.231999
C	2.878860	0.391832	-0.496621
H	3.286534	0.820023	-1.423443
H	3.682767	-0.205421	-0.045536
C	2.387552	1.522914	0.420241
H	3.131592	2.322189	0.529200
H	2.166728	1.151684	1.434285
H	1.385234	2.579010	-1.144754
C	0.261893	2.970421	0.661994
H	0.852316	3.863986	0.903776
H	-0.017455	2.480801	1.604339
H	-0.657727	3.297359	0.160453
C	-3.006998	1.106505	-1.000800
H	-4.008310	0.716545	-1.230008
H	-2.453031	1.175308	-1.946062
H	-3.126001	2.122885	-0.600114
C	-3.059148	0.089140	1.341615
H	-3.202411	1.077697	1.799223
H	-2.529308	-0.544769	2.064388
H	-4.051367	-0.351225	1.170543

D‘ G = -585.939871

C	1.195903	2.319516	-0.020993
C	1.530691	0.955993	-0.549067
C	2.255432	-0.246940	0.041314
C	0.741220	-0.165191	0.096166
C	-0.305843	2.613383	-0.527271
C	-0.359931	-0.744993	-0.750077
C	-1.594275	0.400299	-0.845408
C	-1.259372	1.589637	-0.042650

H	-1.591068	0.706009	-1.903386
H	-0.268486	2.635854	-1.623519
H	1.799554	3.139179	-0.434558
C	-1.869415	1.834013	1.286129
H	-2.193608	0.929967	1.807951
H	-1.231274	2.458618	1.923638
H	-2.783415	2.432091	1.094561
H	-0.590704	3.602414	-0.139476
H	0.399493	0.094174	1.108785
H	1.506475	0.926259	-1.644435
H	0.004944	-0.898503	-1.774250
H	1.236442	2.380031	1.074504
C	-1.099425	-2.021620	-0.242618
C	-2.864309	-0.382966	-0.430008
H	-3.315955	-0.789306	-1.346705
H	-3.626670	0.252802	0.038827
C	-2.377010	-1.537611	0.458154
H	-3.126724	-2.332297	0.560295
H	-2.139241	-1.192321	1.478526
H	-1.413339	-2.572827	-1.145275
C	-0.197966	-2.932606	0.599850
H	-0.743425	-3.845417	0.874346
H	0.121612	-2.436687	1.526420
H	0.701609	-3.231893	0.047038
C	2.939221	-1.175378	-0.953218
H	3.950453	-0.814115	-1.186579
H	2.387378	-1.257600	-1.898713
H	3.035329	-2.184191	-0.527376
C	2.987018	-0.123560	1.371422
H	3.077949	-1.108329	1.851086
H	2.470260	0.544463	2.073402
H	4.000775	0.269768	1.214544

Step D’ – E’

D‘ G = -585.935890

C	0.163719	-2.443503	0.486692
C	-0.763778	-1.568812	-0.312734
C	-2.092755	-0.904717	-0.004998
C	-0.789212	-0.134985	0.145340
C	1.604392	-2.098291	0.037215
C	-0.043468	0.830116	-0.736274
C	1.475644	0.553255	-0.540405
C	2.131377	-0.737183	-0.301555
H	2.063787	1.103081	-1.292414
H	1.745373	-2.545874	-0.980096
H	0.022855	-3.524157	0.326969
C	3.626883	-0.712347	-0.404402
H	4.022926	-0.641352	0.625183
H	4.024726	-1.654915	-0.803644

H	4.013631	0.136891	-0.975410
H	2.368674	-2.624693	0.634243
H	-0.516961	-0.029173	1.202681
H	-0.598149	-1.676587	-1.392447
H	-0.314028	0.680022	-1.792765
H	0.062515	-2.265736	1.567271
C	-0.145503	2.321722	-0.331145
C	1.828439	1.338790	0.860751
H	2.836661	1.758263	0.775778
H	1.819872	0.654009	1.718989
C	0.698821	2.402174	0.962082
H	1.139649	3.397929	1.106495
H	0.072466	2.189525	1.838802
H	0.361215	2.918230	-1.110175
C	-1.581580	2.827549	-0.178533
H	-1.595742	3.882763	0.124134
H	-2.122011	2.248069	0.582823
H	-2.132407	2.741261	-1.124297
C	-3.005894	-0.629608	-1.192756
H	-3.649865	-1.496989	-1.394925
H	-2.444724	-0.408980	-2.110604
H	-3.657052	0.230136	-0.980999
C	-2.839856	-1.231446	1.281117
H	-3.510707	-0.405805	1.558752
H	-2.160518	-1.410201	2.125477
H	-3.455566	-2.131996	1.147809

D‘-E‘-TS G = -585.926062, T = -641

C	-0.046393	-2.570122	0.129709
C	-0.984384	-1.502758	-0.380358
C	-2.176681	-0.726245	0.141479
C	-0.773738	-0.127795	0.203655
C	1.318884	-2.273877	-0.563365
C	0.052520	0.796018	-0.678314
C	1.492475	0.377988	-0.279276
C	2.033321	-0.949591	-0.210804
H	2.179352	-0.204081	-1.280493
H	1.150870	-2.299904	-1.652971
H	-0.335907	-3.592431	-0.157141
C	3.413784	-1.153865	0.385630
H	3.270318	-1.522841	1.413917
H	3.956210	-1.934611	-0.160772
H	4.031402	-0.253579	0.424899
H	2.037035	-3.076480	-0.352083
H	-0.391087	-0.151803	1.231970
H	-0.977171	-1.492013	-1.478261
H	-0.155290	0.567228	-1.735261
H	0.053553	-2.550978	1.224522
C	0.009779	2.328751	-0.422016
C	2.214132	1.591951	0.302919
H	2.842252	2.054469	-0.476459

H	2.865804	1.333601	1.144649
C	1.062691	2.551056	0.678519
H	1.405696	3.590243	0.745309
H	0.650957	2.275695	1.662457
H	0.373719	2.823003	-1.340193
C	-1.385413	2.861396	-0.091574
H	-1.350001	3.944583	0.083378
H	-1.786291	2.383365	0.812623
H	-2.089014	2.676218	-0.913105
C	-3.177537	-0.247873	-0.901188
H	-3.912883	-1.035023	-1.119297
H	-2.692609	0.029413	-1.847003
H	-3.725041	0.629985	-0.531363
C	-2.801183	-1.072362	1.486632
H	-3.311715	-0.198866	1.916637
H	-2.055912	-1.422212	2.213263
H	-3.547791	-1.869007	1.360892

E‘ G = -585.938292

C	0.966736	-2.362991	-0.025894
C	-0.335036	-1.687614	-0.398275
C	-1.661271	-1.434201	0.240759
C	-0.536396	-0.292645	0.151010
C	2.026317	-1.621523	-0.875681
C	-0.235111	0.857367	-0.824086
C	1.151407	0.815942	-0.254611
C	2.307675	-0.116340	-0.510614
H	2.742411	0.326788	-1.435742
H	1.722345	-1.689548	-1.931284
H	0.974783	-3.427295	-0.303396
C	3.395765	-0.029087	0.586040
H	3.030470	-0.434801	1.539524
H	4.262960	-0.625256	0.281058
H	3.749539	0.993614	0.757850
H	2.996808	-2.128995	-0.803451
H	-0.205327	-0.130513	1.183728
H	-0.449183	-1.686280	-1.489665
H	-0.356793	0.509842	-1.856517
H	1.179707	-2.301233	1.051525
C	-0.818802	2.250588	-0.509814
C	1.286381	1.971591	0.694270
H	2.010461	2.637027	0.175741
H	1.766410	1.718183	1.648597
C	-0.098800	2.640424	0.800361
H	-0.028573	3.724641	0.943780
H	-0.642554	2.233216	1.666683
H	-0.477186	2.927350	-1.312930
C	-2.346417	2.289150	-0.433728
H	-2.694298	3.305629	-0.209898
H	-2.714697	1.624556	0.360061
H	-2.805858	1.978808	-1.381418

C	-2.868569	-1.318926	-0.667938
H	-3.303838	-2.318051	-0.821241
H	-2.613610	-0.909867	-1.653382
H	-3.641388	-0.684155	-0.215892
C	-1.959815	-1.890290	1.656013
H	-2.687374	-1.228394	2.144928
H	-1.058309	-1.946745	2.278443
H	-2.401292	-2.897191	1.616029

Step E' – F'

E' G = -585.939868

C	1.725285	1.978232	-0.106486
C	1.708601	0.535508	-0.557632
C	2.251105	-0.786005	-0.052846
C	0.811790	-0.381494	0.241334
C	0.388639	2.577532	-0.620421
C	-0.511478	-0.662823	-0.416556
C	-1.365383	0.547152	-0.437783
C	-0.978286	1.957056	-0.164866
H	-1.770710	2.590789	-0.596624
H	0.398934	2.554685	-1.721524
H	2.550933	2.564039	-0.538400
C	-1.171643	2.099726	1.405324
H	-0.486841	1.463042	1.970984
H	-0.944496	3.147112	1.639434
H	-2.200391	1.887459	1.713942
H	0.318662	3.638194	-0.336124
H	0.684892	-0.073477	1.282149
H	1.506492	0.480972	-1.635460
H	-0.352972	-0.786996	-1.521885
H	1.809018	2.063536	0.986826
C	-1.480897	-1.836357	-0.032771
C	-2.783459	0.141869	-0.618489
H	-3.257482	0.707170	-1.439867
H	-3.331442	0.482764	0.281902
C	-2.787354	-1.401104	-0.743697
H	-2.758960	-1.685578	-1.805734
H	-3.685138	-1.848847	-0.302245
H	-1.087844	-2.770719	-0.454933
C	-1.622035	-1.988772	1.487337
H	-2.304558	-2.815300	1.722004
H	-2.023816	-1.080631	1.963370
H	-0.653494	-2.209445	1.953762
C	2.541428	-1.847627	-1.104780
H	3.554071	-1.724229	-1.513338
H	1.838257	-1.800406	-1.949259
H	2.475757	-2.852860	-0.664955
C	3.180113	-0.831639	1.153405
H	3.122844	-1.807010	1.657106

H	2.939800	-0.053003	1.889481
H	4.219928	-0.678787	0.832951

E‘-F‘-TS G = -585.935382, T = -826

C	-1.748318	-1.953440	-0.282658
C	-1.670688	-0.480824	-0.619947
C	-2.203999	0.821132	-0.045655
C	-0.813945	0.339673	0.329475
C	-0.381039	-2.558844	-0.713906
C	0.567393	0.573373	-0.136984
C	1.391199	-0.589717	-0.282126
C	0.959762	-2.036745	-0.086375
H	1.747617	-2.639967	-0.565009
H	-0.292761	-2.457575	-1.808186
H	-2.544619	-2.478238	-0.831317
C	1.052668	-2.328163	1.441186
H	0.289622	-1.791116	2.015525
H	0.894540	-3.402676	1.596189
H	2.037738	-2.070731	1.849762
H	-0.385001	-3.639363	-0.509363
H	-0.781060	-0.099250	1.331474
H	-1.385900	-0.341496	-1.672339
H	0.697153	0.072049	-1.307372
H	-1.937582	-2.112068	0.788886
C	1.417359	1.838968	-0.140110
C	2.826328	-0.161412	-0.467334
H	3.369530	-0.760579	-1.209615
H	3.318224	-0.345158	0.505069
C	2.747290	1.355200	-0.784011
H	2.707092	1.495612	-1.874721
H	3.617389	1.907065	-0.411429
H	0.937318	2.634090	-0.727107
C	1.561870	2.317286	1.321966
H	2.191080	3.215161	1.351025
H	2.033842	1.550427	1.952682
H	0.585860	2.566979	1.756420
C	-2.367743	1.972873	-1.028384
H	-3.352016	1.931765	-1.514392
H	-1.603079	1.954056	-1.818413
H	-2.291335	2.936317	-0.505038
C	-3.229915	0.802381	1.080345
H	-3.184457	1.730551	1.666994
H	-3.076849	-0.041956	1.765329
H	-4.241678	0.715655	0.662053

F‘ G = -585.946063

C	1.568152	1.943629	-0.605849
C	1.518179	0.442253	-0.782588
C	2.140778	-0.706934	0.034205
C	0.766305	-0.255817	0.427207
C	0.156625	2.567110	-0.826646

C	-0.530288	-0.540994	-0.072101
C	-1.460243	0.592648	-0.340611
C	-1.070829	2.055123	0.001399
H	-1.914428	2.644504	-0.396987
H	-0.098144	2.456386	-1.893971
H	2.258432	2.387537	-1.340688
C	-1.016915	2.384708	1.506233
H	-0.171098	1.928716	2.037720
H	-0.925739	3.469764	1.643562
H	-1.935634	2.062143	2.013104
H	0.245335	3.648465	-0.647916
H	0.751398	0.487997	1.225898
H	1.196626	0.111761	-1.776392
H	-1.427530	0.550755	-1.460092
H	1.954791	2.196809	0.391311
C	-1.219219	-1.848871	-0.326741
C	-2.848192	-0.019237	-0.019379
H	-3.666625	0.553182	-0.470473
H	-3.014610	-0.052239	1.067873
C	-2.700362	-1.444029	-0.613214
H	-2.882811	-1.412330	-1.697253
H	-3.401226	-2.168533	-0.181881
H	-0.757149	-2.403879	-1.159865
C	-1.058784	-2.724012	0.953475
H	-1.586225	-3.672239	0.795127
H	-1.498898	-2.229281	1.828920
H	-0.003396	-2.932167	1.163663
C	2.340960	-2.011146	-0.727117
H	3.305272	-1.998884	-1.251335
H	1.555712	-2.184447	-1.473866
H	2.346806	-2.861152	-0.031740
C	3.253642	-0.378786	1.023240
H	3.348763	-1.186175	1.761609
H	3.070932	0.554772	1.569994
H	4.210581	-0.285335	0.494592

Step F' – G'

F‘ G = -585.946058

C	-1.568931	-1.943331	-0.605426
C	-1.518464	-0.442014	-0.782398
C	-2.140600	0.707660	0.034153
C	-0.766204	0.256176	0.426914
C	-0.157593	-2.567152	-0.826609
C	0.530626	0.540963	-0.072167
C	1.459992	-0.593080	-0.340722
C	1.070052	-2.055455	0.001307
H	1.913538	-2.645023	-0.397021
H	0.096942	-2.456425	-1.893990
H	-2.259551	-2.387142	-1.339997

C	1.015869	-2.385080	1.506133
H	0.169366	-1.929898	2.037222
H	0.925487	-3.470221	1.643343
H	1.934088	-2.061824	2.013459
H	-0.246500	-3.648499	-0.647929
H	-0.751283	-0.487364	1.225888
H	-1.197020	-0.111905	-1.776344
H	1.427311	-0.551161	-1.460213
H	-1.955332	-2.196302	0.391881
C	1.220078	1.848553	-0.326566
C	2.848313	0.018135	-0.019482
H	3.666433	-0.554574	-0.470787
H	3.014837	0.050729	1.067765
C	2.701159	1.443171	-0.612867
H	2.883816	1.411773	-1.696873
H	3.402247	2.167229	-0.181142
H	0.758232	2.403455	-1.159926
C	1.059739	2.723989	0.953347
H	1.587163	3.672187	0.794752
H	1.499942	2.229479	1.828882
H	0.004393	2.932259	1.163665
C	-2.340426	2.011687	-0.727567
H	-3.304947	1.999770	-1.251413
H	-1.555365	2.184279	-1.474686
H	-2.345541	2.861954	-0.032505
C	-3.253405	0.380122	1.023446
H	-3.348338	1.187824	1.761499
H	-3.070744	-0.553239	1.570557
H	-4.210411	0.286587	0.494936

F‘-G‘-TS G = -585.941274, T = -354

C	-0.417393	-2.079593	-0.987107
C	-0.869027	-0.625165	-0.829654
C	-2.178192	-0.162386	0.213596
C	-0.813876	-0.068247	0.692765
C	1.118520	-2.313167	-0.968602
C	0.189250	0.529548	-0.151432
C	1.623341	0.014507	-0.252312
C	1.919266	-1.465156	0.056503
H	2.980482	-1.594518	-0.212921
H	1.516876	-2.094717	-1.971006
H	-0.832085	-2.470375	-1.926060
C	1.791833	-1.936015	1.519538
H	0.763403	-2.186351	1.816311
H	2.376268	-2.852949	1.668475
H	2.172671	-1.183426	2.222364
H	1.290098	-3.384620	-0.793690
H	-0.518320	-0.881152	1.357467
H	-1.202473	-0.061842	-1.698709
H	1.799741	0.081797	-1.346574
H	-0.882400	-2.666092	-0.182851

C	0.236156	2.058335	-0.457456
C	2.433046	1.190970	0.307392
H	3.506834	1.099059	0.103714
H	2.299159	1.293320	1.395765
C	1.776148	2.364763	-0.456730
H	2.166707	2.390151	-1.483969
H	1.973642	3.344713	-0.005562
H	-0.206500	2.300941	-1.435688
C	-0.442145	2.882736	0.655476
H	-0.361378	3.950658	0.417893
H	0.067205	2.716552	1.615044
H	-1.502247	2.652512	0.799657
C	-2.995625	0.956077	-0.411545
H	-3.734395	0.528408	-1.101128
H	-2.397523	1.695023	-0.950153
H	-3.549921	1.475848	0.382196
C	-3.026827	-1.270126	0.808599
H	-3.686569	-0.837568	1.573722
H	-2.436778	-2.061725	1.282910
H	-3.669346	-1.716126	0.038607

G' G = -585.963589

C	0.819102	-1.841571	-1.332660
C	-0.187723	-0.679081	-1.077013
C	-2.306475	-0.898182	0.427426
C	-0.949859	-0.831850	0.263734
C	2.299562	-1.474039	-1.096913
C	0.383706	0.596665	-0.629960
C	1.730137	0.680920	-0.000401
C	2.536257	-0.626548	0.176263
H	3.594627	-0.321182	0.180255
H	2.671718	-0.906815	-1.966379
H	0.692050	-2.184687	-2.368404
C	2.309896	-1.404697	1.485316
H	1.340240	-1.916534	1.526352
H	3.078453	-2.182562	1.579169
H	2.389823	-0.754610	2.365914
H	2.892673	-2.397703	-1.050985
H	-0.333115	-1.003089	1.144378
H	-0.916509	-0.554491	-1.888352
H	2.259697	1.215491	-0.828374
H	0.528557	-2.685515	-0.692571
C	-0.204328	1.975107	-0.660129
C	1.550924	1.736745	1.116800
H	2.498660	2.219498	1.381558
H	1.162551	1.250328	2.022983
C	0.500439	2.724602	0.527179
H	0.972000	3.648971	0.171311
H	-0.247125	3.012553	1.277585
H	0.214090	2.362252	-1.617245
C	-1.718500	2.173596	-0.748511

H	-1.936376	3.243038	-0.856900
H	-2.204912	1.816362	0.167409
H	-2.154752	1.655867	-1.610430
C	-3.328077	-0.803077	-0.670907
H	-3.914330	-1.734270	-0.699587
H	-2.908593	-0.629619	-1.665939
H	-4.046156	0.001953	-0.455785
C	-2.859585	-1.209647	1.795134
H	-3.585475	-0.435389	2.089946
H	-2.083319	-1.275122	2.565838
H	-3.417335	-2.158390	1.776102

Step G’ – I’

G‘ G = -585.968107

C	-0.242373	-1.830702	1.565423
C	0.480324	-0.526616	1.155126
C	2.658596	-0.258917	-0.217285
C	1.384832	-0.714688	-0.076148
C	-1.113403	-2.415984	0.434335
C	-0.461598	0.470153	0.616094
C	-1.826262	0.054008	0.249348
C	-2.064536	-1.386680	-0.263355
H	-3.104056	-1.631701	0.001799
H	-1.704225	-3.247240	0.840640
H	-0.858244	-1.616081	2.453858
C	-1.954913	-1.454916	-1.798984
H	-0.970500	-1.112806	-2.152140
H	-2.091434	-2.490791	-2.134517
H	-2.722044	-0.844062	-2.290828
H	-0.455026	-2.864903	-0.323382
H	0.964368	-1.321680	-0.877359
H	1.074226	-0.107455	1.979553
H	-2.182918	0.047135	1.321747
H	0.512016	-2.567028	1.872703
C	-0.312983	1.954550	0.479954
C	-2.505801	1.287587	-0.380671
H	-3.592433	1.281921	-0.238124
H	-2.311607	1.324664	-1.461892
C	-1.794494	2.440715	0.356995
H	-2.227717	2.569834	1.360581
H	-1.857591	3.402433	-0.164475
H	0.230854	2.382074	1.336647
C	0.474694	2.330242	-0.818980
H	0.409168	3.417133	-0.948433
H	0.045643	1.846548	-1.704397
H	1.522824	2.034428	-0.740359
C	3.382898	0.603746	0.782697
H	4.228232	0.043779	1.212347
H	2.756119	0.966769	1.603915

H	3.824797	1.478368	0.282040
C	3.465695	-0.669312	-1.423455
H	4.381342	-1.193864	-1.110874
H	3.796621	0.222695	-1.978599
H	2.907526	-1.319275	-2.107007

G‘-I‘-TS G = -585.927982, T = -259

C	-1.672433	-0.582778	1.971919
C	-0.420222	-0.651952	1.203998
C	2.266507	-0.991093	-0.265181
C	1.180016	-0.262880	-0.607725
C	-2.737752	-1.128165	0.903198
C	0.163071	0.303132	0.347112
C	-1.578488	0.612721	-0.425193
C	-2.451911	-0.609715	-0.545757
H	-3.407122	-0.217595	-0.948556
H	-3.721599	-0.801331	1.265517
H	-1.938272	0.419465	2.325399
C	-1.936405	-1.689916	-1.505296
H	-0.972070	-2.094490	-1.166296
H	-2.650534	-2.520123	-1.568838
H	-1.793930	-1.291451	-2.517711
H	-2.729307	-2.225634	0.917137
H	1.058735	-0.004928	-1.661341
H	-0.097687	-1.681658	1.010230
H	-1.963646	1.235673	0.389056
H	-1.664186	-1.276122	2.822627
C	0.321538	1.840645	0.553355
C	-1.094199	1.533768	-1.515733
H	-1.971340	2.096918	-1.878082
H	-0.690944	0.992431	-2.380479
C	-0.039652	2.459268	-0.836055
H	-0.413956	3.482317	-0.704254
H	0.861682	2.527642	-1.458656
H	-0.410564	2.172003	1.306816
C	1.718772	2.229563	1.049466
H	1.773743	3.322813	1.140811
H	2.497904	1.904193	0.349927
H	1.931560	1.797834	2.035550
C	2.646707	-1.351294	1.153178
H	2.604003	-2.441138	1.305325
H	2.024148	-0.872600	1.920380
H	3.689037	-1.055467	1.343858
C	3.222536	-1.480637	-1.326700
H	3.333872	-2.574499	-1.269645
H	4.226677	-1.058932	-1.165965
H	2.895288	-1.219324	-2.340327

I‘ G = -585.979207

C	-0.788061	-1.225883	1.452953
C	-0.470058	-0.287467	0.245211

C	2.568396	-1.180246	-0.272955
C	2.089454	0.067441	0.135620
C	-2.265769	-1.630511	1.174312
C	0.767561	0.539860	0.282074
C	-1.770018	0.524771	0.148648
C	-2.865545	-0.571955	0.179943
H	-3.792169	-0.146621	0.592185
H	-2.828644	-1.675999	2.115447
H	-0.695116	-0.640826	2.379220
C	-3.192162	-1.175955	-1.194404
H	-2.302967	-1.608402	-1.679618
H	-3.927191	-1.984853	-1.089668
H	-3.615707	-0.426889	-1.876497
H	-2.305355	-2.633976	0.727905
H	2.872023	0.801192	0.333330
H	-0.404551	-0.937766	-0.647773
H	-1.880814	1.086864	1.094029
H	-0.114149	-2.085475	1.545382
C	0.515543	2.006726	0.435283
C	-1.670390	1.541057	-0.989451
H	-2.588467	2.139533	-1.062105
H	-1.558415	1.011495	-1.947430
C	-0.442289	2.464428	-0.746822
H	-0.758922	3.482228	-0.482837
H	0.173864	2.549035	-1.652895
H	-0.103795	2.100447	1.349619
C	1.720647	2.943803	0.555171
H	1.368506	3.972946	0.694999
H	2.338735	2.930650	-0.353210
H	2.354459	2.693815	1.416248
C	1.742913	-2.342396	-0.735713
H	1.001442	-2.036818	-1.488199
H	1.175917	-2.772408	0.101800
H	2.370882	-3.131532	-1.161541
C	4.047855	-1.384119	-0.315555
H	4.360381	-1.590757	-1.353654
H	4.307870	-2.295612	0.247910
H	4.623732	-0.537591	0.071470

Step I’ – J’

I‘ G = -585.978674

C	0.279034	-1.826415	0.350212
C	0.444004	-0.296352	0.319676
C	-2.811341	-0.616760	-0.143370
C	-1.895897	0.323759	-0.587399
C	1.626503	-2.414990	-0.176490
C	-0.609048	0.644209	-0.076174
C	1.579334	-0.077287	-0.813785
C	2.567078	-1.208422	-0.427480

H	3.218513	-1.397422	-1.296055
H	1.449404	-2.959971	-1.113047
H	-0.547954	-2.135837	-0.302671
C	3.448720	-0.891571	0.788105
H	2.858025	-0.741373	1.704795
H	4.124036	-1.734750	0.982838
H	4.069744	0.000741	0.637247
H	2.065523	-3.128312	0.532543
H	-2.265567	1.004537	-1.363216
H	0.924419	0.074945	1.242756
H	1.102395	-0.372628	-1.762907
H	0.044828	-2.182006	1.360902
C	-0.204365	2.099121	-0.104969
C	2.085868	1.373839	-0.922057
H	1.963056	1.724909	-1.956785
H	3.159041	1.430384	-0.697461
C	1.312759	2.312889	0.023016
H	1.544506	3.364864	-0.191637
H	1.623675	2.138188	1.065353
H	-0.560437	2.519738	-1.063322
C	-0.984141	2.836778	1.027967
H	-0.700290	3.896094	1.017700
H	-0.727159	2.421903	2.011748
H	-2.068597	2.760692	0.887196
C	-2.709912	-1.362363	1.152990
H	-1.837006	-1.095477	1.751104
H	-2.718880	-2.448853	0.977852
H	-3.613157	-1.149681	1.746319
C	-4.093132	-0.796865	-0.900652
H	-4.943766	-0.429033	-0.304207
H	-4.282337	-1.870821	-1.054363
H	-4.094801	-0.288256	-1.870227

I'-J'-TS G = -585.958551, T = -418

C	0.491929	-1.573675	1.146104
C	0.529529	-0.059717	0.871989
C	-2.522495	-0.729500	-0.226125
C	-1.436155	-0.275343	-0.900297
C	1.128884	-2.226412	-0.102865
C	-0.307038	0.491055	-0.297432
C	1.564992	0.141601	-0.210705
C	2.172221	-1.197985	-0.611272
H	2.335070	-1.263411	-1.696885
H	0.360675	-2.390669	-0.872175
H	-0.516647	-1.949574	1.341861
C	3.542897	-1.322723	0.098124
H	3.448977	-1.211003	1.186673
H	3.954038	-2.319397	-0.105081
H	4.259445	-0.577384	-0.267553
H	1.588113	-3.197608	0.116889
H	-1.436876	-0.361696	-1.989028

H	0.580507	0.604786	1.737324
H	0.586296	0.265594	-1.167589
H	1.096727	-1.768832	2.043070
C	-0.311437	2.035936	-0.400170
C	2.175818	1.511605	-0.387496
H	2.559844	1.629443	-1.410818
H	3.037895	1.588486	0.292014
C	1.097688	2.560324	-0.016817
H	1.299525	3.520868	-0.505826
H	1.131000	2.746886	1.065856
H	-0.527368	2.278747	-1.453680
C	-1.420806	2.648564	0.469096
H	-1.398699	3.741735	0.377495
H	-1.282470	2.397504	1.529669
H	-2.410232	2.298241	0.153075
C	-2.766641	-0.563855	1.253556
H	-1.941051	-0.098216	1.798892
H	-2.993031	-1.535520	1.717293
H	-3.660866	0.060151	1.404384
C	-3.643827	-1.395631	-0.985750
H	-4.583368	-0.837669	-0.855111
H	-3.824530	-2.403955	-0.581950
H	-3.436323	-1.482973	-2.058431

J' G = -585.963490

C	0.606514	-1.730310	0.650989
C	0.526170	-0.196352	0.570796
C	-2.624968	-0.794871	-0.075918
C	-1.647460	-0.375579	-0.923148
C	1.612848	-2.099687	-0.462635
C	-0.398229	0.420262	-0.671583
C	1.772355	0.249596	-0.018126
C	2.590377	-0.889497	-0.516370
H	3.012332	-0.691309	-1.513731
H	1.093592	-2.193620	-1.427853
H	-0.369732	-2.207772	0.519666
C	3.795017	-1.051874	0.475359
H	3.451495	-1.218465	1.503881
H	4.372490	-1.928477	0.157852
H	4.451148	-0.173803	0.457824
H	2.136676	-3.043838	-0.273791
H	-1.777939	-0.651029	-1.972800
H	0.197781	0.341043	1.467861
H	0.195145	0.282053	-1.593730
H	0.990792	-2.013056	1.641979
C	-0.504426	1.969882	-0.482759
C	2.054523	1.688357	-0.203943
H	2.472181	1.850506	-1.212496
H	2.883533	1.955890	0.480445
C	0.814277	2.549712	0.109722
H	0.977494	3.573537	-0.249838

H	0.709245	2.623161	1.202890
H	-0.627088	2.369599	-1.502604
C	-1.704038	2.438312	0.349202
H	-1.731403	3.535668	0.375210
H	-1.641998	2.088859	1.388967
H	-2.648517	2.082527	-0.078119
C	-2.663401	-0.615825	1.420089
H	-1.759516	-0.186471	1.856807
H	-2.856716	-1.582878	1.908234
H	-3.506212	0.037377	1.693605
C	-3.825654	-1.516067	-0.641877
H	-4.753326	-0.993595	-0.362339
H	-3.902118	-2.527828	-0.212796
H	-3.792774	-1.604427	-1.733903

Step J’ – M’

J‘ G = -585.971196

C	0.295202	2.121954	0.379617
C	0.512938	0.588346	0.201131
C	2.685759	-0.735295	0.206617
C	-1.209592	2.510556	0.288649
C	-0.677208	0.026607	-0.892558
C	-1.732644	0.083769	0.080700
C	-2.154292	1.363180	0.689831
H	-0.652700	0.792516	-1.676889
H	-1.412218	3.392638	0.909036
H	0.642296	2.336300	1.403533
H	-1.437804	2.805942	-0.746829
H	-3.183601	1.579322	0.343457
C	-0.538229	-1.441013	-1.319965
C	-2.181941	-1.278277	0.491174
H	-2.287178	-1.362147	1.584374
C	-1.103972	-2.224849	-0.114116
H	-0.313104	-2.404022	0.628658
H	-1.522445	-3.198953	-0.393149
H	-1.148667	-1.603991	-2.220517
C	3.990876	-1.040559	-0.484938
H	4.086269	-2.123986	-0.659895
H	4.091800	-0.521529	-1.445115
H	4.840427	-0.758169	0.155782
C	2.483660	-1.437033	1.525114
H	2.456232	-2.526993	1.368511
H	3.341138	-1.247092	2.187998
H	1.573601	-1.146585	2.061388
C	1.807701	0.135476	-0.361114
H	2.063990	0.544316	-1.342111
H	0.331756	0.097923	1.168197
H	-2.257051	1.230151	1.781023
C	1.115242	2.987336	-0.588999

H	2.192769	2.835362	-0.453648
H	0.867657	2.769583	-1.639009
H	0.898539	4.049493	-0.416229
H	0.495420	-1.710344	-1.564330
C	-3.599446	-1.508202	-0.133458
H	-4.336035	-0.802708	0.268996
H	-3.577195	-1.411743	-1.226231
H	-3.918225	-2.527262	0.116889

J‘-M‘-TS G = -585.967139, T = -332

C	0.251300	2.198866	0.411509
C	0.572655	0.693297	0.319439
C	2.643683	-0.741926	0.229620
C	-1.268014	2.508588	0.245272
C	-0.713395	0.013017	-0.981689
C	-1.695426	0.060801	0.023283
C	-2.171736	1.328917	0.633563
H	-0.587086	0.823787	-1.702410
H	-1.534526	3.396544	0.832011
H	0.530061	2.457961	1.448271
H	-1.457015	2.769479	-0.807258
H	-3.205664	1.517444	0.290703
C	-0.473287	-1.430998	-1.386075
C	-2.090195	-1.318245	0.471347
H	-2.097681	-1.392087	1.571304
C	-1.027080	-2.244914	-0.190450
H	-0.222071	-2.457118	0.526704
H	-1.454873	-3.207135	-0.495757
H	-1.038694	-1.630463	-2.310171
C	3.945142	-1.048350	-0.463340
H	3.983316	-2.111088	-0.750846
H	4.103009	-0.436901	-1.358968
H	4.791143	-0.889535	0.223325
C	2.369606	-1.557726	1.464684
H	2.336835	-2.627672	1.206470
H	3.200732	-1.445001	2.177411
H	1.441332	-1.301187	1.985425
C	1.831733	0.243799	-0.259331
H	2.153868	0.748931	-1.172535
H	0.322363	0.176833	1.251518
H	-2.257802	1.193288	1.725245
C	1.073737	3.087026	-0.534523
H	2.150016	3.001495	-0.342147
H	0.891116	2.835594	-1.590048
H	0.789255	4.137934	-0.396465
H	0.582670	-1.638047	-1.602320
C	-3.538272	-1.603000	-0.027726
H	-4.262458	-0.908791	0.416202
H	-3.603882	-1.525510	-1.121009
H	-3.816657	-2.623672	0.261841

M‘ G = -585.963209

C	-0.405194	2.304397	-0.454049
C	-0.752550	0.834547	-0.513788
C	-2.482725	-0.940201	-0.307146
C	1.087344	2.584834	-0.070764
C	0.754104	-0.016744	1.217342
C	1.602742	0.134462	0.147121
C	2.041991	1.443685	-0.431659
H	0.420702	0.795346	1.863788
H	1.409770	3.519586	-0.546629
H	-0.514567	2.619588	-1.511114
H	1.126632	2.764075	1.014408
H	3.055697	1.681739	-0.064967
C	0.458434	-1.454414	1.513305
C	2.022219	-1.204673	-0.421177
H	1.675761	-1.268566	-1.471159
C	1.294616	-2.258345	0.474832
H	0.661363	-2.921667	-0.127381
H	2.028920	-2.898284	0.981174
H	0.712675	-1.699966	2.556369
C	-3.767248	-1.380024	0.331730
H	-3.645633	-2.372031	0.794358
H	-4.132164	-0.676727	1.087885
H	-4.545546	-1.505411	-0.438539
C	-1.925764	-1.865644	-1.349909
H	-1.842716	-2.883264	-0.941339
H	-2.635479	-1.936520	-2.189718
H	-0.950729	-1.571069	-1.746825
C	-1.929768	0.279786	0.044011
H	-2.448009	0.854758	0.812976
H	-0.294409	0.289941	-1.340617
H	2.150383	1.334425	-1.523889
C	-1.343564	3.165388	0.404793
H	-2.383937	3.108208	0.061175
H	-1.308878	2.865732	1.462372
H	-1.031452	4.215808	0.353058
H	-0.628915	-1.644301	1.426906
C	3.559354	-1.365307	-0.444770
H	4.036426	-0.612213	-1.085612
H	3.975590	-1.273213	0.567848
H	3.824569	-2.356450	-0.833753

Step M’ – N’

M‘ G = -585.963210

C	-0.405505	2.304363	-0.453991
C	-0.752747	0.834494	-0.513806
C	-2.482668	-0.940488	-0.307155
C	1.087027	2.584922	-0.070773
C	0.754053	-0.016694	1.217338

C	1.602729	0.134612	0.147172
C	2.041753	1.443859	-0.431718
H	0.420460	0.795372	1.863726
H	1.409348	3.519709	-0.546643
H	-0.514984	2.619578	-1.511048
H	1.126351	2.764156	1.014401
H	3.055506	1.682041	-0.065247
C	0.458607	-1.454407	1.513338
C	2.022432	-1.204476	-0.421090
H	1.675701	-1.268511	-1.470984
C	1.295238	-2.258227	0.475157
H	0.662386	-2.922076	-0.126902
H	2.029840	-2.897597	0.981797
H	0.712642	-1.699829	2.556488
C	-3.767176	-1.380437	0.331632
H	-3.645596	-2.372572	0.793990
H	-4.132074	-0.677311	1.087955
H	-4.545479	-1.505590	-0.438674
C	-1.925552	-1.865793	-1.349962
H	-1.842859	-2.883557	-0.941706
H	-2.635011	-1.936227	-2.190039
H	-0.950346	-1.571278	-1.746490
C	-1.929896	0.279611	0.043966
H	-2.448235	0.854542	0.812902
H	-0.294481	0.289966	-1.340616
H	2.149973	1.334538	-1.523969
C	-1.343917	3.165217	0.404924
H	-2.384299	3.107962	0.061350
H	-1.309160	2.865530	1.462492
H	-1.031906	4.215670	0.353223
H	-0.628685	-1.644533	1.426696
C	3.559563	-1.364839	-0.445049
H	3.976034	-1.272587	0.567459
H	3.824865	-2.355970	-0.834004
H	4.036364	-0.611729	-1.086070

M‘-N‘-TS G = -585.954616, T = -110

C	-0.522269	1.999605	-0.188533
C	-1.154525	0.681876	0.048712
C	-2.963316	-0.976504	-0.054348
C	0.549271	2.285224	0.914457
C	1.804981	-0.805081	1.391652
C	1.911741	0.146878	0.438109
C	1.935509	1.646605	0.658711
H	1.754128	-0.604031	2.464768
H	0.670675	3.375950	0.973224
H	0.047114	1.828468	-1.133993
H	0.159143	1.963483	1.892417
H	2.579668	1.893298	1.515317
C	1.862387	-2.202046	0.813120
C	2.097110	-0.482634	-0.942631

H	1.451670	0.002456	-1.699357
C	1.668727	-1.960209	-0.708044
H	0.603005	-2.081014	-0.968384
H	2.233181	-2.665644	-1.331295
H	2.840389	-2.666239	1.030438
C	-4.375826	-1.268968	-0.429627
H	-4.973012	-1.304636	0.500766
H	-4.817357	-0.512369	-1.086261
H	-4.473538	-2.268764	-0.876618
C	-2.187769	-2.104917	0.539029
H	-2.809437	-2.644739	1.268234
H	-1.979496	-2.833596	-0.264015
H	-1.238044	-1.829097	1.003369
C	-2.452422	0.315201	-0.279638
H	-3.122873	1.044996	-0.737126
H	-0.495646	-0.057198	0.514372
H	2.386560	2.132775	-0.220738
C	-1.489161	3.172028	-0.438081
H	-2.155016	2.987543	-1.290519
H	-2.103543	3.367049	0.451326
H	-0.917281	4.080170	-0.659920
H	1.109656	-2.884151	1.240366
C	3.558416	-0.344919	-1.422037
H	4.244419	-0.838626	-0.719905
H	3.686367	-0.806935	-2.410134
H	3.862151	0.708005	-1.501431

N‘ G = -585.967430

C	0.167244	2.140759	-0.449320
C	-0.676960	1.047642	0.135541
C	-2.575288	-0.513383	-0.226940
C	1.469857	2.348811	0.349892
C	0.824916	-0.469368	1.391328
C	1.643115	-0.157469	0.331413
C	2.394018	1.128890	0.155275
H	0.741682	0.135942	2.295624
H	1.976678	3.264598	0.017638
H	0.420441	1.885945	-1.492419
H	1.227898	2.488759	1.415481
H	3.229151	1.176590	0.873481
C	0.230176	-1.841537	1.266642
C	1.709780	-1.291221	-0.667571
H	1.387579	-0.914651	-1.656974
C	0.713929	-2.361268	-0.118006
H	-0.134827	-2.487745	-0.801744
H	1.202012	-3.339910	-0.028162
H	0.562512	-2.475873	2.104627
C	-3.300876	-1.417747	-1.177918
H	-4.365222	-1.141734	-1.238385
H	-2.872780	-1.413057	-2.186211
H	-3.283744	-2.450244	-0.791513

C	-3.201171	-0.359455	1.131164
H	-4.102592	0.268542	1.036221
H	-3.546572	-1.330772	1.510569
H	-2.550496	0.100758	1.880806
C	-1.449648	0.167625	-0.656946
H	-1.140384	0.028199	-1.696324
H	-0.993631	1.228134	1.165628
H	2.842079	1.155403	-0.848168
C	-0.699932	3.432135	-0.459224
H	-1.631165	3.285168	-1.019397
H	-0.953013	3.746433	0.562620
H	-0.132475	4.241339	-0.935871
H	-0.870311	-1.812178	1.337347
C	3.156473	-1.815964	-0.823461
H	3.540892	-2.187857	0.136093
H	3.173337	-2.644555	-1.542714
H	3.839149	-1.037929	-1.189481

Step N’ – O’

N‘ G = -585.967416

C	0.166550	2.140918	-0.449312
C	-0.677368	1.047562	0.135498
C	-2.575243	-0.513984	-0.226838
C	1.469279	2.349148	0.349725
C	0.825090	-0.469134	1.390958
C	1.643176	-0.157039	0.331031
C	2.393652	1.129535	0.154634
H	0.741716	0.136112	2.295286
H	1.975744	3.265146	0.017511
H	0.419638	1.886346	-1.492490
H	1.227465	2.488858	1.415378
H	3.229201	1.177364	0.872351
C	0.230823	-1.841516	1.266302
C	1.710448	-1.290935	-0.667719
H	1.389208	-0.914646	-1.657529
C	0.714074	-2.360761	-0.118715
H	-0.134880	-2.486182	-0.802399
H	1.201448	-3.339820	-0.029612
H	0.563988	-2.475860	2.103968
C	-3.300645	-1.418626	-1.177687
H	-4.365477	-1.144231	-1.236735
H	-2.873668	-1.412617	-2.186448
H	-3.281449	-2.451381	-0.792024
C	-3.200882	-0.360291	1.131400
H	-4.101376	0.269136	1.036914
H	-3.547688	-1.331361	1.510105
H	-2.549540	0.098497	1.881343
C	-1.449922	0.167429	-0.657008
H	-1.140736	0.028207	-1.696438

H	-0.994054	1.227931	1.165605
H	2.841173	1.156164	-0.849062
C	-0.700905	3.432121	-0.458807
H	-1.632226	3.285066	-1.018809
H	-0.953807	3.746190	0.563152
H	-0.133726	4.241538	-0.935432
H	-0.869632	-1.812747	1.337710
C	3.157316	-1.815759	-0.822065
H	3.540758	-2.187491	0.137946
H	3.174904	-2.644464	-1.541167
H	3.840355	-1.037770	-1.187500

N‘-O‘-TS G = -585.968525, T = -209

C	-0.060714	2.055194	-0.393942
C	-0.652325	0.805845	0.274905
C	-2.637527	-0.680348	-0.178340
C	1.288893	2.464114	0.225685
C	0.713646	-0.225350	1.073767
C	1.741826	0.020073	0.135519
C	2.339257	1.362356	-0.060472
H	0.741053	0.335403	2.011996
H	1.637502	3.415413	-0.196309
H	0.118620	1.818458	-1.458723
H	1.173978	2.623041	1.309396
H	3.193674	1.468236	0.634360
C	0.416783	-1.721432	1.093948
C	2.050901	-1.193239	-0.679618
H	2.036560	-0.951828	-1.756388
C	0.960168	-2.225624	-0.267108
H	0.153812	-2.233743	-1.012534
H	1.363894	-3.243236	-0.206698
H	0.957612	-2.174738	1.938936
C	-3.445663	-1.453005	-1.186804
H	-4.479117	-1.074872	-1.223173
H	-3.020879	-1.405440	-2.196136
H	-3.520213	-2.510104	-0.885560
C	-3.205840	-0.655142	1.217632
H	-4.152035	-0.091189	1.222750
H	-3.458628	-1.674960	1.542952
H	-2.542455	-0.212221	1.967600
C	-1.497544	-0.035465	-0.566497
H	-1.198225	-0.125248	-1.615688
H	-1.116928	1.069068	1.232011
H	2.758154	1.446702	-1.074262
C	-1.096523	3.194645	-0.321279
H	-2.056461	2.882470	-0.751786
H	-1.272895	3.502084	0.719833
H	-0.739247	4.070784	-0.877023
H	-0.648015	-1.943053	1.232351
C	3.502329	-1.654333	-0.332910
H	3.595207	-1.893558	0.734336

H	3.725011	-2.559571	-0.910742
H	4.244012	-0.887632	-0.588412

O‘ G = -585.970157

C	-0.131608	2.039137	-0.370125
C	-0.644018	0.749041	0.312082
C	-2.649166	-0.723796	-0.171720
C	1.232856	2.488954	0.186462
C	0.686659	-0.177735	0.983123
C	1.759468	0.061498	0.066344
C	2.302975	1.418914	-0.143892
H	0.786640	0.360015	1.934182
H	1.535994	3.446813	-0.255531
H	0.012532	1.814266	-1.443589
H	1.165148	2.651555	1.273674
H	3.178011	1.537768	0.525584
C	0.469133	-1.695540	1.045419
C	2.146800	-1.168640	-0.675495
H	2.221277	-0.971619	-1.758185
C	1.048522	-2.204778	-0.297015
H	0.264219	-2.211529	-1.066309
H	1.452462	-3.221581	-0.226207
H	1.031528	-2.099542	1.900345
C	-3.464538	-1.475426	-1.192128
H	-4.491981	-1.081383	-1.229946
H	-3.034509	-1.421783	-2.199136
H	-3.555522	-2.534947	-0.904564
C	-3.216742	-0.719014	1.225774
H	-4.165398	-0.159633	1.242360
H	-3.463193	-1.744727	1.537909
H	-2.553220	-0.284417	1.980851
C	-1.504621	-0.086402	-0.545633
H	-1.199495	-0.171723	-1.594243
H	-1.145319	1.013208	1.251936
H	2.698614	1.517509	-1.166566
C	-1.198536	3.140170	-0.249167
H	-2.165359	2.791461	-0.634475
H	-1.338848	3.440062	0.799847
H	-0.903035	4.030561	-0.818466
H	-0.583232	-1.967882	1.181339
C	3.577352	-1.584826	-0.189358
H	3.588512	-1.772973	0.891581
H	3.853258	-2.512094	-0.705827
H	4.323708	-0.816723	-0.424606

Cartesian coordinates of computed structures (Scheme 4 of main text, Table S12)

Gibbs energies (G in Hartree) and imagininary frequencies of TS (T in cm^{-1}) (wB97M-V/Def2-TZVPPD//B97D3/6-31G(d,p)-sp-density-fitting, 1 bar, 298.15 K)

Step M – P

**M**	G = -585.967219		
C	-0.040736	2.180038	-0.523919
C	-0.684370	0.819572	-0.483221
C	-2.803757	-0.454525	-0.172955
C	1.441695	2.099427	-0.940676
C	0.631412	-0.293867	1.169281
C	1.671627	-0.026969	0.305715
C	2.288415	1.315490	0.106481
H	0.311722	0.389938	1.955905
H	1.526784	1.600096	-1.918321
H	-0.562561	2.643376	-1.390633
H	1.852389	3.111028	-1.058010
H	2.315986	1.878818	1.049214
C	0.219797	-1.738586	1.116260
C	2.062493	-1.267008	-0.470761
H	2.144668	-1.045827	-1.549330
C	0.910279	-2.273620	-0.166837
H	0.195398	-2.289395	-1.001906
H	1.283642	-3.297695	-0.046685
H	0.573809	-2.250805	2.027055
C	-4.084478	-0.603959	0.596521
H	-4.163937	0.095633	1.435707
H	-4.947760	-0.453422	-0.071420
H	-4.180609	-1.632210	0.979662
C	-2.622893	-1.410981	-1.317091
H	-1.703814	-1.266379	-1.891021
H	-2.648022	-2.448335	-0.950559
H	-3.474257	-1.316152	-2.008959
C	-1.922424	0.554933	0.163473
H	-2.207626	1.200538	0.996337
H	-0.411260	0.173717	-1.321251
H	3.317776	1.225466	-0.265250
C	-0.292280	3.085040	0.693836
H	-1.360495	3.282895	0.841492
H	0.102317	2.647608	1.620115
H	0.204059	4.051798	0.546460
H	-0.875505	-1.858742	1.109948
C	3.446910	-1.772171	0.011396
H	4.233050	-1.024816	-0.156264
H	3.425512	-2.013160	1.082914
H	3.718591	-2.680855	-0.540219

M-P-TS G = -585.948664, T = -40

C	-1.767821	2.025508	-0.654870
C	-2.033944	0.577485	-0.724435
C	-2.303791	-1.653058	0.297910
C	-0.195395	2.202590	-0.847502
C	1.180662	-0.679828	-0.802309
C	1.547241	0.430454	-0.120482
C	0.669928	1.592577	0.292836
H	0.170838	-0.896575	-1.153111
H	0.100585	1.773060	-1.813028
H	-2.194248	2.475316	-1.569652
H	-0.046808	3.289727	-0.917718
H	0.022819	1.278652	1.129714
C	2.329628	-1.633685	-1.021181
C	3.016375	0.367300	0.276182
H	3.521727	1.314252	0.015538
C	3.572286	-0.813751	-0.574604
H	4.089630	-0.421583	-1.461395
H	4.296193	-1.420419	-0.015092
H	2.193772	-2.547103	-0.412063
C	-2.369049	-2.431601	1.568630
H	-2.642217	-1.822200	2.436317
H	-3.049359	-3.290687	1.477687
H	-1.369796	-2.865149	1.756632
C	-2.262286	-2.448578	-0.963711
H	-2.102865	-1.872120	-1.878090
H	-1.491381	-3.230575	-0.889080
H	-3.219507	-2.988552	-1.060666
C	-2.278296	-0.247644	0.364088
H	-2.382945	0.201537	1.353250
H	-1.925074	0.123529	-1.712931
H	1.310122	2.380631	0.713458
C	-2.289481	2.768930	0.581534
H	-3.368766	2.618002	0.710143
H	-1.784939	2.451884	1.503330
H	-2.107829	3.844146	0.465345
H	2.403917	-1.977822	-2.064815
C	3.172337	0.156964	1.799223
H	2.714462	0.975141	2.373516
H	2.696919	-0.784663	2.110934
H	4.234387	0.109594	2.073924

P G = -585.963761

C	0.823728	2.101727	-0.001189
C	-0.380813	1.284480	-0.373492
C	-2.612376	0.387495	0.230266
C	2.043519	1.772079	-0.905582
C	0.490602	-0.723633	-1.528220
C	1.515002	-0.674757	-0.611934
C	2.594567	0.361641	-0.604602
H	0.469305	-0.128487	-2.441875

H	1.746622	1.845736	-1.962617
H	0.514813	3.124677	-0.304629
H	2.828793	2.522143	-0.742724
H	3.130535	0.348471	0.353400
C	-0.432436	-1.885017	-1.289056
C	1.440724	-1.861833	0.333812
H	2.202591	-2.568595	-0.057650
C	0.023006	-2.451746	0.079578
H	0.019361	-3.547547	0.106335
H	-0.655594	-2.106343	0.873864
H	-1.492396	-1.582449	-1.290285
C	-3.499155	-0.176333	1.299757
H	-3.020652	-0.203406	2.284831
H	-4.434013	0.401492	1.371045
H	-3.803771	-1.200290	1.026773
C	-3.256985	0.584456	-1.113274
H	-2.552772	0.747085	-1.934780
H	-3.899237	-0.270855	-1.363334
H	-3.923549	1.461520	-1.062304
C	-1.312032	0.746934	0.544094
H	-0.991478	0.609270	1.578039
H	-0.721469	1.449756	-1.397173
H	3.344567	0.109849	-1.374668
C	1.172803	2.140884	1.492360
H	0.338891	2.533570	2.087213
H	1.431074	1.149938	1.883940
H	2.037058	2.796704	1.654599
H	-0.325938	-2.616165	-2.108762
C	1.762951	-1.628434	1.816556
H	2.760866	-1.195782	1.964514
H	1.021659	-0.965341	2.283786
H	1.736098	-2.582610	2.358024

Step P – Q

**P**	G = -585.963393		
C	0.580168	2.151888	-0.356684
C	-0.412733	1.036504	-0.612311
C	-2.448547	-0.075037	0.302784
C	1.752157	1.760754	0.555727
C	0.883110	-0.548184	-1.552039
C	1.736227	-0.498984	-0.460872
C	2.615830	0.655436	-0.124121
H	1.015593	0.089305	-2.428418
H	2.383335	2.638076	0.751167
H	0.997131	2.452611	-1.331796
H	1.386702	1.409805	1.532106
H	3.425081	0.353298	0.553390
C	0.123695	-1.855537	-1.597609
C	1.616937	-1.751579	0.376106

H	2.528081	-2.326779	0.101953
C	0.366539	-2.479771	-0.197381
H	0.508096	-3.565805	-0.240184
H	-0.493947	-2.290308	0.458909
H	-0.942430	-1.722635	-1.828328
C	-3.164350	-0.601061	1.514002
H	-3.375365	-1.675626	1.386256
H	-2.595355	-0.463607	2.440271
H	-4.145929	-0.114888	1.624735
C	-3.219952	-0.159559	-0.985569
H	-3.590134	-1.184207	-1.137232
H	-4.116636	0.476415	-0.913745
H	-2.661567	0.141782	-1.876859
C	-1.187981	0.460358	0.440157
H	-0.765546	0.481657	1.447375
H	-0.905723	1.091153	-1.585639
H	3.066153	1.076422	-1.034780
C	-0.204690	3.367440	0.214376
H	-1.050158	3.638001	-0.430008
H	-0.592651	3.148071	1.217171
H	0.466039	4.232960	0.280907
H	0.539258	-2.481204	-2.405201
C	1.633357	-1.585825	1.904317
H	2.535646	-1.067841	2.253963
H	0.755790	-1.026229	2.253406
H	1.609931	-2.571492	2.385279

P-Q-TS G = -585.967384, T = -354

C	0.368945	2.167062	-0.325241
C	-0.457706	0.917888	-0.638166
C	-2.442273	-0.281411	0.328999
C	1.573879	1.893402	0.583451
C	0.821791	-0.335097	-1.493437
C	1.787028	-0.314280	-0.469024
C	2.565095	0.892925	-0.106889
H	0.962103	0.292383	-2.377592
H	2.117955	2.824362	0.790955
H	0.754439	2.553221	-1.284232
H	1.251016	1.490487	1.554714
H	3.393766	0.659587	0.572384
C	0.224756	-1.731785	-1.609819
C	1.817398	-1.615746	0.276735
H	2.750759	-2.084267	-0.113050
C	0.593523	-2.409530	-0.265803
H	0.816307	-3.476717	-0.377339
H	-0.237046	-2.321007	0.447383
H	-0.856625	-1.717645	-1.795709
C	-3.104335	-0.864538	1.548746
H	-3.319283	-1.933503	1.389148
H	-2.494559	-0.761547	2.453908
H	-4.078679	-0.383637	1.725895

C	-3.264712	-0.340792	-0.932081
H	-3.608817	-1.370544	-1.109889
H	-4.175562	0.265406	-0.806505
H	-2.748893	0.007134	-1.832763
C	-1.194261	0.268094	0.431150
H	-0.724124	0.248129	1.418398
H	-1.038186	1.029621	-1.559640
H	2.967558	1.379488	-1.007382
C	-0.563808	3.247658	0.278375
H	-1.429448	3.431040	-0.370255
H	-0.935694	2.940631	1.264254
H	-0.015727	4.191179	0.391714
H	0.691601	-2.245788	-2.464895
C	1.946715	-1.554418	1.808395
H	2.837959	-1.000340	2.128447
H	1.063136	-1.083063	2.257970
H	2.023832	-2.571206	2.211728

Q G = -585.975369

C	-0.015801	2.160640	-0.298746
C	-0.533000	0.754345	-0.698549
C	-2.401887	-0.611953	0.356769
C	1.190773	2.086549	0.643814
C	0.741419	-0.083969	-1.394520
C	1.821741	-0.004459	-0.431367
C	2.384932	1.286751	-0.020382
H	0.924487	0.522972	-2.292934
H	1.573743	3.088645	0.877675
H	0.318722	2.659214	-1.226065
H	0.919715	1.614540	1.599037
H	3.224715	1.192443	0.676595
C	0.458556	-1.582420	-1.626579
C	2.132419	-1.331115	0.155561
H	3.071798	-1.579387	-0.401849
C	1.004694	-2.272354	-0.355699
H	1.376299	-3.286057	-0.542885
H	0.222385	-2.342060	0.412471
H	-0.607167	-1.781822	-1.787925
C	-2.950282	-1.294141	1.585480
H	-3.134534	-2.361240	1.383565
H	-2.279348	-1.212983	2.449438
H	-3.924910	-0.863552	1.862599
C	-3.307143	-0.654655	-0.850241
H	-3.634414	-1.687576	-1.040996
H	-4.222706	-0.075793	-0.651394
H	-2.858019	-0.266970	-1.771038
C	-1.185541	-0.009215	0.396113
H	-0.635056	-0.075954	1.341649
H	-1.218214	0.855941	-1.550832
H	2.701438	1.863139	-0.903437
C	-1.155126	3.001770	0.311939

H	-2.015887	3.041603	-0.367619
H	-1.496623	2.573440	1.263071
H	-0.817898	4.029578	0.495544
H	1.003386	-1.916941	-2.521625
C	2.462060	-1.397038	1.657425
H	3.302031	-0.747169	1.930653
H	1.587093	-1.111201	2.255825
H	2.732073	-2.425229	1.924360

Step Q – R

**Q**	G = -585.975812		
C	-0.554501	2.051445	-0.434097
C	-0.644051	0.528324	-0.751126
C	-2.355659	-1.065924	0.296428
C	0.427629	2.339350	0.713998
C	0.816850	-0.008277	-1.080304
C	1.834806	0.423078	-0.119035
C	1.857176	1.787101	0.427829
H	1.093571	0.549356	-2.003522
H	0.508164	3.420542	0.884351
H	-0.159995	2.545248	-1.341664
H	0.059297	1.898668	1.652570
H	2.539002	1.872801	1.284597
C	1.048442	-1.526183	-1.293114
C	2.747105	-0.680510	0.241153
H	3.785948	-0.335406	0.364000
C	2.521184	-1.738449	-0.871685
H	3.198463	-1.531023	-1.713285
H	2.726698	-2.756622	-0.520533
H	0.361799	-2.087399	-0.644362
C	-2.869975	-1.749383	1.540208
H	-2.892029	-2.841671	1.400715
H	-2.266111	-1.522424	2.427532
H	-3.908785	-1.446181	1.743191
C	-3.167274	-1.316708	-0.950873
H	-3.327728	-2.396486	-1.087612
H	-4.167735	-0.868725	-0.843447
H	-2.718190	-0.921791	-1.868417
C	-1.250534	-0.282066	0.352913
H	-0.741710	-0.216871	1.323040
H	-1.229882	0.408969	-1.672563
H	2.300828	2.406661	-0.380442
C	-1.944930	2.636629	-0.137718
H	-2.638053	2.430062	-0.963735
H	-2.370778	2.200012	0.775220
H	-1.888627	3.724764	-0.005389
H	0.848773	-1.833409	-2.325725
C	2.265178	-1.185925	1.655756
H	2.367314	-0.407797	2.420535

H	1.225537	-1.531563	1.628856
H	2.905561	-2.034723	1.924090

Q-R-TS G = -585.973480, T = -625

C	-0.579119	2.003164	-0.535193
C	-0.702469	0.454854	-0.685028
C	-2.510753	-1.051191	0.359081
C	0.360859	2.381358	0.628575
C	0.659205	-0.195050	-0.684870
C	1.800412	0.416212	-0.093494
C	1.786258	1.811188	0.463188
H	1.480016	0.494079	-1.414460
H	0.420899	3.474137	0.714575
H	-0.121305	2.375250	-1.472152
H	-0.068581	2.021863	1.576139
H	2.325593	1.784216	1.422331
C	0.970028	-1.641351	-1.024035
C	2.937742	-0.582459	0.016923
H	3.905648	-0.128186	-0.239021
C	2.517159	-1.700663	-0.980906
H	2.924046	-1.478558	-1.979127
H	2.895160	-2.684194	-0.680592
H	0.509800	-2.251724	-0.232149
C	-3.163383	-1.593726	1.609059
H	-3.146042	-2.694842	1.607511
H	-2.678351	-1.238959	2.526520
H	-4.224370	-1.302476	1.644433
C	-3.148518	-1.497726	-0.934013
H	-3.150199	-2.597020	-0.994128
H	-4.204574	-1.188560	-0.959383
H	-2.662504	-1.113023	-1.837525
C	-1.449730	-0.217830	0.454987
H	-1.072550	0.010407	1.457175
H	-1.203752	0.226915	-1.636718
H	2.407945	2.448757	-0.188408
C	-1.960588	2.652843	-0.387200
H	-2.621942	2.367097	-1.216071
H	-2.443814	2.342299	0.548665
H	-1.874656	3.746896	-0.382512
H	0.529100	-1.961769	-1.975793
C	2.989509	-1.076348	1.485929
H	3.251078	-0.269054	2.180707
H	2.028633	-1.506287	1.801326
H	3.755036	-1.856911	1.570646

R G = -585.977649

C	-1.194087	1.970612	-0.536289
C	-0.919098	0.469606	-0.815412
C	-1.598526	-1.758247	0.332718
C	-0.497779	2.490739	0.753455
C	0.505214	0.130870	-0.777700

C	1.451886	0.973513	-0.014699
C	0.855712	1.808948	1.122877
H	1.718508	1.682239	-0.845640
H	-0.352020	3.572876	0.635024
H	-0.758793	2.505647	-1.398188
H	-1.190850	2.377747	1.599938
H	0.717201	1.142800	1.985635
C	1.248846	-0.952072	-1.493424
C	2.713536	0.081850	0.194695
H	3.607825	0.711452	0.299745
C	2.744181	-0.706069	-1.142909
H	3.209290	-0.095300	-1.930171
H	3.307410	-1.643698	-1.071027
H	0.910484	-1.931642	-1.122756
C	-1.975527	-2.478934	1.599927
H	-1.361243	-3.386051	1.716046
H	-1.858210	-1.857466	2.494708
H	-3.019782	-2.822839	1.545494
C	-1.695137	-2.563410	-0.931950
H	-0.931654	-3.360126	-0.929971
H	-2.663661	-3.083008	-0.972642
H	-1.573035	-1.980103	-1.849904
C	-1.227321	-0.444533	0.389063
H	-1.161743	0.026403	1.369040
H	-1.432828	0.112102	-1.718031
H	1.593683	2.558554	1.435194
C	-2.702486	2.250254	-0.507210
H	-3.181019	1.976016	-1.457003
H	-3.191008	1.683794	0.298553
H	-2.892784	3.316516	-0.329441
H	1.009726	-0.952949	-2.569418
C	2.590686	-0.830675	1.424928
H	2.579961	-0.252723	2.357681
H	1.670794	-1.434991	1.396259
H	3.443141	-1.519965	1.472456

Cartesian coordinates of computed structures (alternative mechanisms)

Gibbs energies (G in Hartree) and imagininary frequencies of TS (T in cm^{-1}) (wB97M-V/Def2-TZVPPD//B97D3/6-31G(d,p)-sp-density-fitting, 1 bar, 298.15 K)

Scheme S6

Step G1 – I1

G1 G = -585.968109

C	0.313109	1.954642	-0.479896
C	0.461653	0.470226	-0.616051
C	-2.658720	-0.258905	0.217380
C	1.794623	2.440756	-0.356735
C	-0.480390	-0.526502	-1.154986
C	1.826321	0.053994	-0.249430
C	2.505938	1.287486	0.380689
H	-1.074343	-0.107261	-1.979338
H	2.227888	2.570133	-1.360269
H	-0.230489	2.382133	-1.336762
H	1.857697	3.402340	0.164983
H	2.311877	1.324388	1.461941
C	0.242184	-1.830640	-1.565393
C	2.064640	-1.386768	0.263094
H	3.104059	-1.631838	-0.002415
C	1.113235	-2.415955	-0.434365
H	1.703860	-3.247378	-0.840617
H	0.454847	-2.864649	0.323480
H	-0.512298	-2.566897	-1.872604
C	-3.465798	-0.669401	1.423547
H	-3.797176	0.222540	1.978512
H	-4.381166	-1.194425	1.110932
H	-2.907427	-1.319030	2.107253
C	-3.383153	0.603566	-0.782669
H	-4.228486	0.043491	-1.212170
H	-3.825069	1.478231	-0.282094
H	-2.756471	0.966540	-1.603986
C	-1.384889	-0.714526	0.076317
H	-0.964413	-1.321443	0.877581
H	2.182893	0.047227	-1.321861
H	3.592552	1.281828	0.238007
C	-0.474865	2.330479	0.818775
H	-0.409551	3.417410	0.948023
H	-1.522942	2.034492	0.740074
H	-0.045863	1.847066	1.704369
H	0.857987	-1.616032	-2.453878
C	1.955463	-1.455167	1.798741
H	2.092073	-2.491085	2.134105
H	2.722722	-0.844371	2.290454
H	0.971133	-1.113120	2.152192

G1-I1-TS G = -585.941760, T = -174

C	-0.654767	1.920234	-0.561498
C	-0.075606	0.476373	-0.517295
C	-1.795539	-1.101774	0.673330
C	0.074806	2.612337	0.623588
C	-0.154661	-0.394104	-1.669558
C	1.478736	0.769570	-0.242275
C	1.401312	1.833288	0.872306
H	-0.847593	-0.192138	-2.496556
H	0.246618	3.672870	0.401715
H	-0.280295	2.359611	-1.503611
H	-0.560630	2.575601	1.519836
H	1.394097	1.365035	1.864654
C	0.793096	-1.500607	-1.839956
C	2.395093	-0.479883	-0.086847
H	3.274915	-0.283211	-0.721166
C	1.715335	-1.762956	-0.652386
H	2.479653	-2.497826	-0.936080
H	1.111796	-2.230264	0.142193
H	0.272099	-2.406969	-2.199388
C	-2.184009	-1.773727	1.967543
H	-3.141317	-1.369625	2.330701
H	-2.348911	-2.850055	1.801924
H	-1.432001	-1.652546	2.755307
C	-2.786711	-1.270259	-0.446051
H	-2.781239	-2.314825	-0.794964
H	-3.806543	-1.063434	-0.093345
H	-2.590609	-0.628541	-1.312708
C	-0.619315	-0.415535	0.618118
H	0.027369	-0.483800	1.494662
H	1.792775	1.293149	-1.159158
H	2.288339	2.479800	0.835083
C	-2.174127	2.084332	-0.524746
H	-2.428647	3.151771	-0.492381
H	-2.664100	1.662799	-1.413127
H	-2.604730	1.613692	0.368951
H	1.363455	-1.187704	-2.749073
C	2.924450	-0.750896	1.331416
H	3.603592	-1.614047	1.317619
H	3.484886	0.106296	1.723507
H	2.118469	-0.983994	2.041272

I1 G = -585.982879

C	-0.374775	2.014462	-0.423623
C	-0.744063	0.575497	-0.250108
C	-2.807367	-0.892317	0.080381
C	0.855474	2.386701	0.486628
C	0.347894	-0.396490	-0.569166
C	1.782520	0.236919	-0.517040
C	1.856473	1.231383	0.657180
H	0.163656	-0.662324	-1.633881

H	1.329080	3.262552	0.023057
H	0.034190	2.033130	-1.455091
H	0.462250	2.711291	1.460359
H	1.646939	0.697053	1.597378
C	0.430491	-1.729442	0.257663
C	2.692429	-1.007599	-0.365841
H	2.689781	-1.519832	-1.345484
C	1.921799	-1.896437	0.631637
H	2.241888	-2.945295	0.589068
H	2.104540	-1.547818	1.659030
H	-0.222285	-1.688690	1.139885
C	-4.124797	-0.959582	0.789323
H	-4.942453	-0.817087	0.061984
H	-4.276707	-1.965137	1.209709
H	-4.228605	-0.208629	1.579570
C	-2.515784	-2.026553	-0.848849
H	-2.228697	-2.914155	-0.262325
H	-3.434466	-2.307277	-1.383663
H	-1.726781	-1.819688	-1.575392
C	-2.030881	0.253119	0.232863
H	-2.542185	1.072691	0.747089
H	2.015176	0.771731	-1.451923
H	2.873722	1.632697	0.747039
C	-1.490378	3.059522	-0.325540
H	-1.092416	4.041032	-0.611427
H	-2.332105	2.829873	-0.991380
H	-1.870965	3.156298	0.701258
H	0.098124	-2.576254	-0.353079
C	4.145654	-0.725098	0.025665
H	4.726659	-1.656724	0.031615
H	4.626856	-0.035710	-0.681701
H	4.216669	-0.288238	1.031263

Step I1 – J1

I1 G = -585.984551

C	-0.291358	1.927503	-0.133655
C	-0.808345	0.510170	-0.140339
C	-3.016816	-0.756794	-0.225571
C	1.240982	2.022318	-0.184273
C	0.166979	-0.586424	0.026483
C	1.433580	-0.440911	-0.914552
C	1.811443	1.030144	-1.207924
H	-0.284660	-1.556038	-0.200193
H	1.513257	3.051427	-0.456367
H	-0.701819	2.387426	-1.054946
H	1.666399	1.852922	0.815724
H	2.900900	1.140413	-1.272749
C	0.756698	-0.645748	1.503673
C	2.479422	-1.284831	-0.126082
H	2.148470	-2.335843	-0.216709

C	2.280359	-0.858216	1.337088
H	2.660327	-1.605541	2.046733
H	2.825856	0.076545	1.534678
H	0.525346	0.256527	2.083200
C	-4.448183	-0.642239	-0.645183
H	-4.736322	-1.525946	-1.236483
H	-5.093069	-0.668869	0.250158
H	-4.668274	0.268198	-1.211739
C	-2.633250	-2.070001	0.390574
H	-1.852903	-1.984321	1.151299
H	-3.512775	-2.555272	0.831943
H	-2.270184	-2.750511	-0.398916
C	-2.193153	0.354965	-0.387018
H	-2.705682	1.259574	-0.728421
H	1.204222	-0.936980	-1.869034
H	1.419302	1.304841	-2.200179
C	-0.890913	2.734853	1.056546
H	-0.539930	3.771519	0.986433
H	-1.987093	2.738911	1.048329
H	-0.553162	2.321581	2.014975
H	0.275104	-1.481220	2.029318
C	3.923525	-1.204347	-0.630621
H	4.551628	-1.926927	-0.093050
H	3.989592	-1.435527	-1.702299
H	4.359646	-0.209545	-0.466584

I1-J1-TS G = -585.891495, T = -1256

C	-0.464702	2.021201	-0.240137
C	-0.684785	0.516176	-0.357488
C	-2.822345	-0.872464	-0.297181
C	1.025399	2.326585	0.037268
C	0.278291	-0.432315	0.386107
C	1.402580	-0.110305	-0.550692
C	1.962729	1.291109	-0.602954
H	-0.068205	-1.438198	0.099806
H	1.275212	3.338909	-0.304160
H	-0.780598	2.481079	-1.191433
H	1.183676	2.318904	1.125246
H	2.924669	1.275625	-0.062327
C	1.026973	-0.614694	1.713739
C	2.319328	-1.299073	-0.482076
H	1.790986	-2.204425	-0.815892
C	2.331679	-1.331095	1.144024
H	2.393133	-2.378685	1.461514
H	3.237020	-0.813872	1.484912
H	1.283652	0.330977	2.204723
C	-4.156112	-1.123910	-0.949059
H	-4.276755	-2.200399	-1.150427
H	-4.974815	-0.851556	-0.264313
H	-4.285663	-0.573542	-1.887479
C	-2.564572	-1.678291	0.950627

H	-1.759570	-1.287250	1.577767
H	-3.480140	-1.711918	1.558103
H	-2.336497	-2.724285	0.689747
C	-1.999316	0.088818	-0.821755
H	-2.386048	0.666234	-1.666522
H	0.127897	0.220006	-1.435930
H	2.237144	1.531498	-1.642461
C	-1.362904	2.582591	0.886427
H	-1.161989	3.653920	1.016133
H	-2.426119	2.451692	0.652874
H	-1.153280	2.079171	1.840808
H	0.500596	-1.242262	2.442888
C	3.701040	-1.235890	-1.126562
H	4.269738	-2.141660	-0.880407
H	3.623979	-1.177090	-2.220017
H	4.282286	-0.373537	-0.774877

J1 G = -585.951022

C	-0.193630	1.847652	0.238501
C	-0.623345	0.744088	-0.802874
C	-2.934397	-0.324485	-0.076229
C	1.317441	2.196216	0.112247
C	0.400138	-0.444514	-0.845418
C	1.794779	0.005746	-0.775083
C	2.111044	1.404571	-1.024895
H	0.348594	-0.800458	-1.905180
H	1.464698	3.256277	-0.133467
H	-0.769776	2.735069	-0.066465
H	1.834349	2.010725	1.062177
H	3.180516	1.637753	-0.991755
C	0.375013	-1.747495	0.007621
C	2.694690	-1.048818	-0.247141
H	3.587316	-1.134421	-0.891744
C	1.792561	-2.314250	-0.214727
H	1.849574	-2.834712	-1.182453
H	2.098805	-3.021284	0.564958
H	0.221106	-1.504338	1.066156
C	-4.390371	-0.443799	-0.475432
H	-4.675610	-1.502347	-0.582251
H	-5.039799	-0.029010	0.311308
H	-4.616675	0.070626	-1.416646
C	-2.605820	-1.071955	1.192625
H	-1.604031	-0.889006	1.580741
H	-3.324185	-0.800311	1.980534
H	-2.725321	-2.155721	1.034989
C	-2.100851	0.400873	-0.863657
H	-2.587607	0.841987	-1.738554
H	-0.449131	1.244917	-1.770168
H	1.688869	1.728141	-1.989426
C	-0.551004	1.552910	1.699699
H	-0.265606	2.402673	2.334271

H	-1.627215	1.392246	1.817126
H	-0.020906	0.669026	2.085296
H	-0.427243	-2.424453	-0.299146
C	3.215556	-0.631608	1.171717
H	3.858940	-1.438970	1.539951
H	3.802520	0.293132	1.131209
H	2.386991	-0.498339	1.876805

Scheme S7

For **I1 – J1** cf. cartesian coordinates for Scheme S6.

Scheme S8

Step I2 – J2

I2 G = -585.981942

C	-0.713310	1.539035	0.167285
C	-0.615464	0.037727	0.270418
C	-3.016728	-0.694284	-0.206114
C	0.641767	2.195892	-0.196819
C	0.757256	-0.449997	0.462773
C	1.546886	-0.082282	-0.876779
C	1.382563	1.397034	-1.285323
H	1.231109	0.182007	1.237397
H	0.446474	3.225757	-0.526784
H	-1.444500	1.795473	-0.613711
H	1.271691	2.279058	0.701779
H	2.355906	1.862305	-1.488538
C	1.142340	-1.914923	0.678027
C	2.951904	-0.654799	-0.535303
H	3.459477	-0.888908	-1.484809
C	2.644440	-1.970063	0.258315
H	2.838194	-2.862466	-0.349725
H	3.295364	-2.040694	1.139917
H	0.972895	-2.248272	1.710329
C	-3.916311	-1.887923	-0.212988
H	-4.348344	-2.018129	-1.219578
H	-3.424983	-2.816576	0.093908
H	-4.779829	-1.696304	0.446311
C	-3.655733	0.595419	-0.635026
H	-4.707523	0.446177	-0.899684
H	-3.604117	1.355202	0.155885
H	-3.139185	1.015235	-1.511632
C	-1.684509	-0.880736	0.164834
H	-1.421352	-1.912995	0.402360
H	1.110127	-0.727570	-1.656545
H	0.806964	1.455520	-2.221591
C	-1.244988	2.098576	1.522290
H	-2.209760	1.663079	1.807142
H	-0.529455	1.887807	2.327405

H	-1.358109	3.186164	1.438915
H	0.550611	-2.566639	0.017934
C	3.842342	0.304181	0.266179
H	3.389663	0.579964	1.231331
H	4.800485	-0.181357	0.493623
H	4.063470	1.229026	-0.282255

I2-J2-TS G = -585.961725, T = -460

C	-0.646868	1.905223	-0.034170
C	-0.393458	0.431427	0.353280
C	-2.452949	-0.895349	-0.307266
C	0.703118	2.540279	-0.465051
C	0.926840	0.224265	1.110775
C	1.362765	0.162297	-0.335080
C	1.644184	1.458746	-1.058199
H	1.243856	1.092141	1.693543
H	0.535308	3.350174	-1.185304
H	-1.346840	1.901117	-0.883144
H	1.185129	2.996725	0.410647
H	2.696789	1.728528	-0.884378
C	1.206511	-1.162038	1.710551
C	1.913392	-1.224702	-0.648782
H	1.602493	-1.570920	-1.645405
C	1.332578	-2.106582	0.490298
H	0.341073	-2.481559	0.199821
H	1.967891	-2.976584	0.694916
H	2.153599	-1.108930	2.265337
C	-3.602361	-1.769267	0.124188
H	-3.579303	-2.728000	-0.416942
H	-3.594844	-1.975406	1.200528
H	-4.560391	-1.292908	-0.134486
C	-2.423720	-0.543332	-1.773466
H	-2.521961	-1.455430	-2.380166
H	-3.288083	0.089007	-2.028877
H	-1.518703	-0.016742	-2.103715
C	-1.522155	-0.499613	0.597209
H	-1.596916	-0.867213	1.622863
H	0.064354	0.006708	-0.755511
H	1.520305	1.325834	-2.142624
C	-1.313155	2.661014	1.128150
H	-2.283799	2.217720	1.381900
H	-0.680825	2.651461	2.026829
H	-1.477103	3.707955	0.843697
H	0.436650	-1.486209	2.421777
C	3.458514	-1.135863	-0.619410
H	3.825548	-0.746015	0.339567
H	3.872387	-2.142858	-0.755280
H	3.843964	-0.497625	-1.423749

J2 G = -585.971196

C	-0.295168	2.121829	-0.379696

C	-0.512813	0.588217	-0.201077
C	-2.685752	-0.735320	-0.206662
C	1.209588	2.510538	-0.288773
C	0.677241	0.026698	0.892840
C	1.732560	0.083784	-0.080547
C	2.154469	1.363142	-0.689675
H	0.652628	0.792692	1.677057
H	1.412160	3.392506	-0.909344
H	-0.642321	2.336053	-1.403617
H	1.437742	2.806167	0.746647
H	3.183574	1.579337	-0.342813
C	0.538315	-1.440828	1.320389
C	2.181635	-1.278331	-0.491108
H	2.286316	-1.362177	-1.584381
C	1.103916	-2.224852	0.114564
H	0.312940	-2.404346	-0.628003
H	1.522516	-3.198838	0.393851
H	1.148912	-1.603680	2.220866
C	-3.991076	-1.040236	0.484653
H	-4.087791	-2.123796	0.657955
H	-4.091315	-0.522481	1.445592
H	-4.840319	-0.755790	-0.155583
C	-2.483573	-1.437154	-1.525105
H	-2.455418	-2.527072	-1.368307
H	-3.341328	-1.247884	-2.187816
H	-1.573816	-1.146260	-2.061647
C	-1.807609	0.135288	0.361153
H	-2.063809	0.544090	1.342197
H	-0.331617	0.097736	-1.168114
H	2.257534	1.230085	-1.780821
C	-1.115253	2.987246	0.588852
H	-2.192769	2.835258	0.453474
H	-0.867672	2.769601	1.638882
H	-0.898569	4.049396	0.416006
H	-0.495283	-1.710147	1.564981
C	3.599509	-1.508168	0.132743
H	3.577838	-1.411530	1.225514
H	3.918112	-2.527284	-0.117587
H	4.335831	-0.802753	-0.270301

Scheme S9

Step N2 – I2

N2 G = -585.974572

C	-0.444475	1.838425	-0.322819
C	-0.038037	0.397108	-0.011471
C	-2.451166	-0.933633	-0.357392
C	0.957250	2.513232	-0.280087
C	-0.381137	-0.366446	1.179343
C	1.248354	0.091071	-0.793015

C	1.992792	1.463163	-0.756769
H	-1.101930	0.103825	1.854249
H	0.959256	3.428484	-0.885084
H	-0.803735	1.864643	-1.366061
H	1.169212	2.812853	0.757296
H	2.843913	1.422648	-0.066353
C	0.652462	-1.282829	1.856290
C	2.049442	-1.126465	-0.265106
H	1.519936	-2.043979	-0.584378
C	2.065934	-1.118157	1.273124
H	2.709784	-1.924379	1.650396
H	2.507766	-0.172475	1.629817
H	0.647835	-1.042756	2.929286
C	-2.978358	-1.821729	-1.444079
H	-3.591385	-2.621636	-0.995636
H	-3.660598	-1.257862	-2.098432
H	-2.189899	-2.283654	-2.046825
C	-3.478611	-0.151396	0.392184
H	-3.959263	0.577764	-0.278653
H	-4.282315	-0.838531	0.700280
H	-3.098799	0.369552	1.272110
C	-1.088103	-0.968680	-0.083594
H	-0.527496	-1.743322	-0.607040
H	0.960760	-0.104033	-1.840957
H	2.398699	1.698562	-1.747722
C	-1.437074	2.565287	0.588309
H	-1.382837	3.641943	0.381660
H	-2.477180	2.266742	0.431813
H	-1.184167	2.429595	1.649218
H	0.322688	-2.329321	1.773954
C	3.457173	-1.197199	-0.877390
H	3.948331	-2.136524	-0.592651
H	3.417532	-1.155206	-1.974491
H	4.095204	-0.373456	-0.530962

N2-I2-TS G = -585.945650, T = -294

C	-0.334870	1.881795	-0.413592
C	-0.134535	0.363506	-0.122603
C	-2.443133	-0.865736	-0.383161
C	1.040864	2.455511	-0.021426
C	-0.128470	-0.030962	1.276304
C	1.310425	0.097836	-0.770995
C	2.086587	1.414260	-0.492675
H	-0.536877	0.661915	2.023483
H	1.205482	3.456478	-0.438718
H	-0.446046	1.948092	-1.509709
H	1.085708	2.561644	1.077901
H	2.869260	1.271395	0.264062
C	0.503030	-1.268652	1.771020
C	1.978730	-1.209238	-0.305841
H	1.376647	-2.050183	-0.695578

C	1.978471	-1.317955	1.223317
H	2.419516	-2.264865	1.562299
H	2.565938	-0.507392	1.679426
H	0.462485	-1.326969	2.864523
C	-3.282109	-1.749582	-1.276489
H	-3.663113	-2.610888	-0.705197
H	-4.166815	-1.197751	-1.628646
H	-2.733922	-2.122141	-2.148716
C	-3.155160	-0.353304	0.838763
H	-4.120914	0.097109	0.569845
H	-3.381229	-1.196705	1.510128
H	-2.580854	0.381106	1.414373
C	-1.152206	-0.625186	-0.737013
H	-0.735095	-1.209622	-1.563312
H	1.105974	0.025235	-1.852320
H	2.592815	1.741296	-1.410093
C	-1.522631	2.601521	0.224938
H	-1.494880	3.664006	-0.049611
H	-2.481516	2.197676	-0.120254
H	-1.498733	2.562972	1.324446
H	-0.012096	-2.152116	1.365150
C	3.401606	-1.352293	-0.883768
H	3.823470	-2.331712	-0.625264
H	3.388304	-1.266538	-1.978046
H	4.076898	-0.581158	-0.490848

I2 G = -585.976540

C	-0.344980	2.101704	-0.128910
C	-0.677739	0.631189	-0.013227
C	-2.774751	-0.782386	-0.258668
C	1.119123	2.421644	0.214678
C	0.384708	-0.222584	0.534266
C	1.518652	0.043919	-0.605797
C	2.127400	1.445608	-0.443168
H	0.807150	0.215609	1.455220
H	1.332991	3.459211	-0.074220
H	-0.562434	2.395893	-1.172611
H	1.238201	2.386900	1.309090
H	3.031190	1.411009	0.177595
C	0.328634	-1.748447	0.636409
C	2.437483	-1.239560	-0.520001
H	2.330344	-1.755177	-1.487055
C	1.815278	-2.134569	0.577553
H	1.968572	-3.203560	0.383192
H	2.288906	-1.910620	1.547574
H	-0.154131	-2.104769	1.553139
C	-3.951966	-1.074281	-1.141060
H	-4.070631	-2.163478	-1.251303
H	-4.880658	-0.722148	-0.663034
H	-3.870586	-0.617062	-2.132614
C	-2.766631	-1.483318	1.065727

H	-3.765744	-1.393654	1.518632
H	-2.599062	-2.562616	0.926262
H	-2.031622	-1.089147	1.769631
C	-1.876221	0.197446	-0.645062
H	-2.194518	0.812199	-1.494993
H	1.002509	0.005108	-1.578392
H	2.434816	1.819499	-1.430002
C	-1.326973	2.906050	0.776103
H	-1.094001	3.974207	0.689329
H	-2.370801	2.748620	0.480475
H	-1.212267	2.614020	1.828423
H	-0.215621	-2.168898	-0.224307
C	3.928221	-0.965042	-0.289906
H	4.475626	-1.917720	-0.292329
H	4.359964	-0.330157	-1.074337
H	4.110371	-0.487509	0.683021

For **I2** – **J2** cf. cartesian coordinates for Scheme S8.

Scheme S10

Step G3 – I3

G3 G = -585.970627

C	-0.171177	1.987504	-0.571924
C	0.294706	0.574874	-0.635848
C	-2.446235	-0.819505	0.501740
C	0.625677	2.570629	0.630245
C	-0.398219	-0.638065	-1.091681
C	1.696871	0.484212	-0.151910
C	1.963278	1.783040	0.664861
H	-1.146493	-0.433234	-1.868473
H	0.763159	3.653807	0.534072
H	0.296116	2.396164	-1.502175
H	0.044516	2.394261	1.548582
H	2.262066	1.537205	1.691850
C	0.579679	-1.763525	-1.475976
C	2.113374	-0.877118	0.472384
H	1.635330	-0.906093	1.466790
C	1.610342	-2.095489	-0.367570
H	2.469429	-2.587409	-0.844633
H	1.180521	-2.838503	0.318434
H	0.003631	-2.662080	-1.732640
C	-2.957841	-1.186252	1.871525
H	-3.602331	-0.381691	2.259424
H	-3.592535	-2.083917	1.815219
H	-2.153922	-1.370336	2.593195
C	-3.508143	-0.541429	-0.525871
H	-4.184742	-1.407478	-0.587763
H	-4.131977	0.308751	-0.212563
H	-3.125734	-0.331997	-1.529046

C	-1.100371	-0.859631	0.262500
H	-0.458706	-1.159772	1.090415
H	2.248803	0.528955	-1.123305
H	2.782713	2.361774	0.220916
C	-1.660609	2.320101	-0.619719
H	-1.792076	3.408004	-0.658276
H	-2.154466	1.891401	-1.499466
H	-2.159031	1.946792	0.283673
H	1.100940	-1.446939	-2.392869
C	3.633131	-0.931578	0.687985
H	3.913183	-1.879049	1.166473
H	4.165527	-0.869499	-0.272424
H	3.989294	-0.115731	1.330157

G3-I3-TS G = -585.944974, T = -165

C	-0.539578	1.873185	-0.672168
C	-0.065396	0.411645	-0.430489
C	-2.021449	-0.989672	0.590379
C	0.041557	2.620872	0.558308
C	0.001728	-0.536535	-1.521255
C	1.446496	0.638838	0.053158
C	1.300444	1.830160	1.024674
H	-0.523173	-0.362968	-2.469800
H	0.268488	3.663716	0.305018
H	0.002299	2.215832	-1.572496
H	-0.709469	2.645997	1.360852
H	1.178410	1.466949	2.055064
C	0.915050	-1.682049	-1.456798
C	2.175213	-0.625862	0.548549
H	2.016060	-0.713592	1.635078
C	1.592101	-1.920864	-0.107431
H	2.379796	-2.675828	-0.228366
H	0.834662	-2.357580	0.560733
H	0.442501	-2.588363	-1.876472
C	-2.647520	-1.536765	1.849840
H	-3.632082	-1.073331	2.015195
H	-2.831364	-2.617513	1.743239
H	-2.030054	-1.373306	2.740159
C	-2.825362	-1.210319	-0.662426
H	-2.822523	-2.279694	-0.925278
H	-3.875691	-0.929746	-0.502825
H	-2.454283	-0.652749	-1.530766
C	-0.820425	-0.353102	0.674751
H	-0.318291	-0.371629	1.644768
H	1.964190	1.004019	-0.850375
H	2.213025	2.440439	1.009142
C	-2.034174	2.111180	-0.887383
H	-2.228377	3.188090	-0.975057
H	-2.405393	1.640025	-1.807865
H	-2.623022	1.735151	-0.040402
H	1.657515	-1.422985	-2.252604

C	3.690976	-0.496017	0.308757
H	4.231147	-1.340881	0.754979
H	3.922308	-0.476002	-0.766997
H	4.085361	0.427773	0.752293

I3 G = -585.982125

C	-0.140555	2.037843	-0.238615
C	-0.617088	0.610945	-0.184427
C	-2.858059	-0.599854	0.061377
C	1.369496	2.186244	0.089869
C	0.276565	-0.474226	-0.723816
C	1.819925	-0.175931	-0.694269
C	2.206841	1.298958	-0.824729
H	-0.032020	-0.547626	-1.789922
H	1.638276	3.243074	-0.037606
H	-0.212935	2.229960	-1.335988
H	1.533052	1.941080	1.150578
H	3.276664	1.413800	-0.599782
C	0.132716	-1.881102	-0.031472
C	2.295052	-0.900497	0.585971
H	1.908714	-0.345653	1.464360
C	1.542296	-2.239253	0.491555
H	2.062910	-2.890832	-0.227474
H	1.502366	-2.776216	1.448054
H	-0.583047	-1.823881	0.798319
C	-4.131482	-0.603516	0.850749
H	-4.954603	-0.213413	0.228008
H	-4.416171	-1.636464	1.101654
H	-4.073788	-0.003670	1.765147
C	-2.804502	-1.571226	-1.074383
H	-2.659105	-2.587922	-0.675493
H	-3.776817	-1.588586	-1.587547
H	-2.016025	-1.371836	-1.803435
C	-1.905242	0.372318	0.352924
H	-2.254855	1.134657	1.053554
H	2.257066	-0.727124	-1.544800
H	2.073304	1.630911	-1.867129
C	-1.002655	3.096871	0.465948
H	-0.579019	4.088043	0.266545
H	-2.040141	3.105577	0.109864
H	-1.002798	2.955634	1.556362
H	-0.238958	-2.635744	-0.733166
C	3.812163	-1.045369	0.710958
H	4.073579	-1.643003	1.594311
H	4.230201	-1.551545	-0.171863
H	4.311317	-0.072451	0.811611

Step I3 – J3

I3 G = -585.984886

C	0.957647	-0.457506	1.451207

C	0.314806	-0.447183	-0.001862
C	-2.851041	-0.826911	-0.140604
C	-2.110285	0.335202	-0.345029
C	2.491110	-0.540668	1.233543
C	-0.731284	0.585265	-0.149410
C	1.538432	-0.235544	-0.984751
C	2.680576	-0.976992	-0.232482
H	3.650705	-0.631936	-0.623286
H	2.953463	-1.241195	1.942186
H	0.567918	-1.330756	1.990851
C	2.603857	-2.503530	-0.403581
H	1.678388	-2.925052	0.017293
H	2.651144	-2.788848	-1.463299
H	3.441763	-2.989408	0.113174
H	2.950632	0.442148	1.405528
H	-2.694031	1.198090	-0.679931
H	-0.089087	-1.440726	-0.207730
H	1.305979	-0.751382	-1.928128
H	0.672979	0.421985	2.042112
C	-0.307991	2.032448	-0.182504
C	1.830838	1.254950	-1.288137
H	1.428185	1.505181	-2.282687
H	2.916561	1.412321	-1.345707
C	1.213281	2.224610	-0.269390
H	1.413866	3.264162	-0.562789
H	1.666972	2.100727	0.725003
H	-0.768494	2.444969	-1.102721
C	-0.930309	2.824072	1.006481
H	-0.646394	3.879016	0.910355
H	-0.545796	2.450404	1.963639
H	-2.024487	2.760289	1.024269
C	-4.300203	-0.815261	-0.511167
H	-4.911095	-0.877591	0.405962
H	-4.546035	-1.722926	-1.085194
H	-4.601932	0.071785	-1.077194
C	-2.358087	-2.101940	0.478547
H	-1.570486	-1.948781	1.221335
H	-1.959808	-2.761619	-0.311676
H	-3.188991	-2.646940	0.943468

I3-J3-TS G = -585.889571, T = -1252

C	1.250026	-0.345994	1.691613
C	0.435362	-0.285298	0.394651
C	-2.673864	-0.851918	-0.119570
C	-1.903757	0.092001	-0.746829
C	2.531054	-1.113949	1.125838
C	-0.582430	0.584515	-0.376500
C	1.508581	-0.010250	-0.606927
C	2.495163	-1.143524	-0.495464
H	3.470301	-0.850240	-0.909360
H	2.550446	-2.152300	1.478953

H	0.770831	-0.907498	2.502499
C	2.105539	-2.515871	-1.062044
H	1.153866	-2.883061	-0.655744
H	2.026918	-2.477309	-2.155901
H	2.878348	-3.249376	-0.801549
H	3.451334	-0.617721	1.454908
H	-2.345461	0.598874	-1.609681
H	0.095312	-1.314646	0.203304
H	0.186847	0.222518	-1.463433
H	1.524562	0.641631	2.079905
C	-0.398937	2.098658	-0.378166
C	2.026875	1.398186	-0.790563
H	2.243487	1.561689	-1.858878
H	3.014234	1.441711	-0.298510
C	1.093328	2.459891	-0.190232
H	1.301535	3.447809	-0.619852
H	1.297721	2.541144	0.886947
H	-0.766692	2.478946	-1.345854
C	-1.263711	2.720866	0.742564
H	-1.087123	3.803498	0.783490
H	-0.999631	2.295992	1.721316
H	-2.331967	2.547104	0.567120
C	-4.028894	-1.179858	-0.687989
H	-4.821864	-0.878826	0.014855
H	-4.130121	-2.270474	-0.807522
H	-4.216414	-0.700436	-1.655076
C	-2.335433	-1.566847	1.164255
H	-1.527856	-1.102135	1.735496
H	-2.065905	-2.615196	0.957641
H	-3.226118	-1.605348	1.807107

J3 G = -585.953635

C	0.421131	-1.626776	0.416919
C	0.448345	-0.441104	-0.594567
C	-2.953173	-0.520000	-0.236155
C	-2.081582	0.183896	-1.000586
C	1.880736	-2.114972	0.389532
C	-0.651622	0.653326	-0.799442
C	1.793996	0.126023	-0.435843
C	2.695842	-0.796412	0.296302
H	2.712131	-0.340427	1.317092
H	2.074789	-2.724207	-0.507914
H	-0.314036	-2.387108	0.139295
C	4.151016	-0.885619	-0.199611
H	4.182090	-1.278884	-1.223664
H	4.655601	0.087918	-0.181402
H	4.711496	-1.570253	0.447357
H	2.157994	-2.711568	1.265643
H	-2.481384	0.483755	-1.973717
H	0.540382	-0.931585	-1.596312
H	-0.389591	1.065265	-1.788533

H	0.168950	-1.254755	1.419388
C	-0.436021	1.895645	0.154326
C	2.030488	1.525268	-0.757263
H	1.674299	1.781299	-1.766219
H	3.068531	1.849766	-0.632848
C	1.066361	2.264050	0.280173
H	1.234536	3.335437	0.105125
H	1.434744	2.045013	1.292714
H	-0.942151	2.720193	-0.372196
C	-1.066864	1.790970	1.547830
H	-0.928565	2.734508	2.093129
H	-0.603956	0.996633	2.150992
H	-2.141803	1.596016	1.478535
C	-4.333737	-0.812386	-0.785179
H	-5.107047	-0.376524	-0.133463
H	-4.521283	-1.897677	-0.800558
H	-4.478382	-0.423495	-1.799906
C	-2.730975	-1.084737	1.145178
H	-1.793618	-0.783294	1.612280
H	-2.766994	-2.185446	1.115111
H	-3.550390	-0.773606	1.810380

Scheme S11

For **I3 – J3** cf. cartesian coordinates for Scheme S10.

Scheme S12

Step G4 – E4

G4 G = -585.975559

C	0.388205	2.107040	-0.061292
C	0.051243	0.631356	0.189136
C	-2.436290	-0.380421	-0.376418
C	1.755103	2.003619	-0.783845
C	-0.497256	0.102894	1.430239
C	0.866826	-0.264847	-0.747642
C	1.608722	0.748228	-1.656415
H	-0.585290	0.834458	2.240001
H	1.974595	2.913348	-1.355957
H	-0.332924	2.482618	-0.810607
H	2.559471	1.875941	-0.042025
H	2.564502	0.362835	-2.026691
C	-0.096426	-1.289247	1.927924
C	1.778267	-1.168799	0.142091
H	2.377307	-0.511909	0.799462
C	0.885758	-2.065734	1.019642
H	0.354047	-2.764067	0.354088
H	1.519500	-2.697313	1.657585
H	0.400693	-1.100562	2.891356
C	-3.666441	0.211866	-0.998716

H	-4.557319	-0.246757	-0.536860
H	-3.737147	1.297914	-0.878638
H	-3.718978	-0.047206	-2.067103
C	-2.169512	-1.808129	-0.717955
H	-1.489218	-2.313608	-0.034965
H	-3.118051	-2.361193	-0.767131
H	-1.744452	-1.861767	-1.735999
C	-1.673368	0.428927	0.454497
H	-2.074045	1.430374	0.617231
H	0.235821	-0.941470	-1.344442
H	0.986414	0.976419	-2.535201
C	0.362780	3.024456	1.163496
H	-0.636855	3.102850	1.614395
H	1.063457	2.676387	1.935006
H	0.663331	4.038255	0.871049
H	-0.984304	-1.889652	2.170038
C	2.733119	-2.028263	-0.698955
H	3.451427	-1.423691	-1.264568
H	3.309999	-2.700573	-0.050417
H	2.173649	-2.649304	-1.414327

G4-I4-TS G = -585.941818, T = -188

C	-0.648840	1.878729	-0.207536
C	-0.181950	0.503301	0.365087
C	-2.151589	-1.116962	-0.209319
C	0.632817	2.375862	-0.894444
C	0.497883	0.599621	1.643216
C	0.868218	-0.091154	-0.741571
C	1.155833	1.139208	-1.646894
H	0.358759	1.491111	2.268328
H	0.467958	3.242698	-1.546285
H	-1.396153	1.650336	-0.983276
H	1.361848	2.686090	-0.120330
H	2.223609	1.210497	-1.891731
C	1.338578	-0.463908	2.180509
C	2.124500	-0.757500	-0.150319
H	2.847032	0.037433	0.122573
C	1.759272	-1.508918	1.148985
H	0.937149	-2.213789	0.948517
H	2.605465	-2.091501	1.534962
H	2.198226	0.026412	2.681379
C	-3.269938	-1.987809	0.324023
H	-3.216624	-2.985750	-0.138685
H	-3.236863	-2.108460	1.412960
H	-4.248214	-1.565649	0.049101
C	-2.131981	-0.984372	-1.711810
H	-1.828254	-1.939083	-2.170567
H	-3.150518	-0.783187	-2.074132
H	-1.479600	-0.198275	-2.099851
C	-1.327935	-0.514746	0.683066
H	-1.546562	-0.693697	1.737405

H	0.326453	-0.871569	-1.285577
H	0.608731	1.029914	-2.595070
C	-1.294494	2.845160	0.788679
H	-2.127851	2.371744	1.326511
H	-0.577388	3.236789	1.525337
H	-1.695889	3.716150	0.255902
H	0.789240	-0.889175	3.051806
C	2.783587	-1.682237	-1.183039
H	3.016108	-1.144768	-2.111683
H	3.720490	-2.098945	-0.791668
H	2.119084	-2.520965	-1.437723

I4 G = -585.984582

C	-0.895159	1.563725	0.170209
C	-0.666129	0.086283	0.365998
C	-2.897773	-0.907018	-0.384130
C	0.425901	2.350416	-0.004051
C	0.713737	-0.255337	0.744230
C	1.599921	0.149665	-0.520982
C	1.414625	1.624303	-0.937946
H	1.041078	0.443852	1.535839
H	0.184533	3.351174	-0.388361
H	-1.511930	1.710793	-0.729613
H	0.892267	2.511128	0.980304
H	2.380964	2.148074	-0.922957
C	1.203085	-1.670330	1.067557
C	2.995552	-0.305033	-0.029096
H	3.352903	0.473912	0.668811
C	2.734188	-1.617348	0.772149
H	3.038806	-2.495543	0.186806
H	3.324436	-1.634015	1.697345
H	0.970097	-1.965317	2.099298
C	-3.684514	-2.177698	-0.410814
H	-3.935259	-2.433842	-1.454145
H	-3.174913	-3.025559	0.057629
H	-4.656192	-2.015041	0.085322
C	-3.570615	0.279579	-1.012544
H	-4.534821	0.000755	-1.449782
H	-3.746948	1.075817	-0.276576
H	-2.945579	0.714962	-1.806204
C	-1.622963	-0.938074	0.181979
H	-1.307862	-1.922797	0.531335
H	1.290318	-0.518887	-1.343437
H	1.042766	1.677314	-1.971581
C	-1.696277	2.110936	1.391418
H	-2.645123	1.583310	1.541451
H	-1.104578	2.007985	2.309971
H	-1.903891	3.176058	1.233726
H	0.728549	-2.403948	0.399445
C	4.011591	-0.454888	-1.164500
H	4.163347	0.490821	-1.702701

H	4.985675	-0.773643	-0.768867
H	3.680324	-1.212444	-1.889925

Step I4 – J4

I4 G = -585.984583

C	-0.895176	1.563718	0.170360
C	-0.666009	0.086263	0.365973
C	-2.897691	-0.907084	-0.384185
C	0.425885	2.350341	-0.004174
C	0.713771	-0.255222	0.744344
C	1.599754	0.149474	-0.521182
C	1.414041	1.623995	-0.938465
H	1.041234	0.444384	1.535481
H	0.184553	3.351128	-0.388431
H	-1.512065	1.710829	-0.729364
H	0.892586	2.510987	0.980039
H	2.380379	2.147781	-0.924417
C	1.203406	-1.669955	1.068331
C	2.995495	-0.304822	-0.029255
H	3.352859	0.474377	0.668339
C	2.734414	-1.616947	0.772375
H	3.038849	-2.495243	0.187084
H	3.325003	-1.633401	1.697361
H	0.970809	-1.964173	2.100378
C	-3.684368	-2.177804	-0.410901
H	-3.933685	-2.434568	-1.454455
H	-3.175173	-3.025397	0.058492
H	-4.656702	-2.015066	0.083853
C	-3.570776	0.279557	-1.012271
H	-4.534553	0.000484	-1.450286
H	-3.748111	1.075056	-0.275702
H	-2.945507	0.716059	-1.805104
C	-1.622753	-0.938166	0.181611
H	-1.307577	-1.922934	0.530792
H	1.290225	-0.519405	-1.343394
H	1.041350	1.676626	-1.971825
C	-1.696163	2.110798	1.391673
H	-2.645015	1.583163	1.541652
H	-1.104416	2.007715	2.310178
H	-1.903772	3.175943	1.234112
H	0.728743	-2.404156	0.400973
C	4.011397	-0.454919	-1.164792
H	4.162952	0.490641	-1.703306
H	4.985573	-0.773426	-0.769186
H	3.680106	-1.212749	-1.889920

I4-J4-TS G = -585.963981, T = -452

C	0.311436	1.973527	0.273336
C	0.222907	0.570312	-0.367616
C	2.376113	-0.654320	0.185831

C	-1.111863	2.401194	0.725587
C	-1.037445	0.379280	-1.220928
C	-1.521078	0.031549	0.167742
C	-1.962057	1.152318	1.079453
H	-1.415200	1.302348	-1.666545
H	-1.053470	3.090281	1.576757
H	0.972705	1.889382	1.148613
H	-1.601007	2.952423	-0.089490
H	-3.031036	1.344297	0.898101
C	-1.167075	-0.902269	-2.059848
C	-1.935005	-1.431429	0.217078
H	-3.027076	-1.375466	0.030440
C	-1.247236	-2.049885	-1.027398
H	-0.237981	-2.387794	-0.748079
H	-1.791921	-2.923575	-1.403642
H	-2.101009	-0.834034	-2.636149
C	3.616805	-1.346156	-0.317421
H	3.663441	-2.377141	0.066676
H	3.667501	-1.378608	-1.411679
H	4.516427	-0.836307	0.059860
C	2.260189	-0.546803	1.685556
H	2.412328	-1.535086	2.144386
H	3.057655	0.100217	2.082416
H	1.302188	-0.152540	2.047772
C	1.445270	-0.203832	-0.692550
H	1.591959	-0.396664	-1.757466
H	-0.242642	-0.076180	0.635450
H	-1.862959	0.850685	2.131761
C	0.946818	2.973029	-0.708427
H	1.965832	2.668705	-0.976837
H	0.355511	3.060052	-1.630565
H	0.995427	3.966513	-0.245333
H	-0.350554	-1.029885	-2.781634
C	-1.685881	-2.157526	1.541900
H	-2.197824	-1.668219	2.380499
H	-2.050228	-3.190347	1.484081
H	-0.609468	-2.196971	1.766834

J4 G = -585.973559

C	-0.051448	2.073172	-0.519817
C	-0.378853	0.590344	-0.162739
C	-2.616268	-0.613195	-0.305613
C	1.451440	2.404370	-0.286795
C	0.621912	0.146369	1.137908
C	1.796294	0.014121	0.316216
C	2.360341	1.169225	-0.413376
H	0.553457	1.016166	1.802576
H	1.781839	3.182738	-0.986109
H	-0.256844	2.155183	-1.599519
H	1.570905	2.827940	0.722130
H	3.364870	1.380096	0.002221

C	0.340677	-1.236162	1.744201
C	2.198481	-1.414081	0.162827
H	3.025564	-1.496730	0.908706
C	0.986824	-2.207289	0.735860
H	0.291324	-2.434883	-0.086933
H	1.292699	-3.160392	1.181578
H	0.841605	-1.297892	2.722347
C	-4.013596	-0.772280	0.239708
H	-4.199173	-1.822605	0.515230
H	-4.198353	-0.143166	1.118155
H	-4.757967	-0.521975	-0.531812
C	-2.296036	-1.475579	-1.499944
H	-2.332705	-2.539129	-1.215033
H	-3.064031	-1.343079	-2.276582
H	-1.320540	-1.277856	-1.957998
C	-1.762508	0.274678	0.271177
H	-2.112285	0.811441	1.157112
H	-0.107227	-0.038649	-1.023331
H	2.558155	0.873329	-1.458334
C	-0.931882	3.098988	0.209339
H	-1.991712	2.973170	-0.042381
H	-0.827799	3.021293	1.302173
H	-0.634177	4.116521	-0.075287
H	-0.729020	-1.416895	1.899289
C	2.735791	-1.866797	-1.201770
H	3.635512	-1.312722	-1.496732
H	2.996293	-2.931069	-1.162600
H	1.972597	-1.736146	-1.981947

Scheme S13

Step N4 – H4

**N4**	G = -585.976193		
C	-0.191912	1.758384	0.143058
C	0.094186	0.284642	0.069677
C	-2.744698	-0.669710	-0.315773
C	0.983556	2.395738	-0.665063
C	-0.442128	-0.802724	0.895255
C	1.220139	-0.008252	-0.851726
C	1.446624	1.297039	-1.643517
H	-0.932811	-0.455737	1.813426
H	0.675981	3.321313	-1.164795
H	-1.127186	1.923454	-0.428055
H	1.792780	2.656145	0.034541
H	2.489130	1.418692	-1.961384
C	0.621251	-1.896459	1.155846
C	2.455299	-0.443369	0.077237
H	2.950562	0.488482	0.389726
C	1.989114	-1.212903	1.346525
H	2.747155	-1.959956	1.617775

H	1.914021	-0.517612	2.198665
H	0.334735	-2.479586	2.040134
C	-3.636859	-1.185572	-1.412911
H	-4.503173	-1.708493	-0.978791
H	-4.048373	-0.343427	-1.991839
H	-3.122931	-1.869152	-2.097619
C	-3.357305	0.343569	0.608387
H	-3.362636	1.337542	0.128347
H	-4.408767	0.096243	0.806110
H	-2.832392	0.438929	1.563994
C	-1.468816	-1.147689	-0.204910
H	-1.101462	-1.841618	-0.964153
H	1.014677	-0.886310	-1.482962
H	0.820788	1.293814	-2.548429
C	-0.369888	2.325302	1.563672
H	-0.485901	3.414535	1.506465
H	-1.251250	1.922300	2.075281
H	0.515242	2.112575	2.177774
H	0.662283	-2.602157	0.312370
C	3.434289	-1.261460	-0.778444
H	2.999933	-2.234216	-1.051744
H	3.704553	-0.736165	-1.703788
H	4.357929	-1.448822	-0.214933

N4-H4-TS G = -585.943696, T = -279

C	-0.122911	1.941640	-0.488733
C	0.022621	0.425895	-0.150203
C	-2.377428	-0.673469	-0.232008
C	1.298640	2.457377	-0.198855
C	0.051419	0.080977	1.262865
C	1.411971	0.053854	-0.860942
C	2.245497	1.366024	-0.745952
H	-0.347703	0.801940	1.986888
H	1.480038	3.448461	-0.632611
H	-0.293368	1.974386	-1.579088
H	1.428570	2.561131	0.894455
H	3.121298	1.226351	-0.097427
C	0.694084	-1.128944	1.796593
C	2.125880	-1.202235	-0.313777
H	3.173534	-1.117368	-0.654049
C	2.161058	-1.180976	1.221825
H	2.639632	-2.079959	1.633228
H	2.722126	-0.313060	1.596184
H	0.675366	-1.141406	2.891906
C	-3.306152	-1.533410	-1.057571
H	-3.705571	-2.355929	-0.443350
H	-4.175659	-0.942889	-1.383979
H	-2.822248	-1.960812	-1.942650
C	-3.003621	-0.097971	1.009173
H	-3.970578	0.368581	0.774610
H	-3.215379	-0.909788	1.722731

H	-2.381811	0.641654	1.523588
C	-1.096662	-0.513438	-0.665594
H	-0.774609	-1.130915	-1.507236
H	1.147946	-0.115295	-1.917405
H	2.627481	1.637936	-1.739000
C	-1.238876	2.738101	0.186397
H	-1.183432	3.785893	-0.135918
H	-2.232629	2.363161	-0.084821
H	-1.150444	2.744920	1.283220
H	0.181631	-2.035446	1.440621
C	1.585248	-2.533843	-0.863834
H	0.555311	-2.740058	-0.538204
H	1.587001	-2.537190	-1.961786
H	2.208885	-3.371779	-0.526217

H4 G = -585.954610

C	0.162006	2.003245	-0.472946
C	-0.022955	0.495171	-0.122481
C	-2.391734	-0.398365	-0.227664
C	1.645780	2.327288	-0.189972
C	0.257450	0.126510	1.232252
C	1.470507	-0.091169	-0.781528
C	2.466467	1.050354	-0.515379
H	0.711897	0.880287	1.887686
H	1.972389	3.199428	-0.769635
H	-0.012633	2.070115	-1.560173
H	1.777492	2.600872	0.869146
H	3.149392	0.795074	0.306783
C	0.350902	-1.259630	1.686191
C	1.858600	-1.511948	-0.360807
H	2.923405	-1.614551	-0.644001
C	1.802792	-1.671633	1.168216
H	1.974716	-2.707797	1.489849
H	2.557666	-1.045098	1.663480
H	0.331598	-1.347848	2.778740
C	-3.538297	-0.934079	-1.052303
H	-3.931194	-1.860962	-0.606398
H	-4.373008	-0.216616	-1.061977
H	-3.247121	-1.142504	-2.088222
C	-2.707340	-0.132559	1.221829
H	-3.677420	0.374163	1.321245
H	-2.778069	-1.072111	1.792440
H	-1.951195	0.495114	1.725679
C	-1.186410	-0.170639	-0.802300
H	-1.069479	-0.367151	-1.870491
H	1.160831	-0.075854	-1.838491
H	3.088566	1.185159	-1.410966
C	-0.835862	2.926969	0.231387
H	-0.689958	3.964222	-0.096025
H	-1.871035	2.643360	0.003401
H	-0.701880	2.904290	1.323296

H	-0.387954	-1.923060	1.229242
C	1.071342	-2.605484	-1.105141
H	-0.005604	-2.548198	-0.901544
H	1.207359	-2.512076	-2.190136
H	1.421744	-3.602442	-0.809046

Step H4 – I4

H4 G = -585.954627

C	0.162944	2.003444	-0.472918
C	-0.023101	0.495581	-0.122334
C	-2.391948	-0.397580	-0.227581
C	1.646892	2.326704	-0.189968
C	0.257856	0.126575	1.232073
C	1.470699	-0.091759	-0.781332
C	2.467051	1.049305	-0.514959
H	0.713224	0.879978	1.887261
H	1.973964	3.198501	-0.769877
H	-0.011737	2.070432	-1.560129
H	1.778725	2.600545	0.869066
H	3.149582	0.793783	0.307458
C	0.350451	-1.259681	1.686024
C	1.857815	-1.512846	-0.360946
H	2.922561	-1.616001	-0.644165
C	1.801821	-1.672986	1.168038
H	1.972843	-2.709417	1.489260
H	2.557270	-1.047301	1.663503
H	0.331129	-1.347745	2.778598
C	-3.538719	-0.932575	-1.052334
H	-3.932268	-1.859229	-0.606543
H	-4.372997	-0.214601	-1.062079
H	-3.247593	-1.141106	-2.088245
C	-2.707265	-0.132521	1.222134
H	-3.677197	0.374410	1.321937
H	-2.778235	-1.072378	1.792209
H	-1.950851	0.494577	1.726263
C	-1.186682	-0.169638	-0.802269
H	-1.070005	-0.365448	-1.870622
H	1.160749	-0.075936	-1.838201
H	3.089487	1.183690	-1.410384
C	-0.834426	2.927687	0.231509
H	-0.687906	3.964902	-0.095749
H	-1.869731	2.644660	0.003423
H	-0.700491	2.904706	1.323415
H	-0.389120	-1.922490	1.229330
C	1.069916	-2.605677	-1.105667
H	-0.006985	-2.547843	-0.902017
H	1.205963	-2.511978	-2.190631
H	1.419784	-3.602922	-0.809903

H4-I4-TS G = -585.954785

C	0.185339	2.012798	-0.471566
C	-0.044252	0.518339	-0.108118
C	-2.409471	-0.376882	-0.221167
C	1.676340	2.314661	-0.195892
C	0.290156	0.125320	1.218258
C	1.485934	-0.109496	-0.763201
C	2.485489	1.023086	-0.498297
H	0.810492	0.855068	1.851078
H	2.014124	3.170934	-0.792411
H	0.005863	2.078548	-1.558009
H	1.813900	2.606224	0.857208
H	3.158950	0.769825	0.332001
C	0.346268	-1.266072	1.673649
C	1.846650	-1.541047	-0.363014
H	2.910154	-1.655532	-0.646522
C	1.779375	-1.725696	1.164323
H	1.920274	-2.772440	1.465401
H	2.553347	-1.131589	1.670463
H	0.322164	-1.344688	2.767512
C	-3.551645	-0.909223	-1.053639
H	-3.942542	-1.841059	-0.616385
H	-4.389010	-0.194808	-1.059282
H	-3.257474	-1.108137	-2.090664
C	-2.730759	-0.129234	1.231143
H	-3.701596	0.376584	1.328377
H	-2.812326	-1.077854	1.785150
H	-1.978682	0.488260	1.748437
C	-1.205584	-0.134817	-0.793500
H	-1.094702	-0.306759	-1.866788
H	1.147429	-0.074695	-1.810219
H	3.115475	1.141155	-1.391180
C	-0.790671	2.960349	0.234331
H	-0.624802	3.993388	-0.096785
H	-1.832241	2.696368	0.011986
H	-0.651426	2.935097	1.325391
H	-0.420404	-1.903388	1.225235
C	1.039833	-2.605449	-1.128922
H	-0.035644	-2.531446	-0.924039
H	1.179030	-2.495240	-2.212013
H	1.371641	-3.613634	-0.850268

I4 G = -585.981619

C	-0.402216	2.107141	-0.172209
C	-0.681901	0.628249	-0.042106
C	-2.727840	-0.850016	-0.300597
C	1.035092	2.491028	0.210491
C	0.377228	-0.170713	0.590163
C	1.632888	0.087617	-0.392923
C	2.071452	1.562340	-0.455524
H	0.687487	0.304371	1.538096
H	1.212193	3.539107	-0.065721

H	-0.594933	2.380712	-1.226523
H	1.141791	2.445399	1.306069
H	3.042726	1.686383	0.044461
C	0.336055	-1.704515	0.723105
C	2.640638	-0.933163	0.179949
H	2.996710	-0.517517	1.140767
C	1.792254	-2.193837	0.459962
H	1.810374	-2.855814	-0.417821
H	2.194080	-2.772189	1.301483
H	-0.029663	-2.010357	1.710883
C	-3.858105	-1.224324	-1.212272
H	-3.929635	-2.321241	-1.279059
H	-4.818302	-0.890738	-0.786351
H	-3.754339	-0.807707	-2.219463
C	-2.761536	-1.465165	1.065722
H	-3.761288	-1.297802	1.496506
H	-2.644960	-2.557699	1.002021
H	-2.020003	-1.057658	1.754985
C	-1.839664	0.134764	-0.702202
H	-2.141829	0.702660	-1.589958
H	1.323492	-0.262217	-1.393722
H	2.213807	1.860078	-1.503629
C	-1.447712	2.879972	0.690315
H	-1.255139	3.955608	0.597754
H	-2.473408	2.676057	0.361665
H	-1.356735	2.604405	1.749378
H	-0.345719	-2.134362	-0.023043
C	3.841747	-1.173237	-0.738707
H	3.516373	-1.555941	-1.717089
H	4.418332	-0.253511	-0.904812
H	4.518306	-1.916883	-0.295878

For **I4 – J4** cf. cartesian coordinates for Scheme S12.

Scheme S14

Step G5 – H5

G5 G = -585.976123

C	0.520942	2.057113	0.465840
C	0.115108	0.594399	0.460550
C	1.818616	-1.436125	-0.471784
C	0.176212	2.515747	-0.965693
C	0.346633	-0.383911	1.517181
C	-0.981834	0.389585	-0.570976
C	-1.141309	1.777991	-1.255991
H	0.761523	0.048331	2.436563
H	0.091891	3.606268	-1.045325
H	-0.259206	2.480722	1.139739
H	0.974886	2.191138	-1.653587
C	-0.730373	-1.443085	1.807411

C	-2.245713	-0.175896	0.134361
H	-2.536463	0.548729	0.918386
C	-1.903633	-1.519543	0.802222
H	-2.788393	-1.897515	1.333489
H	-1.705499	-2.251427	0.005062
H	-0.265537	-2.427530	1.956145
C	3.177694	-1.388641	-1.108786
H	3.716644	-2.325123	-0.889961
H	3.078950	-1.351986	-2.205187
H	3.789727	-0.545783	-0.771146
C	0.889274	-2.467834	-1.022188
H	1.455811	-3.328915	-1.399572
H	0.141971	-2.810423	-0.306730
H	0.354707	-2.054317	-1.897228
C	1.553320	-0.550476	0.559616
H	2.398768	0.069387	0.851562
H	-0.673698	-0.352017	-1.327227
C	1.881861	2.491345	1.010266
H	1.909217	3.584673	1.092132
H	2.088704	2.084586	2.009757
H	2.692776	2.195607	0.330545
H	-1.138744	-1.157725	2.788667
C	-3.411466	-0.326845	-0.852151
H	-3.689534	0.631537	-1.307590
H	-3.147954	-1.023431	-1.662113
H	-4.298678	-0.724329	-0.342168
H	-1.364782	1.687636	-2.324872
H	-1.979083	2.318975	-0.788618

G5-H5-TS G = -585.954094, T = -76

C	-0.124751	1.961136	0.535490
C	0.205295	0.378761	0.293888
C	2.308927	-0.940932	-0.387617
C	-0.277306	2.412544	-0.927119
C	0.119533	-0.295987	1.564538
C	-0.860132	-0.012510	-0.788812
C	-1.036155	1.278496	-1.649455
H	0.904918	-0.074214	2.295839
H	-0.805792	3.375821	-0.961088
H	-1.116457	1.971539	1.014735
H	0.715932	2.577974	-1.370538
C	-0.968488	-1.180785	1.982914
C	-2.185893	-0.517059	-0.165493
H	-2.668733	0.332822	0.354265
C	-1.926160	-1.626134	0.870902
H	-2.872732	-1.955584	1.319879
H	-1.505788	-2.512091	0.365775
H	-0.581124	-2.000990	2.612617
C	3.714121	-0.916923	-0.935939
H	4.392293	-1.469378	-0.266728
H	3.753705	-1.433183	-1.907602

H	4.103562	0.099313	-1.064436
C	1.725506	-2.314523	-0.186275
H	1.798778	-2.901689	-1.113757
H	2.302908	-2.863677	0.573131
H	0.677489	-2.310285	0.134957
C	1.660649	0.232411	-0.155467
H	2.224730	1.155215	-0.300535
H	-0.447714	-0.830676	-1.399058
C	0.846784	2.770106	1.389035
H	0.495773	3.810411	1.425486
H	0.900741	2.409189	2.424461
H	1.863065	2.788976	0.976600
H	-1.501553	-0.541254	2.731024
C	-3.148749	-1.027627	-1.250205
H	-3.386127	-0.252104	-1.988143
H	-2.707650	-1.879185	-1.788813
H	-4.093965	-1.363497	-0.804111
H	-0.662591	1.136339	-2.670372
H	-2.102735	1.524377	-1.735897

H5 G = -585.957462

C	-0.233916	1.741583	0.779120
C	0.213227	0.213147	0.198893
C	2.478901	-0.719355	-0.454915
C	-0.574562	2.421206	-0.571281
C	0.136211	-0.520957	1.421691
C	-0.904695	-0.030931	-0.865725
C	-1.023869	1.338268	-1.584787
H	0.980617	-0.419896	2.112613
H	-1.354205	3.172093	-0.376638
H	-1.160445	1.590916	1.349615
H	0.308785	2.965587	-0.932248
C	-1.040926	-1.279092	1.868555
C	-2.241405	-0.529073	-0.258414
H	-2.713303	0.307347	0.290581
C	-1.995625	-1.679464	0.733784
H	-2.945560	-2.026762	1.160975
H	-1.561079	-2.538640	0.195512
H	-0.710588	-2.124232	2.496425
C	3.801383	-0.553300	-1.163430
H	4.632608	-0.800161	-0.484944
H	3.874733	-1.252400	-2.010770
H	3.950725	0.465582	-1.538883
C	2.217112	-2.093283	0.108615
H	2.450766	-2.869150	-0.635067
H	2.851155	-2.292703	0.985774
H	1.167751	-2.249288	0.420066
C	1.601449	0.309997	-0.386907
H	1.878117	1.255083	-0.857701
H	-0.539433	-0.808074	-1.556908
C	0.795978	2.469575	1.631762

H	0.440020	3.496078	1.798715
H	0.935369	2.008857	2.617498
H	1.770947	2.534528	1.134348
H	-1.548256	-0.609893	2.601151
C	-3.211641	-0.982197	-1.362216
H	-3.426753	-0.179045	-2.077360
H	-2.792417	-1.829014	-1.924937
H	-4.167150	-1.305149	-0.928849
H	-0.379468	1.362541	-2.471911
H	-2.048668	1.509036	-1.935525

Step H5 – I6

H5 G = -585.954177

C	0.041180	0.956429	1.285433
C	0.233562	-0.022829	0.082859
C	2.629126	-0.337113	-0.699075
C	-0.345137	2.288802	0.601998
C	-0.056950	-1.413316	0.206514
C	-1.199950	0.376640	-0.804014
C	-1.097317	1.921481	-0.704042
H	0.347279	-2.059458	-0.581297
H	-0.948714	2.909954	1.275548
H	-0.820104	0.608297	1.877188
H	0.561386	2.863764	0.368228
C	-1.113289	-1.978011	1.039545
C	-2.492175	-0.194695	-0.187576
H	-2.695093	0.345370	0.752099
C	-2.386037	-1.691356	0.125138
H	-3.263843	-2.064937	0.670200
H	-2.304303	-2.274577	-0.804926
H	-1.011173	-3.061805	1.170444
C	3.780723	0.093792	-1.575406
H	4.626252	0.437226	-0.959605
H	4.153909	-0.756693	-2.166579
H	3.503517	0.899836	-2.264887
C	2.942931	-1.461061	0.259730
H	3.850219	-1.225931	0.835443
H	2.141449	-1.665928	0.988775
H	3.148216	-2.396849	-0.282401
C	1.432398	0.289895	-0.779694
H	1.342571	1.126974	-1.474308
H	-1.059552	0.001901	-1.827333
C	1.254035	1.047946	2.214365
H	1.057371	1.787951	3.001420
H	1.466081	0.089110	2.705871
H	2.151441	1.362769	1.667614
H	-1.241008	-1.481855	2.007387
C	-3.680661	0.052004	-1.147352
H	-3.813436	1.119018	-1.361445
H	-3.529938	-0.471208	-2.101964

H	-4.611685	-0.315867	-0.699313
H	-0.586499	2.323567	-1.586420
H	-2.118972	2.327718	-0.720271

H5-I6-TS G = -585.954259, T = -120

C	0.029122	1.068383	1.238183
C	0.308935	-0.001757	0.158399
C	2.672125	-0.403050	-0.660007
C	-0.423295	2.316628	0.440028
C	-0.070254	-1.354239	0.294986
C	-1.220505	0.276653	-0.846726
C	-1.079100	1.812963	-0.875176
H	0.312777	-2.032143	-0.476135
H	-1.107461	2.929553	1.039874
H	-0.817128	0.728614	1.854958
H	0.442885	2.949963	0.205210
C	-1.142650	-1.878770	1.152187
C	-2.515619	-0.222899	-0.190462
H	-2.717570	0.405706	0.692672
C	-2.425023	-1.686907	0.258448
H	-3.307060	-1.992297	0.836980
H	-2.361558	-2.350233	-0.618352
H	-1.000650	-2.948502	1.353430
C	3.820325	-0.003136	-1.555012
H	4.680205	0.328892	-0.953014
H	4.168781	-0.867509	-2.141066
H	3.551222	0.800931	-2.250070
C	2.971117	-1.537929	0.291113
H	3.918307	-1.345487	0.815948
H	2.197799	-1.695409	1.057655
H	3.105577	-2.484446	-0.254911
C	1.496591	0.267401	-0.717603
H	1.438391	1.124788	-1.390121
H	-1.035220	-0.201153	-1.816695
C	1.227825	1.312049	2.163121
H	0.980037	2.096806	2.889685
H	1.495235	0.406996	2.724640
H	2.108088	1.636884	1.594456
H	-1.255782	-1.335156	2.096840
C	-3.689503	-0.045435	-1.185183
H	-3.820379	1.001532	-1.482744
H	-3.529140	-0.643141	-2.093176
H	-4.624510	-0.378465	-0.718440
H	-0.495138	2.115170	-1.751532
H	-2.088248	2.228380	-1.013875

I6 G = -585.986371

C	-0.001967	1.629908	0.612777
C	0.701450	0.381793	0.170736
C	3.002058	-0.637481	-0.230910
C	-0.937840	2.156868	-0.548790

C	-0.194927	-0.767301	-0.146814
C	-1.534644	-0.312019	-0.855595
C	-1.383830	1.057053	-1.526619
H	0.323396	-1.488885	-0.785938
H	-1.798196	2.640092	-0.067437
H	-0.698852	1.277995	1.396638
H	-0.391434	2.943346	-1.088329
C	-0.674528	-1.490834	1.186161
C	-2.658278	-0.467938	0.197184
H	-2.655814	0.412488	0.866148
C	-2.191081	-1.693078	1.000779
H	-2.707239	-1.807586	1.963200
H	-2.378722	-2.607065	0.413985
H	-0.130070	-2.430862	1.342138
C	4.437937	-0.286223	-0.451100
H	5.033227	-0.654213	0.403069
H	4.827453	-0.827275	-1.327977
H	4.616254	0.787532	-0.567883
C	2.685384	-2.101099	-0.264049
H	3.581937	-2.695963	-0.051277
H	1.887664	-2.387413	0.426278
H	2.364528	-2.376729	-1.284132
C	2.103705	0.400715	0.028109
H	2.573784	1.379516	0.141653
H	-1.723766	-1.064469	-1.640013
C	0.841808	2.763760	1.205833
H	0.174685	3.552384	1.575745
H	1.457156	2.423127	2.048756
H	1.497238	3.226762	0.454907
H	-0.470793	-0.856678	2.060389
C	-4.055285	-0.605629	-0.414732
H	-4.332438	0.287387	-0.991126
H	-4.102905	-1.472432	-1.090377
H	-4.814811	-0.745296	0.365644
H	-0.641957	0.966308	-2.336689
H	-2.330093	1.346963	-2.005184

Step I6 – J6

I6 G = -585.984366

C	0.272952	2.067940	0.348256
C	0.726354	0.650364	0.140552
C	2.887227	-0.712799	-0.019277
C	-1.214463	2.166178	0.723780
C	-0.306615	-0.378970	-0.100263
C	-1.653389	0.076446	-0.744721
C	-2.121128	1.499411	-0.323919
H	0.096435	-1.197381	-0.704057
H	-1.369551	1.713102	1.714691
H	0.895906	2.518439	1.140019
H	-1.478659	3.227754	0.824691

C	-0.722161	-1.010309	1.313560
C	-2.625720	-1.036211	-0.261156
H	-3.664461	-0.693245	-0.383083
C	-2.263770	-1.167076	1.233759
H	-2.747020	-0.371211	1.817723
H	-2.582895	-2.126631	1.662798
H	-0.207047	-1.970075	1.441760
C	4.373402	-0.598776	0.091206
H	4.840131	-0.953984	-0.842768
H	4.726069	0.413416	0.312519
H	4.734371	-1.288945	0.872728
C	2.371644	-2.078757	-0.372564
H	1.874229	-2.061247	-1.355148
H	3.187347	-2.807299	-0.424312
H	1.633440	-2.433804	0.356798
C	2.123886	0.431324	0.206713
H	2.706574	1.321024	0.463586
H	-1.538561	0.042789	-1.838754
C	0.597520	2.852876	-0.968285
H	0.226528	3.879419	-0.854895
H	1.675492	2.888775	-1.162278
H	0.106191	2.400532	-1.837664
H	-0.415733	-0.373333	2.152845
C	-2.462676	-2.360889	-1.025433
H	-2.621193	-2.219969	-2.103326
H	-1.465621	-2.806760	-0.888898
H	-3.193807	-3.099607	-0.671939
H	-2.170284	2.141612	-1.214478
H	-3.146257	1.452124	0.068185

I6-J6-TS G = -585.887605, T = -1240

C	0.358054	2.101399	0.397183
C	0.602960	0.643888	0.002432
C	2.701263	-0.797860	-0.002212
C	-1.149757	2.306579	0.694977
C	-0.414627	-0.440205	0.442647
C	-1.455732	0.086743	-0.485142
C	-2.025441	1.465204	-0.245332
H	-0.019167	-1.356099	-0.023642
H	-1.355870	2.007686	1.730937
H	0.911437	2.232352	1.345439
H	-1.410509	3.369319	0.616015
C	-1.282591	-0.916299	1.614898
C	-2.392081	-1.055900	-0.777994
H	-3.361636	-0.680520	-1.134687
C	-2.496069	-1.524149	0.772341
H	-3.454847	-1.189111	1.184695
H	-2.473072	-2.620669	0.791962
H	-0.811469	-1.679367	2.245621
C	4.074755	-0.994548	-0.587219
H	4.187147	-2.033502	-0.936314

H	4.286994	-0.317796	-1.422360
H	4.845469	-0.851378	0.186604
C	2.332739	-1.773852	1.086460
H	2.103709	-2.760787	0.653176
H	3.198433	-1.928023	1.746210
H	1.490380	-1.456673	1.706044
C	1.945039	0.262158	-0.429289
H	2.411051	0.932281	-1.153442
H	-0.108506	0.598167	-1.176885
C	0.906106	3.115022	-0.624037
H	0.681416	4.134595	-0.288178
H	1.993077	3.042561	-0.744050
H	0.440132	2.975642	-1.610913
H	-1.625262	-0.104055	2.266266
C	-1.915144	-2.176191	-1.712259
H	-1.796491	-1.803609	-2.737569
H	-0.962847	-2.615622	-1.387486
H	-2.660631	-2.980657	-1.726006
H	-2.219772	1.950486	-1.215112
H	-3.026011	1.322853	0.198950

J6 G = -585.964096

C	0.418123	1.585742	0.364269
C	0.650813	0.588344	-0.808114
C	2.792414	-0.689859	-0.017605
C	-0.876656	2.391055	0.123736
C	-0.554297	-0.451765	-0.819686
C	-1.810959	0.146854	-0.359049
C	-2.058768	1.570789	-0.568193
H	-0.735724	-0.682859	-1.893309
H	-1.261964	2.771149	1.078480
H	0.276074	1.002446	1.291512
H	-0.670339	3.256273	-0.520357
C	-0.448110	-1.798965	-0.051377
C	-2.604203	-0.802576	0.454127
H	-2.323674	-0.476033	1.489525
C	-1.922224	-2.172379	0.197725
H	-2.062857	-2.867583	1.033115
H	-2.368655	-2.630822	-0.698982
H	0.113516	-2.552472	-0.611452
C	4.068267	-1.360231	-0.474551
H	4.095139	-2.408726	-0.137633
H	4.189825	-1.342358	-1.563823
H	4.944317	-0.870712	-0.021000
C	2.599863	-0.709598	1.479332
H	2.592996	-1.750364	1.839782
H	3.454150	-0.226269	1.978187
H	1.686941	-0.223298	1.830519
C	1.963873	-0.141138	-0.943191
H	2.277543	-0.267975	-1.983816
H	0.546440	1.186092	-1.730653

C	1.598029	2.548663	0.554303
H	1.386438	3.272297	1.352232
H	2.515817	2.009498	0.810436
H	1.788464	3.111089	-0.371090
H	0.071589	-1.637747	0.903718
C	-4.136990	-0.732521	0.342398
H	-4.527215	0.260581	0.595461
H	-4.456363	-0.986854	-0.676333
H	-4.583797	-1.457606	1.032178
H	-1.982367	1.771419	-1.650273
H	-3.041351	1.906339	-0.221171

Scheme 15

Step N5 – H5

N5 G = -585.973957

C	-0.457977	1.581043	-0.768751
C	0.012614	0.218086	-0.236873
C	-2.510740	-0.771575	0.319496
C	0.858478	2.404868	-0.637405
C	-0.211104	-1.090561	-0.830584
C	1.268394	0.397823	0.637237
C	1.450934	1.931332	0.703468
H	-0.779371	-1.126806	-1.764333
H	1.544086	2.156931	-1.462111
H	-0.782330	1.496758	-1.817047
H	0.663279	3.483286	-0.678994
H	0.894096	2.361774	1.547513
C	0.902003	-2.125693	-0.688024
C	2.546585	-0.301697	0.011990
H	3.098188	0.487230	-0.523634
C	2.228099	-1.416336	-1.011131
H	2.156596	-0.993541	-2.025998
H	3.048307	-2.147433	-1.030582
H	0.931620	-2.536943	0.332776
C	-3.359105	-0.998964	1.529694
H	-3.912207	-0.072429	1.757145
H	-4.129966	-1.756494	1.317420
H	-2.787369	-1.297718	2.413865
C	-3.240242	-0.528969	-0.966122
H	-3.520832	-1.518122	-1.370900
H	-4.179349	0.012507	-0.799626
H	-2.650969	-0.013503	-1.728508
C	-1.129714	-0.902377	0.421840
H	-0.734031	-1.267321	1.369279
H	1.111484	-0.040437	1.635343
H	2.504061	2.209129	0.833060
C	-1.570900	2.254357	0.060771
H	-1.721246	3.282088	-0.292113
H	-2.530445	1.734284	-0.029196

H	-1.313709	2.297107	1.126279
H	0.709379	-2.969326	-1.362876
C	3.440807	-0.810233	1.155486
H	4.408157	-1.157547	0.769898
H	3.636402	-0.018830	1.892138
H	2.967378	-1.651253	1.684637

N5-H5-TS G = -585.940220, T = -146

C	-0.149813	1.544456	-0.941122
C	-0.114547	0.150976	-0.193443
C	-2.540554	-0.551863	0.469424
C	1.017586	2.365347	-0.311108
C	-0.049389	-1.031767	-1.033719
C	1.226712	0.168344	0.717484
C	1.345811	1.667507	1.018768
H	-0.238214	-0.958667	-2.112688
H	1.896528	2.359185	-0.970936
H	0.069212	1.355143	-2.005823
H	0.734514	3.415584	-0.167310
H	0.607773	1.945492	1.787268
C	0.532322	-2.260138	-0.521379
C	2.408379	-0.446806	-0.073881
H	2.538566	0.153659	-0.991276
C	2.107673	-1.901812	-0.508934
H	2.524989	-2.113094	-1.500433
H	2.532140	-2.629037	0.198578
H	0.244799	-2.455210	0.516915
C	-3.580795	-0.639385	1.560579
H	-4.427336	0.025309	1.330073
H	-3.992763	-1.659364	1.612360
H	-3.185804	-0.371860	2.546928
C	-3.022121	-0.941761	-0.902994
H	-3.178157	-2.029995	-0.959457
H	-3.991590	-0.473107	-1.123988
H	-2.325920	-0.662135	-1.706866
C	-1.290741	-0.126184	0.778135
H	-1.052131	0.051942	1.829871
H	1.068223	-0.424030	1.631804
H	2.333884	1.930766	1.417814
C	-1.485913	2.295074	-0.855937
H	-1.413111	3.227593	-1.430114
H	-2.323356	1.717821	-1.266035
H	-1.734426	2.556695	0.181167
H	0.357719	-3.141928	-1.145106
C	3.723300	-0.399702	0.723248
H	4.530783	-0.894417	0.168294
H	4.040666	0.631728	0.917270
H	3.615564	-0.911787	1.690977

H5 G = -585.942496

C	-0.152412	1.669063	-0.934989

C	-0.183267	0.249539	-0.241000
C	-2.490774	-0.575235	0.478147
C	1.133895	2.359047	-0.399276
C	-0.027081	-0.872213	-1.140119
C	1.212220	0.172012	0.695047
C	1.438408	1.668442	0.938358
H	0.117411	-0.684629	-2.212699
H	1.970713	2.213500	-1.097785
H	-0.057155	1.508503	-2.022221
H	0.995942	3.441488	-0.284845
H	0.747249	2.023581	1.717084
C	0.349979	-2.185657	-0.637954
C	2.355621	-0.575753	-0.022730
H	2.595130	-0.020277	-0.946392
C	1.937991	-2.010621	-0.425864
H	2.457356	-2.322122	-1.339953
H	2.183816	-2.733570	0.365544
H	-0.107172	-2.402930	0.332296
C	-3.601447	-0.617844	1.502441
H	-4.495232	-0.103121	1.118220
H	-3.900704	-1.657966	1.703182
H	-3.311512	-0.149631	2.449713
C	-2.797048	-1.221985	-0.843946
H	-2.783390	-2.320391	-0.774408
H	-3.788622	-0.928163	-1.217715
H	-2.062864	-0.944396	-1.632455
C	-1.320990	0.039683	0.757809
H	-1.175909	0.490365	1.741908
H	0.951442	-0.371976	1.614664
H	2.457131	1.860408	1.301790
C	-1.415365	2.509421	-0.698297
H	-1.339190	3.447788	-1.262406
H	-2.323769	1.988429	-1.026575
H	-1.542523	2.765239	0.362268
H	0.173067	-3.002085	-1.345782
C	3.624455	-0.631880	0.847783
H	4.403548	-1.226164	0.353466
H	4.036904	0.367866	1.028006
H	3.413217	-1.095861	1.822632

Step H5 – I5

H5 G = -585.942493

C	-0.152350	1.668964	-0.935129
C	-0.183200	0.249473	-0.241065
C	-2.490738	-0.575232	0.478206
C	1.133769	2.359088	-0.399125
C	-0.027129	-0.872376	-1.140096
C	1.212117	0.172001	0.695052
C	1.438125	1.668445	0.938539
H	0.117120	-0.684972	-2.212738

H	1.970740	2.213667	-1.097473
H	-0.056812	1.508360	-2.022333
H	0.995665	3.441508	-0.284656
H	0.746777	2.023461	1.717158
C	0.350205	-2.185691	-0.637730
C	2.355665	-0.575575	-0.022716
H	2.595175	-0.019988	-0.946312
C	1.938179	-2.010450	-0.426101
H	2.457348	-2.321591	-1.340431
H	2.184420	-2.733589	0.365007
H	-0.106728	-2.402770	0.332670
C	-3.601438	-0.617661	1.502488
H	-4.495147	-0.102835	1.118226
H	-3.900808	-1.657739	1.703267
H	-3.311450	-0.149442	2.449739
C	-2.796973	-1.222230	-0.843767
H	-2.783383	-2.320625	-0.774021
H	-3.788518	-0.928434	-1.217633
H	-2.062748	-0.944820	-1.632299
C	-1.320978	0.039756	0.757761
H	-1.175865	0.490619	1.741772
H	0.951391	-0.372081	1.614631
H	2.456761	1.860513	1.302161
C	-1.415469	2.509158	-0.698775
H	-1.339310	3.447482	-1.262961
H	-2.323727	1.988003	-1.027201
H	-1.542891	2.765057	0.361739
H	0.173140	-3.002306	-1.345308
C	3.624433	-0.631665	0.847882
H	4.403619	-1.225829	0.353561
H	4.036776	0.368099	1.028232
H	3.413164	-1.095765	1.822667

H5-I5-TS G = -585.941940, T = -41

C	-0.219478	1.727731	-0.889113
C	-0.222161	0.285431	-0.250093
C	-2.494220	-0.608365	0.452040
C	1.159491	2.329918	-0.517233
C	0.017575	-0.786016	-1.174755
C	1.239255	0.189476	0.670420
C	1.517217	1.686853	0.830530
H	0.265386	-0.538587	-2.216098
H	1.916449	2.065104	-1.270972
H	-0.297306	1.611121	-1.983319
H	1.127174	3.425233	-0.462936
H	0.882665	2.097839	1.628963
C	0.328601	-2.138467	-0.723149
C	2.361478	-0.627153	-0.000301
H	2.667902	-0.095475	-0.917798
C	1.899368	-2.048156	-0.403113
H	2.469231	-2.400402	-1.271303

H	2.059232	-2.764530	0.415478
H	-0.206327	-2.393807	0.196422
C	-3.589800	-0.727161	1.486265
H	-4.494299	-0.198996	1.147979
H	-3.876476	-1.780981	1.624380
H	-3.291426	-0.317202	2.457785
C	-2.812908	-1.183243	-0.902035
H	-2.794286	-2.283930	-0.888877
H	-3.815927	-0.880142	-1.235305
H	-2.099758	-0.858710	-1.687573
C	-1.330463	0.009394	0.753647
H	-1.191047	0.423313	1.754424
H	0.943334	-0.305480	1.605870
H	2.562513	1.859118	1.121840
C	-1.386835	2.615293	-0.432460
H	-1.335173	3.578636	-0.955609
H	-2.359168	2.158720	-0.653386
H	-1.351579	2.818459	0.646412
H	0.164006	-2.901714	-1.491695
C	3.589293	-0.730260	0.925621
H	4.360824	-1.361101	0.466103
H	4.037870	0.251197	1.119161
H	3.317703	-1.179334	1.892428

I5 G = -585.980773

C	-0.216133	2.112079	-0.226787
C	-0.733423	0.688438	-0.151950
C	-2.993486	-0.472760	0.028812
C	1.053146	2.223406	-1.094495
C	0.247751	-0.377111	-0.476632
C	1.664495	0.050144	0.071666
C	2.209294	1.318262	-0.597079
H	0.359993	-0.344612	-1.584013
H	0.789275	1.948764	-2.127173
H	-1.022484	2.710182	-0.688465
H	1.368049	3.274864	-1.125889
H	2.845768	1.862840	0.113059
C	0.064047	-1.849478	-0.013617
C	2.466487	-1.254697	-0.072164
H	2.632647	-1.421346	-1.153682
C	1.473942	-2.315113	0.449510
H	1.714644	-3.326044	0.097323
H	1.518951	-2.336738	1.549295
H	-0.669186	-1.917116	0.802046
C	-4.324371	-0.411826	0.714113
H	-5.094600	-0.070972	0.001531
H	-4.634304	-1.421878	1.022173
H	-4.332178	0.256129	1.581759
C	-2.857274	-1.521930	-1.028858
H	-2.791093	-2.518751	-0.564835
H	-3.776257	-1.537894	-1.633882

H	-2.002049	-1.378441	-1.691475
C	-2.060289	0.522264	0.302717
H	-2.459994	1.373030	0.866407
H	1.539111	0.230400	1.154807
H	2.849794	1.051317	-1.450700
C	-0.005968	2.715711	1.199637
H	0.291833	3.764738	1.082626
H	-0.920350	2.684841	1.803320
H	0.785712	2.192854	1.746376
H	-0.303598	-2.477988	-0.832718
C	3.815475	-1.240202	0.648784
H	4.324033	-2.207891	0.543224
H	4.480710	-0.466243	0.243046
H	3.683416	-1.047165	1.723894

Step I5 – J5

I5 G = -585.980774

C	-0.216398	2.111999	-0.226796
C	-0.733564	0.688351	-0.151819
C	-2.993567	-0.472873	0.028744
C	1.052831	2.223359	-1.094592
C	0.247764	-0.377111	-0.476307
C	1.664604	0.050268	0.071674
C	2.209136	1.318568	-0.597033
H	0.359832	-0.344700	-1.583724
H	0.788902	1.948468	-2.127191
H	-1.022790	2.710075	-0.688433
H	1.367537	3.274872	-1.126242
H	2.845258	1.863360	0.113263
C	0.064320	-1.849521	-0.013267
C	2.466707	-1.254511	-0.072295
H	2.632658	-1.421167	-1.153841
C	1.474340	-2.314972	0.449631
H	1.715054	-3.325937	0.097547
H	1.519560	-2.336440	1.549411
H	-0.668832	-1.917324	0.802445
C	-4.324618	-0.411945	0.713703
H	-5.095011	-0.072470	0.000653
H	-4.633903	-1.421894	1.022822
H	-4.333020	0.256866	1.580677
C	-2.857070	-1.522151	-1.028805
H	-2.790566	-2.518875	-0.564602
H	-3.776003	-1.538491	-1.633865
H	-2.001837	-1.378563	-1.691396
C	-2.060428	0.522110	0.302880
H	-2.460222	1.372946	0.866394
H	1.539432	0.230441	1.154857
H	2.849944	1.051854	-1.450491
C	-0.006279	2.715529	1.199694
H	0.291649	3.764531	1.082808

H	-0.920714	2.684677	1.803295
H	0.785273	2.192557	1.746516
H	-0.303273	-2.478042	-0.832383
C	3.815838	-1.239864	0.648380
H	4.324515	-2.207478	0.542687
H	4.480893	-0.465809	0.242523
H	3.683963	-1.046866	1.723514

I5-J5-TS G = -585.961397, T = -408

C	0.086089	2.047467	-0.152617
C	-0.194083	0.530865	-0.076609
C	-2.574580	-0.219837	0.214672
C	1.495768	2.251933	-0.781607
C	0.380389	-0.276808	-1.246233
C	1.551404	-0.165016	-0.294936
C	2.417604	1.070502	-0.379066
H	0.450694	0.277942	-2.184007
H	1.418221	2.293076	-1.875336
H	-0.678340	2.451464	-0.837253
H	1.923947	3.208391	-0.459010
H	2.940216	1.240757	0.572004
C	0.042947	-1.774874	-1.339673
C	1.950310	-1.550161	0.197961
H	2.755850	-1.830421	-0.510326
C	0.700992	-2.416395	-0.098224
H	0.962138	-3.469916	-0.251695
H	0.016818	-2.371689	0.762843
H	-1.033982	-1.966612	-1.391302
C	-3.697725	-0.648478	1.126639
H	-4.521838	0.080065	1.092769
H	-4.117177	-1.606753	0.783109
H	-3.376325	-0.762836	2.168364
C	-2.960203	-0.015898	-1.229975
H	-3.445846	-0.918080	-1.630434
H	-3.710491	0.787432	-1.295343
H	-2.127444	0.250964	-1.888746
C	-1.343198	0.018914	0.728233
H	-1.205698	-0.061776	1.807392
H	0.780769	0.232106	0.727085
H	3.186689	0.898076	-1.146920
C	-0.060597	2.719779	1.220798
H	0.101043	3.800509	1.130452
H	-1.058211	2.557800	1.645970
H	0.682902	2.331249	1.934484
H	0.496631	-2.162080	-2.263616
C	2.487749	-1.623344	1.629451
H	2.777101	-2.652229	1.875441
H	3.369266	-0.984792	1.770351
H	1.715873	-1.314529	2.350839

J5 G = -585.969285

C	-0.448028	1.819356	0.071508
C	-0.367792	0.305127	0.422458
C	-2.787290	-0.519675	0.118423
C	0.657957	2.240993	-0.936978
C	0.351380	-0.530671	-0.858748
C	1.674534	-0.009891	-0.617168
C	1.955187	1.437215	-0.727555
H	-0.178090	-0.159563	-1.741992
H	0.305490	2.083598	-1.966893
H	-1.419960	2.011525	-0.401774
H	0.870656	3.313622	-0.842852
H	2.527424	1.761268	0.158365
C	0.432717	-2.053456	-0.674014
C	2.583156	-1.056056	-0.071528
H	3.140131	-1.363749	-0.990621
C	1.626314	-2.234122	0.285586
H	2.120441	-3.207305	0.190821
H	1.308554	-2.125961	1.334655
H	-0.505197	-2.478829	-0.299232
C	-3.968673	-1.219910	0.743901
H	-4.835395	-0.542747	0.791439
H	-4.280647	-2.074426	0.122539
H	-3.757666	-1.584209	1.755912
C	-3.035729	0.011586	-1.270451
H	-3.379766	-0.803799	-1.925402
H	-3.854453	0.748064	-1.251175
H	-2.172322	0.487396	-1.743578
C	-1.626177	-0.408371	0.817607
H	-1.587592	-0.908389	1.787928
H	0.319092	0.208370	1.279724
H	2.655991	1.588300	-1.571730
C	-0.387730	2.635995	1.374849
H	-0.555182	3.701728	1.173335
H	-1.155660	2.300049	2.084105
H	0.591819	2.534504	1.866213
H	0.645971	-2.514393	-1.650495
C	3.606420	-0.640830	0.994071
H	4.210479	-1.508025	1.285873
H	4.289860	0.135789	0.629112
H	3.098547	-0.266555	1.893956

Scheme S16

Step G6 – I6

G6 G = -585.977092

C	0.518857	1.886700	0.032900
C	-0.104680	0.563144	0.379250
C	2.355223	-1.048897	-0.446385
C	-0.560813	2.601757	-0.838543

C	0.421431	-0.522334	1.214059
C	-1.464658	0.464174	-0.201324
C	-1.459415	1.470111	-1.376365
H	1.181100	-0.195241	1.935493
H	-1.151269	3.274814	-0.197466
H	1.398391	1.672542	-0.599962
H	-0.108178	3.212818	-1.627673
H	-1.014320	0.988717	-2.262024
C	-0.697523	-1.360572	1.865557
C	-2.043842	-0.950389	-0.375592
H	-1.493966	-1.390466	-1.225212
C	-1.808194	-1.838062	0.888481
H	-2.746566	-1.901754	1.457637
H	-1.594579	-2.862878	0.554347
H	-0.239737	-2.223496	2.366686
C	2.868248	-1.907583	-1.571877
H	3.270168	-1.273929	-2.378418
H	3.709288	-2.526217	-1.222561
H	2.099801	-2.566924	-1.990524
C	3.334974	-0.065122	0.125794
H	4.320538	-0.536898	0.243870
H	3.486391	0.770612	-0.579493
H	3.032227	0.359160	1.087911
C	1.076140	-1.220471	0.002602
H	0.448447	-1.947040	-0.511206
H	-2.037844	0.955777	0.632288
H	-2.464369	1.811178	-1.646453
C	0.999340	2.691165	1.260541
H	1.395365	3.656722	0.923158
H	0.165613	2.888208	1.947178
H	1.792042	2.175705	1.815855
H	-1.139383	-0.734948	2.655718
C	-3.530367	-0.910757	-0.760906
H	-3.920083	-1.930223	-0.878471
H	-4.125365	-0.412457	0.018558
H	-3.692042	-0.380959	-1.708397

G6-I6-TS G = -585.946144, T = -126

C	0.329554	2.011201	0.040678
C	0.113330	0.463236	0.180716
C	2.319788	-0.657362	-0.479569
C	-0.747700	2.465888	-1.002963
C	0.113833	-0.150103	1.486457
C	-1.437275	0.245731	-0.277055
C	-1.497459	1.202613	-1.475806
H	0.374011	0.415568	2.389246
H	-1.452180	3.155210	-0.515503
H	1.337446	2.170301	-0.366380
H	-0.298909	3.011315	-1.841875
H	-1.009176	0.743375	-2.348253
C	-0.437551	-1.497274	1.684581

C	-1.844656	-1.228408	-0.458754
H	-1.663064	-1.506953	-1.508568
C	-0.978603	-2.182835	0.429523
H	-1.561046	-3.065387	0.724746
H	-0.123143	-2.548742	-0.155255
H	0.297319	-2.123566	2.227387
C	3.148229	-1.368377	-1.520417
H	3.999504	-0.740987	-1.826227
H	3.580852	-2.288889	-1.097639
H	2.570962	-1.632581	-2.413758
C	3.051184	-0.279849	0.782410
H	3.271218	-1.176093	1.383258
H	4.020605	0.180710	0.544724
H	2.494485	0.420321	1.422928
C	1.016482	-0.371454	-0.733057
H	0.585617	-0.720895	-1.672619
H	-2.047114	0.688034	0.530099
H	-2.540466	1.405977	-1.754717
C	0.210907	2.787342	1.359326
H	0.265191	3.865336	1.160154
H	-0.753616	2.601837	1.857681
H	1.020341	2.551827	2.065566
H	-1.221649	-1.350707	2.461245
C	-3.350068	-1.396212	-0.175671
H	-3.678847	-2.416524	-0.410539
H	-3.579795	-1.201061	0.882601
H	-3.948512	-0.702244	-0.780697

I6 G = -585.982800

C	0.394463	2.114624	-0.204235
C	0.642816	0.633520	-0.062101
C	2.727372	-0.824648	-0.295622
C	-1.063870	2.543325	0.024849
C	-0.361452	-0.174288	0.674782
C	-1.873972	0.193571	0.244400
C	-2.003379	1.484469	-0.559838
H	-0.283547	0.193568	1.720880
H	-1.262467	2.660957	1.103205
H	0.697766	2.395291	-1.228610
H	-1.215489	3.530516	-0.432217
H	-1.743193	1.302794	-1.617789
C	-0.291079	-1.727556	0.618689
C	-2.386123	-1.093753	-0.430733
H	-1.955502	-1.135340	-1.450310
C	-1.741962	-2.202692	0.414004
H	-2.264114	-2.275431	1.381854
H	-1.791245	-3.191352	-0.060195
H	0.321772	-2.035733	-0.240911
C	3.870948	-1.155050	-1.207035
H	4.814369	-0.770257	-0.785694
H	3.996421	-2.247498	-1.262268

H	3.746504	-0.751483	-2.217197
C	2.768350	-1.466713	1.056389
H	2.596878	-2.550773	0.967650
H	3.784077	-1.359845	1.466978
H	2.057311	-1.047324	1.770497
C	1.820779	0.149471	-0.690378
H	2.132737	0.732141	-1.565039
H	-2.426318	0.321591	1.190701
H	-3.045915	1.828328	-0.539990
C	1.374655	2.852601	0.764468
H	1.200674	3.931903	0.674146
H	1.185105	2.560570	1.805897
H	2.422727	2.641425	0.522794
H	0.161592	-2.159443	1.517832
C	-3.912753	-1.162851	-0.526524
H	-4.228944	-2.114151	-0.975036
H	-4.371561	-1.093642	0.470919
H	-4.318384	-0.350992	-1.144778

For **I6 – J6** cf. cartesian coordinates for Scheme S14.

Scheme S17

For **I6 – J6** cf. cartesian coordinates for Scheme S14.

Scheme S18

Step I7 – J7

I7 G = -585.969128

C	-0.604881	1.570562	-0.847210
C	-0.547232	0.078130	-0.671668
C	-2.798224	-0.790396	0.186464
C	0.690262	2.260836	-0.298274
C	0.822435	-0.447250	-0.682928
C	1.373596	0.035094	0.747202
C	1.207171	1.551476	0.970587
H	1.440668	0.092010	-1.418951
H	1.477770	2.262900	-1.063960
H	-0.506928	1.594999	-1.958792
H	0.459039	3.314031	-0.091941
H	0.493061	1.724794	1.790378
C	1.162221	-1.938125	-0.671939
C	2.788026	-0.616807	0.721537
H	3.110507	-0.753213	1.766115
C	2.552373	-2.003854	0.034875
H	3.352721	-2.209680	-0.688078
H	2.568809	-2.821589	0.766220
H	0.413487	-2.490528	-0.085160
C	-3.850049	-1.837962	-0.031435
H	-3.893337	-2.523518	0.830151

H	-4.840419	-1.358919	-0.080134
H	-3.688989	-2.423756	-0.942342
C	-3.011815	0.114537	1.362971
H	-3.891344	0.757082	1.203542
H	-3.249147	-0.503015	2.242984
H	-2.145677	0.733126	1.605755
C	-1.648956	-0.820210	-0.579604
H	-1.502580	-1.740196	-1.158169
H	0.777392	-0.513930	1.494320
H	2.154453	2.011547	1.281315
C	-1.895135	2.340337	-0.534783
H	-1.862734	3.302127	-1.060285
H	-2.791126	1.805837	-0.871953
H	-1.993695	2.558943	0.534577
H	1.175239	-2.369458	-1.681839
C	3.849034	0.217634	-0.008127
H	3.586630	0.391339	-1.062854
H	4.015836	1.193097	0.467000
H	4.807420	-0.318042	-0.003907

I7-J7-TS G = -585.959116, T = -453

C	-0.405000	2.032759	-0.626321
C	-0.324047	0.492410	-0.674494
C	-2.400024	-0.757889	0.174027
C	1.011981	2.583771	-0.276739
C	0.989043	-0.032296	-1.248345
C	1.314068	0.185429	0.221670
C	1.758145	1.573507	0.633996
H	1.467736	0.638491	-1.964897
H	1.593632	2.740380	-1.193464
H	-0.652926	2.327644	-1.660810
H	0.927618	3.561323	0.212960
H	1.563915	1.735366	1.703456
C	1.117966	-1.541153	-1.509300
C	1.702527	-1.153077	0.856423
H	1.303486	-1.245731	1.877131
C	1.087071	-2.201522	-0.109015
H	1.638745	-3.148954	-0.080266
H	0.050183	-2.413694	0.183729
H	0.335224	-1.927053	-2.174245
C	-3.595100	-1.601452	-0.193554
H	-3.547299	-2.578351	0.312081
H	-4.521739	-1.119383	0.152923
H	-3.674134	-1.772003	-1.273288
C	-2.249582	-0.470309	1.646420
H	-3.115724	0.101131	2.011941
H	-2.238698	-1.414272	2.211376
H	-1.350010	0.096867	1.915797
C	-1.532226	-0.360897	-0.786947
H	-1.695902	-0.713404	-1.809805
H	0.063532	0.272209	0.581535

H	2.844172	1.652978	0.483405
C	-1.496917	2.564279	0.311133
H	-1.590716	3.649669	0.185275
H	-2.471567	2.110501	0.097930
H	-1.248079	2.375311	1.364763
H	2.081438	-1.719580	-2.006160
C	3.245133	-1.221620	0.921422
H	3.700861	-1.085511	-0.068747
H	3.658083	-0.465336	1.599986
H	3.539941	-2.210236	1.295136

J7 G = -585.973457

C	-0.275904	2.119194	-0.301267
C	-0.518278	0.596344	-0.119290
C	-2.753044	-0.619570	0.035525
C	1.225469	2.465669	-0.525787
C	0.681187	-0.244416	-0.986003
C	1.737570	0.071093	-0.061236
C	2.163070	1.466307	0.176672
H	0.676056	0.272466	-1.953181
H	1.449890	2.460001	-1.601918
H	-0.821899	2.429806	-1.206038
H	1.439176	3.481829	-0.170700
H	2.256376	1.635942	1.263534
C	0.530955	-1.772553	-0.991390
C	2.179193	-1.127979	0.705464
H	2.289199	-0.910657	1.779571
C	1.097044	-2.199926	0.382212
H	1.513138	-3.214166	0.377588
H	0.309386	-2.169482	1.149032
H	-0.506516	-2.087668	-1.150386
C	-4.056881	-0.972108	-0.635770
H	-4.206663	-2.063412	-0.630257
H	-4.904004	-0.543752	-0.078219
H	-4.107438	-0.617905	-1.671781
C	-2.615260	-1.100254	1.457584
H	-3.479195	-0.769932	2.053459
H	-2.630671	-2.201319	1.483249
H	-1.705906	-0.760734	1.964844
C	-1.816070	0.098806	-0.639390
H	-2.020545	0.356596	-1.684014
H	-0.404991	0.339395	0.944365
H	3.197394	1.574403	-0.203910
C	-0.874999	2.864713	0.904539
H	-0.846817	3.949835	0.743457
H	-1.920768	2.571649	1.066342
H	-0.315784	2.645105	1.826588
H	1.134669	-2.184337	-1.813528
C	3.594757	-1.525765	0.162905
H	3.569192	-1.728951	-0.914990
H	4.335442	-0.741395	0.358346

H	3.908433	-2.440639	0.679932

Scheme S19

Step N7 – I7

N7 G = -585.978889

C	0.241924	1.830799	-0.536621
C	0.400558	0.389310	-0.214945
C	-2.711579	-0.538703	0.284217
C	0.974062	2.561700	0.620150
C	-0.340146	-0.755495	-0.768369
C	1.497281	0.199897	0.784349
C	2.132766	1.606607	0.956246
H	-0.847849	-0.497439	-1.706914
H	1.300670	3.565608	0.325852
H	0.936068	1.880266	-1.417980
H	0.283447	2.666146	1.472395
H	2.546015	1.748704	1.960954
C	0.482191	-2.062469	-0.896808
C	2.392549	-1.038758	0.473687
H	3.011588	-1.220284	1.365785
C	1.476814	-2.265778	0.258130
H	2.095450	-3.149449	0.047708
H	0.932079	-2.486566	1.189896
H	-0.219645	-2.904982	-0.958127
C	-3.585507	-0.770956	1.491638
H	-4.156154	0.142518	1.722489
H	-4.331421	-1.551768	1.278684
H	-3.017171	-1.063909	2.381523
C	-3.444235	-0.131951	-0.964919
H	-4.233764	-0.869380	-1.174762
H	-3.957954	0.829061	-0.813912
H	-2.811880	-0.046616	-1.852972
C	-1.373273	-0.789402	0.378434
H	-0.986801	-1.128325	1.341035
H	0.961071	-0.025976	1.731047
H	2.951279	1.743536	0.234807
C	-1.114607	2.383887	-0.970381
H	-1.017553	3.450758	-1.202760
H	-1.504986	1.880509	-1.861630
H	-1.840961	2.271645	-0.155546
H	1.021960	-2.026349	-1.854545
C	3.330131	-0.787748	-0.721217
H	2.782077	-0.463389	-1.618996
H	4.081628	-0.021305	-0.497175
H	3.869244	-1.708662	-0.978392

N7-I7-TS G = -585.946117, T = -165

C	-0.082291	1.834821	-0.600870
C	0.024504	0.340033	-0.121288

C	-2.385343	-0.597333	0.333894
C	0.799811	2.606265	0.399079
C	0.132614	-0.660326	-1.161977
C	1.367024	0.292878	0.839543
C	1.997154	1.677152	0.627330
H	-0.071435	-0.385568	-2.205173
H	1.083298	3.594956	0.017410
H	0.426467	1.870010	-1.583364
H	0.244852	2.759429	1.339433
H	2.613831	1.956806	1.492678
C	0.568623	-2.041685	-0.899764
C	2.242472	-0.957321	0.625325
H	2.809171	-1.111738	1.558182
C	1.320907	-2.186758	0.434946
H	1.898855	-3.119900	0.418596
H	0.607927	-2.258396	1.269550
H	-0.315734	-2.706755	-0.943951
C	-3.425009	-0.920763	1.380535
H	-4.296948	-0.259528	1.262455
H	-3.795743	-1.948621	1.242112
H	-3.046378	-0.820265	2.403541
C	-2.848713	-0.764461	-1.086885
H	-2.957491	-1.835431	-1.323203
H	-3.838509	-0.310034	-1.230842
H	-2.164401	-0.334125	-1.829714
C	-1.152127	-0.195042	0.739200
H	-0.938706	-0.235637	1.810049
H	0.962240	0.251128	1.862698
H	2.644870	1.685473	-0.260940
C	-1.487095	2.422366	-0.756917
H	-1.407415	3.472663	-1.065831
H	-2.082923	1.904544	-1.518642
H	-2.041582	2.393053	0.189652
H	1.166982	-2.366236	-1.768951
C	3.251551	-0.822341	-0.526867
H	2.781016	-0.479830	-1.464655
H	4.036427	-0.095839	-0.287294
H	3.736842	-1.785063	-0.735501

I7 G = -585.982477

C	-0.318274	2.028056	-0.531445
C	-0.624313	0.571318	-0.282190
C	-2.685654	-0.866484	0.117128
C	0.782149	2.501395	0.486678
C	0.523266	-0.345247	-0.467732
C	1.601814	0.139212	0.629445
C	2.010153	1.592780	0.431017
H	1.006720	-0.128830	-1.435658
H	1.032785	3.543009	0.243607
H	0.169448	2.054871	-1.523400
H	0.340378	2.500051	1.495898

H	2.725055	1.881871	1.217275
C	0.449089	-1.862018	-0.207961
C	2.673996	-0.971659	0.521784
H	3.245083	-0.984296	1.463822
C	1.834531	-2.271073	0.381741
H	2.344563	-3.002552	-0.258205
H	1.694225	-2.747641	1.360511
H	-0.345154	-2.084921	0.517153
C	-3.906463	-1.027613	0.972135
H	-4.816413	-0.852493	0.375197
H	-3.977564	-2.069931	1.319812
H	-3.917828	-0.356627	1.837449
C	-2.559031	-1.822028	-1.030110
H	-2.469432	-2.856757	-0.666093
H	-3.495186	-1.791091	-1.610131
H	-1.731667	-1.595807	-1.704944
C	-1.864271	0.233554	0.313472
H	-2.300453	1.016704	0.945011
H	1.104582	0.040299	1.611509
H	2.523769	1.717912	-0.533998
C	-1.520613	2.982649	-0.540803
H	-1.200902	3.962159	-0.916731
H	-2.328578	2.617708	-1.187458
H	-1.928291	3.143431	0.467064
H	0.215620	-2.415939	-1.124863
C	3.660594	-0.777418	-0.638877
H	3.153556	-0.736149	-1.615070
H	4.256457	0.137312	-0.526495
H	4.357386	-1.624840	-0.676961

For **I7 – J7** cf. cartesian coordinates for Scheme S18.

Step N7 – H7

N7 G = -585.978880

C	-0.241183	1.830723	0.536580
C	-0.400452	0.389269	0.215080
C	2.711341	-0.539220	-0.284141
C	-0.973175	2.561759	-0.620326
C	0.339890	-0.755856	0.768310
C	-1.497335	0.200222	-0.784130
C	-2.132410	1.607199	-0.955836
H	0.847784	-0.497924	1.706802
H	-1.299094	3.565946	-0.326223
H	-0.935249	1.880786	1.417929
H	-0.282617	2.665502	-1.472702
H	-2.546107	1.749299	-1.960355
C	-0.482827	-2.062534	0.896691
C	-2.392872	-1.038167	-0.473796
H	-3.011916	-1.219499	-1.365924
C	-1.477494	-2.265469	-0.258273

H	-2.096390	-3.148966	-0.047936
H	-0.932767	-2.486366	-1.190027
H	0.218726	-2.905295	0.957894
C	3.585425	-0.771532	-1.491441
H	4.155356	0.142296	-1.722742
H	4.331940	-1.551651	-1.278134
H	3.017306	-1.065259	-2.381215
C	3.443815	-0.132017	0.964982
H	3.956033	0.829857	0.814458
H	2.811595	-0.048295	1.853309
H	4.234498	-0.868403	1.174145
C	1.373079	-0.790072	-0.378483
H	0.986784	-1.129603	-1.340946
H	-0.961004	-0.025349	-1.730865
H	-2.950513	1.744449	-0.233992
C	1.115526	2.383541	0.970080
H	1.018695	3.450459	1.202385
H	1.505948	1.880225	1.861353
H	1.841772	2.271072	0.155189
H	-1.022500	-2.026362	1.854486
C	-3.330420	-0.787100	0.721156
H	-2.782237	-0.463202	1.619026
H	-4.081617	-0.020306	0.497315
H	-3.869877	-1.707891	0.978049

N7-H7-TS G = -585.930757, T = -71

C	0.013312	1.303072	0.912596
C	-0.012209	-0.150635	0.246000
C	2.558143	-0.684626	-0.265345
C	-0.084190	2.283540	-0.291493
C	-0.603086	-1.036447	1.251615
C	-0.829965	0.023391	-1.075458
C	-0.457214	1.450547	-1.546158
H	-0.095915	-1.090064	2.222737
H	-0.837960	3.050724	-0.071135
H	-0.955099	1.375013	1.436570
H	0.867189	2.817161	-0.430534
H	0.409158	1.387325	-2.218625
C	-1.818379	-1.821794	1.062282
C	-2.342148	-0.287762	-0.912228
H	-2.753352	-0.318782	-1.933777
C	-2.479308	-1.711379	-0.322033
H	-3.535173	-2.000711	-0.235168
H	-2.004499	-2.439454	-0.997888
H	-1.652922	-2.859862	1.411901
C	3.518004	-1.821441	-0.523270
H	3.876360	-1.798856	-1.563965
H	4.411199	-1.699225	0.109886
H	3.082865	-2.806968	-0.322473
C	3.140071	0.681916	-0.492600
H	2.401619	1.483116	-0.518987

H	3.889706	0.914954	0.280386
H	3.684926	0.683394	-1.448398
C	1.254812	-0.989138	0.015391
H	1.025613	-2.059048	-0.082114
H	-0.454405	-0.710654	-1.804871
H	-1.269932	1.906863	-2.125812
C	1.076586	1.596288	1.973369
H	0.878152	2.583561	2.411250
H	1.044892	0.869475	2.796860
H	2.094184	1.608415	1.577295
H	-2.479939	-1.431048	1.873269
C	-3.171936	0.746316	-0.129001
H	-2.900557	0.806028	0.934560
H	-3.063685	1.753139	-0.550293
H	-4.237393	0.484407	-0.172674

H7 G = -585.935653

C	-0.076108	0.938539	1.214355
C	0.033154	-0.432803	0.185695
C	2.623444	-0.523463	-0.392457
C	-0.217823	2.092583	0.163163
C	-0.719914	-1.198853	1.135605
C	-0.717559	0.034724	-1.103909
C	-0.292381	1.504576	-1.264143
H	-0.177289	-1.539642	2.026573
H	-1.115841	2.672955	0.404728
H	-1.025234	0.897455	1.775730
H	0.640859	2.767680	0.288664
H	0.688617	1.551736	-1.749316
C	-2.123438	-1.615471	0.981690
C	-2.268194	-0.178834	-1.088434
H	-2.556287	-0.204887	-2.151852
C	-2.598365	-1.552626	-0.481074
H	-3.676880	-1.753079	-0.523868
H	-2.095653	-2.351805	-1.047383
H	-2.208943	-2.620389	1.433884
C	3.765186	-1.495302	-0.605598
H	4.157155	-1.404699	-1.630254
H	4.602093	-1.253590	0.067888
H	3.475938	-2.539026	-0.437005
C	3.007921	0.909061	-0.660383
H	2.249518	1.646385	-0.399355
H	3.926127	1.154659	-0.105552
H	3.258514	1.033122	-1.725429
C	1.421926	-1.025333	-0.011660
H	1.409126	-2.112975	0.107562
H	-0.323515	-0.571152	-1.933174
H	-0.985058	2.063960	-1.905173
C	1.056134	1.054659	2.229034
H	0.891443	1.968716	2.816945
H	1.073234	0.213948	2.932983

H	2.037905	1.121703	1.754779
H	-2.757051	-0.993532	1.645854
C	-3.125730	0.926054	-0.438666
H	-2.931919	1.054631	0.636134
H	-2.977726	1.900032	-0.918462
H	-4.188653	0.672724	-0.544204

Step H7 – I8

H7 G = -585.935655

C	-0.076057	0.938137	1.214500
C	0.033331	-0.432663	0.185704
C	2.623451	-0.523384	-0.392680
C	-0.218234	2.092583	0.163774
C	-0.719862	-1.198820	1.135513
C	-0.717485	0.035027	-1.103883
C	-0.292504	1.504969	-1.263704
H	-0.177403	-1.539454	2.026652
H	-1.116455	2.672541	0.405520
H	-1.025118	0.896793	1.776054
H	0.640196	2.767957	0.289525
H	0.688577	1.552397	-1.748680
C	-2.123224	-1.615889	0.981246
C	-2.268103	-0.178683	-1.088474
H	-2.556135	-0.204536	-2.151916
C	-2.598133	-1.552657	-0.481467
H	-3.676641	-1.753186	-0.524342
H	-2.095361	-2.351630	-1.047994
H	-2.207891	-2.621256	1.432687
C	3.765413	-1.494948	-0.605497
H	4.158245	-1.403745	-1.629759
H	4.601745	-1.253483	0.068801
H	3.476163	-2.538782	-0.437641
C	3.007434	0.909253	-0.660869
H	2.249488	1.646432	-0.398118
H	3.926724	1.154770	-0.107849
H	3.255845	1.033579	-1.726400
C	1.421968	-1.025343	-0.011870
H	1.409152	-2.112974	0.107413
H	-0.323364	-0.570602	-1.933285
H	-0.985151	2.064457	-1.904675
C	1.056204	1.054178	2.229219
H	0.891779	1.968405	2.816942
H	1.072969	0.213620	2.933361
H	2.038043	1.120781	1.755036
H	-2.757246	-0.995040	1.645994
C	-3.125787	0.925938	-0.438451
H	-2.932011	1.054266	0.636394
H	-2.977957	1.900048	-0.918036
H	-4.188671	0.672448	-0.544039

H7-I8-TS G = -585.928707, T = -222

C	-0.085182	0.975946	1.123286
C	0.041412	-0.432069	0.304634
C	2.632637	-0.525179	-0.341570
C	-0.105968	2.087611	0.027197
C	-0.717198	-1.410845	1.058765
C	-0.727869	-0.029158	-1.085368
C	-0.191359	1.389349	-1.342270
H	-0.155247	-2.228247	1.529638
H	-0.974443	2.734662	0.206497
H	-1.078712	0.965914	1.593175
H	0.777860	2.733872	0.106092
H	0.803794	1.315862	-1.796434
C	-2.174566	-1.478033	1.112548
C	-2.272967	-0.176967	-1.066558
H	-2.531332	-0.290849	-2.136547
C	-2.677621	-1.490057	-0.390076
H	-3.766874	-1.628783	-0.365297
H	-2.243228	-2.357059	-0.907911
H	-2.522748	-2.348767	1.677774
C	3.725522	-1.515871	-0.689712
H	4.105871	-1.326245	-1.705420
H	4.583417	-1.389970	-0.011250
H	3.393614	-2.559247	-0.633889
C	3.097384	0.906400	-0.414905
H	2.348655	1.651001	-0.152207
H	3.965236	1.047335	0.248055
H	3.459196	1.124136	-1.431665
C	1.407052	-1.017413	-0.031281
H	1.343236	-2.109561	-0.112045
H	-0.344205	-0.739185	-1.831941
H	-0.828911	1.925235	-2.059040
C	0.932729	1.126626	2.255259
H	0.786817	2.109314	2.725315
H	0.786911	0.362376	3.029432
H	1.968464	1.067320	1.911903
H	-2.609313	-0.572491	1.567821
C	-3.098994	1.014236	-0.550520
H	-2.930205	1.229141	0.513969
H	-2.879211	1.932428	-1.106774
H	-4.168717	0.804350	-0.675809

I8 G = -585.972025

C	0.223996	1.011049	0.669318
C	0.539907	-0.321827	0.077119
C	3.081743	-0.495271	-0.123617
C	-0.429905	1.959806	-0.401981
C	-0.652272	-1.088699	-0.387756
C	-1.718387	-0.184157	-1.141176
C	-1.160929	1.197322	-1.526723
H	-0.311120	-1.897851	-1.050189

H	-1.100159	2.635608	0.143271
H	-0.630938	0.747146	1.323228
H	0.362953	2.588627	-0.833545
H	-0.449215	1.042650	-2.353729
C	-1.461343	-1.754152	0.796809
C	-3.046290	-0.277423	-0.330873
H	-3.879304	-0.270684	-1.051683
C	-2.928064	-1.666922	0.333625
H	-3.627793	-1.809709	1.168203
H	-3.124504	-2.458376	-0.405981
H	-1.112605	-2.777794	0.980195
C	4.228524	-1.454858	-0.030375
H	4.652692	-1.631008	-1.033177
H	5.042678	-1.006941	0.559704
H	3.947796	-2.419245	0.405393
C	3.431050	0.900010	-0.533973
H	2.571366	1.504764	-0.827571
H	3.979925	1.405089	0.276708
H	4.131818	0.856198	-1.381786
C	1.788799	-0.982935	0.019413
H	1.709902	-2.077033	-0.001895
H	-1.903799	-0.722280	-2.084252
H	-1.969846	1.822256	-1.931427
C	1.234030	1.728112	1.575156
H	0.684522	2.380950	2.264698
H	1.815953	1.018465	2.177469
H	1.927804	2.369076	1.022525
H	-1.312387	-1.188138	1.727403
C	-3.324977	0.844730	0.681306
H	-2.593908	0.875330	1.503439
H	-3.337955	1.831990	0.202215
H	-4.309180	0.692214	1.143148

Scheme S20

Step G8 – I8

G8 G = -585.973045

C	-0.373075	1.922604	-0.120735
C	0.208304	0.625657	0.378933
C	-2.285731	-0.998973	-0.370158
C	0.789671	2.576451	-0.931764
C	-0.379414	-0.366234	1.285802
C	1.606356	0.487454	-0.076460
C	1.720638	1.409300	-1.312832
H	-1.150031	0.048437	1.948575
H	0.416789	3.138579	-1.795380
H	-1.181503	1.652446	-0.824030
H	1.323590	3.285910	-0.280185
H	2.752562	1.720338	-1.512408
C	0.700930	-1.167641	2.045163

C	2.317232	-0.886793	-0.016044
H	3.368829	-0.663697	0.220408
C	1.749810	-1.812354	1.114204
H	1.306544	-2.706014	0.651063
H	2.577055	-2.186824	1.731464
H	1.184117	-0.477178	2.754418
C	-2.781378	-1.930990	-1.444228
H	-3.639956	-2.512122	-1.073788
H	-3.153008	-1.352818	-2.305171
H	-2.012040	-2.629164	-1.792760
C	-3.264990	0.039305	0.098503
H	-3.337872	0.853460	-0.643555
H	-4.272803	-0.391544	0.172010
H	-3.005216	0.490199	1.061406
C	-1.021527	-1.152357	0.122692
H	-0.392862	-1.919790	-0.325861
H	2.035334	1.088988	0.776420
H	1.347523	0.879280	-2.201723
C	-0.968832	2.821035	0.982552
H	-1.325683	3.755035	0.531523
H	-1.814313	2.351918	1.498684
H	-0.205203	3.076777	1.729055
H	0.210377	-1.947450	2.642962
C	2.315785	-1.598097	-1.383751
H	1.296445	-1.768410	-1.759266
H	2.860345	-1.021967	-2.142134
H	2.808298	-2.574591	-1.292993

G8-I8-TS G = -585.943287, T = -120

C	-0.130007	2.036811	-0.233440
C	-0.028260	0.537648	0.212774
C	-2.184457	-0.596597	-0.586940
C	1.076831	2.230784	-1.214655
C	-0.286903	0.172222	1.584385
C	1.559764	0.200398	0.040439
C	1.825596	0.882193	-1.309960
H	-0.666850	0.897940	2.313235
H	0.748142	2.580491	-2.200775
H	-1.078618	2.164053	-0.772999
H	1.747598	3.002324	-0.810696
H	2.905073	1.009603	-1.469945
C	0.129740	-1.136449	2.104916
C	1.947606	-1.283244	0.292082
H	2.844232	-1.248262	0.932413
C	0.850321	-2.037350	1.104171
H	0.107191	-2.450748	0.405691
H	1.292181	-2.892065	1.632609
H	0.762010	-0.889068	2.987080
C	-2.889262	-1.452879	-1.608934
H	-3.451784	-2.256761	-1.108366
H	-3.631748	-0.856461	-2.161181

H	-2.199878	-1.906680	-2.330032
C	-3.074308	0.035872	0.451140
H	-3.969072	0.468348	-0.018506
H	-3.432790	-0.721348	1.165927
H	-2.580600	0.830397	1.030098
C	-0.839865	-0.417139	-0.669460
H	-0.301886	-0.945848	-1.456370
H	2.051669	0.817856	0.809226
H	1.445610	0.267625	-2.137352
C	-0.094285	3.039252	0.928430
H	-0.047532	4.062438	0.533994
H	-0.989429	2.983622	1.564937
H	0.794817	2.898674	1.562916
H	-0.736000	-1.636387	2.582720
C	2.323345	-2.088494	-0.963658
H	1.476414	-2.204375	-1.653223
H	3.146836	-1.616587	-1.513129
H	2.649274	-3.096954	-0.676024

I8 G = -585.988758

C	-0.405035	1.880316	-0.518523
C	-0.814320	0.467782	-0.211314
C	-2.840031	-0.989609	0.268600
C	0.670699	2.298061	0.549608
C	0.268869	-0.549418	-0.180370
C	1.456858	-0.098397	0.766817
C	1.871967	1.356036	0.516772
H	-0.104620	-1.488563	0.241638
H	0.964156	3.334184	0.331232
H	0.144841	1.833092	-1.476960
H	0.196309	2.297794	1.544140
H	2.597329	1.656954	1.286850
C	0.987352	-0.871685	-1.538337
C	2.528906	-1.154863	0.392064
H	2.180074	-2.118115	0.804517
C	2.449259	-1.237849	-1.151408
H	3.150286	-0.523631	-1.606450
H	2.730968	-2.233711	-1.516686
H	0.465037	-1.689810	-2.052581
C	-4.226061	-0.970690	0.831828
H	-4.952530	-1.121958	0.014408
H	-4.369560	-1.825605	1.510613
H	-4.473950	-0.039115	1.351019
C	-2.378361	-2.318501	-0.249795
H	-3.234454	-2.887979	-0.635866
H	-1.612902	-2.251306	-1.026593
H	-1.974660	-2.913542	0.587677
C	-2.143438	0.216700	0.193994
H	-2.740798	1.095649	0.446849
H	1.139190	-0.201040	1.817119
H	2.386794	1.453342	-0.451794

C	-1.518451	2.927242	-0.643399
H	-1.086523	3.870713	-0.998610
H	-2.292806	2.623036	-1.359261
H	-1.997058	3.137702	0.323582
H	0.958329	-0.009696	-2.216271
C	3.931595	-0.880102	0.945078
H	4.352435	0.047763	0.534211
H	3.922424	-0.797738	2.040425
H	4.612838	-1.698561	0.677098

Step I8 – J8

I8 G = -585.983913

C	0.324447	1.949397	0.473671
C	0.832260	0.570289	0.159398
C	3.043499	-0.688196	-0.117131
C	-1.156335	1.958374	0.884566
C	-0.166638	-0.471755	-0.156344
C	-1.529370	-0.012030	-0.759482
C	-2.068646	1.327623	-0.182458
H	0.253458	-1.232886	-0.823089
H	-1.269327	1.434949	1.845984
H	0.945685	2.373053	1.281202
H	-1.460139	2.998916	1.063721
C	-0.561136	-1.227893	1.201758
C	-2.409438	-1.252498	-0.430711
H	-2.001897	-2.091148	-1.025394
C	-2.076221	-1.521960	1.049045
H	-2.659057	-0.847629	1.694565
H	-2.315180	-2.549456	1.354382
H	0.042321	-2.139122	1.297229
C	4.524843	-0.523758	-0.005990
H	4.998305	-0.790425	-0.965635
H	4.839427	0.482309	0.288555
H	4.918072	-1.255038	0.720741
C	2.579456	-2.040871	-0.576096
H	2.089270	-1.964637	-1.559652
H	3.421294	-2.733731	-0.676562
H	1.849108	-2.477308	0.115736
C	2.237588	0.403562	0.201710
H	2.786683	1.293778	0.522704
H	-1.409529	0.091709	-1.848411
C	0.584237	2.833371	-0.793660
H	0.179590	3.834916	-0.600084
H	1.654464	2.922983	-1.011948
H	0.084889	2.422762	-1.679057
H	-0.345381	-0.613836	2.084727
C	-3.901845	-1.141010	-0.752166
H	-4.401864	-0.374507	-0.144757
H	-4.063565	-0.895272	-1.810344
H	-4.406009	-2.095039	-0.549019

H	-2.205479	2.042318	-1.005586
H	-3.065336	1.186482	0.253322

I8-J8-TS G = -585.889524, T = -1253

C	0.449808	1.954324	0.587635
C	0.720954	0.559622	0.020090
C	2.861631	-0.795069	-0.237619
C	-1.049002	2.073449	0.964577
C	-0.246111	-0.596914	0.371716
C	-1.344376	-0.000868	-0.450314
C	-1.943059	1.319374	-0.031728
H	0.145058	-1.441592	-0.218024
H	-1.202473	1.649252	1.965371
H	1.037637	1.994639	1.523305
H	-1.342799	3.128849	1.021800
C	-1.039088	-1.237639	1.521226
C	-2.221515	-1.157086	-0.837141
H	-1.651120	-1.882019	-1.436523
C	-2.292223	-1.746957	0.677859
H	-3.229676	-1.401807	1.131130
H	-2.322841	-2.841018	0.613610
H	-0.517525	-2.065002	2.016857
C	4.212111	-0.874443	-0.899174
H	4.338616	-1.860630	-1.373854
H	4.365539	-0.097489	-1.656357
H	5.013254	-0.796417	-0.147309
C	2.575128	-1.899572	0.747623
H	2.379372	-2.844625	0.215969
H	3.468949	-2.079484	1.362056
H	1.740609	-1.693981	1.422272
C	2.053410	0.277752	-0.507866
H	2.464392	1.042369	-1.169067
H	-0.044825	0.628003	-1.128043
C	0.925724	3.095295	-0.329896
H	0.683507	4.061979	0.128013
H	2.008797	3.074693	-0.496334
H	0.425627	3.052036	-1.308967
H	-1.351370	-0.525327	2.293421
C	-3.580067	-0.900176	-1.482695
H	-4.198917	-0.218324	-0.884906
H	-3.465116	-0.471059	-2.486529
H	-4.130062	-1.844411	-1.584447
H	-2.197957	1.907033	-0.927370
H	-2.916636	1.097778	0.438168

J8 G = -585.962033

C	0.170978	1.575027	0.301756
C	0.555145	0.634335	-0.876258
C	2.700723	-0.539410	0.050975
C	-1.154926	2.301244	-0.007836
C	-0.576107	-0.482962	-1.024873

C	-1.894064	0.006828	-0.615398
C	-2.237578	1.418071	-0.776705
H	-0.665004	-0.653778	-2.120481
H	-1.618002	2.639209	0.927795
H	0.003066	0.954927	1.199893
H	-0.964231	3.189636	-0.624489
C	-0.426603	-1.865844	-0.329902
C	-2.672998	-1.026571	0.104991
H	-3.719703	-1.030684	-0.244873
C	-1.889248	-2.337298	-0.169134
H	-2.017298	-3.074083	0.632779
H	-2.249603	-2.792325	-1.103506
H	0.193316	-2.555727	-0.910470
C	4.048140	-1.116812	-0.318113
H	4.110224	-2.174170	-0.015136
H	4.258024	-1.050363	-1.391891
H	4.852341	-0.595617	0.224456
C	2.391197	-0.616717	1.526228
H	2.443912	-1.663958	1.863182
H	3.158635	-0.078335	2.103830
H	1.415161	-0.217639	1.811485
C	1.915889	-0.011491	-0.924480
H	2.317998	-0.088280	-1.939258
H	0.487285	1.256899	-1.785516
C	1.266986	2.606274	0.602694
H	0.952405	3.287148	1.404306
H	2.199492	2.119980	0.907612
H	1.482833	3.211339	-0.289679
H	0.054457	-1.741500	0.649372
C	-2.714861	-0.639860	1.626031
H	-1.705990	-0.609058	2.053714
H	-3.201112	0.329232	1.786766
H	-3.289497	-1.413457	2.148989
H	-2.135772	1.665026	-1.847504
H	-3.258963	1.656706	-0.461230

Scheme S21

For **I8 – J8** cf. cartesian coordinates for Scheme S20.

Scheme S22

Step H9 – I9

H9 G = -585.975557

C	0.265667	-2.175462	0.097301
C	-0.515523	-0.869432	-0.268078
C	-3.001242	-0.287222	-0.030469
C	-1.830374	-0.734102	0.470792
C	1.640544	-2.259301	-0.579496
C	0.362467	0.316376	-0.287931

C	1.774710	0.248570	-0.722008
C	2.535079	-1.036559	-0.275834
H	3.444740	-1.104795	-0.891453
H	1.510230	-2.357080	-1.669844
H	-0.365350	-3.026574	-0.187192
C	2.956155	-0.964437	1.202152
H	2.093582	-0.821427	1.870054
H	3.451867	-1.898705	1.495267
H	3.662999	-0.147005	1.388589
H	2.151334	-3.172684	-0.242575
H	-1.805688	-1.087392	1.506089
H	-0.725324	-0.951692	-1.363685
H	1.627912	0.147157	-1.826793
H	0.366410	-2.213180	1.192310
C	-0.020430	1.696907	0.049831
C	2.353233	1.669078	-0.485633
H	3.123572	1.923930	-1.221772
H	2.815598	1.733212	0.508701
C	1.101856	2.570568	-0.570733
H	0.856951	2.789902	-1.621013
H	1.216767	3.526010	-0.045768
H	-1.051911	1.934370	-0.246033
C	0.010906	1.739959	1.631810
H	-0.138659	2.790517	1.909762
H	0.973489	1.409824	2.037644
H	-0.793920	1.133546	2.056153
C	-3.197272	0.204622	-1.445988
H	-3.952091	-0.409743	-1.959776
H	-2.287175	0.202390	-2.059756
H	-3.592524	1.232241	-1.443350
C	-4.242952	-0.264774	0.829786
H	-4.649765	0.755883	0.899611
H	-4.056470	-0.634802	1.844997
H	-5.033517	-0.882643	0.376824

H9-I9-TS G = -585.967857, T = -666

C	0.287155	-2.233318	-0.078122
C	-0.415491	-0.879631	-0.043009
C	-2.919270	-0.321857	-0.056695
C	-1.804044	-0.891900	0.464531
C	1.699571	-2.227202	-0.686042
C	0.347131	0.299888	-0.275520
C	1.805827	0.257929	-0.727199
C	2.534884	-1.010326	-0.230287
H	3.510291	-1.055053	-0.738740
H	1.624628	-2.228734	-1.785465
H	-0.363574	-2.965758	-0.575234
C	2.781829	-1.008185	1.288424
H	1.858137	-0.840737	1.863083
H	3.199482	-1.971886	1.608298
H	3.495772	-0.228679	1.579833

H	2.203401	-3.162329	-0.406296
H	-1.928825	-1.556080	1.324750
H	-0.408681	-0.317915	-1.221046
H	1.790860	0.223624	-1.833502
H	0.323882	-2.541194	0.981655
C	-0.074385	1.727064	0.044100
C	2.332032	1.662131	-0.343865
H	3.182217	1.955906	-0.970021
H	2.671432	1.677867	0.701197
C	1.097815	2.567735	-0.524096
H	0.930343	2.770037	-1.593301
H	1.190701	3.533119	-0.012390
H	-1.036125	1.987490	-0.414859
C	-0.216610	1.863026	1.582870
H	-0.451058	2.906990	1.824003
H	0.714818	1.598278	2.099017
H	-1.021795	1.226917	1.967464
C	-2.975640	0.476295	-1.334406
H	-3.800695	0.114106	-1.964115
H	-2.058871	0.440250	-1.940047
H	-3.195126	1.534297	-1.120955
C	-4.249029	-0.518955	0.627867
H	-4.717107	0.455457	0.835675
H	-4.160792	-1.070386	1.570826
H	-4.943972	-1.061742	-0.031135

I9 G = -585.978704

C	0.250170	-1.939700	0.561839
C	-0.600963	-0.792972	0.139681
C	-3.073965	-0.163673	-0.135387
C	-1.998524	-0.926836	0.322876
C	1.283584	-2.255530	-0.556425
C	0.083352	0.388610	-0.424954
C	1.534471	0.220168	-1.026093
C	2.296127	-1.105296	-0.704118
H	2.905736	-1.329614	-1.595572
H	0.744719	-2.427749	-1.501121
H	-0.355810	-2.814067	0.829552
C	3.276224	-1.083229	0.482559
H	2.799040	-0.822186	1.437252
H	3.726593	-2.077538	0.604787
H	4.098045	-0.375873	0.322810
H	1.792359	-3.194856	-0.300463
H	-2.287492	-1.809993	0.900215
H	-0.545708	0.794875	-1.230365
H	1.373557	0.201378	-2.116117
H	0.805647	-1.639680	1.470247
C	0.170656	1.612076	0.597739
C	2.262629	1.546603	-0.658512
H	2.823627	1.953089	-1.509510
H	2.989016	1.380324	0.145638

C	1.169263	2.510946	-0.161756
H	0.656047	2.990113	-1.010413
H	1.565820	3.308627	0.481055
H	-0.821414	2.080591	0.671687
C	0.627149	1.247685	2.014284
H	0.682084	2.161462	2.620577
H	1.623466	0.789736	2.032639
H	-0.075421	0.564505	2.510717
C	-3.007026	1.027663	-1.046221
H	-4.007584	1.332377	-1.370262
H	-2.400388	0.820038	-1.939428
H	-2.542403	1.883619	-0.536745
C	-4.450717	-0.582895	0.267566
H	-4.976886	0.275059	0.718325
H	-4.472202	-1.430204	0.959899
H	-5.035022	-0.835747	-0.633878
